# The Influence of Genotype and Environment on Small RNA Profiles in Grapevine Berry

**DOI:** 10.3389/fpls.2016.01459

**Published:** 2016-10-05

**Authors:** Daniela Lopes Paim Pinto, Lucio Brancadoro, Silvia Dal Santo, Gabriella De Lorenzis, Mario Pezzotti, Blake C. Meyers, Mario E. Pè, Erica Mica

**Affiliations:** ^1^Institute of Life Sciences, Sant'Anna School of Advanced StudiesPisa, Italy; ^2^Department of Agricultural and Environmental Sciences-Production, Landscape, Agroenergy, University of MilanMilan, Italy; ^3^Laboratory of Plant Genetics, Department of Biotechnology, University of VeronaVerona, Italy; ^4^Donald Danforth Plant Science CenterSt. Louis, MO, USA; ^5^Division of Plant Sciences, University of Missouri–ColumbiaColumbia, MO, USA; ^6^Genomics Research Centre, Agricultural Research CouncilFiorenzuola d'Arda, Italy

**Keywords:** *Vitis vinifera*, Genotype x Environment (GxE), small RNAs, miRNAs, high throughput sequencing, berry

## Abstract

Understanding the molecular mechanisms involved in the interaction between the genetic composition and the environment is crucial for modern viticulture. We approached this issue by focusing on the small RNA transcriptome in grapevine berries of the two varieties Cabernet Sauvignon and Sangiovese, growing in adjacent vineyards in three different environments. Four different developmental stages were studied and a total of 48 libraries of small RNAs were produced and sequenced. Using a proximity-based pipeline, we determined the general landscape of small RNAs accumulation in grapevine berries. We also investigated the presence of known and novel miRNAs and analyzed their accumulation profile. The results showed that the distribution of small RNA-producing loci is variable between the two cultivars, and that the level of variation depends on the vineyard. Differently, the profile of miRNA accumulation mainly depends on the developmental stage. The vineyard in Riccione maximizes the differences between the varieties, promoting the production of more than 1000 specific small RNA loci and modulating their expression depending on the cultivar and the maturation stage. In total, 89 known vvi-miRNAs and 33 novel vvi-miRNA candidates were identified in our samples, many of them showing the accumulation profile modulated by at least one of the factors studied. The *in silico* prediction of miRNA targets suggests their involvement in berry development and in secondary metabolites accumulation such as anthocyanins and polyphenols.

## Introduction

The ability of a genotype to produce different phenotypes as a function of environmental cues is known as phenotypic plasticity (Bradshaw, 1965; Sultan, 2000; Pigliucci, 2001; Gratani, 2014). Phenotypic plasticity is considered one of the main processes by which plants, as sessile organisms, can face and adapt to the spatio-temporal variation of environmental factors (Nicotra et al., 2010; Palmer et al., 2012; Gratani, 2014).

Grapevine (*Vitis vinifera* L.) berries are characterized by high phenotypic plasticity (Dal Santo et al., 2013) and a genotype (cultivar or clone) can present variability within berries, among berries in a cluster, and among vines (Gray, 2002; Keller, 2010). Berry phenotypic traits, such as the content of sugars, acids, phenolic, anthocyanins, and flavor compounds, are the result of cultivar (G) and environmental influences (E), and often strong G × E interactions (Sadras et al., 2007). Although grapevine plasticity in response to environmental conditions and viticulture practices may provide advantages related to the adaptation of a cultivar to specific growing conditions, it may also cause irregular ripening (Selvaraj et al., 1994) and large inter-seasonal fluctuations (Clingeleffer, 2010), which are undesirable characteristics for wine making (Keller, 2010).

Due to its complex nature, the study of phenotypic plasticity is challenging and the mechanisms by which the genes affecting plastic responses operate are poorly characterized (Holloway, 2002; DeWitt and Scheiner, 2003; Nicotra et al., 2010; Gianoli and Valladares, 2012; Gratani, 2014). In fact it is often difficult to assess the performance of different phenotypes in different environments (Schmitt, 1993; Schmitt et al., 1999; Callaway et al., 2003).

It has been suggested that genetic and epigenetic regulation of gene expression might be at the basis of phenotypic plasticity through the activation of alternative gene pathways (Schlichting and Pigliucci, 1993; Pigllucci, 1996) or multiple genes (Lind et al., 2015). Epigenetics has been proposed as crucial in shaping plant phenotypic plasticity, putatively explaining the rapid and reversible alterations in gene expression in response to environmental changes. This fine-tuning of gene expression can be achieved through DNA methylation, histone modifications and chromatin remodeling (Goldberg et al., 2007; Geng et al., 2013; Duncan et al., 2014).

Small non-coding RNAs (small ncRNAs) are ubiquitous and adjustable repressors of gene expression across a broad group of eukaryotic species and are directly involved in controlling, in a sequence specific manner, multiple epigenetic phenomena such as RNA-directed DNA methylation and chromatin remodeling (Bernstein and Allis, 2005; Fagegaltier et al., 2009; Ha et al., 2009; Swami, 2010; Burkhart et al., 2011; Castel and Martienssen, 2013; Duncan et al., 2014) and might play a role in genotype by environment (GxE) interactions. In plants, small ncRNAs are typically 20–24 nt long RNA molecules and participate in a wide series of biological processes controlling gene expression via transcriptional and post-transcriptional regulation (Finnegan and Matzke, 2003; Kim, 2005; Chen, 2009; Guleria et al., 2011; Lelandais-Briere et al., 2012; Matsui et al., 2013). Moreover, small RNAs have been recently shown to play an important role in plants environmental plasticity (Formey et al., 2014; Borges and Martienssen, 2016).

Fruit maturation, the process that starts with fruit-set and ends with fruit ripening (Coombe, 1976), has been largely investigated in fleshy fruits such as tomato and grapevine. These studies highlighted, among others, the vast transcriptomic reprogramming underlying the berry ripening process (Guillaumie et al., 2011; Matas et al., 2011; Lijavetzky et al., 2012), the extensive plasticity of berry maturation in the context of a changing environment (Dal Santo et al., 2013; Gapper et al., 2014), and the epigenetic regulatory network which contributes to adjust gene expression to internal and external stimuli (Zhong et al., 2013; Liu et al., 2015). In particular, small RNAs, and especially microRNAs (miRNA), are involved, among others, in those biological processes governing fruit ripening (Karlova et al., 2013; Kullan et al., 2015).

In this work, we assessed the role of small ncRNAs in the plasticity of grapevine berries development, by employing next-generation sequencing. We focused on two cultivars of *Vitis vinifera*, Cabernet Sauvignon, and Sangiovese, collecting berries at four different developmental stages in three Italian vineyards, diversely located. First, we described the general landscape of small RNAs originated from hotspots present along the genome, examining their accumulation according to cultivars, environments and developmental stages. Subsequently, we analyzed miRNAs, identifying known and novel miRNA candidates and their distribution profiles in the various samples. Based on the *in silico* prediction of their targets, we suggest the potential involvement of this class of small RNAs in GxE interactions. The results obtained provide insights into the complex molecular machinery that connects the genotype and the environment.

## Materials and methods

### Plant material

Two *V. vinifera* varieties Sangiovese (SG), a red Italian grape variety, and Cabernet Sauvignon (CS), an international variety, were grown side by side in three different Italian locations, representing traditional areas of Sangiovese cultivation in Italy with a long-standing winemaking tradition.

In order to reduce factors of variation, the age of the plants (between 10 and 12 years old), the clone type (Sangiovese clone R5 and Cabernet Sauvignon clone VCR23), the rootstock (*Vitis berlandieri* × *Vitis riparia*), the cultivation techniques (training system: low cordon; planting space: 2.40 × 0.8 m) and the health status were the same among all the locations.

The vineyards were located in Bolgheri (Bol), a coastal area of Tuscany, 50 m asl (above sea level) [GPS coordinates: SG 43.194090, 10.625186, CS 43.194622, 10.624392], in Montalcino (Mont) a mountain area of Tuscany, 195 m asl; [GPS coordinates: SG 42.980669, 11.433708, CS 42.985091, 11.435853] and in Riccione (Ric), a plain area of Emilia Romagna, 111 m asl; [GPS coordinates: SG 43.945261, 12.647235, CS 43.944372, 12.648995]. Further details on the environmental conditions of the vineyards are provided in Supplementary Figure 1.

Berries from four developmental stages were collected in two biological replicates, during the 2011 growing season, for a total of 48 samples (Table 1). The four sampled stages corresponded to pea size (ps), representing the first stage of berry development in this experimental plan, bunch closure (bc) also known as Lag Phase, 19–20 °Brix (19), which corresponds to 50% of sugar accumulation in berries, and harvest (hv), when the berries are fully ripened and the onset of sugar accumulation is over. About 200 berries per each developmental berry stage were sampled from upper, central and lower part of cluster, both from sun-exposed and shaded side and split in two biological replicates. Per each vineyard, the berries were collected from about 20 vines selected in a single uniform row and immediately frozen in liquid nitrogen and stored at −80°C prior to analysis.

**Table 1:** **List of berry samples of *Vitis vinifera* used for the construction of the small RNA libraries**.

**Vineyard**	**Variety**	**Developmental Stages**	**Replicate**	**Library Codes**
Montalcino	Cabernet Sauvignon	Pea size	1	Mont_CS_ps_1
			2	Mont_CS_ps_2
		Bunch closure	1	Mont_CS_bc_1
			2	Mont_CS_bc_2
		19 °Brix	1	Mont_CS_19_1
			2	Mont_CS_19_2
		Harvest	1	Mont_CS_hv_1
			2	Mont_CS_hv_2
Montalcino	Sangiovese	Pea size	1	Mont_SG_ps_1
			2	Mont_SG_ps_2
		Bunch closure	1	Mont_SG_bc_1
			2	Mont_SG_bc_2
		19 °Brix	1	Mont_SG_19_1
			2	Mont_SG_19_2
		Harvest	1	Mont_SG_hv_1
			2	Mont_SG_hv_2
Bolgheri	Cabernet Sauvignon	Pea size	1	Bol_CS_ps_1
			2	Bol_CS_ps_2
		Bunch closure	1	Bol_CS_bc_1
			2	Bol_CS_bc_2
		19 °Brix	1	Bol_CS_19_1
			2	Bol_CS_19_2
		Harvest	1	Bol_CS_hv_1
			2	Bol_CS_hv_2
Bolgheri	Sangiovese	Pea size	1	Bol_SG_ps_1
			2	Bol_SG_ps_2
		Bunch closure	1	Bol_SG_bc_1
			2	Bol_SG_bc_2
		19 °Brix	1	Bol_SG_19_1
			2	Bol_SG_19_2
		Harvest	1	Bol_SG_hv_1
			2	Bol_SG_hv_2
Riccione	Cabernet Sauvignon	Pea size	1	Ric_CS_ps_1
			2	Ric_CS_ps_2
		Bunch closure	1	Ric_CS_bc_1
			2	Ric_CS_bc_2
		19 °Brix	1	Ric_CS_19_1
			2	Ric_CS_19_2
		Harvest	1	Ric_CS_hv_1
			2	Ric_CS_hv_2
Riccione	Sangiovese	Pea size	1	Ric_SG_ps_1
			2	Ric_SG_ps_2
		Bunch closure	1	Ric_SG_bc_1
			2	Ric_SG_bc_2
		19 °Brix	1	Ric_SG_19_1
			2	Ric_SG_19_2
		Harvest	1	Ric_SG_hv_1
			2	Ric_SG_hv_2

The libraries were named using the initials of the vineyard where the berries were collected, followed by the initial of the cultivar and the developmental stage. For example, the sample containing berries of Sangiovese, collected in Montalcino at pea size, was named Mont_SG_ps.

### RNA extraction and small RNA libraries construction

RNA extraction was performed as described in Kullan et al. (2015). Briefly, total RNA was extracted from 200 mg of ground berries pericarp tissue (entire berries without seeds) using 1 ml of Plant RNA Isolation Reagent (Life Technologies) following manufacturer's recommendations.

The small RNA fraction was isolated from the total RNA using the mirPremier® microRNA Isolation kit (Sigma-Aldrich) and dissolved in DEPC water. All the steps suggested in the technical bulletin for small RNA isolation of plant tissues were followed except the “Filter Lysate” step, which was omitted. The quality and quantity of small RNAs were evaluated by a NanoDrop 1000 spectrometer (Thermo Fisher Scientific), and their integrity assessed by an Agilent 2100 Bioanalyzer using a small RNA chip (Agilent Technologies) according to the manufacturer's instructions.

Small RNA libraries were prepared using the TruSeq Small RNA Sample Preparation Kit (Illumina®), following all manufacturers' instructions. Forty-eight bar-coded small RNA libraries were constructed starting from 50 ng of small RNAs. The quality of each library was assessed using an Agilent DNA 1000 chip for the Agilent 2100 Bioanalyzer. Libraries were grouped in pools with six libraries each (6-plex).

The pools of libraries were sequenced on an Illumina Hiseq 2000 at IGA Technology Services (Udine, Italy).

The sequencing data were submitted to GEO–NCBI under the accession number GSE85611.

### Bioinformatics analysis of sequencing data

Adaptor sequences were trimmed and only reads ranging from 18 to 34 nt in length after adapter removal were kept. Retained reads were mapped to the reference *Vitis vinifera* L. genomic sequence V1 (PN40024, Jaillon et al., 2007) using Bowtie (Langmead et al., 2009) and reads perfectly aligned to the genome were retained. Reads matching rRNAs, tRNAs, snRNAs, and snoRNAs were excluded.

Read counts were normalized by the linear count scaling method TP4M (transcripts per 4 million), in order to reduce sequencing bias and to allow the comparison of small RNA accumulation from different libraries. The normalized abundance was calculated as:

$$\begin{array}{l} \overset{TPM}{abundance}=\left[\frac{raw\;value}{\begin{array}{l} (total\;genome\;matches-t/r/sn/snoRNA\\ /chloroplast\;mitochondria\;matches) \end{array}}\right]\\ \;\times n\_base \end{array}$$

where *n* base is 4,000,000.

To perform the clustering analysis, the “hits-normalized-abundance” (HNA) values were calculated as:

$$HNA=\frac{TP\text{4}M}{Hits}$$

where *TP*4*M* is the normalized abundance of each small RNA sequence mapping in a giving cluster and a *Hit* is defined as the number of loci at which a given sequence perfectly matches the genome.

One database was produced using the grapevine genome, and made available on the website (https://mpss.danforthcenter.org/dbs/index.php?SITE=grape_sRNA_GxE), in order to store and assist the visualization of all the sequenced libraries.

### Static clustering analysis

The static clustering analysis was carried out as previously described by Lee et al. (2012), using a proximity-based pipeline built with custom Perl and database scripts (McCormick et al., 2011) and MySQL database queries, to group and quantify clusters of small RNAs. Briefly, the grapevine genome was divided into a series of windows of 500 bp, each window defined as a cluster. For every individual library, the small RNAs ranging from 21 to 24 nt and mapping in each cluster had their “hits-normalized-abundance” (HNA) summed up which determined the “cluster abundance.” The cluster abundance was averaged for the two replicates of each library. The clusters were annotated for gene and repeat information using the V1 annotation of the reference genome (Jaillon et al., 2007; Vitulo et al., 2014), allowing the characterization of specific small RNA-producing loci.

We set a selection criterion, by which a cluster was considered as expressed when the cluster abundance was equal or greater than 30 HNA. Additionally, when investigating the ratio between two cultivars in each environment (CS/SG ratio), only those clusters where the HNA of each library in the comparison was greater than or equal to 5 (library A ≥ 5 HNA and library B ≥ 5 HNA) and the sum of the cluster abundance of these same libraries was higher than 30 (library A + library B > 30) were selected.

All the clustering analyses were performed using only two developmental stages for each cultivar: bunch closure was used to represent “green tissues” (g) and 19 °Brix to represent “ripened tissues” (r).

### Identification of conserved miRNAs and prediction of novel candidates

The identification of annotated (conserved or known) and novel (or specie-specific) miRNAs was carried out applying a conservative and robust pipeline as described by Jeong et al. (2011) and Zhai et al. (2011), and successfully deployed in various published studies (Jeong et al., 2013; Xu et al., 2013; Arikit et al., 2014; Hu et al., 2015). Shortly, in order to recognize the conserved miRNAs, all small RNAs sequenced in the libraries were initially compared against all annotated vvi-miRNAs deposited in miRBase (version 20, Kozomara and Griffiths-Jones, 2014, http://www.mirbase.org/). Subsequently, the whole set of small RNAs passed through the five filters designed according to the properties of validated plant miRNAs and their precursors (Meyers et al., 2008), keeping track of known miRNAs throughout the filtering. The filters included, but were not limited to, minimum abundance threshold (≥30 TP4M), size range (18–26 nt), maximum hits to the grapevine genome (1–20), strand bias (sense/total ≥ 0.9), and abundance bias [(top1+top2)/total ≥ 0.7]. For each possible precursor found, the most abundant read was retained as the biologically active miRNA (also called “mature”) and in cases where both the 3′-end (3p) and the 5′-end (5p) reads were highly abundant (abundance greater than 200 TP4M), the two tags were kept.

All the known vvi-miRNAs identified by the pipeline were manually inspected, to ensure that the tags identified as known miRNAs were assigned correctly to their actual precursor, and to retrieve the most abundant isoform within the tags mapping in each precursor.

Regarding the novel miRNA candidates identified using this pipeline, only those for which the most abundant tag was 20, 21, or 22 nt were retained. They were compared with all the known mature plant miRNAs in miRBase (version 20) to identify homologs. Finally, novel candidates passed through a manual evaluation of precursor secondary structures, using the plant version of the UEA sRNA hairpin folding and annotation tool (Stocks et al., 2012) and the Mfold web server (Zuker, 2003), with default settings.

### miRNA accumulation and statistical analysis

A miRNA was considered as “expressed” only when the values of both biological replicates were greater than or equal to the threshold set at 10 TP4M. We defined a miRNA as “vineyard-, cultivar-, or stage-specific” when it was expressed only in a given vineyard, cultivar or one specific developmental stage.

Differentially expressed miRNAs were identified using the CLCbio Genomics Workbench (v.8, Qiagen, http://www.qiagenbioinformatics.com/products/clc-genomics-workbench/) using multiple comparison analysis. We loaded the total raw redundant reads from our 48 libraries in the CLCbio package and trimmed the adaptors, considering only reads between 18 and 34 nt. We annotated miRNAs against the user defined database, comprehending our set of 122 MIRNA loci and their corresponding mature sequences. For each library, the total counts of read perfectly mapping to the miRNA precursors was considered as the input of the expression analysis.

Given the main focus of our work, we aimed at identifying miRNAs differentially expressed between the two cultivars in the same environment and developmental stage (genotypic effect), or between the three vineyards in the same cultivar and in the same developmental stage (environmental effect). For this reason, we considered each developmental stage (12 libraries) and we performed the Empirical Analysis of digital gene expression (DGE), an implementation of the “Exact Test” present in the EdgeR Bioconductor package, as implemented in CLCbio software and estimating tagwise dispersion with pairwise comparisons and setting the significance threshold to FDR-adjusted *p* ≤ 0.05.

### Correlation analysis

The normalized reads (TP4M) of all miRNAs identified in this study and also the cluster abundances obtained from the static clustering analysis were submitted to another *ad-hoc* normalization [log_10_(1+TP4M) or log_10_(1+HNA)] for correlation analysis. This normalization was chosen because of the enormous range of abundance values that produced a log-unimodal distribution and may cause significant biases in the correlation analysis when performed using TP4M or HNA values. A unity was then added to the abundance value due to the presence of zero entries. After this addition, a value of zero still corresponds to zero of the log_10_(1+TP4M) function, thus making consistent the comparisons among profiles.

The dendrogram was generated using the function hclust and the Pearson correlation was calculated using the function cor in R, based on the log_10_(1+TP4M) or log_10_(1+HNA) values for miRNAs and sRNA-generating loci respectively. Pearson's correlation coefficients were converted into distance coefficients to define the height of the dendrogram.

Heat maps were produced using MeV (MultiExperiment Viewer; Eisen et al., 1998) based on TP4M values of miRNAs abundance. The Venn diagrams were produced using the function vennDiagram in R, based on the miRNA list for each cultivar, environment and developmental stage.

### Target prediction

miRNA targets were predicted using miRferno, a built-in, plant-focused target prediction module of the software sPARTA (small RNA-PARE Target Analyzer; Kakrana et al., 2014). miRferno was run using the greedy prediction mode (tarPred H) and a seed-free system (tarScore S) for target scoring. The prediction was done in genic regions (genomeFeature 0) of the whole 12X version of the grapevine genome (Jaillon et al., 2007). The fasta file with spliced exons for each GFF transcript (V2.1.mRNA.fa-downloaded from http://genomes.cribi.unipd.it/grape/) of the V2.1 annotation (Vitulo et al., 2014) was used as “feature file.” To reduce the number of false positives, only targets with a prediction score value smaller than 2.5 were retained (complete range of prediction score values: 0–10).

## Results

### High-throughput sequencing statistics

Small RNA libraries were constructed and sequenced for 48 samples of grapevine berries (Table 1). We obtained a total of 752,020,195 raw redundant reads (Supplementary Table 1). After adaptors trimming, 415,910,891 raw clean reads were recovered, ranging from 18 to 34 nt in length (Supplementary Table 1). Eliminating the reads mapping to rRNA, tRNA, snRNA, and snoRNA sequences, 199,952,950 reads represented by 20,318,708 distinct sequences, i.e., non-redundant sequences found in the 48 libraries (Supplementary Table 1), were perfectly mapped to the *V. vinifera* PN40024 reference genome (Jaillon et al., 2007).

The libraries were analyzed to assess the size distributions of mapped reads. Distinct peaks at 21- and 24-nt (Supplementary Figure 2) were observed in all the libraries. Consistent with previous reports in grapevine (Pantaleo et al., 2010) and other plant species (Moxon et al., 2008; Ge et al., 2013), the 21-nt peak was the highest, comprising a higher proportion of redundant reads, whereas the 24-nt peak was less abundant. A few exceptions regarding the highest peak in the small RNA size profile were observed: Ric_SG_ps had the highest peak at 24-nt whereas Mont_CS_ps and Mont_SG_bc did not show a clear difference between the 21- and the 24-nt peak.

Using the Pearson coefficients (Supplementary Table 2) we observed a strong association between the replicates as indicated by the high coefficients (ranging from 0.79 to 0.97).

To facilitate access and utilization of these data, we have incorporated the small RNAs into a website (https://mpss.danforthcenter.org/dbs/index.php?SITE=grape_sRNA_GxE). This website provides a summary of the library information, including samples metadata, mapped reads, and GEO accession numbers. It also includes pages for data analysis, such as quick summary of the abundances of annotated microRNAs from grapevine or other species. Small RNA-related tools are available, for example target prediction for user-specified small RNA sequences and matching criteria. Finally, and perhaps most importantly, a customized browser allows users to examine specific loci (genes or intergenic regions) for the position, abundance, length, and genomic context of matched small RNAs; with this information, coupled with the target prediction output, users can develop and assess hypotheses about whether there is evidence for small RNA-mediated regulation of grapevine loci of interest.

### General landscape of small RNAs distribution in grapevine berries in different environments

In order to investigate whether the overall distribution and accumulation of small RNA is affected by the interaction between different *V. vinifera* genotypes [Cabernet Sauvignon (CS) and Sangiovese (SG)] and environments [Bolgheri (Bol), Montalcino (Mont) and Riccione (Ric)], we investigated the regions in the grapevine genome from where a high number of small RNAs were being produced (sRNA-producing regions), by applying a proximity-based pipeline to group and quantify clusters of small RNAs as described by Lee et al. (2012).

**Figure 1:**
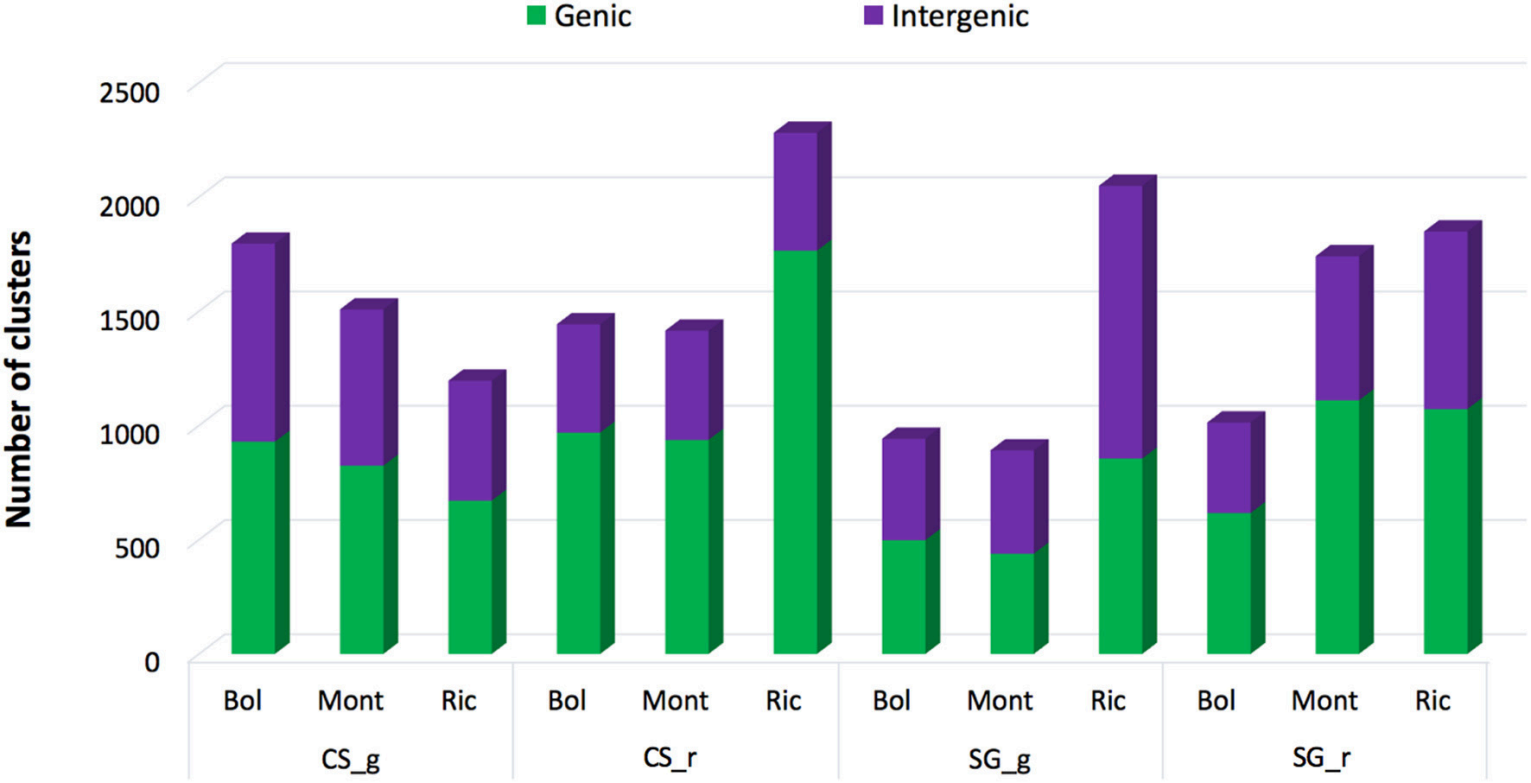
**Number of small RNA clusters expressed in ripened and green berries of grapevine collected from 2 different cultivars growing in 3 vineyards**. The proportion of clusters located in Genic and Intergenic regions (based on the V1 grapevine genome annotation) is shown in green and blue respectively. CS, Cabernet Sauvignon; SG, Sangiovese; g, green; r, ripened; Bol, Bolgheri; Mont, Montalcino; Ric, Riccione. Green corresponds to bunch closure and ripened corresponds to 19 °Brix developmental stages.

The nuclear grapevine genome was divided in 972,413 adjacent, non-overlapping, fixed-size (500 bp) windows or clusters. To determine the small RNA cluster abundance, we summed the hits-normalized-abundance (HNA) values of all the small RNAs mapping to each of the 500 bp clusters, for each library (for details, see Materials and Methods). To reduce the number of false positives, we considered a cluster as expressed when the cluster abundance was greater than the threshold (HNA = 30) for a given library, eliminating regions where few small RNAs were generated, possibly by chance. Libraries from bunch closure, representing green berries, and 19 °Brix representing ripened berries, where used in this analysis. From the 972,413 clusters covering the whole grapevine genome, 4408 (0.45%) were identified as expressed (sRNA-producing regions) in at least one sample. As showed in Figure 1, CS-derived libraries have a higher number of expressed clusters when compared to SG-derived libraries of the same developmental stage and from the same vineyard. The exceptions were the Sangiovese green berries collected in Riccione and Sangiovese ripened berries collected in Montalcino, which have a higher number of expressed clusters than the respective CS ones. The two cultivars show a completely different small RNA profile across environments. When Cabernet berries were green, a higher number of sRNA-generating regions were found active in Bolgheri than in Montalcino and Riccione. Differently, ripened berries had the highest number of sRNA-producing regions expressed in Riccione, while Bolgheri and Montalcino show a similar level of expressed clusters (Figure 1). Sangiovese green berries instead show the highest number of active sRNA-generating regions in Riccione, and this number is twice the number found in Bolgheri and Montalcino that is similar. Ripened berries collected in Montalcino and Riccione show almost the same high level of sRNA-generating clusters, whereas those collected in Bogheri present a lower number (Figure 1). We also noted that when cultivated in Bolgheri, neither Cabernet Sauvignon or Sangiovese change dramatically the number of expressed clusters during ripening, while in Riccione Cabernet Sauvignon shows a 2-fold increase of sRNA-producing clusters, which is not observed in Sangiovese.

Next, the small RNA-generating clusters were characterized on the basis of the genomic regions where they map, i.e., genic, intergenic and transposable elements. In general, when the berries were green, the numbers of sRNA-generating loci located in genic and intergenic regions were roughly equal in all environments and for both cultivars, except for Sangiovese berries collected in Riccione, which show a slight intergenic disposition of sRNA-producing regions (Figure 1). Differently, in ripened berries on average 65% of the sRNA-generating loci were in genic regions, indicating a strong genic disposition of the sRNA-producing clusters (Figure 1). The shift of sRNA-producing clusters from intergenic to mostly genic is more pronounced in Cabernet Sauvignon berries collected in Riccione, with an increase of approximately 20% of expressed clusters in genic regions (Figure 1) when berries pass from the green to the ripened stage.

**Figure 2:**
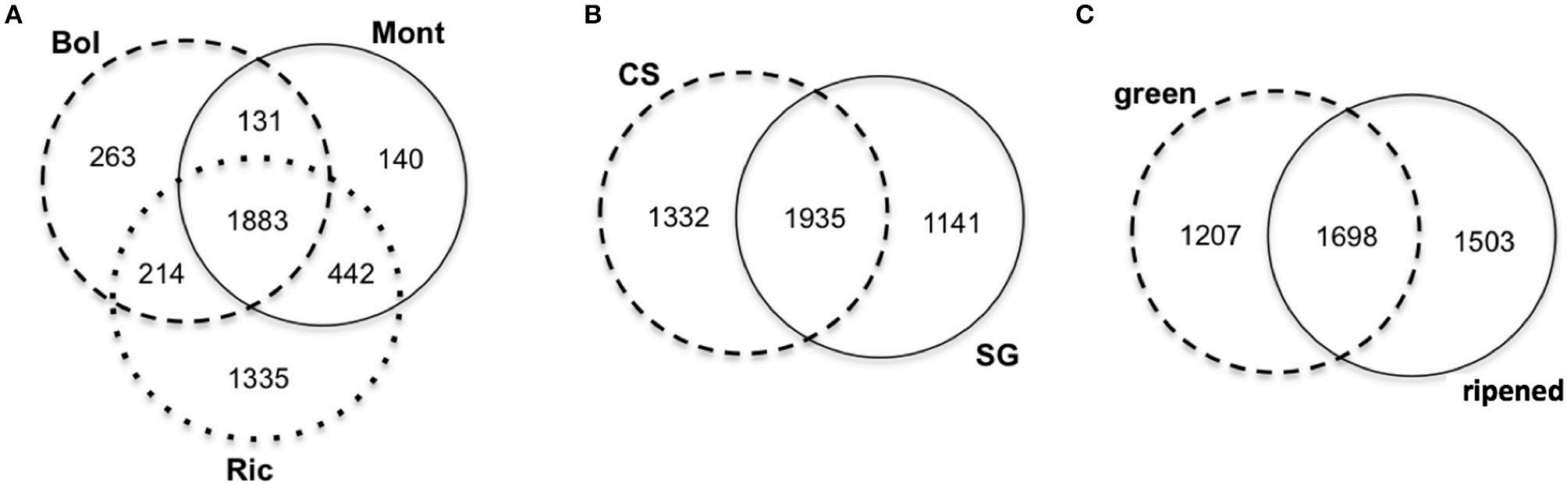
**Venn Diagrams comparing the sRNA-generating clusters of *Vitis vinifera* expressed by (A) environment, (B) cultivar, (C) developmental stage**. Bol, Bolgheri; Mont, Montalcino; Ric, Riccione; CS, Cabernet Sauvignon; SG, Sangiovese. Green corresponds to bunch closure and ripened corresponds to 19°Brix developmental stages.

When comparing the clusters abundance among libraries, we found that 462 clusters were expressed in all libraries. The remaining 3946 expressed clusters were either shared among groups of libraries or specific to unique libraries. Interestingly, 1335 (30.3%) of the 4408 expressed clusters were specific to Riccione-derived libraries (Figure 2A). The other two environments showed a much lower percentage of specific clusters, 263 (6%) and 140 (3.2%) in Bolgheri and Montalcino respectively (Figure 2A). Comparing the expressed clusters between cultivars or developmental stages, we did not observe a similar discrepancy of specific clusters toward one cultivar or developmental stage; roughly the same proportion of specific clusters was found for each cultivar (Figure 2B) and for each developmental stage (Figure 2C). Among the 1335 specific clusters of Riccione, 605 were specific to Cabernet Sauvignon ripened berries of and 499 to Sangiovese green berries. Other smaller groups of expressed clusters were identified as specific to one cultivar, one developmental stage or also one cultivar in a specific developmental stage.

**Figure 3:**
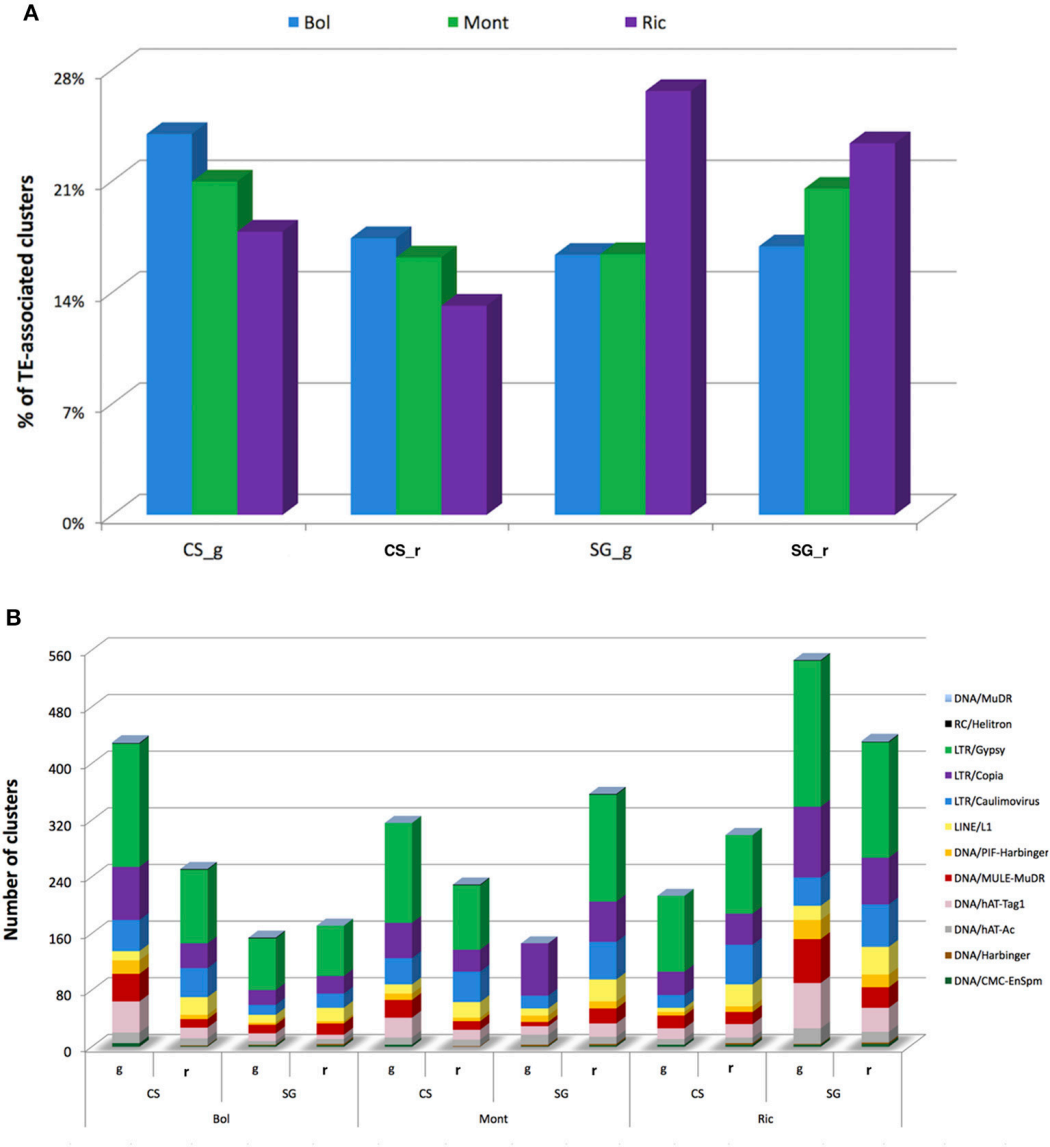
**Profile of small RNA-producing clusters expressed in ripened and green berries of grapevine collected from 2 different cultivars growing in 3 vineyards associated with Transposable Elements (TE)**. **(A)** Percentage of sRNA-producing clusters associated with TE in each sample. **(B)** Number of small RNA-generating clusters associated with different classes of TE in different samples. Bol, Bolgheri; Mont, Montalcino; Ric, Riccione; CS, Cabernet Sauvignon; SG, Sangiovese; g, green; r, ripened. Green corresponds to bunch closure and ripened corresponds to 19 °Brix developmental stages.

When comparing the expressed clusters with the presence of transposable elements (TE) annotated in the grapevine genome (V1), we noticed that approximately 23% of the sRNA-generating regions were TE-associated. Sangiovese green berries from Riccione have the highest proportion (26%) of TE-associated expressed clusters, while Cabernet Sauvignon ripened berries also from Riccione show the lowest proportion (13%) of TE-associated expressed clusters. Sangiovese berries (both green and ripened) have the highest percentage of expressed clusters located in TE when cultivated in Riccione, compared to the other two vineyards. Interestingly, Cabernet Sauvignon berries show the lowest proportion of TE-associated clusters when growing in Riccione (Figure 3A), independently from the berry stage.

In all the libraries, Long Terminal Repeat (LTR) retrotransposons were the most represented TE. More specifically, the gypsy family was the LTR class associated with the highest number of sRNA hotspots. The other classes of TE associated with the sRNA-generating regions can be visualized in Figure 3B.

### The distribution of sRNA-producing loci is variable between the two cultivars, and the level of variation depends on the vineyard

**Figure 4:**
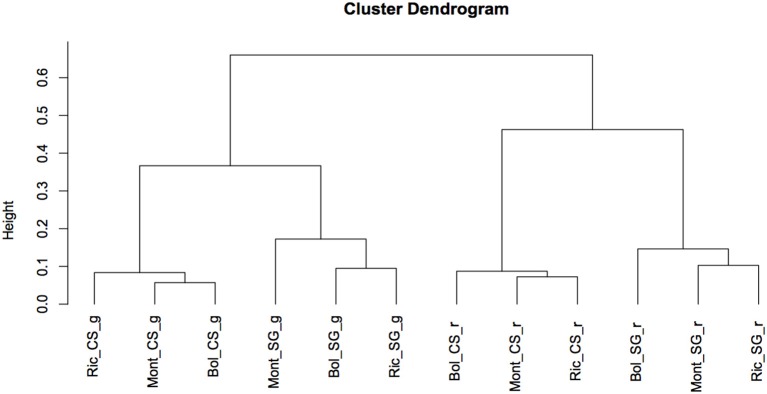
**Cluster dendrogram showing global relationships of small RNA-producing loci in 2 different grapevine cultivars, collected in different vineyards and developmental stages**. The Pearson correlation coefficients, calculated based on sum of HNA of small RNAs mapping to each loci, were converted into distance coefficients to define the height of the dendrogram. Bol, Bolgheri; Mont, Montalcino; Ric, Riccione; CS, Cabernet Sauvignon; SG, Sangiovese; g, green; r, ripened. Green corresponds to bunch closure and ripened corresponds to 19 °Brix developmental stages.

To determine the global relationship of small RNA-producing loci in the different environments, cultivars and developmental stages, we performed a hierarchical clustering analysis. As showed in Figure 4, the libraries clearly clustered according to the developmental stage and cultivar and not according to the environments. Ripened and green berries had their profile of sRNA-generating loci clearly distinguished from each other. Inside each branch of green and ripened samples, Cabernet Sauvignon and Sangiovese were also well separated, indicating that, the cultivar and the stage of development in which the berries were sampled modulate the outline of sRNA-producing loci more than the environment.

**Figure 5:**
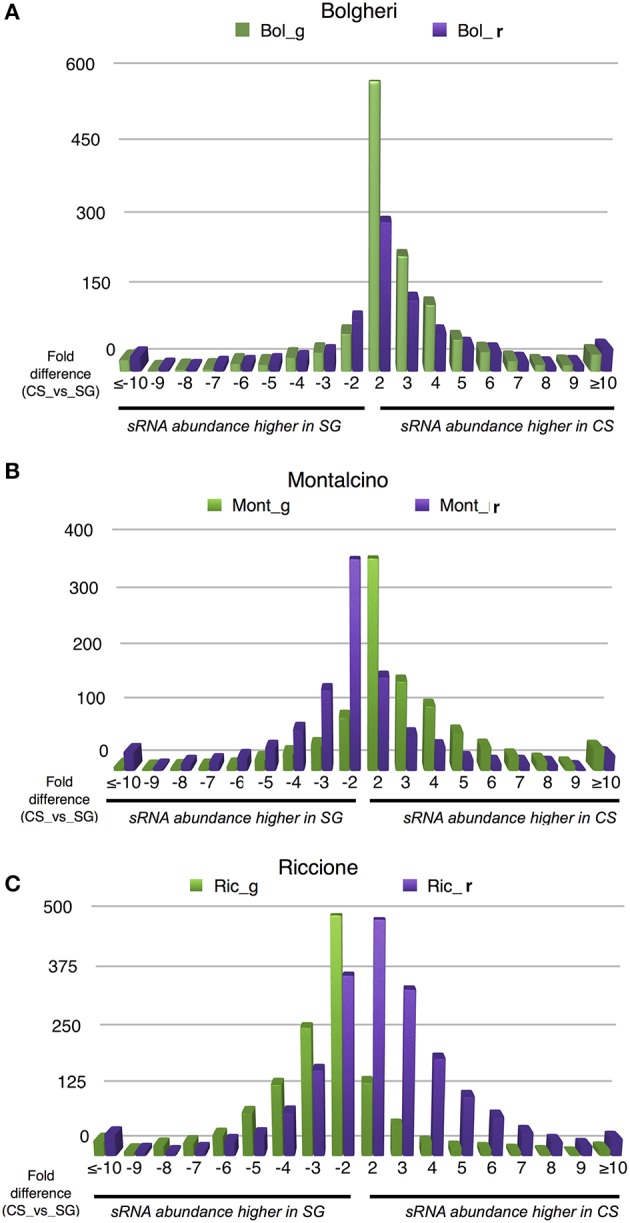
**Number of differentially regulated small RNA-generating clusters of ripened and green grape berries, collected from 2 varieties in 3 different vineyards**. The CS/SG ratios were calculated based on summed cluster abundances. **(A)** Data relative to berries collected in Bolgheri, **(B)** data relative to berries collected in Montalcino, **(C)** data relative to berries collected in Riccione. Bol, Bolgheri; Mont, Montalcino; Ric, Riccione; CS, Cabernet Sauvignon; SG, Sangiovese.

Notwithstanding the evidence that developmental stage and variety have the strongest effect in terms of distinguishing samples clustering, we were interested to verify the environmental influence on small RNA loci expression in the two cultivars. Thus, for each sRNA-generating cluster we calculated the ratio between cluster abundance in Cabernet Sauvignon and Sangiovese (CS/SG) in each environment and developmental stage, thereby revealing the genomic regions with regulated clusters, considering a 2-fold change threshold, a minimum abundance of 5 HNA in each library and a minimum sum of abundance of 30 HNA (library A ≥ 5 HNA and library B ≥ 5 HNA; library A + library B > 30 HNA; library A/library B ≥ 2). Figure 5 shows how different environments affect the production of small RNAs. In Bolgheri, regardless the developmental stage there were many clusters with a very high abundance level in Cabernet Sauvignon (Figure 5A). In Montalcino (Figure 5B) and even more in Riccione (Figure 5C) we also observed differences between the expressions of clusters in the two cultivars, with ripened and green berries showing an almost opposite profile in terms of number of clusters more expressed in Cabernet Sauvignon or Sangiovese. When the berries were green, in Montalcino Cabernet Sauvignon shows the highest number of up-regulated clusters, while in Riccione, Sangiovese has the highest number of up-regulated clusters. The opposite behavior was noticed in ripened berries, with Sangiovese having the highest number of up-regulated clusters in Montalcino and Cabernet Sauvignon in Riccione (Figures 5B,C).

**Figure 6:**
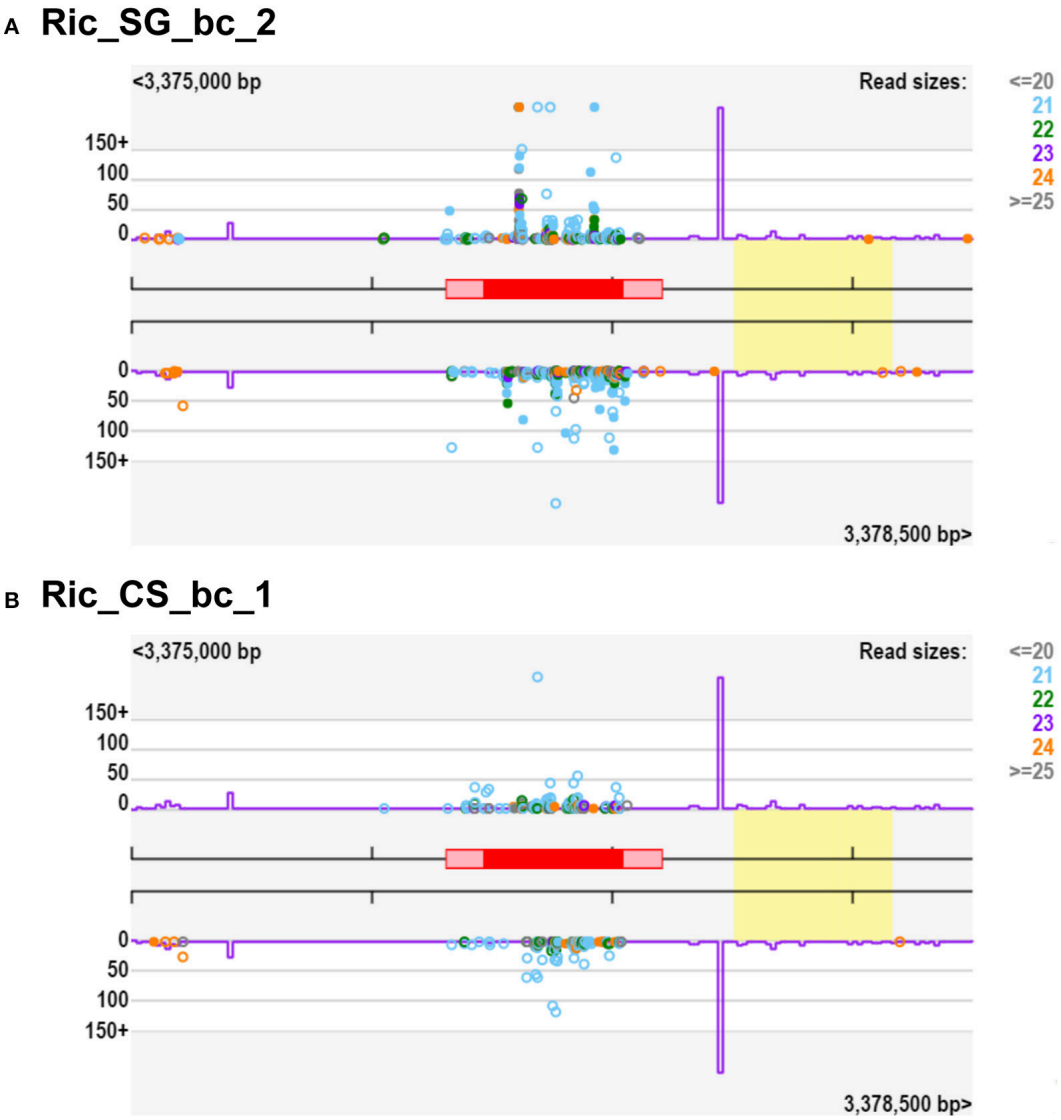
**Small RNA-producing loci of *Vitis vinifera* berries with 390-fold change considering SG/CS**. The gene in this locus (red box) codes for a protein annotated as “BURP domain-containing protein 3-like,” located on chromosome 4 (VIT_204s0008g04040). **(A)** Representation of small RNAs from Ric_SG_bc_2 mapping to this locus, **(B)** Representation of small RNAs from Ric_CS_bc_1 mapping to this locus. Violet line is the “k-mer” frequency and the yellow box indicate homology to a transposable element. Ric, Riccione; CS, Cabernet Sauvignon; SG, Sangiovese; bc, green, bunch closure developmental stages.

Notably, we observed a small percentage of regulated clusters (from 0.5 to 5%) exhibiting at least a 10-fold higher abundance of small RNA in Cabernet Sauvignon or Sangiovese when compared to each other (Figures 5A–C). An examination of those clusters showed that a substantial difference (50-fold or more) could exist between the cultivars, depending on the vineyard and the developmental stage. For example, in Riccione, a cluster matching a locus encoding a BURP domain-containing protein showed a fold change of 390 when comparing green berries of Sangiovese with Cabernet Sauvignon. The small RNAs mapping in this region were mainly 21-nt and produced from both strands (Figure 6). Similarly, the majority of the highly differentially expressed clusters (50-fold or more) showed a similar profile: strong bias toward 21-nt sRNAs and a low strand bias. These findings suggest that these small RNAs might be the product of RDR (RNA-dependent RNA) polymerase activity rather than degradation products of mRNAs.

### miRNAs identification and target prediction

We applied a pipeline adapted from Jeong et al. (2011) and Zhai et al. (2011) to identify annotated vvi-miRNAs, their variants, novel species-specific candidates and, when possible, the complementary 3p or 5p sequences. Starting from 25,437,525 distinct sequences from all the 48 libraries, the first filter of the pipeline removed sequences matching t/r/sn/snoRNAs as well as those that did not meet the threshold of 30 TP4M in at least one library or, conversely, that mapped in more than 20 loci of the grapevine genome (considered overly repetitive to be a miRNA). Only sequences 18–26-nt in length were retained. Overall, 27,332 sequences, including 56 known vvi-miRNAs, passed through this first filter and were subsequently analyzed by a modified version of miREAP (https://sourceforge.net/projects/mireap) as described by Jeong et al. (2011). miREAP identified 1819 miRNA precursors producing 1108 unique miRNA candidates, including 47 known vvi-miRNA. Next, the sequences were submitted to the third filter to evaluate the single-strand and abundance bias retrieving only one or two most abundant miRNA sequence for each precursor previously identified. A total of 150 unique miRNA corresponding to 209 precursors were identified as candidate miRNAs. Among these 209 candidate precursors, 61 belonged to 31 known vvi-miRNA that passed all the filters and 148 were identified as putatively novel miRNA candidates. To certify that they were novel candidates rather than variants of known vvi-miRNAs we compared their sequences and coordinates with the miRNAs registered in miRBase (version 20, Kozomara and Griffiths-Jones, 2014). In order to reduce false positives and the selection of siRNA-like miRNAs, we considered only 20, 21, and 22 nt candidates whose stem-loop structures were manually evaluated (see Supplementary Figure 3). Eventually, 26 miRNAs homologous to other plant species were identified with high confidence. Twenty-two were new members of nine known *V. vinifera* families, whereas the other four belong to three families not yet described in grapevine (Table 2). For 16 homologs we were able to retrieve also the complementary sequence. Finally, excluding these 26 miRNAs and other si-RNA like miRNAs, we identified 7 completely novel *bona fide* miRNAs.

**Table 2:** **List of novel *Vitis vinifera* miRNAs identified in Cabernet Sauvignon and Sangiovese derived small RNA libraries**.

**miRNA**	**Chr^a^**	**Start^a^**	**End^a^**	**Strand^a^**	**5p Sequence**	**nt^b^**	**Abundance 5p^c^**	**3p Sequence**	**nt^b^**	**Abundance 3p^c^**
vvi-miRC169z	14	25,082,720	25,082,864	−	TAGCCAAGGATGACTTGCCT	20	3136			
vvi−miRC171k	12	5,542,396	5,542,529	−				TTGAGCCGCGTCAATATCTCC	21	1068
vvi-miRC171l	17	893,536	893,632	+				TGATTGAGCCGTGCCAATATC	21	811
vvi-miRC171j	18	1,502,294	1,502,443	+	TGTTGGCTCGGTTCACTCAGA	21	241	TGATTGAGCCGTGCCAATATC	21	811
vvi-miRC171n	20	19,112,187	19,112,299	+	TATTGGCCCGGTTCACTCAGA	21	548	TGATTGAGCCGTGCCAATATC	21	811
vvi-miRC172e	13	6,181,370	6,181,485	−				TGAGAATCTTGATGATGCTGC	21	6451
vvi-miRC172g	6	17,652,412	17,652,523	−				TGAGAATCTTGATGATGCTGC	21	6451
vvi−miRC3624a	8	2,169,718	2,169,937	+				TCAGGGCAGCAGCATACTACT	21	20,435
vvi-miRC390a	8	9,571,412	9,571,529	+	AAGCTCAGGAGGGATAGCGCC	21	5046			
vvi−miRC396e	1	1,997,798	1,998,004	+	TTCCACGGCTTTCTTGAACTT	21	66,306	TTCAAGAAAGCCGTGGGAAAA	21	2074
vvi-miRC403g	10	15,150,892	15,150,979	−	TTAGATTCACGCACAAACTC	20	3262			
vvi−miRC477c	19	12,889,963	12,890,080	+	TCCCTCAAAGGCTTCCAATTT	21	453	GTTGGAAGCCGGTGGGGGACC	21	83,879
vvi-miRC477i	20	16,672,963	16,673,080	−	TCCCTCAAAGGCTTCCAATTT	21	453	GTTGGAAGCCGGTGGGGGACC	21	83,879
vvi-miRC477j	6	19,950,730	19,950,847	−	TCCCTCAAAGGCTTCCAATTT	21	453	GCTGGAAGCCGATGGGGGACC	21	62,642
vvi-miRC477k	1	22,740,264	22,740,350	+	TCCCTCAAAGGCTTCCAATTT	21	453			
vvi-miRC477l	19	13,141,671	13,141,788	+	TCCCTCAAAGGCTTCCAATTT	21	453	GTTGGAAGCCGGTGGGGGACC	21	83,879
vvi-miRC477m	19	13,510,093	13,510,210	+	TCCCTCAAAGGCTTCCAATTT	21	453	GTTGGAAGCCGGTGGGGGACC	21	83,879
vvi-miRC477n	19	18,678,338	18,678,455	+	TCCCTCAAAGGCTTCCAATTT	21	453	GTTGGAAGTCGGTGGGGGAAC	21	17,594
vvi-miRC477o	19	18,872,706	18,872,824	−	TCCCTCAAAGGCTTCCAATTT	21	453	GTTGGAAGTCGGTGGGGGACC	21	30,683
vvi-miRC477p	19	18,881,351	18,881,468	−	TCCCTCAAAGGCTTCCAATTT	21	453	GTTGGAAGCCGGTGGGGGACC	21	83,879
vvi-miRC482a	14	19,755,466	19,755,588	−	GGAATGGGCTGATTGGGATA	20	189,848	TTCCCAATGCCGCCCATTCCAA	22	17,229
vvi-miRC482b	1	3,865,560	3,865,686	+	CATGGGCGGTTTGGTAAGAGG	21	143,513	TCTTACCAACACCTCCCATTCC	22	37,745
vvi−miRC530	6	17,896,112	17,896,288	+	TCTGCATTTGCACCTGCACCT	21	19,330	AGGTGCAGGTGAAGGTGCAGA	21	207
vvi-miRC530b	8	18,999,725	18,999,889	−	TCTGCATTTGCACCTGCACCT	21	19,330			
vvi-miRC827	5	24,742,113	24,742,240	−	TTTTGTTGCTGGTCATCTAGTC	22	6721	TTAGATGATCATCAACAAACA	21	12,436
vvi−miRC7122	5	6,820,719	6,820,833	−	TTACACAGAGAGATGACGGTGG	22	535	ACCGTCTTTCTCTGTATAAGC	21	1227
grape-m0642	17	3,771,923	3,772,046	+	GAGGTGATAGATCAGCAAGAG	21	31			
grape−m1188	29	1,474,782	1,474,863	+				CCCGAGAGGACTTAGTGGATC	21	413
grape-m1191	29	469,980	470,068	-				TTGCTGAACAAGAGAGAACCT	21	1859
grape−m1355	3	13,241,508	13,241,588	−	GCCGCGTTGGAGCAGGAGCTT	21	740			
grape-m1517	5	11,023,593	11,023,756	−				AACTACTGAATCATTGACCAG	21	90
grape−m1577	6	6,902,214	6,902,359	−				GGCACGACAGTCTTGGACGCC	21	31
grape-m1738	8	21,905,716	21,905,814	−	TCGTAGTGGCTGCGACAGCTCC	22	2406			

*A temporary name has been given to each miRNA sequence either using a sequential numbering associated with the abbreviation mirC (miRNA Candidate) for those similar to other known plant miRNAs or using “grape-m” with a random numbering for those completely new candidates*.

a *Refers to the genomic localization on the GRAPE_IGGP12Xv1 genome sequence*.

b *Nucleotide, sequence length of the microRNA*.

c *Sum of TP4M values from 48 libraries*.

Apart from the 61 known vvi-miRNAs identified by the pipeline, we searched the dataset for others known vvi-miRNAs eliminated throughout the pipeline, looking for isomiRs that were actually more abundant than the annotated sequences. Their complementary 3p or 5p sequence was also retrieved when possible. Hence 89 known vvi-miRNAs were identified in at least one of our libraries (Table 3). Among the known vvi-miRNAs identified, 24 had an isomiR more abundant than the annotated sequence and 4 have the complementary sequence as the most abundant sequence mapping to their precursor. We found 16 vvi-miRNA isomiRs that were either longer or shorter than the annotated sequence, 7 vvi-miRNAs that mapped in the precursor in a position shifted with respect to the annotated ones and one miRNA that contains a nucleotide gap when compared to the annotated sequence (Table 3). An extreme case of shifted position was found in vvi-miRNA169c, where the annotated sequence had only 5 TP4M when summing its individual abundance in the 48 libraries. Another sequence, shifted 16 bp as compared to its annotated position on the precursor had an abundance sum of 1921 TP4M, and was retained together with the annotated sequence, and named vvi-miRNA169c.1. For 36 of the 48 *V. vinifera* miRNA families deposited in miRBase we found at least one member.

**Table 3:** **List of reliable *Vitis vinifera* miRNAs from known miRNA precursors identified in Cabernet Sauvignon and Sangiovese derived small RNA libraries**.

**Family**	**miRNA**	**Chr^a^**	**Start^a^**	**End^a^**	**Strand^a^**	**5p Sequence**	**nt^b^**	**Abundance 5p^c^**	**3p Sequence**	**nt^b^**	**Abundance 3p^c^**
vvi−miR156	vvi-miR156b	4	5,357,061	5,357,310	−				TGACAGAAGAGAGTGAGCAC	20	10,561
	vvi-miR156c	4	848,207	848,307	−	TGACAGAAGAGAGTGAGCAC	20	10,561			
	vvi-miR156d	11	7,623,202	7,623,334	−	TGACAGAAGAGAGTGAGCAC	20	10,561	TGCTCACCTCTCTTTCTGTCAGC	23	1089
	vvi-miR156e	11	1,504,195	1,504,301	−	TGACAGAGGAGAGTGAGCAC	20	234			
	vvi-miR156f	14	26,463,671	26,463,773	+	TTGACAGAAGATAGAGAGCAC	21	26,445			
	vvi-miR156g	17	3,046,310	3,046,441	−	TTGACAGAAGATAGAGAGCAC	21	26,445	GCTCTCTAGACTTCTGTCATC	21	901
	vvi-miR156i	14	19,727,139	19,727,358	−				TTGACAGAAGATAGAGAGCAC	21	26,445
vvi-miR159	vvi-miR159c	17	2,609,190	2,609,409	−	GAGCTCCTTGAAGTCCAATAG	21	1644	TTTGGATTGAAGGGAGCTCTA	21	198,072
vvi−miR162	vvi-miR162	17	4,716,504	4,716,636	+	GGATGCAGCGGTTCATCGATC	21	365	TCGATAAACCTCTGCATCCAG	21	130,641
vvi-miR164	vvi-miR164a	7	3,287,472	3,287,590	−	TGGAGAAGCAGGGCACGTGCA	21	198			
	vvi-miR164c	8	1,008,0445	10,080,636	+	TGGAGAAGCAGGGCACGTGCA	21	198	CATGTGCCCCTCTTCCCCATC	21	976
	vvi-miR164d	14	1,414,567	1,414,682	−	TGGAGAAGCAGGGCACGTGCA	21	198			
vvi−miR166	vvi-miR166a	8	3,302,784	3,302,939	−	AATGAGGTTTGATCCAAGATC	21	1886	TCTCGGACCAGGCTTCATTCC	21	1,172,737
	vvi-miR166b	12	17,937,384	17,937,510	+	TCGGACCAGGCTTCATTCCTC	21	10,941	GGAATGTTGGCTGGCTCGAGG	21	120,762
	vvi-miR166c	15	16,978,558	16,978,745	−	GGAATGTTGTCTGGCTCGAGG	21	54,367	TCGGACCAGGCTTCATTCCCC	21	17,706,997
	vvi-miR166d	16	21,405,202	21,405,388	−	GATTGTTGTCTGGCTCGAGGC	21	2661	TCGGACCAGGCTTCATTCCCC	21	17,706,997
	vvi-miR166e	2	2,255,708	2,255,901	+	GGAATGTTGTCTGGCTCGAGG	21	54,367	TCGGACCAGGCTTCATTCCCC	21	17,706,997
	vvi-miR166f	7	19,450,000	19,450,127	+	GGAATGTTGGCTGGCTCGAGG	21	10,941	TCGGACCAGGCTTCATTCCCC	21	17,706,997
	vvi-miR166g	7	453,844	453,971	−	GGAATGTTGTCTGGTTCGAGA	21	262	TCGGACCAGGCTTCATTCCCC	21	17,706,997
	vvi-miR166h	5	6,741,189	6,741,288	−				TCGGACCAGGCTTCATTCCCC	21	17,706,997
vvi-miR167	vvi-miR167b	14	7,137,373	7,137,501	+	TGAAGCTGCCAGCATGATCT-	21	1143	AGATCATGTGGCAGTTTCACC	21	536
	vvi-miR167d	20	7,490,493	7,490,606	+	TGAAGCTGCCAGCATGATCT-	21	1143			
	vvi-miR167e	5	5,845,370	5,845,489	+	TGAAGCTGCCAGCATGATCT-	21	1143	AGATCATGTGGCAGTTTCACC	21	536
vvi−miR168	vvi-miR168	2	17,944,786	17,944,947	−	TCGCTTGGTGCAGGTCGGGAA	21	43,158	CCCGCCTTGCATCAACTGAAT	21	3558
	vvi-miR169c	4	2,265,913	2,266,028	−	CAGCCAAGGATGACTTGCCGG	21	5			
vvi-miR169	vvi-miR169c.1	4	2,265,913	2,266,028	−	TGTAGGGAGTAGAATGCAGC	20	1921			
	vvi-miR169e	14	25,082,574	25,082,756	−	TAGCCAAGGATGACTTGCCT–	22	3136			
	vvi-miR169g	8	21,104,448	21,104,568	+	CAGCCAAGGATGACTTGCCGA	21	13	^*^CCGGCAAGTTGTCTTTGGCTAC	23	151
	vvi-miR169r	11	16,415,128	16,415,239	+	TGAGTCAAGGATGACTTGCCG	21	11	^*^GGCAAGTTGACTTGACTCAGT	22	1181
	vvi-miR169t	11	16,399,564	16,399,676	+	CGAGTCAAGGATGACTTGCCGA	22	14	^*^GGCAAGTTGACTTGACTCAGT	22	1181
vvi−miR171	vvi-miR171b	12	5,542,396	5,542,529	−				—TTGAGCCGCGTCAATATCTCC	24	1068
	vvi-miR171i	17	893,536	893,632	+				TGATTGAGCCGTGCCAATATC	21	811
vvi-miR172	vvi-miR172d	8	12,667,173	12,667,279	+				TGAGAATCTTGATGATGCTGC–	23	6451
vvi−miR2111	vvi-miR2111	8	15,368,664	15,368,748	−	TAATCTGCATCCTGAGGTCTA	21	1476	GTCCTCTGGTTGCAGATTACT	21	95
vvi-miR2950	vvi-miR2950	7	14,340,406	14,340,517	+	TTCCATCTCTTGCACACTGGA	21	3176	TGGTGTGCACGGGATGGAATA	21	581
vvi−miR319	vvi-miR319b	1	4,189,556	4,189,753	+				TTGGACTGAAGGGAGCTCCC-	21	9157
	vvi-miR319c	2	855,548	855,756	−	AGAGCTTTCTTCAGTCCACTC	21	131	TTGGACTGAAGGGAGCTCCC-	21	9157
	vvi-miR319e	11	4,317,223	4,317,329	+				TTTGGACTGAAGGGAGCTCCT	21	40,107
	vvi-miR319f	6	9,137,252	9,137,445	+				TTGGACTGAAGGGAGCTCCC-	21	9157
	vvi-miR319g	17	3,675,979	3,676,209	−				TTGGACTGAAGGGAGCTCCC-	21	9157
vvi-miR3623	vvi-miR3623	18	24,650,004	24,650,151	+	TCACAAGTTCATCCAAGCACCA	22	108,278	GTGCTTGGACGAATTTGCTAAG	22	20,404
vvi−miR3627	vvi-miR3627	14	28,302,559	28,302,681	−	TTGTCGCAGGAGAGACGGCACT	22	2444	TCGCCGCTCTCCTGTGACAAG	21	12,342
vvi-miR3629	vvi-miR3629a	13	18,253,987	18,254,170	+	CGCATTTTCTCAGCAGCCAAG	21	1	TGGCTGCTGAGAAAATGTAGG-	22	371
	vvi-miR3629c	17	822,209	822,338	+	TGGCTGCTGAGAAAATGTAGG-	22	371			
vvi−miR3632	vvi-miR3632	14	23,394,889	23,395,015	+	GGATTGGGGGCCGATGGAAAGG	22	887	TTTCCCAGACCCCCAATACCAA	22	1578
vvi-miR3633	vvi-miR3633a	17	5,521,913	5,522,060	+	GGAATGGATGGTTAGGAGAG	20	45,786	TTCCTATACCACCCATTCCCTA	22	1469
	vvi-miR3633b	17	5,521,557	5,521,691	+	GGAATGGGTGGCTGGGATCT	20	10,270	GTTCCCATGCCATCCATTCCTA	22	5610
vvi−miR3634	vvi-miR3634	17	5,681,202	5,681,314	−	GGCATATGTGTGACGGAAAGA	21	1563	TTTCCGACTCGCACTCATGCCGT	23	753,254
vvi-miR3635	vvi-miR3635	18	27,357,619	27,357,780	+	GGCATGTGTGGGGCATAATAG	21	289	ATTATGTCCCACACATGCCTC	21	399
vvi−miR3636	vvi-miR3636	16	5,414,817	5,414,967	−	TCGGTTTGCTTCTTTGATAGATTC	24	352	— TGTCGGAGAAGCAAGTCGGAGAGT	24	4495
vvi-miR3640	vvi-miR3640	16	11,986,842	11,987,014	+	ACCTGATTGGTGATGCTTTTTTGG	24	1321	—- AAAGGCATCATCAATCAGGTAATG	24	1203
vvi−miR390	vvi-miR390	6	8,159,519	8,159,657	+	AAGCTCAGGAGGGATAGCGCC	21	5046			
vvi-miR393	vvi-miR393a	16	17,247,172	17,247,327	−	TCCAAAGGGATCGCATTGATC	21	5152	ATCATGCTATCCCTTAGGAAC	21	775
	vvi-miR393b	13	4,265,132	4,265,213	+	TCCAAAGGGATCGCATTGATC	21	5152			
vvi−miR394	vvi-miR394a	12	17,122,005	17,122,092	−	TTGGCATTCTGTCCACCTCC–	22	412			
	vvi-miR394b	18	1,413,038	141,3130	−	TTGGCATTCTGTCCACCTCC	20	412			
vvi-miR395	vvi-miR395a	1	6,527,921	6,528,019	+				CTGAAGTGTTTGGGGGAACTC	21	6899
	vvi-miR395c	1	6,499,899	6,500,020	−	GTTCCCTTGACCACTTCACTG	21	5850	CTGAAGTGTTTGGGGGAACTC	21	6899
	vvi-miR395d	1	6,512,760	6,512,848	+				CTGAAGTGTTTGGGGGAACTC	21	6899
	vvi-miR395e	1	6,505,233	6,505,354	+	GTTCCCTTGACCACTTCACTG	21	5850	CTGAAGTGTTTGGGGGAACTC	21	6899
	vvi-miR395f	1	6,489,527	6,489,642	+	GTTCCCCTGACCACTTCACTG	21	4729	CTGAAGTGTTTGGGGGAACTC	21	6899
	vvi-miR395h	1	6,566,637	6,566,758	+	GTTCCCTTGACCACTTCACTG	21	5850	CTGAAGTGTTTGGGGGAACTC	21	6899
	vvi-miR395i	1	6,562,627	6,562,742	+	GTTCCCCTGACCACTTCACTG	21	4729	CTGAAGTGTTTGGGGGAACTC	21	6899
	vvi-miR395j	1	6,553,011	6,553,126	+	GTTCCCCTGACCACTTCACTG	21	4729	CTGAAGTGTTTGGGGGAACTC	21	6899
	vvi-miR395k	1	6,536,764	6,536,885	+	GTTCCCTTGACCACTTCACTG	21	5850	CTGAAGTGTTTGGGGGAACTC	21	6899
	vvi-miR395l	1	6,559,083	6,559,199	+	GTTCCCCTGACCACTTCACTG	21	4729	CTGAAGTGTTTGGGGGAACTC	21	6899
vvi−miR396	vvi-miR396a	9	7,372,499	7,372,649	−	TTCCACAGCTTTCTTGAACTA	21	15,069	AAGAAAGCTGTGGGAGGACATGGC	24	2147
	vvi-miR396b	11	5,246,778	5,246,913	+	TTCCACAGCTTTCTTGAACTT	21	85,683	GTTCAAGAAAGCTGTGGGAAA	21	6917
	vvi-miR396c	4	5,119,592	5,119,698	−	TTCCACAGCTTTCTTGAACTG	21	3900			
	vvi-miR396d	11	5,253,095	5,253,244	−	TTCCACAGCTTTCTTGAACT-	21	7387	GTTCAATAAAGCTGTGGGAAG	21	1899
vvi-miR397	vvi-miR397a	20	11,971,890	11,972,015	−	TCATTGAGTGCAGCGTTGATG	21	432			
vvi−miR398	vvi-miR398b	6	16,503,544	16,503,633	−				TGTGTTCTCAGGTCGCCCCTG	21	16,121
	vvi-miR398c	6	15,575,579	15,575,668	+				TGTGTTCTCAGGTCGCCCCTG	21	16,121
vvi-miR399	vvi-miR399a	10	2,989,435	2,989,561	+	GTGTGATTCTCCTTTGGCAGA	21	503	TGCCAAAGGAGAATTGCCCTG	21	1289
	vvi-miR399b	16	15,618,708	15,618,845	−				TGCCAAAGGAGAGTTGCCCTG	21	2996
	vvi-miR399c	15	15,232,197	15,232,281	+				TGCCAAAGGAGAGTTGCCCTG	21	2996
	vvi-miR399e	10	2,992,220	2,992,341	−				TGCCAAAGGAGATTTGCCCGG	21	184
	vvi-miR399h	10	2,983,543	2,983,634	+				TGCCAAAGGAGAATTGCCCTG	21	1289
	vvi-miR399i	2	4,101,786	4,101,937	+	GGGCTTCTCTCCTTCTGGCAGG	22	148	CGCCAAAGGAGAGTTGCCCTG	21	113,055
vvi−miR403	vvi-miR403a	5	65,247	65,361	+				TTAGATTCACGCACAAACTCG	21	82,567
	vvi-miR403b	5	600,176	600,266	+				TTAGATTCACGCACAAACTCG	21	82,567
	vvi-miR403d	5	166,467	166,582	+	AGTTTGTGCGCGAATCCAACC	21	201	TTAGATTCACGCACAAACTCG	21	82,567
	vvi-miR403e	5	168,096	168,213	+				TTAGATTCACGCACAAACTCG	21	82,567
	vvi-miR403f	7	4,179,658	4,179,795	−	AGTTTGTGCGTGACTCTAAAA	21	523	TTAGATTCACGCACAAACTCG	21	82,567
vvi-miR408	vvi-miR408	7	5,011,920	5,012,056	+	CGGGGACGAGGTAGTGCATGG	21	3223	ATGCACTGCCTCTTCCCTGGC	21	6566
vvi−miR477	vvi-miR477b	2	1,237,529	1,237,808	+	ACTCTCCCTCAAGGGCTTCT-G	22	513	CGAAGTCTTTGGGGAGAGTGG	21	3289
vvi-miR479	vvi-miR479	16	21,573,744	21,573,866	+	TGTGGTATTGGTTCGGCTCATC	22	4449	CGAGCCGAACCAATATCACTC	21	46,073
vvi−miR482	vvi-miR482	17	5,523,009	5,523,155	+	^*^AATTGGAGAGTAGGAAAGCTT	22	139,166	TCTTTCCTACTCCTCCCATTCC	22	42,801
vvi-miR535	vvi-miR535a	25	1,392,255	1,392,353	−	TGACAACGAGAGAGAGCACGC	21	18,893			
	vvi-miR535c	25	1,346,353	1,346,483	−	TGACAACGAGAGAGAGCACGC	21	18,893	GTGCTCTCTGTCGCTGTCATA	21	2191

a *Refers to the genomic localization on the GRAPE_IGGP12Xv1 genome sequence*.

b *Nucleotide, sequence length of the microRNA*.

c *Sum of TP4M values from 48 libraries*.

*−Missing nucleotide*.

*Nucleotides that differ from the annotated sequence are shown in red*.

* *Indicates that the most abundant sequence in our dataset does not correspond to the mature annotated miRNA, but to the star sequence*.

An *in silico* prediction of miRNA targets was performed for the 191 mature miRNAs here identified. Using the miRferno tool (Kakrana et al., 2014), and considering only targets predicted with high stringency, 1192 targets were predicted for 143 miRNAs, including six completely novel vvi-miRNA candidates (Supplementary Table 3).

Two novel candidates (grape-m1191 and grape-m1355) seem to be involved in the regulation of important secondary metabolites biosynthesis. Among the six targets predicted for grape-m1191, the *TT12* gene (*transparent testa 12* - VIT_212s0028g01160) is known to be involved in the vacuolar accumulation of proanthocyanidins in grapevine (Marinova et al., 2007). For grape-m1355, 12 targets were predicted and all of them are involved in secondary metabolism pathways. Nine targets code a bifunctional dihydroflavonol 4-reductase (DFR) that is responsible for the production of anthocyanins (Davies et al., 2003), catalyzing the first step in the conversion of dihydroflavonols to anthocyanins (Boss and Davies, 2001). Another targeted gene codes a phenylacetaldehyde reductase (VIT_213s0064g00340) which, in tomato, was demonstrated to catalyze the last step in the synthesis of the aroma volatile 2-phenylethanol, important for the aroma and flavor (Tieman et al., 2007). Still this same miRNA candidate was predicted to target with high confidence (score = 0) a cinnamoyl reductase-like protein (VIT_203s0110g00350) that is part of polyphenol biosynthetic pathway (Martínez-Esteso et al., 2013). The grape-m1355 candidate maps on chromosome 3, exactly on the first exon of its target (VIT_203s0110g00350.1), in a region where another two isoforms of the same gene are located (Supplementary Figure 4). The last target of this miRNA candidate, codes a cinnamyl alcohol dehydrogenase known to be involved in the lignin biosynthesis (Trabucco et al., 2013).

Other novel vvi-miRNA candidates seem to be involved in cell proliferation (grape-m0642 targets VIT_200s0291g00090, a cyclin-related protein with hydrolase activity) and in chloroplasts-related functions (grape-m1517 targets VIT_203s0063g02020, a tic62 protein). Furthermore, for the new vvi-miRC482b candidate, besides the already known involvement of this miRNA family with disease resistance (Li et al., 2010) also predicted here, one predicted target encodes an anthocyanin 5-aromatic acyltransferase-like protein known to be involved in the biosynthesis of anthocyanin in different species (Provenzano, 2011).

As for the conserved known vvi-miRNAs, most of the well-established examples of miR-targets, such as miR156-*SPB*, miR166-*HD-ZIP*, miR171-*GRAS*, miR172-*AP2*, confirmed in several plant species and already predicted in grapevine, were also predicted here.

### miRNA accumulation among vineyards and genotypes

We studied miRNA profile of accumulation in the different samples. Using their normalized abundance (TP4M), i.e., their relative cloning frequency, we set an empirical cut off value equal to at least 10 TP4M in both biological replicates to consider a miRNA as expressed in a given library. Also, a miRNA was considered specific when it was expressed in one or more libraries of a unique cultivar, unique environment or unique developmental stage.

**Figure 7:**
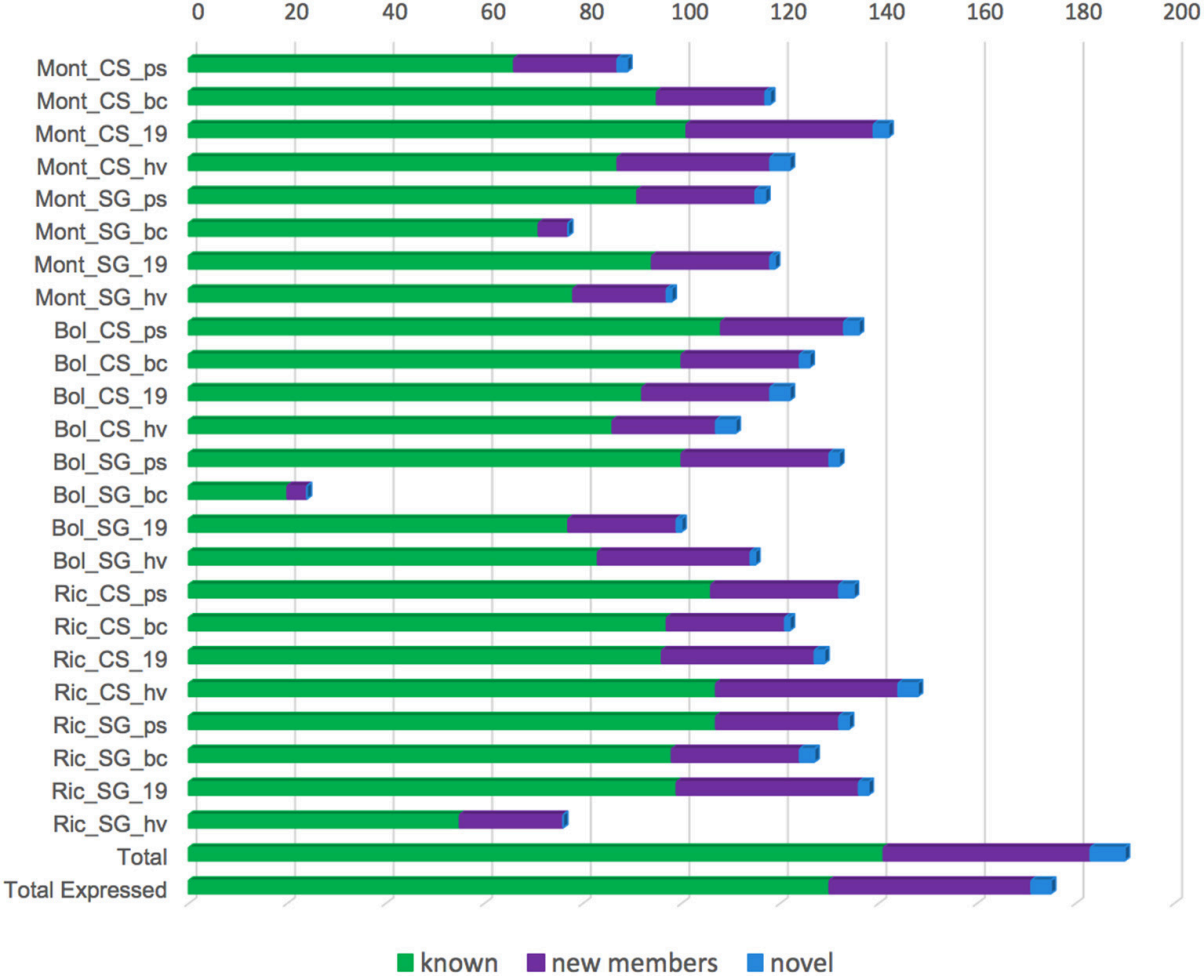
**Number of miRNA expressed in each small RNA library constructed using berries of 2 grapevine cultivars [Cabernet Sauvignon (CS) and Sangiovese (SG)], collected in 4 different developmental stages [pea size (ps), bunch closure (bc), 19 °Brix (19), harvest (hv)] and 3 vineyards [Montalcino (Mont), Bolgheri (Bol), Riccione (Ric)]**. “*Total”* refers to the total number of miRNAs, comprising known, novel and their complementary molecules identified in our data. “*Total expressed”* refers to the number of miRNAs expressed in at least one library. Library codes are found in Table 1.

According to our established cut off, 175 miRNAs were classified as expressed in at least one of our libraries (Figure 7). The libraries constructed from Sangiovese berries at bunch closure collected in Bolgheri showed only 24 expressed miRNAs (Figure 7). For all the other libraries, expressed miRNAs ranged from 76 (Ric_SG_hv) to 148 (Ric_CS_hv) (Figure 7).

**Figure 8:**
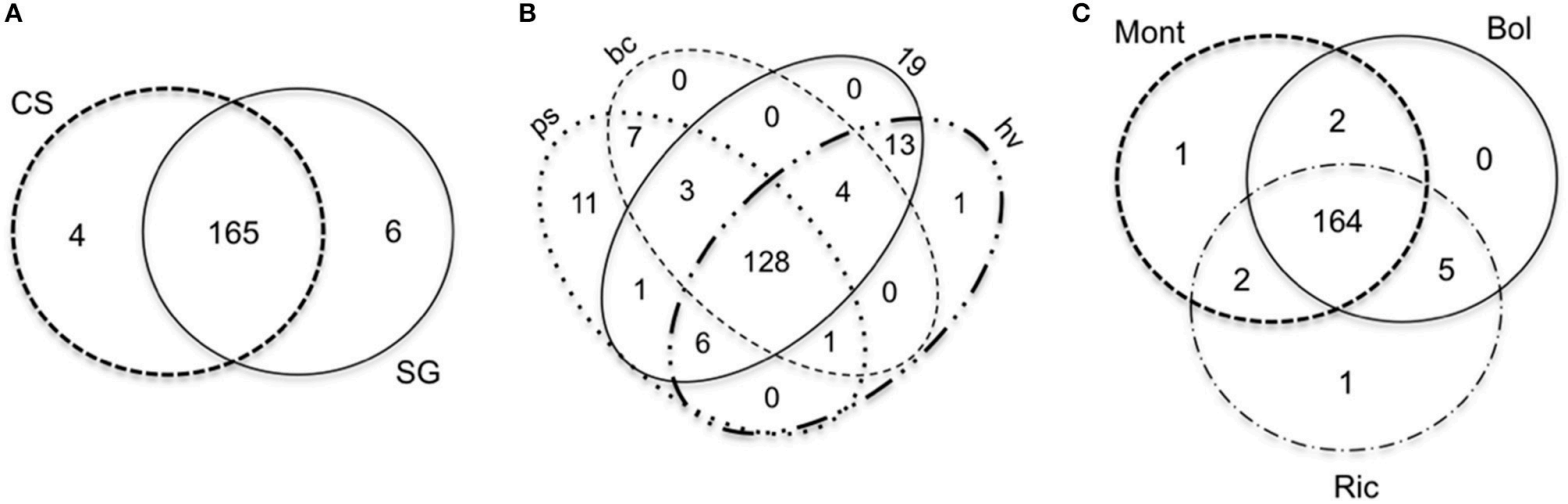
**Venn diagrams comparing the miRNAs expressed in small RNA libraries of *Vitis vinifera* constructed using berries**. The venn diagrams are divided by **(A)**, cultivar; **(B)**, developmental stage; **(C)**, environment. Bol, Bolgheri; Mont, Montalcino; Ric, Riccione; CS, Cabernet Sauvignon; SG, Sangiovese.

We found very few miRNAs specific to a given condition. The number of specific miRNAs for each cultivar, developmental stage and environment is reported in Figures 8A–C, respectively.

**Figure 9:**
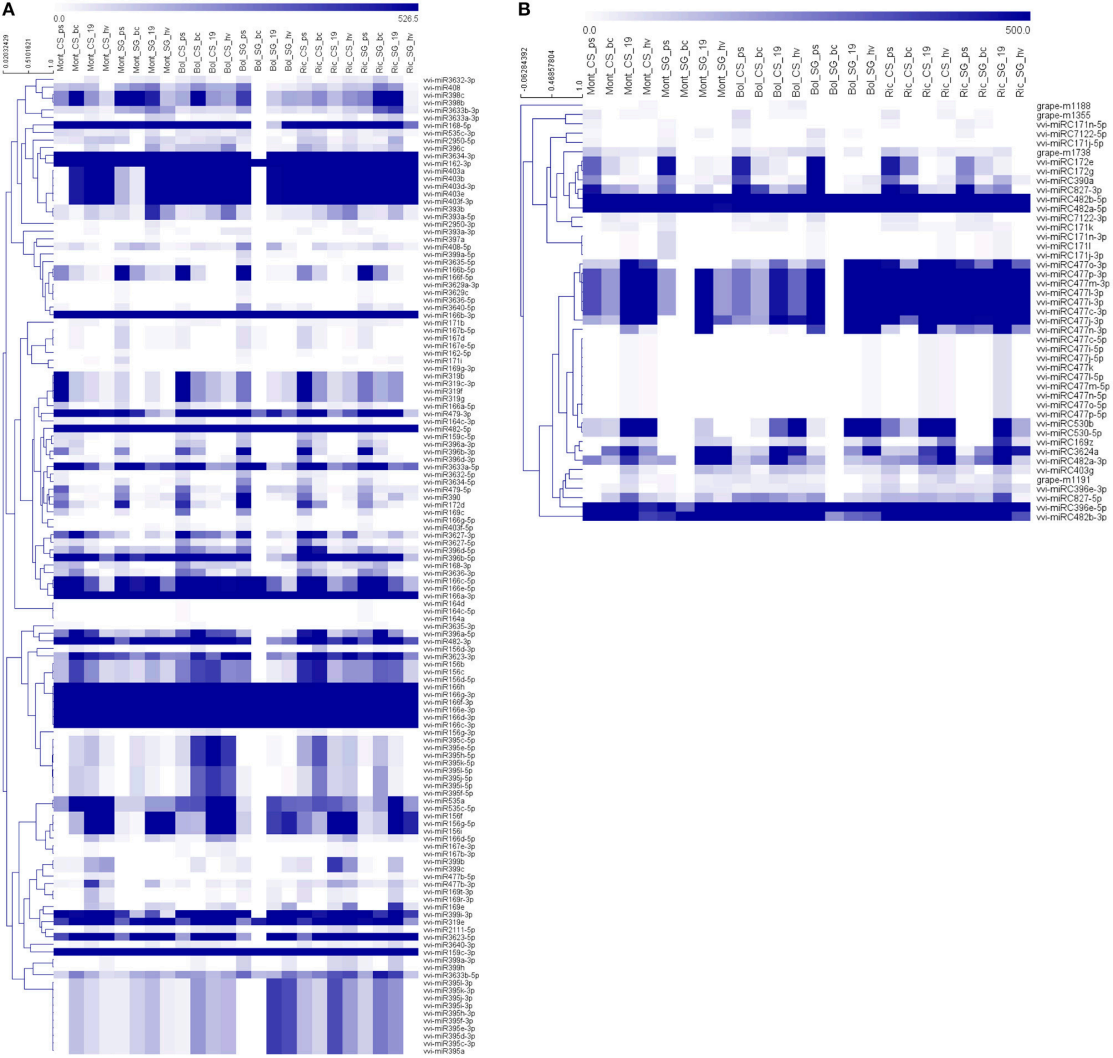
**Heatmap of hierarchically clustered (HCL) miRNAs, expressed in grapevine berries collected from 2 varieties in 4 developmental stages and growing in 3 vineyards**. The HCL tree was generated with the average linkage clustering method. Blue and white represent high and low expression, respectively. Known and novel miRNAs are represented in two separated panels in order to reduce figures complexity **(A)** HCL of known miRNAs; **(B)** HCL of novel miRNA candidates.

Thirty-nine vvi-miRNAs were highly expressed in almost all libraries [21 ubiquitous plus 18 expressed in all libraries except in Bol_SG_bc (Figure 9)], whereas other miRNAs had different accumulation patterns.

**Figure 10:**
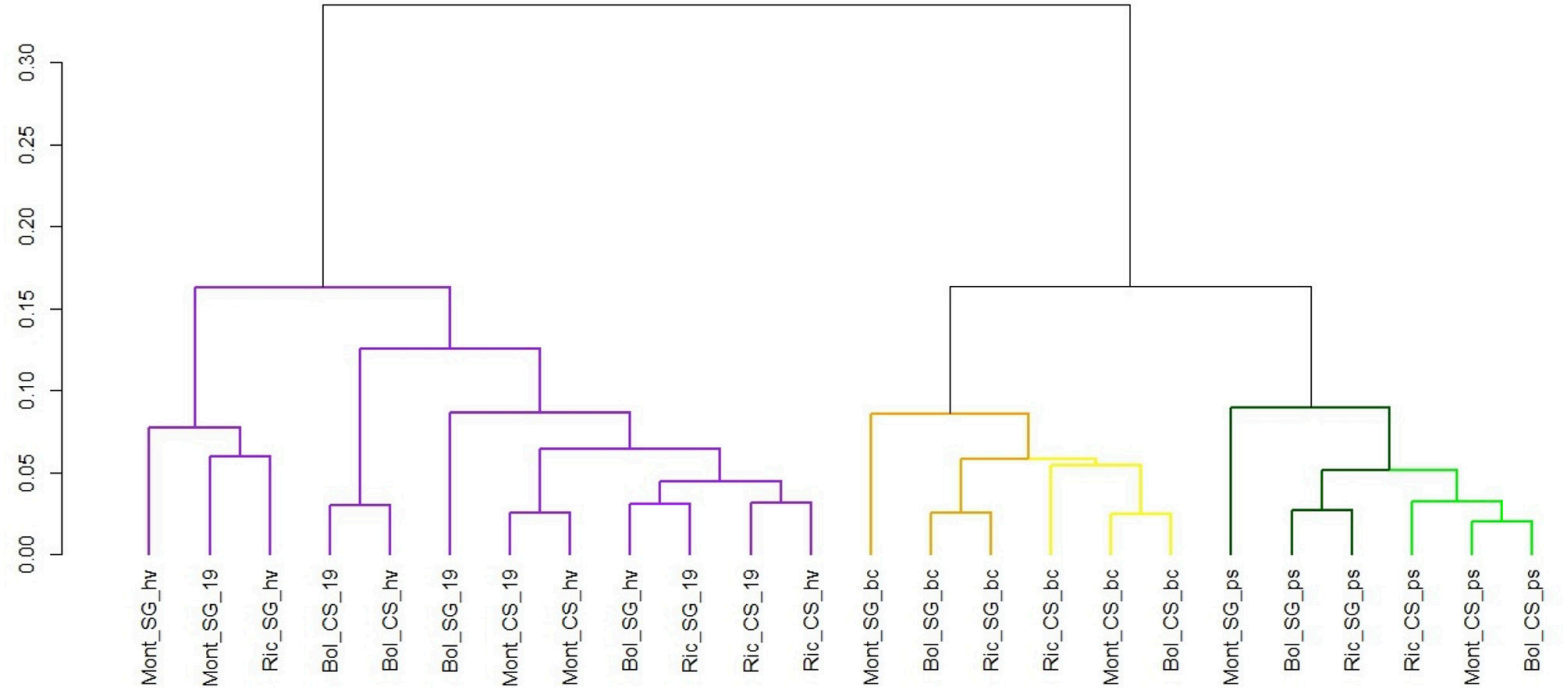
**Cluster dendrogram showing global relationships of miRNAs expressed in different grapevine cultivars, collected in different vineyards and developmental stages**. The Pearson correlation coefficients, calculated based on the abundance of expressed miRNAs in each library, were converted into distance coefficients to define the height of the dendrogram. Different colors distinguish different clusters of samples. Library codes can be found in Table 1.

The normalized expression values of miRNAs were subjected to hierarchical clustering (HCL) and represented in a heat map (Figure 9). To examine the relatedness among cultivars, environments and developmental stages, we generated a correlation dendrogram (Figure 10). The dendrogram shows, as already suggested by the heatmaps, that a fundamental dichotomy emerges between ripened and green berries. The most evident pattern of expression is observed when comparing different developmental stages, and confirm previous observation of miRNA modulation during fruit ripening (Manning et al., 2006; Giovannoni, 2007; Carra et al., 2009; Sun et al., 2012; Cao et al., 2016). For example, some members of the vvi-miRNA156 family (f/ i and the g-5p) were highly expressed in all ripened berries, but weakly or not expressed in green berries. Differently, vvi-miR396a-3p and vvi-miR396b-3p showed the opposite profile. Similarly, vvi-miR172d, vvi-miR166b-5p, vvi-miR166f-5p, and vvi-miR396d-5p were highly expressed in green berries but weakly expressed in ripened berries and the members of the vvi-miR319 family (b/f/g and c-3p) showed a gradient of decreasing abundance from pea size to harvest.

To gain statistical evidence of miRNA differential expression driven by the environment and/or genotype, we made pairwise comparisons, keeping constant the developmental stage, and evaluating the miRNA modulation among vineyards (Montalcino vs Bolgheri vs Riccione) or between cultivars (Cabernet Sauvignon vs Sangiovese). The analyses (with an FDR ≤ 0.05) reveal that some miRNAs are differentially expressed between the two genotypes grown in the same environment, but also that a number of miRNAs are modulated by the environment. In particular the number of differentially expressed miRNAs is higher in ripened berries (19 °Brix and at harvest), while no miRNAs are differentially expressed at bunch closure stage (Supplementary Table 4). In details, 14 reads are differentially expressed at pea size stage, in at least one comparison, corresponding to 6 distinct miRNA families; 27 reads are modulated at 19 °Brix stage, corresponding to 12 miRNA families and 35 reads are differentially expressed in berries at harvest, corresponding to 12 miRNA families. It is worth noting that 4 of the 6 families modulated in the berries at pea size, are still present among the miRNAs differentially expressed in the berries sampled at 19 °Brix and at harvest (miR166, miR3627, miR477, miR3636, and miR3640), even though not always in the same comparisons.

Some of the modulated miRNAs, both novel (grape-m1355, grape-m1191) and known (miR395, miR399, and miR396) are intriguingly connected to berry development and secondary metabolism, even though most of the modulated families are still uncharacterized, or with targets not clearly involved in berry ripening and development, and deserve further studies to fully understand their biological roles.

## Discussion

Using high throughput sequencing coupled with robust bioinformatics pipelines we analyzed small RNAs derived from the berries of Cabernet Sauvignon and Sangiovese, grown side-by-side in three vineyards, representative of different grapevine cultivation areas in Italy (Bolgheri, Montalcino, and Riccione). We obtained nearly 750 MB reads comprising a significant proportion of small (21–24 nt) RNAs. The size distribution profiles of our libraries were in general consistent with previous reports in berry grapevine, where the 21-nt class was more abundant than the 24-nt class (Pantaleo et al., 2010; Wang et al., 2012; Han et al., 2014; Kullan et al., 2015).

Our analysis revealed dynamic features of the regulatory network mediated by miRNAs and other small RNAs, at the basis of genotype-environment interactions.

### Genotype and environment effects on small RNA profiles

Plants evolved a series of pathways that generate small RNAs of different sizes with dedicated functions (Vazquez, 2006; Khraiwesh et al., 2012). Although the various small RNA classes have been intensively studied, we are still far from understanding how many small RNA pathways exist, and how they are connected (Vazquez, 2006). Additionally, new classes of small non-coding RNAs continue to be discovered and many studies demonstrate a substantial redundancy and cross-talk between known small RNA pathways (Agarwal and Chen, 2009; Ghildiyal and Zamore, 2009; Bond and Baulcombe, 2014; Harding et al., 2014). Estimating the exact percentage of the plant genome covered by small RNA-generating loci still remains a challenge.

By applying static cluster analysis, we investigated small RNA abundances across the genome, identifying 4408 small RNAs producing hotspots. We analyzed their expression in different cultivars, environments and developmental stages, highlighting that the majority of the considered small RNA producing regions was modulated in different conditions. This suggests a strong influence of small RNAs in the response to environment in grapevine berries. Only 462 small RNA-generating loci, corresponding to about 10% of the total, were expressed in all the analyzed libraries, possibly involved in essential biological pathways.

Comparing the two cultivars, we observed, with few exceptions, that Cabernet Sauvignon berries have a higher number of expressed sRNA-generating loci than Sangiovese berries (Figure 1) when collected in the same conditions (i.e., vineyard and developmental stage). Considering the fact that small RNAs are implicated in the regulation of gene expression in several processes (Chen, 2009; Trindade et al., 2011), the higher number of small RNAs expressed in Cabernet Sauvignon compared to Sangiovese berries may reflect a buffering effect of small RNAs influencing grapevine response to diverse growing environments. We believe that these characteristics may have contributed to the wide diffusion of Cabernet Sauvignon, allowing its wide cultivation in almost all wine producing countries. This is not the case for Sangiovese whose cultivation is more restricted. It is worth noting that Sangiovese is considered a very unsettled grapevine cultivar (Poni, 2000), showing a wide range of variability in response to year, clone and bunch exposure (Rustioni et al., 2013). Differently, Cabernet Sauvignon is a cultivars showing less inter-annual differences in terms, for example, of concentration of secondary metabolites (Ortega-Regules et al., 2006).

To better evaluate varietal differences in response to the environment, we calculated the CS/SG ratio for the small RNA producing hotspots in the three vineyards. An interesting example is found in green berries sampled in Riccione. A region on chromosome 4 (3,376,501–3,377,000) showed a 390-fold change in the small RNA abundance, when comparing Cabernet vs. Sangiovese (Figure 6). Most of the reads produced in this region are 21 nt long and are also phased in intervals of 21 nt from both strands, typical of a *phased locus (PHAS)*. The gene in this locus, also known as *VvRD22g*, encodes a BURP domain-containing protein, involved in an ABA-mediated abiotic stress response, which persists still after long periods of stress (Matus et al., 2014). The small RNAs profile suggests that the locus is regulated by *phased* siRNAs similarly to the mechanisms already described for PPR, NB-LRR, and MYB gene families (Howell et al., 2007; Zhai et al., 2011; Xia et al., 2013; Zhu et al., 2013). This is a clear example of GxE interactions since the BURP domain gene modulates *phased* siRNAs production in the two cultivars only when grown in Riccione.

When removing the threshold of minimum cluster abundance set to 5 HNA, in the CS/SG ratio, a high number of clusters (ranging from 70 to 370 depending on the sample analyzed) with fold change greater than 50 was found, where one of the libraries has 0 HNA and the other any number greater than 30 HNA. This fact suggests a very strong modulation of the expression of small RNAs between the two cultivars, which is more or less pronounced depending on the vineyard where the berries were cultivated. A similar situation was observed comparing the expression level of small RNAs between reciprocal hybrids of *Solanum lycopersicum* and *S. pimpinellifolium* (Li et al., 2014).

The ripening process of grapevine berries is highly affected by the environment (van Leeuwen et al., 2004, 2007) and we observed the impact of the environment on the ripening process in the expression of small RNAs. The most relevant observation is that Riccione is very peculiar in relation to the activation of sRNA hotspots, as indicated by the high number of Riccione-specific clusters (Figure 2A) and by the extreme modification it induces in the CS/SG ratio (Figure 5): in Riccione in fact this ratio decreases in green berries and increases in ripened berries, and this is not observed in any other vineyard; in addition to this the already discussed example of BURP domain gene, is observed in Riccione, as well. Riccione is the most diverse environment when compared to Montalcino and Bolgheri. Riccione is located at the Adriatic coast and has a temperate sub-littoral climate, while Montalcino and Bolgheri are both located in Tuscany with typically Mediterranean climate.

Moreover, both cultivars show a peculiar profile of small RNA loci during berries ripening, in Riccione. The expression of small RNA loci in Cabernet Sauvignon berries drastically changed during development, especially when collected in Riccione (Figure 1), not only in the number of active loci but also in the different genic or intergenic disposition: ripened berries have a 2.6-fold increase in small RNA loci active in genic regions. Differently, when Sangiovese is grown in Riccione, there is a very high number of small RNA loci active in green berries, mainly associated to transposable elements that remains almost stable during development although the proportion of intergenic loci is reduced. Sangiovese berries collected in Montalcino show a 2.5-fold increase of small RNA producing loci during development.

Differences during berry development between the cultivars may explain their different behavior in different environments, and the characteristics of each vineyard may favor one or other variety according to their demands. For example, Sangiovese needs a long growing season (it is slow to ripen) with sufficient warmth to fully ripen (Poni, 2000). Consequently, cooler environments will require a reprograming of Sangiovese gene expression in order to achieve ripening. Other factors such as composition of soil, level of humidity, photoperiod and density of cultivation may be exerting the same influence on the ripening of the berries triggering the activation of different small RNA loci.

### miRNAs expression is mainly dependent on the developmental stage but a few miRNAs are directly modulated by the vineyard and the cultivar

Applying a conservative pipeline to the analysis of our 48 small RNA libraries, we recognized 89 known and annotated grapevine miRNAs. In addition, when compared to previous reports in grapevine (Alabi et al., 2012; Han et al., 2014; Wang et al., 2014) we identified 7 completely novel miRNAs plus 26 homologous to other plant species, but novel to grapevine. This is a remarkable number considering the stringency of our pipeline and that our study is based only on four developmental stages of berries.

The outline of miRNA accumulation across samples is different from that of sRNA-producing loci. While the expression of sRNA-generating regions allows distinguishing very well between ripened and green berries and also between cultivars (Figure 4), the accumulation of miRNAs shows a clear distinction only between ripened and green berries, and when the berries were green, we observe a further dichotomy separating the two cultivars and the two green developmental stages. The same pattern of miRNA accumulation among green and ripened berries of grapevine (cv. Corvina) was observed when we described the miRNA expression atlas of *Vitis vinifera* (Kullan et al., 2015).

Comparing the distribution of miRNAs expressed throughout our samples, we found a set of 39 miRNAs ubiquitous (21) or nearly ubiquitous (18) to all the libraries, and very few miRNAs specific of a cultivar, vineyard or developmental stage. All these 39 miRNAs belong to known vvi-miRNA families. With few exceptions, the same set of miRNAs was also found expressed in all the small RNA libraries constructed with different tissues of the grapevine cv. Corvina (Kullan et al., 2015), where the population of expressed miRNAs appears highly variable apart from a well-defined group of miRNAs, probably related to the basal metabolism. These findings are also consistent with previous report in grapevine where a small number of known tissue-specific miRNAs was described (Wang et al., 2014).

Considering the ripening process as shown in the heat maps (Figure 9), and the correlation dendrogram, it is clear that most miRNAs are modulated during the developmental process.

For some miRNA families, we observed the same peculiar patterns of miRNA accumulation, previously described in the grapevine miRNA atlas (Kullan et al., 2015), e.g., an increase of accumulation toward ripening for miR156 f/g/i, and a decrease for miR166c/e, miR172d, miR319, and miR396a/b, but this is not the main focus of our paper.

To establish genotype and environmental influence on miRNA modulation, we performed a statistical analysis that revealed a number of miRNAs differentially expressed. Being aware of the fact that we had only two biological replicates, we applied the exact test as implemented in the EdgeR package. This test has been recently judged a very robust tool that can be used in experiments similar to our, because of its low false positive rate and relative high true positive ratein the presence of a fold change higher than 4 (Schurch et al., 2016).

Considering berries at the same developmental stages, we compared Sangiovese vs. Cabernet Sauvignon in a given vineyard and Montalcino vs. Bolgheri, Montalcino vs. Riccione, and Bolgheri vs. Riccione keeping the cultivar fixed. In total we performed 9 pairwise comparisons for each developmental stage. In general, we observed that berries at 19 °Brix and at harvest show a higher number of differentially expressed miRNAs.

The most interesting examples are represented by two novel miRNAs, whose predicted targets are related to the biosynthesis and accumulation of secondary metabolites, which are of crucial importance in grapevine berries, since its quality depends mainly on its metabolites (Ali et al., 2010). The candidate grape-m1191 is differentially expressed in Sangiovese between Riccione and Bolgheri (Ric_SG_19 vs. Bol_SG_19) and was predicted to target the transparent-testa 12 gene (VIT_212s0028g01160) that encodes a multidrug secondary transporter-like protein (MATE) involved in the vacuolar accumulation of the flavonoid proanthocyanidin in different species including grapevine (Debeaujon et al., 2001; Bogs et al., 2007; Marinova et al., 2007; Zhao et al., 2010). Also, in grapevine some studies provide evidences that the intracellular transport of acylated anthocyanins is catalyzed by a MATE transporter (Gomez et al., 2009; He et al., 2010).

The grape-m1355 seems to be involved in four different pathways, all related to secondary metabolites. It is differentially expressed in Montalcino between the two varieties (Mon_CS_hv vs. Mon_SG_hv) and was predicted to target a cinnamoyl reductase-like protein (CCR) (VIT_203s0110g00350), which is part of the of the polyphenol biosynthetic pathway (Leple et al., 2007); a cinnamyl alcohol dehydrogenase (VIT_206s0004g02380) involved in the lignin biosynthesis (Trabucco et al., 2013); a phenylacetaldehyde reductase (VIT_213s0064g00340), which catalyzes, in tomato, the last step in the synthesis of the volatile 2-phenylethanol, important for the aroma and flavor of many foods (Tieman et al., 2007); and different bifunctional dihydroflavonol 4-reductases (DFR) (see Supplementary Table 3). DFR catalyzes the first step in the conversion of dihydroflavonols to anthocyanins and are responsible for the production of colored anthocyanins (Boss and Davies, 2001; Davies et al., 2003). The same miRNA candidate was described in the grape miRNA atlas (Kullan et al., 2015) also predicted to target several genes of DFR-like and one CCR.

As for known miRNAs, several members of the miR395 family are differentially expressed at 19 °Brix and at harvest in Bolgheri and in both Bolgheri and Riccione, respectively, when comparing the two cultivars. Moreover, miR395f is differentially expressed also in CS at harvest between Montalcino and Bolgheri. This miRNA has been shown to target genes involved in Sulphate assimilation and metabolism (Liang and Yu, 2010; Kawashima et al., 2011; Matthewman et al., 2012), and hence it could be connected to flavonoid and stilbene pathways as suggested by Tavares et al. (2013).

miR399 family members are also differentially expressed in several comparisons: at 19 °Brix between Riccione and Bolgheri in CS and between Riccione and Montalcino in SG, plus in Montalcino between CS and SG. At harvest, miR399 are differentially expressed in SG in all the three comparisons among vineyards and in Riccione between CS and SG. miR399 is implicated in Phosphate homeostasis being rapidly up-regulated upon Pi starvation (Fujii et al., 2005). miR399 regulatory network has been shown to be important in flowering time (Kim et al., 2011) and was identified as a temperature-sensitive miRNA (Lee et al., 2010), however its characterization in fruit ripening is lacking, although intriguing.

miR396 family members are known to be regulated during organ development, targeting Growth Regulating Factors (Liu et al., 2009; Wang et al., 2011) and also in berry development (Kullan et al., 2015; Cao et al., 2016), and we observed their modulation during berry ripening in our data as well, but more interestingly, they are also differentially expressed between CS and SG in berries sampled in Bolgheri at 19 °Brix.

Finally, the investigation of the global relationships of different small RNA classes and miRNAs expressed in different grapevine cultivars, collected in different vineyards and developmental stages, suggests that although the vineyard may influence their profile of abundance it probably does in less proportion than developmental stage and cultivar. Somehow, this behavior would be expected because although the epigenetic state is dynamic and responsive to both developmental and environmental signals, small RNAs in general and even more miRNAs are well known to play numerous crucial roles at each major stage of plants development (Jones-Rhoades et al., 2006; Chen, 2009, 2012). The results here described are in agreement with those reported in the grapevine miRNA atlas (Kullan et al., 2015), especially with respect to the clustering of berries according to their developmental stage, sustaining the idea that miRNAs influence organ identity and clearly separate green and ripened berries. Also, in the study of the grapevine transcriptome performed by Dal Santo et al. (2013), they observed that other factors such as year and developmental stage had more influence on the gene expression, rather than the environment.

## Author contributions

DPP prepared small RNA libraries, performed the *in silico* analysis and wrote the paper. LB conceived the experimental plan and sampled biological material. SDS prepared plant material for RNA extraction, read critically the paper. GDL prepared plant material for RNA extraction, sampled the biological material, read critically the paper. MP conceived the work. MEP supported the lab work, contributed to data analysis and read critically the paper. BM gave a substantial contribution to *in silico* analysis. EM wrote the paper, prepared plant material for RNA extraction, supported small RNA libraries preparation and helped data analysis.

### Conflict of interest statement

The authors declare that the research was conducted in the absence of any commercial or financial relationships that could be construed as a potential conflict of interest.

## References

[B1] Agarwal M. C. J. Chen X. (2009). Endogenous small RNA pathways in *Arabidopsis*, in Regulation of Gene Expression by Small RNAs, Vol. xvii, eds Gaur R. K. Rossi J. J. (Boca Raton, FL: CRC Press), 431.

[B2] Alabi O. J. Zheng Y. Jagadeeswaran G. Sunkar R. Naidu R. A. (2012). High-throughput sequence analysis of small RNAs in grapevine (*Vitis vinifera* L.) affected by grapevine leafroll disease. Mol. Plant Pathol. 13, 1060–1076. 10.1111/j.1364-3703.2012.00815.x 22827483 PMC6638782

[B3] Ali K. Maltese F. Choi Y. H. Verpoorte R. (2010). Metabolic constituents of grapevine and grape-derived products. Phytochem. Rev. 9, 357–378. 10.1007/s11101-009-9158-0 20835385 PMC2928446

[B4] Arikit S. Xia R. Kakrana A. Huang K. Zhai J. Yan Z.. (2014). An atlas of soybean small RNAs identifies phased siRNAs from hundreds of coding genes. Plant Cell 26, 4584–4601. 10.1105/tpc.114.131847 25465409 PMC4311202

[B5] Bernstein E. Allis C. D. (2005). RNA meets chromatin. Genes Dev. 19, 1635–1655. 10.1101/gad.1324305 16024654

[B6] Bogs J. Jaffe F. W. Takos A. M. Walker A. R. Robinson S. P. (2007). The grapevine transcription factor VvMYBPA1 regulates proanthocyanidin synthesis during fruit development. Plant Physiol. 143, 1347–1361. 10.1104/pp.106.093203 17208963 PMC1820911

[B7] Bond D. M. Baulcombe D. C. (2014). Small RNAs and heritable epigenetic variation in plants. Trends Cell Biol. 24, 100–107. 10.1016/j.tcb.2013.08.001 24012194

[B8] Borges F. Martienssen R. A. (2016). The expanding world of small RNAs in plants. Nat. Rev. Mol. Cell Biol. 16, 727–741. 10.1038/nrm4085 26530390 PMC4948178

[B9] Boss P. K. Davies C. (2001). Molecular biology of sugar and anthocyanin accumulation in grape berries, in Molecular Biology and Biotechnology of the Grapevine, ed Roubelakis-Angelakis K. (Dordrecht: Springer), 1–33.

[B10] Bradshaw A. D. (1965). Evolutionary significance of phenotypic plasticity in plants, in Advances in Genetics, eds Caspari E. W. Thoday J. M. (New York, NY: Academic Press), 115–155.

[B11] Burkhart K. B. Guang S. Buckley B. A. Wong L. Bochner A. F. Kennedy S. (2011). A pre-mRNA-associating factor links endogenous siRNAs to chromatin regulation. PLoS Genet. 7:e1002249. 10.1371/journal.pgen.1002249 21901112 PMC3161925

[B12] Callaway R. M. Pennings S. C. Richards C. L. (2003). Phenotypic plasticity and interactions among plants. Ecology 84, 1115–1128. 10.1890/0012-9658(2003)084[1115:PPAIAP]2.0.CO;2

[B13] Cao D. Wang J. Ju Z. Liu Q. Li S. Tian H.. (2016). Regulations on growth and development in tomato cotyledon, flower and fruit via destruction of miR396 with short tandem target mimic. Plant Sci. 247, 1–12. 10.1016/j.plantsci.2016.02.012 27095395

[B14] Carra A. Mica E. Gambino G. Pindo M. Moser C. Pè M. E.. (2009). Cloning and characterization of small non-coding RNAs from grape. Plant J. 59, 750–763. 10.1111/j.1365-313X.2009.03906.x 19453456

[B15] Castel S. E. Martienssen R. A. (2013). RNA interference in the nucleus: roles for small RNAs in transcription, epigenetics and beyond. Nat. Rev. Genet. 14, 100–112. 10.1038/nrg3355 23329111 PMC4205957

[B16] Chen X. (2009). Small RNAs and their roles in plant development. Annu. Rev. Cell Dev. Biol. 25, 21–44. 10.1146/annurev.cellbio.042308.113417 19575669 PMC5135726

[B17] Chen X. (2012). Small RNAs in development - insights from plants. Curr. Opin. Genet. Dev. 22, 361–367. 10.1016/j.gde.2012.04.004 22578318 PMC3419802

[B18] Clingeleffer P. R. (2010). Plant management research: status and what it can offer to address challenges and limitations. Aust. J. Grape Wine Res. 16, 25–32. 10.1111/j.1755-0238.2009.00075.x

[B19] Coombe B. G. (1976). The Development of Fleshy Fruits. Ann. Rev. Plant Phys. 27, 207–228. 10.1146/annurev.pp.27.060176.001231

[B20] Dal Santo S. Tornielli G. B. Zenoni S. Fasoli M. Farina L. Anesi A.. (2013). The plasticity of the grapevine berry transcriptome. Genome Biol. 14:r54. 10.1186/gb-2013-14-6-r54 23759170 PMC3706941

[B21] Davies K. Schwinn K. Deroles S. Manson D. Lewis D. Bloor S. (2003). Enhancing anthocyanin production by altering competition for substrate between flavonol synthase and dihydroflavonol 4-reductase. Euphytica 131, 259–268. 10.1023/A:1024018729349

[B22] Debeaujon I. Peeters A. J. Leon-Kloosterziel K. M. Koornneef M. (2001). The TRANSPARENT TESTA12 gene of *Arabidopsis* encodes a multidrug secondary transporter-like protein required for flavonoid sequestration in vacuoles of the seed coat endothelium. Plant Cell 13, 853–871. 10.1105/tpc.13.4.853 11283341 PMC135529

[B23] DeWitt T. J. Scheiner, S. M. (2003). Phenotypic Plasticity: Functional and Conceptual Approaches. New York, NY: Oxford University Press.

[B24] Duncan E. J. Gluckman P. D. Dearden P. K. (2014). Epigenetics, plasticity, and evolution: how do we link epigenetic change to phenotype? J. Exp. Zool. B Mol. Develop. Evol. 322, 208–220. 10.1002/jez.b.22571 24719220

[B25] Eisen M. B. Spellman P. T. Brown P. O. Botstein D. (1998). Cluster analysis and display of genome-wide expression patterns. Proc. Natl. Acad. Sci. U.S.A. 95, 14863–14868. 9843981 10.1073/pnas.95.25.14863 PMC24541

[B26] Fagegaltier D. Bougé A.-L. Berry B. Poisot É. Sismeiro O. Coppée J.-Y.. (2009). The endogenous siRNA pathway is involved in heterochromatin formation in *Drosophila*. Proc. Natl. Acad. Sci. U.S.A. 106, 21258–21263. 10.1073/pnas.0809208105 19948966 PMC2795490

[B27] Finnegan E. J. Matzke M. A. (2003). The small RNA world. J. Cell Sci. 116, 4689–4693. 10.1242/jcs.00838 14600255

[B28] Formey D. Sallet E. Lelandais-Brière C. Ben C. Bustos-Sanmamed P. Niebel A.. (2014). The small RNA diversity from *Medicago truncatula* roots under biotic interactions evidences the environmental plasticity of the miRNAome. Genome Biol. 15:457. 10.1186/s13059-014-0457-4 25248950 PMC4212123

[B29] Fujii H. Chiou T. J. Lin S. I. Aung K. Zhu J. K. (2005). A miRNA involved in phosphate-starvation response in *Arabidopsis*. Curr. Biol. 15, 2038–2043. 10.1016/j.cub.2005.10.016 16303564

[B30] Gapper N. E. Giovannoni J. J. Watkins C. B. (2014). Understanding development and ripening of fruit crops in an ‘omics’ era. Hort. Res. 1, 14034 10.1038/hortres.2014.34 26504543 PMC4596339

[B31] Ge A. Shangguan L. Zhang X. Dong Q. Han J. Liu H.. (2013). Deep sequencing discovery of novel and conserved microRNAs in strawberry (*Fragaria ananassa*). Physiol. Plant. 148, 387–396. 10.1111/j.1399-3054.2012.01713.x 23061771

[B32] Geng Y. Gao L. Yang J. (2013). Epigenetic flexibility underlying phenotypic plasticity, in Progress in Botany, eds Lüttge U. Beyschlag W. Francis D. Cushman J. (Berlin; Heidelberg: Springer), 153–163.

[B33] Ghildiyal M. Zamore P. D. (2009). Small silencing RNAs: an expanding universe. Nat. Rev. Genet. 10, 94–108. 10.1038/nrg2504 19148191 PMC2724769

[B34] Gianoli E. Valladares F. (2012). Studying phenotypic plasticity: the advantages of a broad approach. Biol. J. Linn. Soc. 105, 1–7. 10.1111/j.1095-8312.2011.01793.x

[B35] Giovannoni J. J. (2007). Fruit ripening mutants yield insights into ripening control. Curr. Opin. Plant Biol. 10, 283–289. 10.1016/j.pbi.2007.04.008 17442612

[B36] Goldberg A. D. Allis C. D. Bernstein E. (2007). Epigenetics: a landscape takes shape. Cell 128, 635–638. 10.1016/j.cell.2007.02.006 17320500

[B37] Gomez C. Terrier N. Torregrosa L. Vialet S. Fournier-Level A. Verriès C.. (2009). Grapevine MATE-type proteins act as vacuolar H+-dependent acylated anthocyanin transporters. Plant Physiol. 150, 402–415. 10.1104/pp.109.135624 19297587 PMC2675721

[B38] Gratani L. (2014). Plant phenotypic plasticity in response to environmental factors. Adv. Bot. 2014:17 10.1155/2014/208747

[B39] Gray J. D. (2002). The Basis of Variation in the Size and Composition of Grape Berries. PhD, University of Adelaide.

[B40] Guillaumie S. Fouquet R. Kappel C. Camps C. Terrier N. Moncomble D.. (2011). Transcriptional analysis of late ripening stages of grapevine berry. BMC Plant Biol. 11:165. 10.1186/1471-2229-11-165 22098939 PMC3233516

[B41] Guleria P. Mahajan M. Bhardwaj J. Yadav S. K. (2011). Plant small RNAs: biogenesis, mode of action and their roles in abiotic stresses. Genom. Proteomics Bioinformatics 9, 183–199. 10.1016/S1672-0229(11)60022-3 22289475 PMC5054152

[B42] Ha M. Lu J. Tian L. Ramachandran V. Kasschau K. D. Chapman E. J.. (2009). Small RNAs serve as a genetic buffer against genomic shock in *Arabidopsis* interspecific hybrids and allopolyploids. Proc. Natl. Acad. Sci. U.S.A. 106, 17835–17840. 10.1073/pnas.0907003106 19805056 PMC2757398

[B43] Han J. Fang J. Wang C. Yin Y. Sun X. Leng X.. (2014). Grapevine microRNAs responsive to exogenous gibberellin. BMC Genomics 15:111. 10.1186/1471-2164-15-111 24507455 PMC3937062

[B44] Harding J. L. Horswell S. Heliot C. Armisen J. Zimmerman L. B. Luscombe N. M.. (2014). Small RNA profiling of *Xenopus* embryos reveals novel miRNAs and a new class of small RNAs derived from intronic transposable elements. Genome Res. 24, 96–106. 10.1101/gr.144469.112 24065776 PMC3875865

[B45] He F. Mu L. Yan G. L. Liang N. N. Pan Q. H. Wang J.. (2010). Biosynthesis of anthocyanins and their regulation in colored grapes. Molecules 15, 9057–9091. 10.3390/molecules15129057 21150825 PMC6259108

[B46] Holloway G. J. (2002). Phenotypic plasticity: beyond nature and nurture. Heredity 89, 410–410. 10.1038/sj.hdy.6800153

[B47] Howell M. D. Fahlgren N. Chapman E. J. Cumbie J. S. Sullivan C. M. Givan S. A.. (2007). Genome-wide analysis of the RNA-DEPENDENT RNA POLYMERASE6/DICER-LIKE4 pathway in *Arabidopsis* reveals dependency on miRNA- and tasiRNA-directed targeting. Plant Cell 19, 926–942. 10.1105/tpc.107.050062 17400893 PMC1867363

[B48] Hu H. Yu D. Liu H. (2015). Bioinformatics analysis of small RNAs in pima (*Gossypium barbadense* L.). PLoS ONE 10:e0116826. 10.1371/journal.pone.0116826 25679373 PMC4332481

[B49] Jaillon O. Aury J. M. Noel B. Policriti A. Clepet C. Casagrande A.. (2007). The grapevine genome sequence suggests ancestral hexaploidization in major angiosperm phyla. Nature 449, 463–467. 10.1038/nature06148 17721507

[B50] Jeong D. H. Park S. Zhai J. Gurazada S. G. De Paoli E. Meyers B. C.. (2011). Massive analysis of rice small RNAs: mechanistic implications of regulated microRNAs and variants for differential target RNA cleavage. Plant Cell 23, 4185–4207. 10.1105/tpc.111.089045 22158467 PMC3269859

[B51] Jeong D. H. Schmidt S. A. Rymarquis L. A. Park S. Ganssmann M. German M. A.. (2013). Parallel analysis of RNA ends enhances global investigation of microRNAs and target RNAs of *Brachypodium distachyon*. Genome Biol. 14:R145. 10.1186/gb-2013-14-12-r145 24367943 PMC4053937

[B52] Jones-Rhoades M. W. Bartel D. P. Bartel B. (2006). MicroRNAs and their regulatory roles in plants. Annu. Rev. Plant Biol. 57, 19–53. 10.1146/annurev.arplant.57.032905.105218 16669754

[B53] Kakrana A. Hammond R. Patel P. Nakano M. Meyers B. C. (2014). sPARTA: a parallelized pipeline for integrated analysis of plant miRNA and cleaved mRNA data sets, including new miRNA target-identification software. Nucleic Acids Res. 42:e139. 10.1093/nar/gku693 25120269 PMC4191380

[B54] Karlova R. van Haarst J. C. Maliepaard C. van de Geest H. Bovy A. G. Lammers M.. (2013). Identification of microRNA targets in tomato fruit development using high-throughput sequencing and degradome analysis. J. Exp. Bot. 64, 1863–1878. 10.1093/jxb/ert049 23487304 PMC3638818

[B55] Kawashima C. G. Matthewman C. A. Huang S. Lee B.-R. Yoshimoto N. Koprivova A.. (2011). Interplay of SLIM1 and miR395 in the regulation of sulfate assimilation in *Arabidopsis*. Plant J. 66, 863–876. 10.1111/j.1365-313X.2011.04547.x 21401744

[B56] Keller M. (2010). Managing grapevines to optimise fruit development in a challenging environment: a climate change primer for viticulturists. Aust. J. Grape Wine Res. 16, 56–69. 10.1111/j.1755-0238.2009.00077.x

[B57] Khraiwesh B. Zhu J. K. Zhu J. (2012). Role of miRNAs and siRNAs in biotic and abiotic stress responses of plants. Biochim. Biophys. Acta 1819, 137–148. 10.1016/j.bbagrm.2011.05.001 21605713 PMC3175014

[B58] Kim V. N. (2005). Small RNAs: classification, biogenesis, and function. Mol. Cells 19, 1–15. 15750334

[B59] Kim W. Ahn H. J. Chiou T.-J. Ahn J. H. (2011). The role of the miR399-*PHO2* module in the regulation of flowering time in response to different ambient temperatures in *Arabidopsis thaliana*. Mol. Cells 32, 83–88. 10.1007/s10059-011-1043-1 21533549 PMC3887651

[B60] Kozomara A. Griffiths-Jones S. (2014). miRBase: annotating high confidence microRNAs using deep sequencing data. Nucleic Acids Res. 42, D68–D73. 10.1093/nar/gkt1181 24275495 PMC3965103

[B61] Kullan J. B. Paim Pinto D. L. Bertolini E. Fasoli M. Zenoni S. Tornielli G. B.. (2015). miRVine: a microRNA expression atlas of grapevine based on small RNA sequencing. BMC Genomics 16:393. 10.1186/s12864-015-1610-5 25981679 PMC4434875

[B62] Langmead B. Trapnell C. Pop M. Salzberg S. L. (2009). Ultrafast and memory-efficient alignment of short DNA sequences to the human genome. Genome Biol. 10:R25. 10.1186/gb-2009-10-3-r25 19261174 PMC2690996

[B63] Lee H. Yoo S. J. Lee J. H. Kim W. Yoo S. K. Fitzgerald H.. (2010). Genetic framework for flowering-time regulation by ambient temperature-responsive miRNAs in *Arabidopsis*. Nucleic Acids Res. 38, 3081–3093. 10.1093/nar/gkp1240 20110261 PMC2875011

[B64] Lee T.-F. Gurazada S. G. R. Zhai J. Li S. Simon S. A. Matzke M. A.. (2012). RNA polymerase V-dependent small RNAs in *Arabidopsis* originate from small, intergenic loci including most SINE repeats. Epigenetics 7, 781–795. 10.4161/epi.20290 22647529 PMC3679228

[B65] Lelandais-Briere C. Sorin C. Crespi M. Hartmann C. (2012). Non-coding RNAs involved in plant responses to environmental constraints. Biol. Aujourdhui. 206, 313–322. 10.1051/jbio/2012032 23419258

[B66] Leple J. C. Dauwe R. Morreel K. Storme V. Lapierre C. Pollet B.. (2007). Downregulation of cinnamoyl-coenzyme A reductase in poplar: multiple-level phenotyping reveals effects on cell wall polymer metabolism and structure. Plant Cell 19, 3669–3691. 10.1105/tpc.107.054148 18024569 PMC2174873

[B67] Li H. Deng Y. Wu T. Subramanian S. Yu O. (2010). Misexpression of miR482, miR1512, and miR1515 increases soybean nodulation. Plant Physiol. 153, 1759–1770. 10.1104/pp.110.156950 20508137 PMC2923892

[B68] Li J. Sun Q. Yu N. Zhu J. Zou X. Qi Z.. (2014). The role of small RNAs on phenotypes in reciprocal hybrids between *Solanum lycopersicum* and S. pimpinellifolium. BMC Plant Biol. 14:296. 10.1186/s12870-014-0296-1 25367629 PMC4232637

[B69] Liang G. Yu D. (2010). Reciprocal regulation among miR395, APS and SULTR2;1 in *Arabidopsis thaliana*. Plant Signal. Behav. 5, 1257–1259. 10.4161/psb.5.10.12608 20935495 PMC3115361

[B70] Lijavetzky D. Carbonell-Bejerano P. Grimplet J. Bravo G. Flores P. Fenoll J.. (2012). Berry flesh and skin ripening features in *Vitis vinifera* as assessed by transcriptional profiling. PLoS ONE 7:e39547. 10.1371/journal.pone.0039547 22768087 PMC3386993

[B71] Lind M. I. Yarlett K. Reger J. Carter M. J. Beckerman A. P. (2015). The alignment between phenotypic plasticity, the major axis of genetic variation and the response to selection. Proc. Biol. Sci. 282:20151651. 10.1098/rspb.2015.1651 26423845 PMC4614775

[B72] Liu D. Song Y. Chen Z. Yu D. (2009). Ectopic expression of miR396 suppresses GRF target gene expression and alters leaf growth in *Arabidopsis*. Physiol. Plant. 136, 223–236. 10.1111/j.1399-3054.2009.01229.x 19453503

[B73] Liu R. How-Kit A. Stammitti L. Teyssier E. Rolin D. Mortain-Bertrand A.. (2015). A DEMETER-like DNA demethylase governs tomato fruit ripening. Proc. Natl. Acad. Sci. U.S.A. 112, 10804–10809. 10.1073/pnas.1503362112 26261318 PMC4553810

[B74] Manning K. Tör M. Poole M. Hong Y. Thompson A. J. King G. J.. (2006). A naturally occurring epigenetic mutation in a gene encoding an SBP-box transcription factor inhibits tomato fruit ripening. Nat. Genet. 38, 948–952. 10.1038/ng1841 16832354

[B75] Marinova K. Pourcel L. Weder B. Schwarz M. Barron D. Routaboul J.-M.. (2007). The *Arabidopsis* MATE transporter TT12 acts as a vacuolar flavonoid/H(+)-antiporter active in proanthocyanidin-accumulating cells of the seed coat. Plant Cell 19, 2023–2038. 10.1105/tpc.106.046029 17601828 PMC1955721

[B76] Martínez-Esteso M. J. Vilella-Antón M. T. Pedreño M. Á. Valero M. L. Bru-Martínez R. (2013). iTRAQ-based protein profiling provides insights into the central metabolism changes driving grape berry development and ripening. BMC Plant Biol. 13:167. 10.1186/1471-2229-13-167 24152288 PMC4016569

[B77] Matas A. J. Yeats T. H. Buda G. J. Zheng Y. Chatterjee S. Tohge T.. (2011). Tissue- and cell-type specific transcriptome profiling of expanding tomato fruit provides insights into metabolic and regulatory specialization and cuticle formation. Plant Cell. 23, 3893–3910. 10.1105/tpc.111.091173 22045915 PMC3246317

[B78] Matsui A. Nguyen A. H. Nakaminami K. Seki M. (2013). *Arabidopsis* non-coding RNA regulation in abiotic stress responses. Int. J. Mol. Sci. 14, 22642–22654. 10.3390/ijms141122642 24252906 PMC3856082

[B79] Matthewman C. A. Kawashima C. G. Húska D. Csorba T. Dalmay T. Kopriva S. (2012). miR395 is a general component of the sulfate assimilation regulatory network in *Arabidopsis*. FEBS Lett. 586, 3242–3248. 10.1016/j.febslet.2012.06.044 22771787

[B80] Matus J. T. Aquea F. Espinoza C. Vega A. Cavallini E. Dal Santo S.. (2014). Inspection of the grapevine BURP superfamily highlights an expansion of RD22 genes with distinctive expression features in berry development and ABA-mediated stress responses. PLoS ONE 9:e110372. 10.1371/journal.pone.0110372 25330210 PMC4199669

[B81] McCormick K. P. Willmann M. R. Meyers B. C. (2011). Experimental design, preprocessing, normalization and differential expression analysis of small RNA sequencing experiments. Silence 2:2. 10.1186/1758-907X-2-2 21356093 PMC3055805

[B82] Meyers B. C. Axtell M. J. Bartel B. Bartel D. P. Baulcombe D. Bowman J. L.. (2008). Criteria for annotation of plant MicroRNAs. Plant Cell 20, 3186–3190. 10.1105/tpc.108.064311 19074682 PMC2630443

[B83] Moxon S. Jing R. Szittya G. Schwach F. Rusholme Pilcher R. L. Moulton V.. (2008). Deep sequencing of tomato short RNAs identifies microRNAs targeting genes involved in fruit ripening. Genome Res. 18, 1602–1609. 10.1101/gr.080127.108 18653800 PMC2556272

[B84] Nicotra A. B. Atkin O. K. Bonser S. P. Davidson A. M. Finnegan E. J. Mathesius U.. (2010). Plant phenotypic plasticity in a changing climate. Trends Plant Sci. 15, 684–692. 10.1016/j.tplants.2010.09.008 20970368

[B85] Ortega-Regules A. Romero-Cascales I. Lopez-Roca J. M. Ros-Garcia J. M. Gomez-Plaza E. (2006). Anthocyanin fingerprint of grapes: environmental and genetic variations. J. Sci. Food Agric. 86, 1460–1467. 10.1002/jsfa.2511

[B86] Palmer C. M. Bush S. M. Maloof J. N. (2012). Phenotypic and developmental plasticity in plants, in *Encyclopedia of Life Sciences* (Chichester: John Wiley & Sons, Ltd.), 1–9.

[B87] Pantaleo V. Szittya G. Moxon S. Miozzi L. Moulton V. Dalmay T.. (2010). Identification of grapevine microRNAs and their targets using high-throughput sequencing and degradome analysis. Plant J. 62, 960–976. 10.1111/j.0960-7412.2010.04208.x 20230504

[B88] Pigliucci M. (2001). Phenotypic Plasticity: Beyond Nature and Nurture. Baltimore, MD: John Hopkins University Press.

[B89] Pigllucci M. (1996). How organisms respond to environmental changes: from phenotypes to molecules (and vice versa). Trends Ecol. Evol. 11, 168–173. 10.1016/0169-5347(96)10008-2 21237793

[B90] Poni S. (2000). Fisiologia di comportamento del Sangiovese. Aspetti di base e considerazioni applicative in Il Sangiovese: Atti del Simposio Internazionale, ed ARSIA, (Firenze: ARSIA).

[B91] Provenzano S. (2011). The Genetics of Anthocyanin Production, Accumulation and Display: A Comparative Study in Different species. PhD, Vrije Universiteit.

[B92] Rustioni L. Rossoni M. Failla O. Scienza A. (2013). Anthocyanin esterification in Sangiovese grapes. Ital. J. Food Sci. 25, 131–141.

[B93] Sadras V. O. Stevens R. M. Pech J. M. Taylor E. J. Nicholas P. R. McCarthy M. G. (2007). Quantifying phenotypic plasticity of berry traits using an allometric-type approach: a case study on anthocyanins and sugars in berries of Cabernet Sauvignon. Aust. J. Grape Wine Res. 13, 72–80. 10.1111/j.1755-0238.2007.tb00237.x

[B94] Schlichting C. D. Pigliucci M. (1993). Control of phenotypic plasticity via regulatory genes. Am. Nat. 142, 366–370. 10.1086/285543 19425982

[B95] Schmitt J. (1993). Reaction norms of morphological and life-history traits to light availability in Impatiens capensi. Evolution 47, 1654–1668. 10.1111/j.1558-5646.1993.tb01258.x 28568001

[B96] Schmitt J. Dudley S. Pigliucci M. (1999). Manipulative approaches to testing adaptive plasticity: phytochrome−mediated shade−avoidance responses in plants. Am. Nat. 154, S43–S54. 10.1086/303282 29586708

[B97] Schurch N. J. Schofield P. Gierliñski M. Cole C. Sherstnev A. Singh V.. (2016). How many biological replicates are needed in an RNA-seq experiment and which differential expression tool should you use? RNA 22, 839–851. 10.1261/rna.058339.116 27022035 PMC4878611

[B98] Selvaraj Y. Pal D. K. Singh R. Roy T. K. (1994). Biochemistry of uneven ripening in Gulabi grape. J. Food Biochem. 18, 325–340. 10.1111/j.1745-4514.1994.tb00507.x

[B99] Stocks M. B. Moxon S. Mapleson D. Woolfenden H. C. Mohorianu I. Folkes L.. (2012). The UEA sRNA workbench: a suite of tools for analysing and visualizing next generation sequencing microRNA and small RNA datasets. Bioinformatics 28, 2059–2061. 10.1093/bioinformatics/bts311 22628521 PMC3400958

[B100] Sultan S. E. (2000). Phenotypic plasticity for plant development, function and life history. Trends Plant Sci. 5, 537–542. 10.1016/S1360-1385(00)01797-0 11120476

[B101] Sun X. Korir N. K. Han J. Shangguan L. F. Kayesh E. Leng X. P.. (2012). Characterization of grapevine microR164 and its target genes. Mol. Biol. Rep. 39, 9463–9472. 10.1007/s11033-012-1811-9 22733489

[B102] Swami M. (2010). An epigenetic silencing influence. Nat. Rev. Genet. 11:172. 10.1038/nrg2755 21485430

[B103] Tavares S. Vesentini D. Fernandes J. C. Ferreira R. B. Laureano O. Ricardo-Da-Silva J. M.. (2013). *Vitis vinifera* secondary metabolism as affected by sulfate depletion: diagnosis through phenylpropanoid pathway genes and metabolites. Plant Physiol. Biochem. 66, 118–126. 10.1016/j.plaphy.2013.01.022 23500714

[B104] Tieman D. M. Loucas H. M. Kim J. Y. Clark D. G. Klee H. J. (2007). Tomato phenylacetaldehyde reductases catalyze the last step in the synthesis of the aroma volatile 2-phenylethanol. Phytochemistry 68, 2660–2669. 10.1016/j.phytochem.2007.06.005 17644147

[B105] Trabucco G. M. Matos D. A. Lee S. J. Saathoff A. J. Priest H. D. Mockler T. C.. (2013). Functional characterization of cinnamyl alcohol dehydrogenase and caffeic acid O-methyltransferase in *Brachypodium distachyon*. BMC Biotechnol. 13:61. 10.1186/1472-6750-13-61 23902793 PMC3734214

[B106] Trindade I. S. D. Dalmay T. Fevereiro P. (2011). Facing the environment: small RNAs and the regulation of gene expression under abiotic stress in plants, in Abiotic Stress Response in Plants - Physiological, Biochemical and Genetic Perspectives, ed Shanker A. V. (InTech), 113–136. Available online at: http://www.intechopen.com/books/abiotic-stress-response-in-plants-physiological-biochemical-and-genetic-perspectives

[B107] van Leeuwen C. Friant P. Choné X. Tregoat O. Koundouras S. Dubourdieu D. (2004). Influence of climate, soil, and cultivar on Terroir. Am. J. Enol. Vitic. 55, 207–217.

[B108] van Leeuwen C. T. G. O. Choneì X. GaudilleÌre J. P. Pernet, D. (2007). Different environmental conditions, different results: the role of controlled environmental stress on grape quality and the way to monitor it, in Proceedings of the Thirteenth Australian Wine Industry Technical Conference, ed. Williams P. J. B. Pretorius R. J. (Adelaide: Australian Wine Industry Technical Conference Incorporated), 400.

[B109] Vazquez F. (2006). *Arabidopsis* endogenous small RNAs: highways and byways. Trends Plant Sci. 11, 460–468. 10.1016/j.tplants.2006.07.006 16893673

[B110] Vitulo N. Forcato C. Carpinelli E. C. Telatin A. Campagna D. D'Angelo M.. (2014). A deep survey of alternative splicing in grape reveals changes in the splicing machinery related to tissue, stress condition and genotype. BMC Plant Biol. 14:99. 10.1186/1471-2229-14-99 24739459 PMC4108029

[B111] Wang C. Han J. Liu C. Kibet K. N. Kayesh E. Shangguan L.. (2012). Identification of microRNAs from Amur grape *(Vitis amurensis* Rupr.) by deep sequencing and analysis of microRNA variations with bioinformatics. BMC Genomics 13:122. 10.1186/1471-2164-13-122 22455456 PMC3353164

[B112] Wang C. Leng X. Zhang Y. Kayesh E. Zhang Y. Sun X.. (2014). Transcriptome-wide analysis of dynamic variations in regulation modes of grapevine microRNAs on their target genes during grapevine development. Plant Mol. Biol. 84, 269–285. 10.1007/s11103-013-0132-2 24081692

[B113] Wang L. Gu X. Xu D. Wang W. Wang H. Zeng M.. (2011). miR396-targeted AtGRF transcription factors are required for coordination of cell division and differentiation during leaf development in Arabidopsis. J. Exp. Bot. 62, 761–773. 10.1093/jxb/erq307 21036927 PMC3003814

[B114] Xia S. Cheng Y. T. Huang S. Win J. Soards A. Jinn T.-L.. (2013). Regulation of transcription of nucleotide-binding leucine-rich repeat-encoding genes SNC1 and RPP4 via H3K4 trimethylation. Plant Physiol. 162, 1694–1705. 10.1104/pp.113.214551 23690534 PMC3707539

[B115] Xu F. Liu Q. Chen L. Kuang J. Walk T. Wang J.. (2013). Genome-wide identification of soybean microRNAs and their targets reveals their organ-specificity and responses to phosphate starvation. BMC Genomics 14, 66–66. 10.1186/1471-2164-14-66 23368765 PMC3673897

[B116] Zhai J. Jeong D.-H. De Paoli E. Park S. Rosen B. D. Li Y.. (2011). MicroRNAs as master regulators of the plant NB-LRR defense gene family via the production of phased, trans-acting siRNAs. Genes Dev. 25, 2540–2553. 10.1101/gad.177527.111 22156213 PMC3243063

[B117] Zhao J. Pang Y. Dixon R. A. (2010). The mysteries of proanthocyanidin transport and polymerization. Plant Physiol. 153, 437–443. 10.1104/pp.110.155432 20388668 PMC2879784

[B118] Zhong S. Fei Z. Chen Y. R. Zheng Y. Huang M. Vrebalov J.. (2013). Single-base resolution methylomes of tomato fruit development reveal epigenome modifications associated with ripening. Nat. Biotechnol. 31, 154–159. 10.1038/nbt.2462 23354102

[B119] Zhu Q.-H. Fan L. Liu Y. Xu H. Llewellyn D. Wilson I. (2013). miR482 Regulation of NBS-LRR Defense Genes during Fungal Pathogen Infection in Cotton. PLoS ONE 8:e84390. 10.1371/journal.pone.0084390 24391949 PMC3877274

[B120] Zuker M. (2003). Mfold web server for nucleic acid folding and hybridization prediction. Nucleic Acids Res. 31, 3406–3415. 10.1093/nar/gkg595 12824337 PMC169194

