# Supplementary material for: The Influence of Genotype and Environment on Small RNA Profiles in Grapevine Berry
Source: Front Plant Sci. 2016 Oct 5;7:1459. doi: 10.3389/fpls.2016.01459 (PMC5050227; doi:10.3389/fpls.2016.01459)
Supplement: Supplementary file 1 [file DataSheet1.pdf]

Supplementary Material

The Influence of Genotype and Environment on small RNA Profiles in Grapevine Berry

Daniela Lopes Paim Pinto, Lucio Brancadoro, Silvia Dal Santo, Gabriella De Lorenzis, Mario Pezzotti, Blake C. Meyers, Mario Enrico Pè, Erica Mica *

*** Correspondence:** Erica Mica: erica.mica@crea.gov.it

1 Supplementary Figures

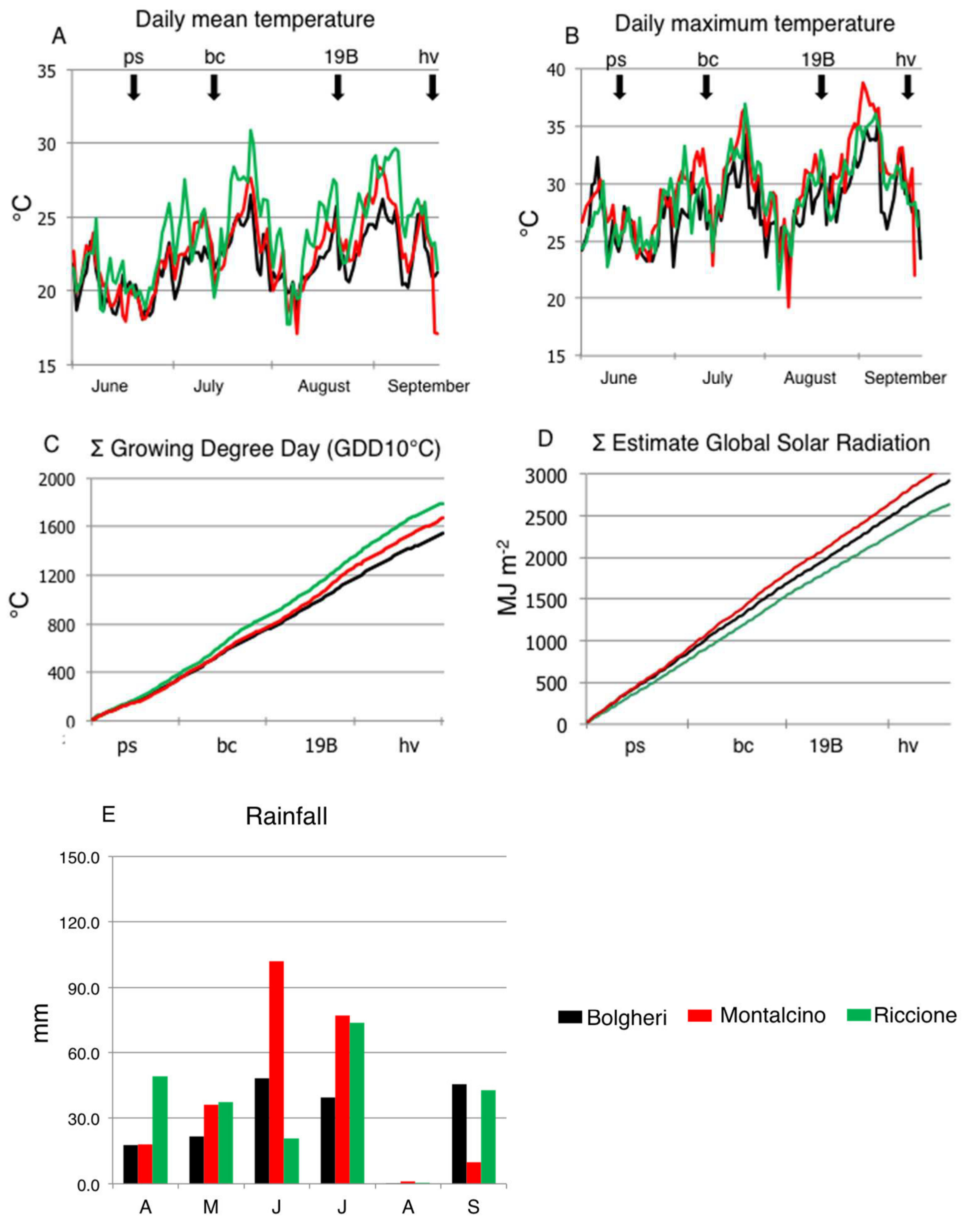

Supplementary Figure 1. Details of the environmental conditions of vineyards. A: The **Daily Mean Temperature** (Td) was generally higher in Riccione, followed by Montalcino. Bolgheri had the Td slightly lower than Montalcino. The difference on the Td among the vineyards was more pronounced towards the end of August and beginning of September.

B: The **Daily Maximum Temperature** (Tx) of the 3 vineyards was in general similar during all the stages of the berries development. A very low value occurred during the month of August for all vineyards, however the lower peak was less pronounced in Bolgheri. Montalcino showed the higher

Tx (39˚C) at the beginning of September as well as Bolgheri (36˚C), while Riccione had the highest Tx (37˚C) at the end of July.

C: The Growing Degree Day ($GDD_{10°C}$) gradually increased from the ps towards the hv stage. During the ps stage the three vineyards showed the same rate of increase. From the transition between ps and the bc stage to the end of berry ripening (hv), Riccione had higher $GDD_{10°C}$. Bolgheri and Montalcino showed almost the same $GDD_{10°C}$ until middle of 19B stage. From this point to the end of berry ripening (hv) Montalcino showed higher $GDD_{10°C}$ than Bolgheri, but still smaller than Riccione. At the end of the ripening process, Riccione reached 1721 $GDD_{10°C}$, Montalcino 1564 $GDD_{10°C}$ and Bolgheri 1465 $GDD_{10°C}$.

D: The **Estimate Global Solar Radiation** (Rs) also had a linear increase in the three vineyards, from the ps towards the hv stage. Montalcino had the higher Rs followed by Bolgheri and then Riccione. Bolgheri and Montalcino showed nearly the same rate during the ps stage. From bc to hv the difference between the two vineyards increased. On the other hand, Riccione had the lower Rs during all the stages.

E: Bolgheri had the lower average **Rainfall** (28,7 mm) from April to September. During the period from May to July, Bolgheri had always the lower rainfall. Montalcino had the higher average rainfall (40,5 mm) followed by Riccione (37,2 mm). In Montalcino the month of June followed by July was responsible for the major part of the precipitation. The highest peak of rainfall in Montalcino was in June reaching 102.0 mm. In this same month, Bolgheri and Riccione showed much lower values, 48.1 and 20.6 mm respectively, which was also the most rainfall in Bolgheri. Riccione reached the most rainfall (73,6 mm) in July. August was the driest month in all vineyards, with no precipitation.

ps = pea size; bc = bunch closure; 19B = 19 ˚Brix; hv = harvest.

*Brancadoro, L.; Zenoni, S. Analisi dell'interazione genotipo-ambiente in Sangiovese e Cabernet Sauvignon attraverso l'espressione globale dei geni. 2013. Unpublished data.

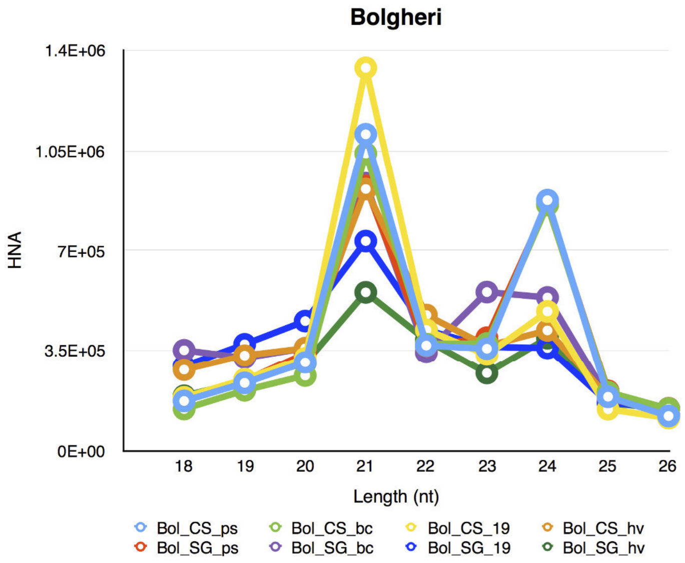

Bolgheri
1.4E+06
1.05E+06
7E+05
3.5E+05
0E+00
HNA
18
19
20
21
22
23
24
25
26
Length (nt)
Bol_CS_ps
Bol_SG_ps
Bol_CS_bc
Bol_SG_bc
Bol_CS_19
Bol_SG_19
Bol_CS_hv
Bol_SG_hv

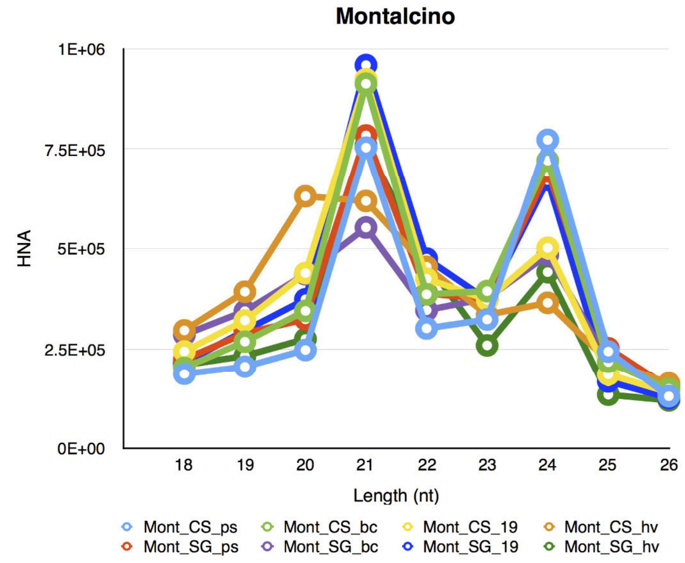

Montalcino
1E+06
7.5E+05
5E+05
2.5E+05
0E+00
HNA
18
19
20
21
22
23
24
25
26
Length (nt)
Mont_CS_ps
Mont_SG_ps
Mont_CS_bc
Mont_SG_bc
Mont_CS_19
Mont_SG_19
Mont_CS_hv
Mont_SG_hv

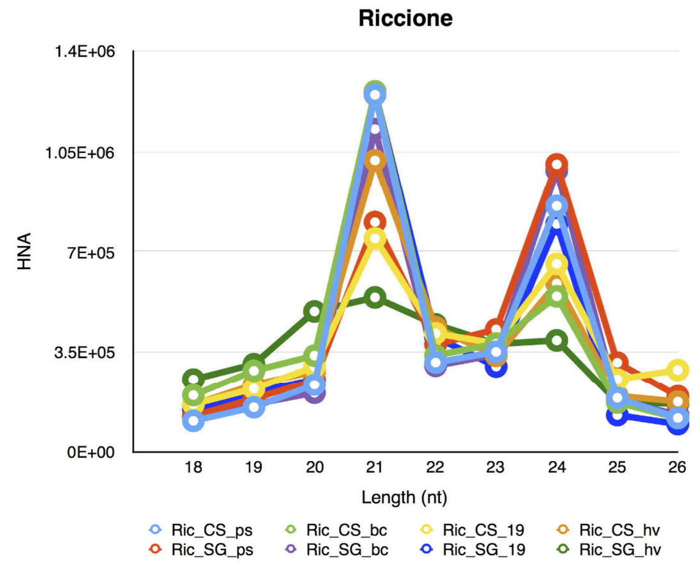

Riccione
1.4E+06
1.05E+06
7E+05
3.5E+05
0E+00
HNA
18
19
20
21
22
23
24
25
26
Length (nt)
Ric_CS_ps
Ric_SG_ps
Ric_CS_bc
Ric_SG_bc
Ric_CS_19
Ric_SG_19
Ric_CS_hv
Ric_SG_hv

Supplementary Figure 2: Small RNA size distributions from grapevine berries. The X-axis indicates the size in nucleotides (nt) and the Y-axis indicates the "hits-normalized- abundance" (HNA) for each size class as an average of two biological replicates. Small RNA size profiles are grouped by vineyard as indicated, and the library codes are specified in Table 1.

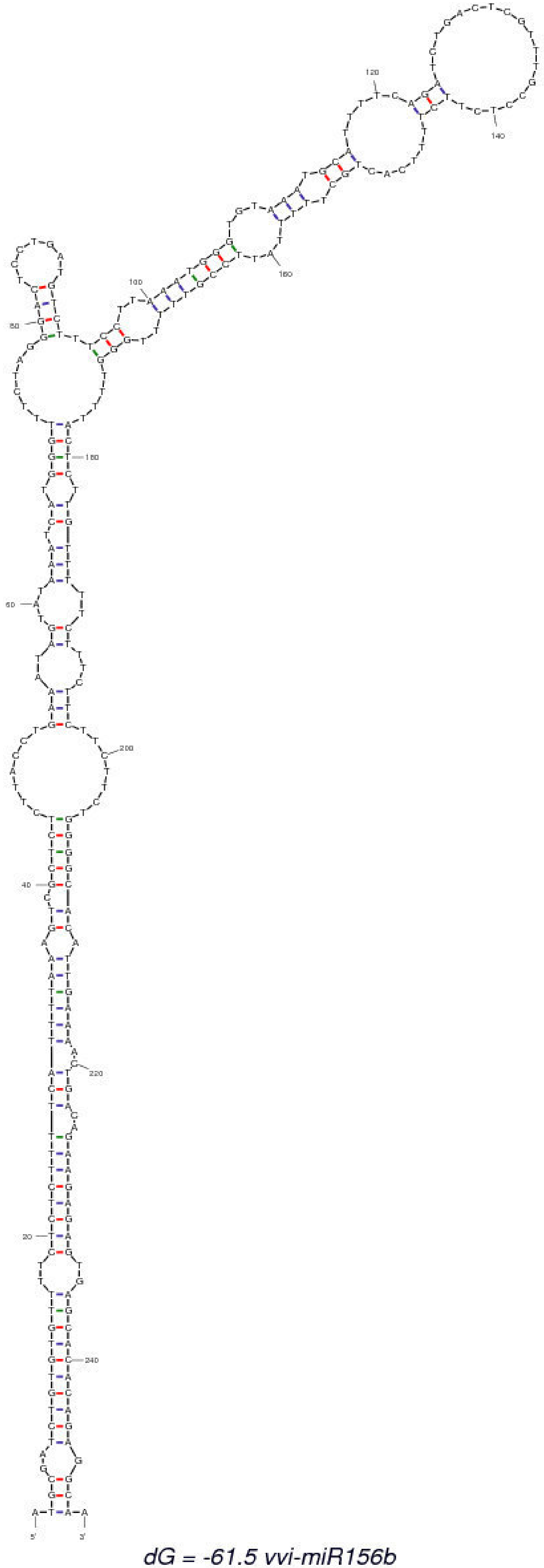

dG = -61.5 vvi-miR156b

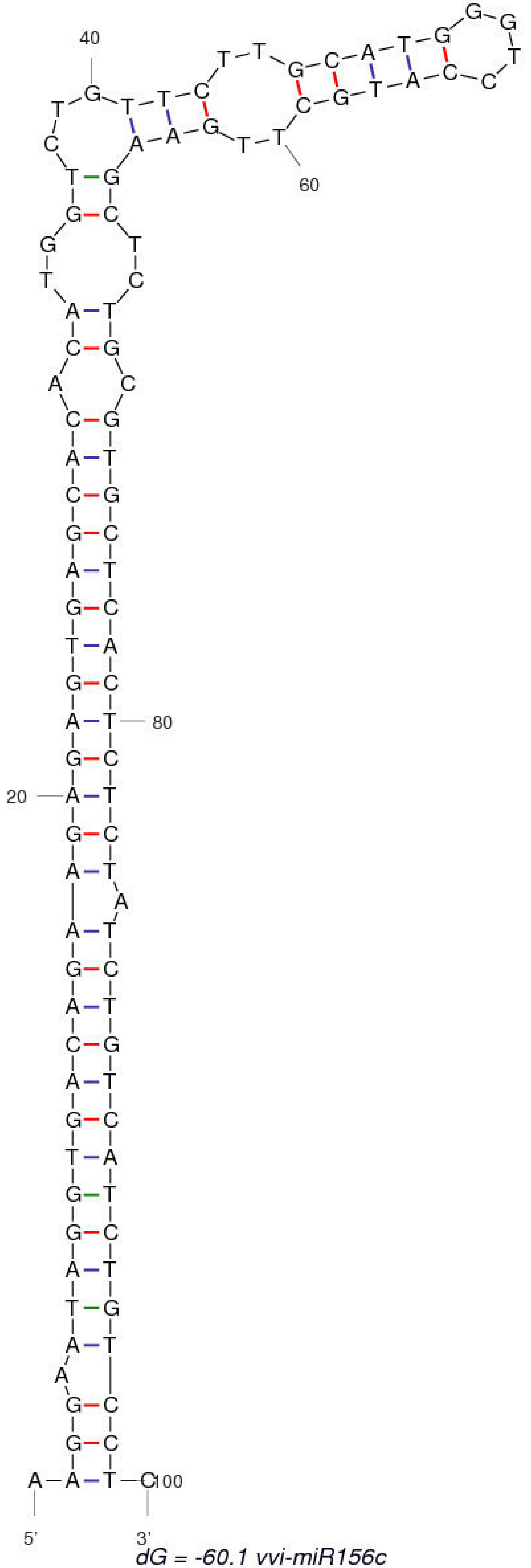

dG = -60.1 vvi-miR156c

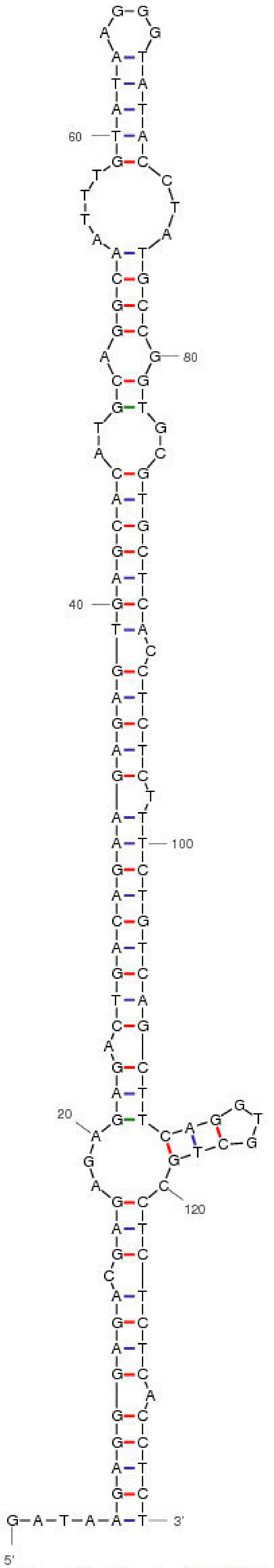

dG = -60.7 vvi-miR156d

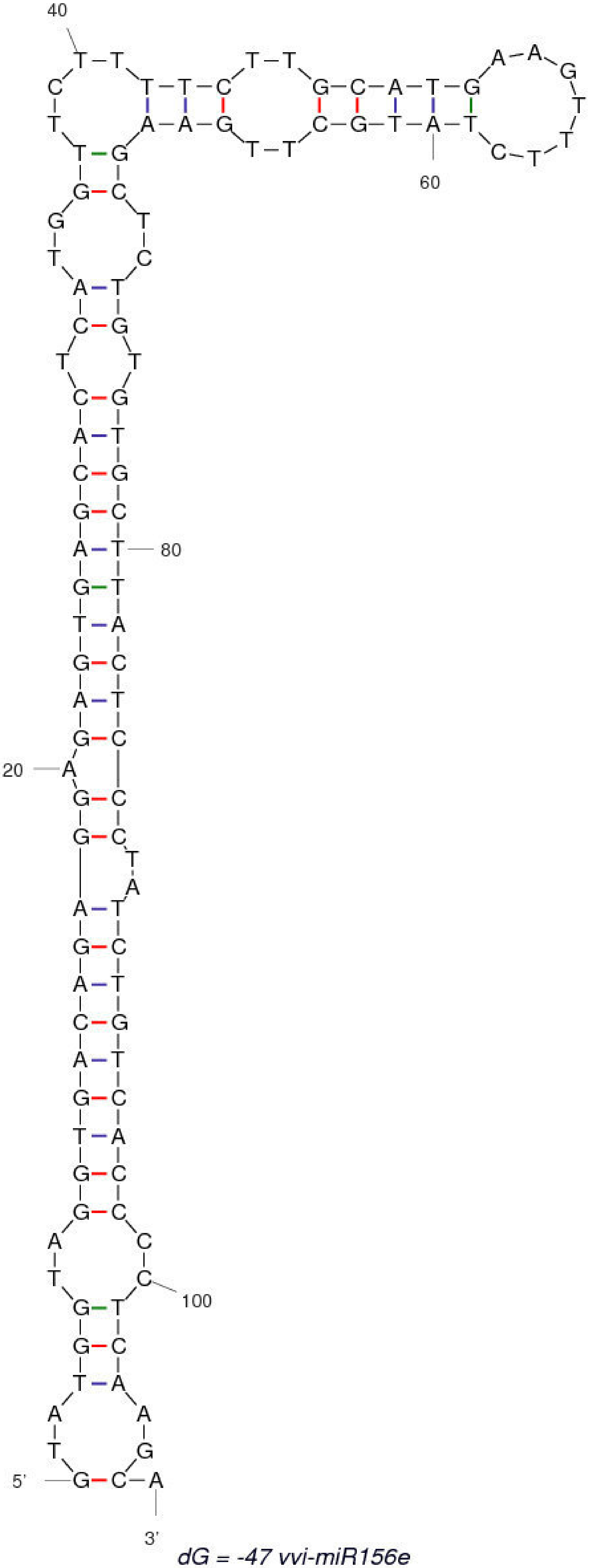

dG = -47 vvi-miR156e

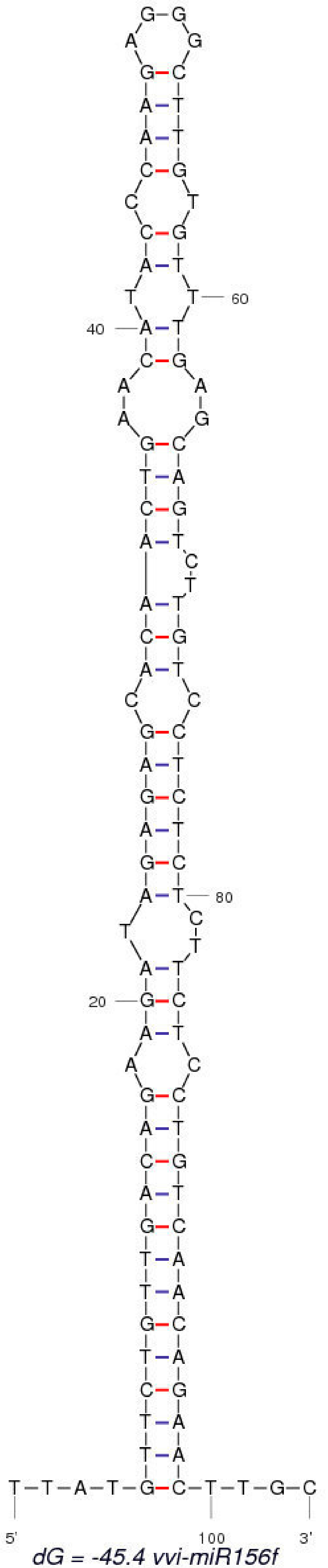

dG = -45.4 vvi-miR156f

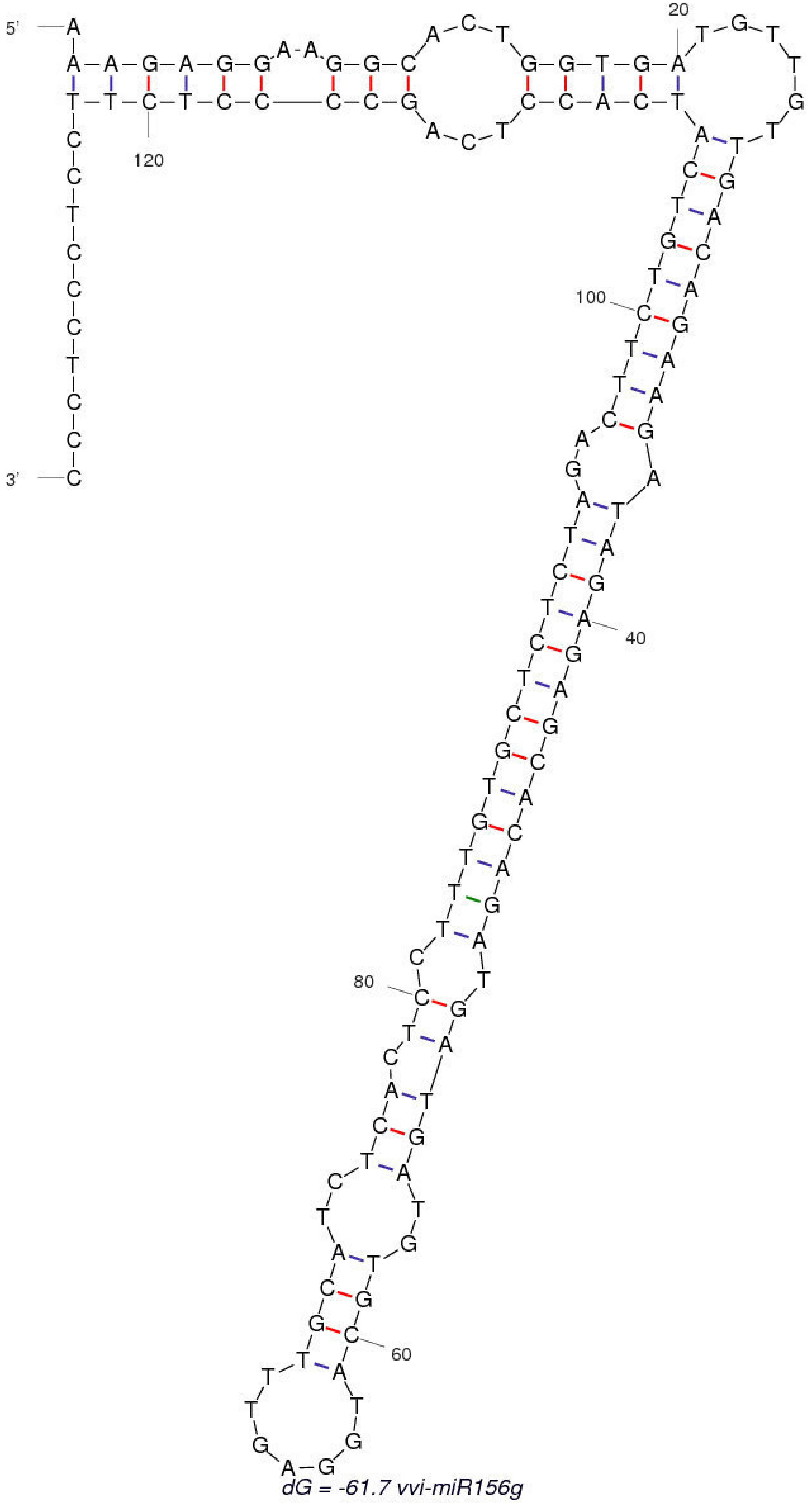

dG = -61.7 vvi-miR156g

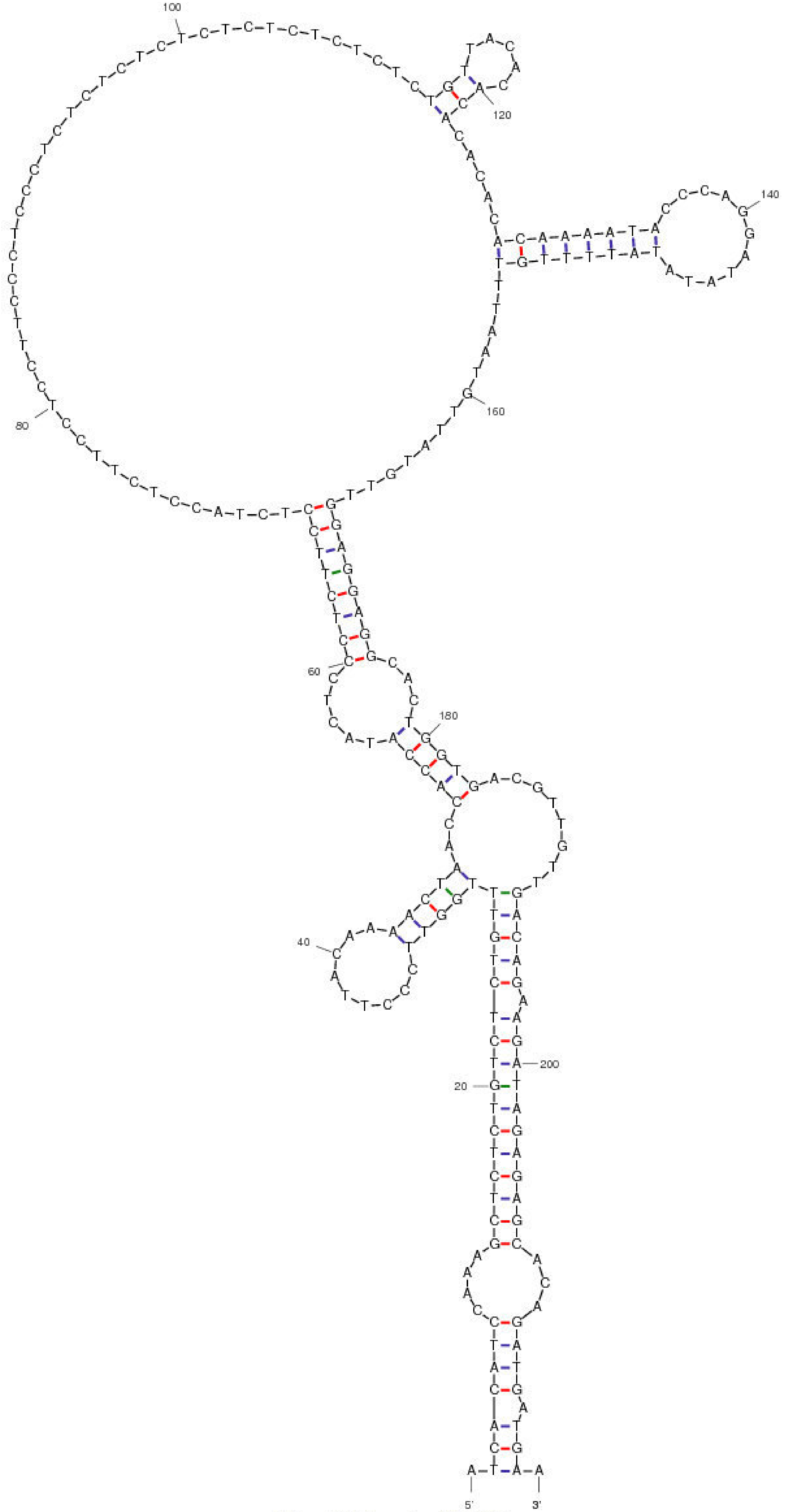

dG = -51.2 vvi-miR156i

Output of sir_graph (©)
mfold_util 4.7

Created Tue Aug 30 07:57:33 2016

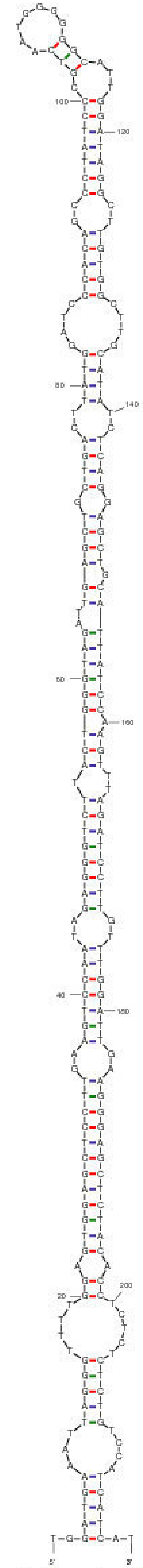

dG = -99.3 vvi-miR159c

Output of sir_graph (©)
mfold_util 4.7

Created Tue Aug 30 07:57:33 2016

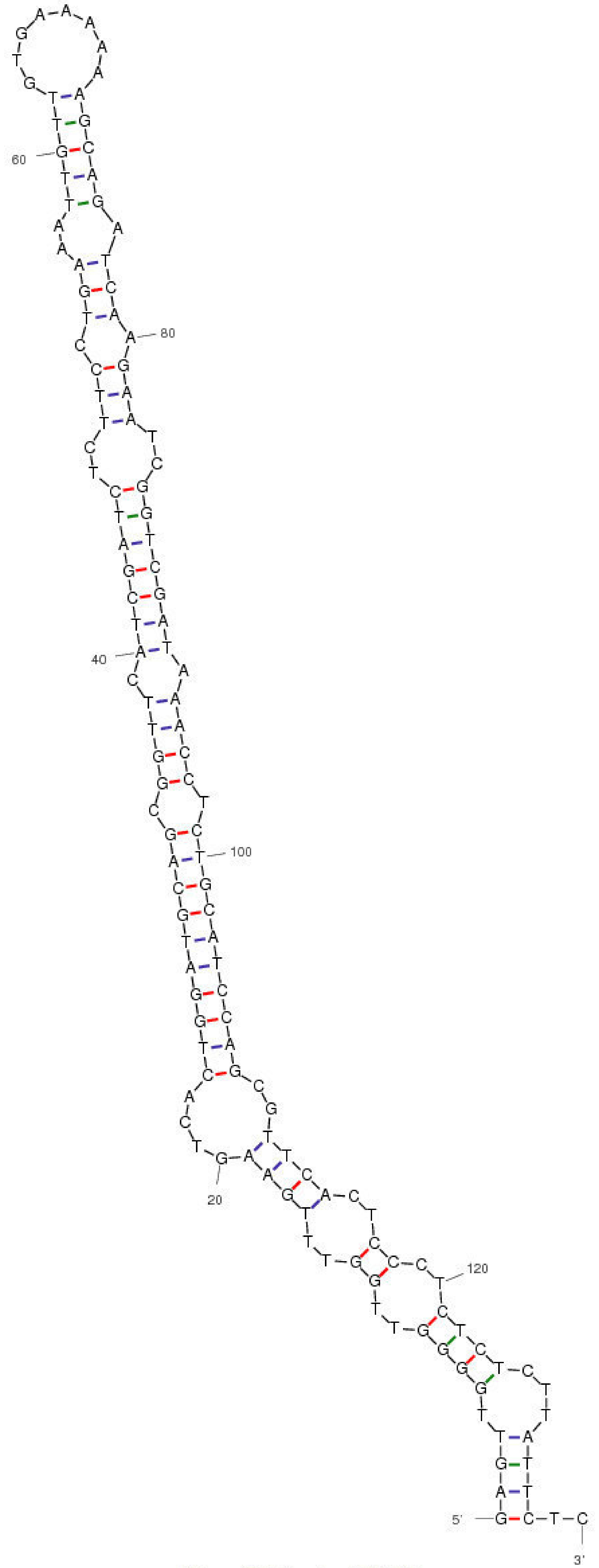

dG = -50.4 vvi-miR162

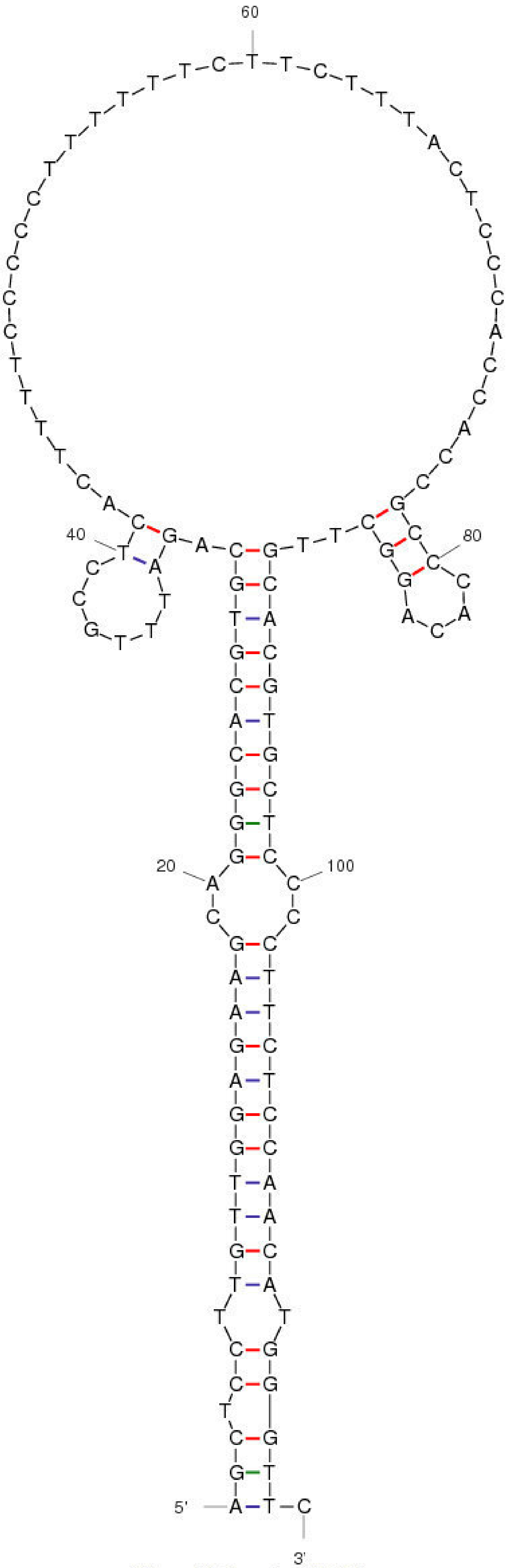

dG = -44.9 vvi-miR164a

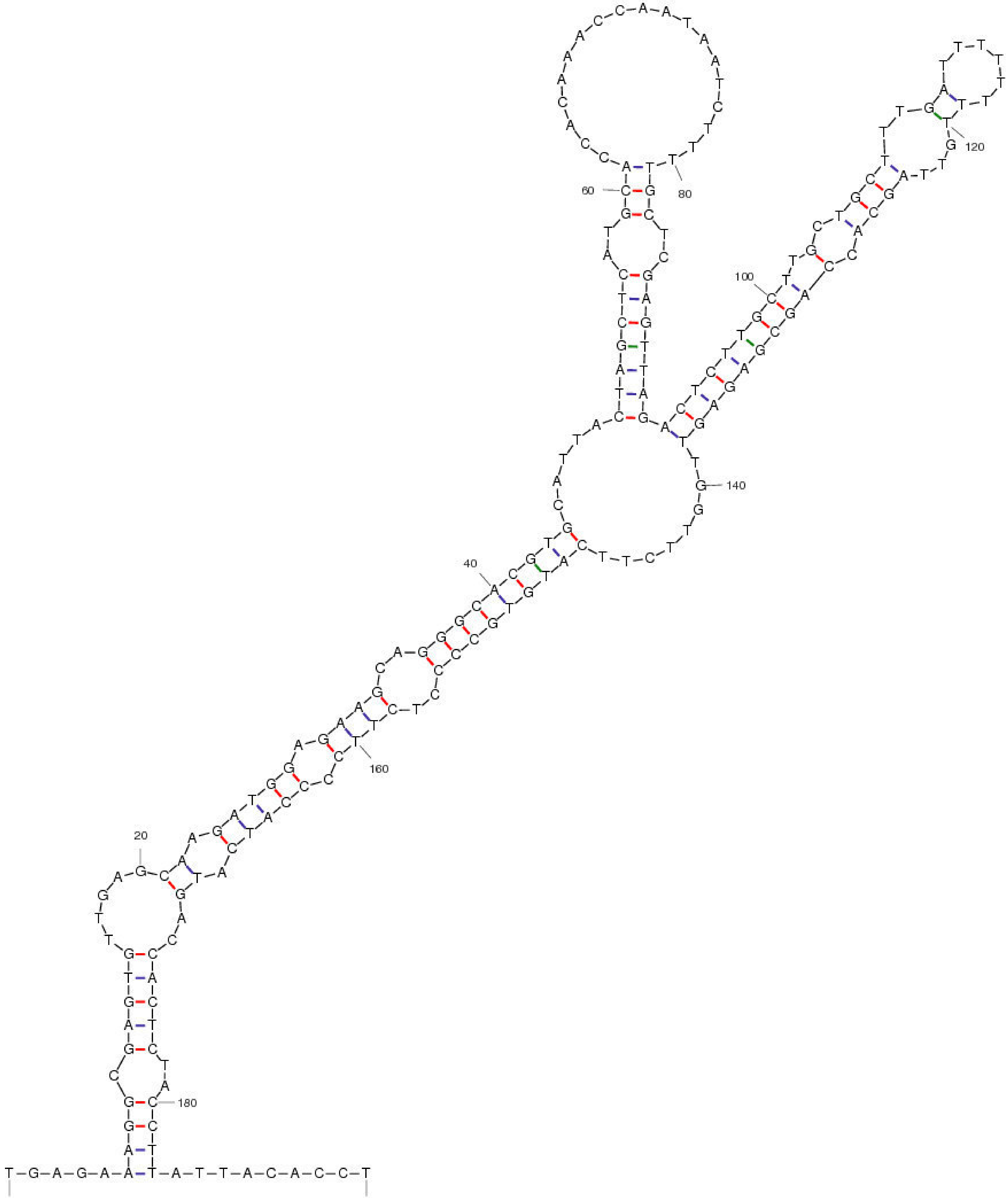

dG = -64.1 vvi-miR164c

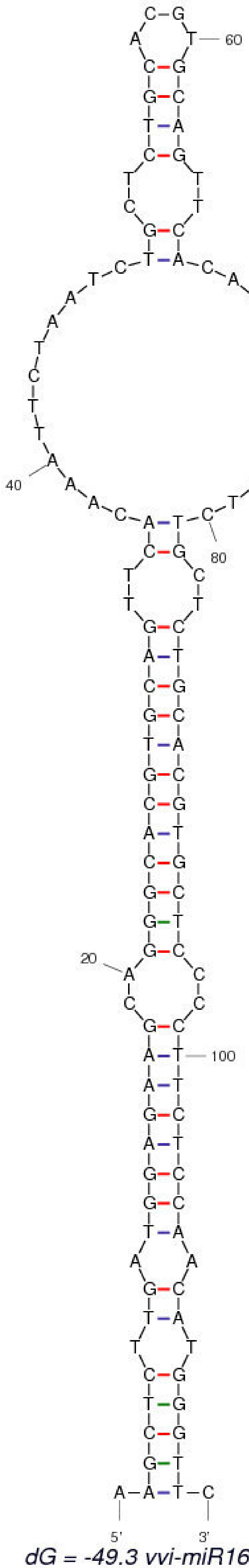

dG = -49.3 vvi-miR164d

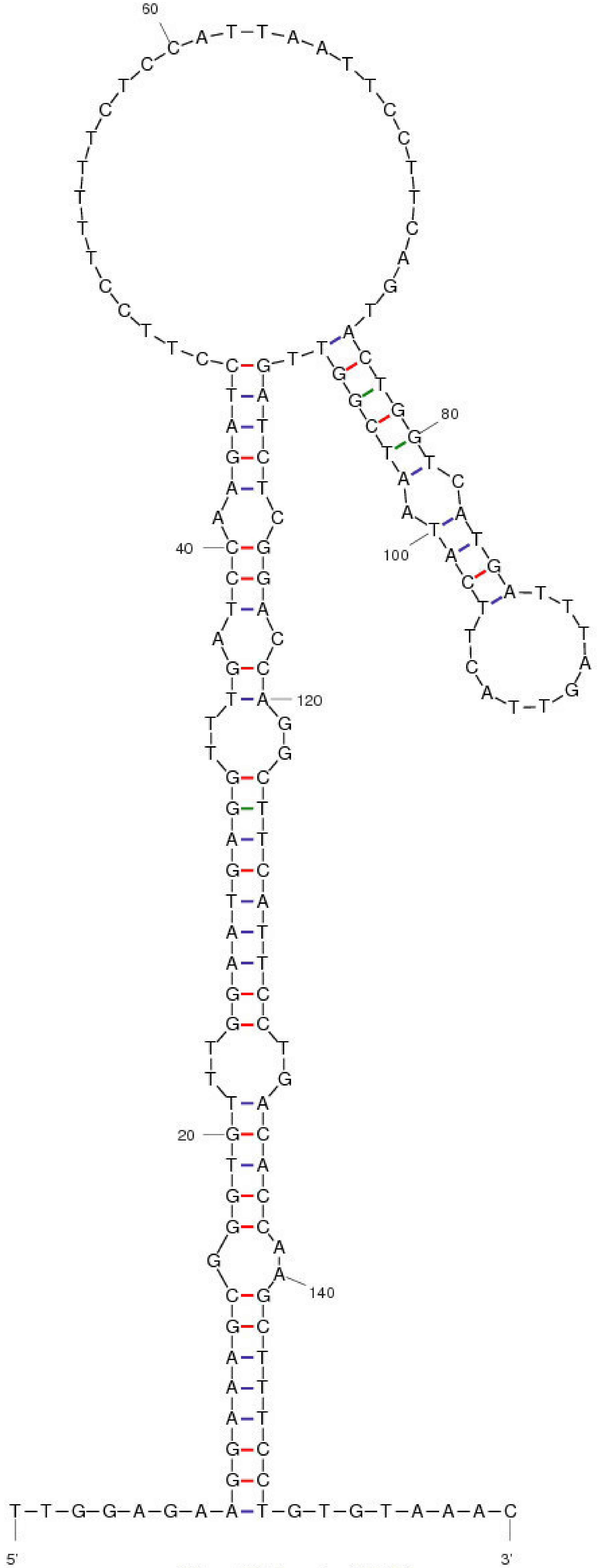

dG = -54.3 vvi-miR166a

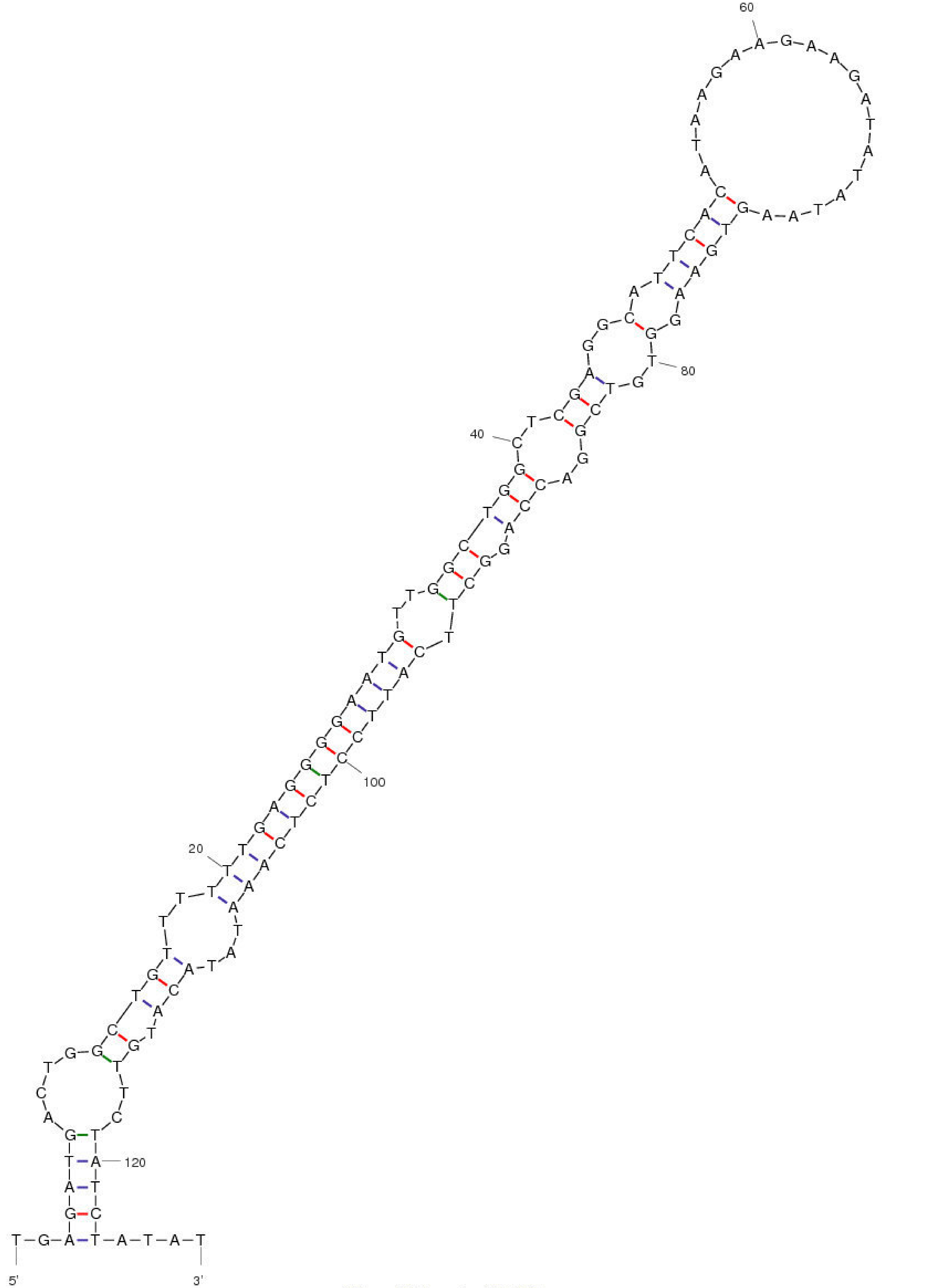

dG = -42.4 vvi-miR166b

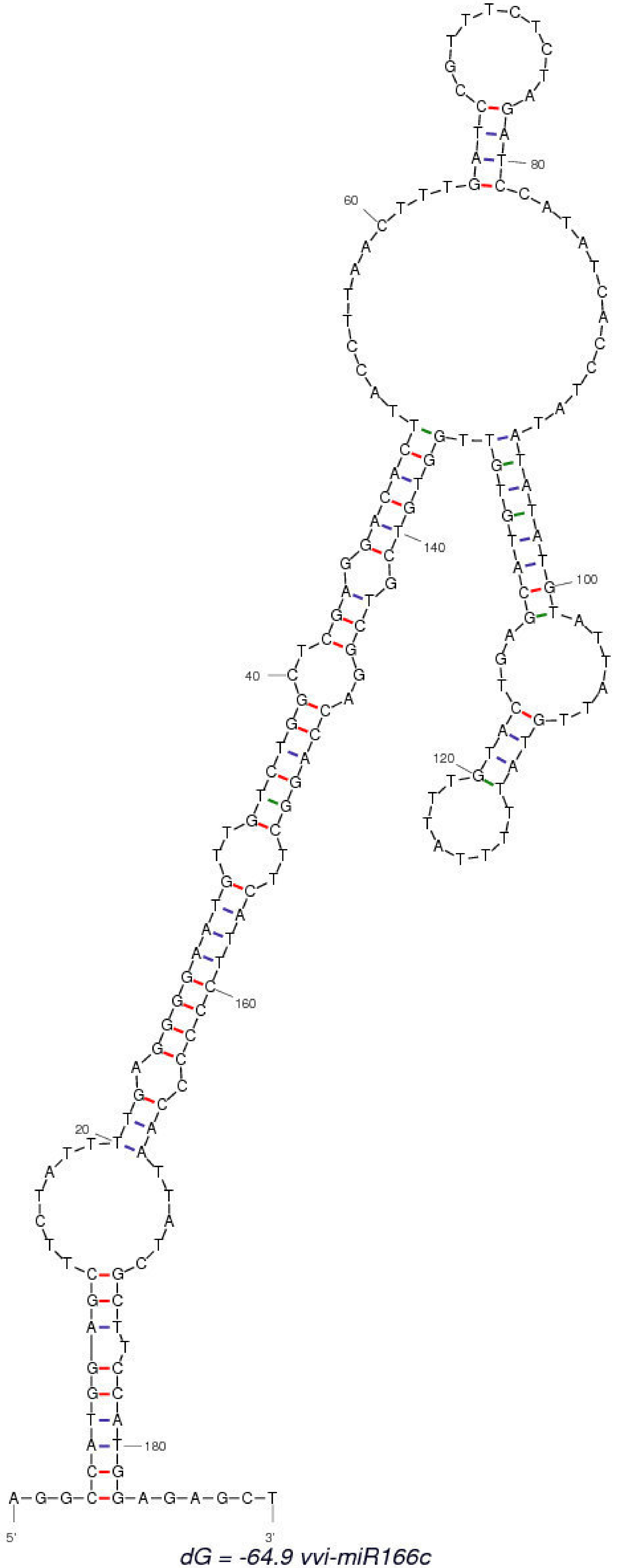

dG = -64.9 vvi-miR166c

Output of sir_graph (©)
mfold_util 4.7

Created Tue Aug 30 07:57:34 2016

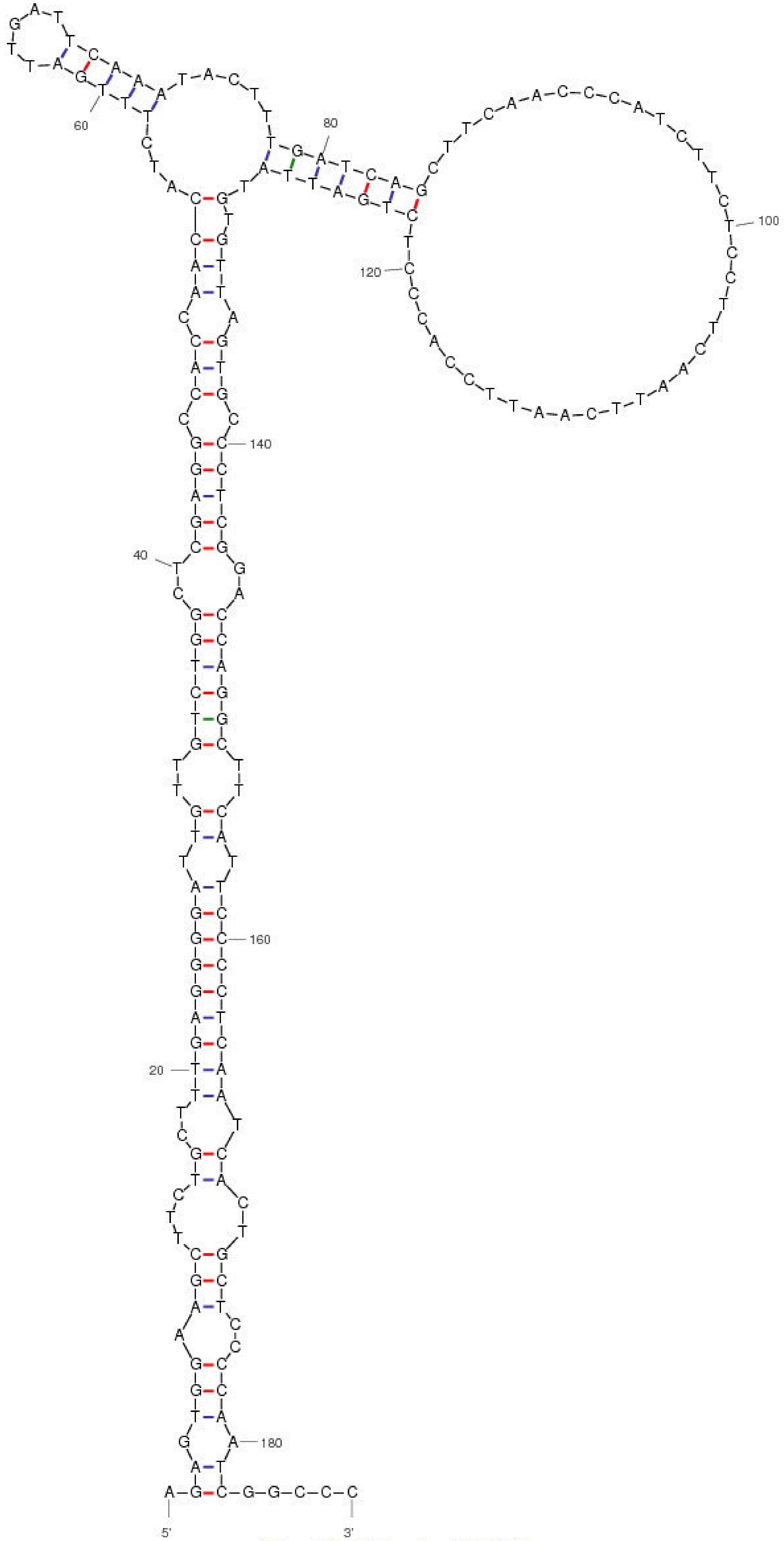

dG = -59.274 vvi-miR166d

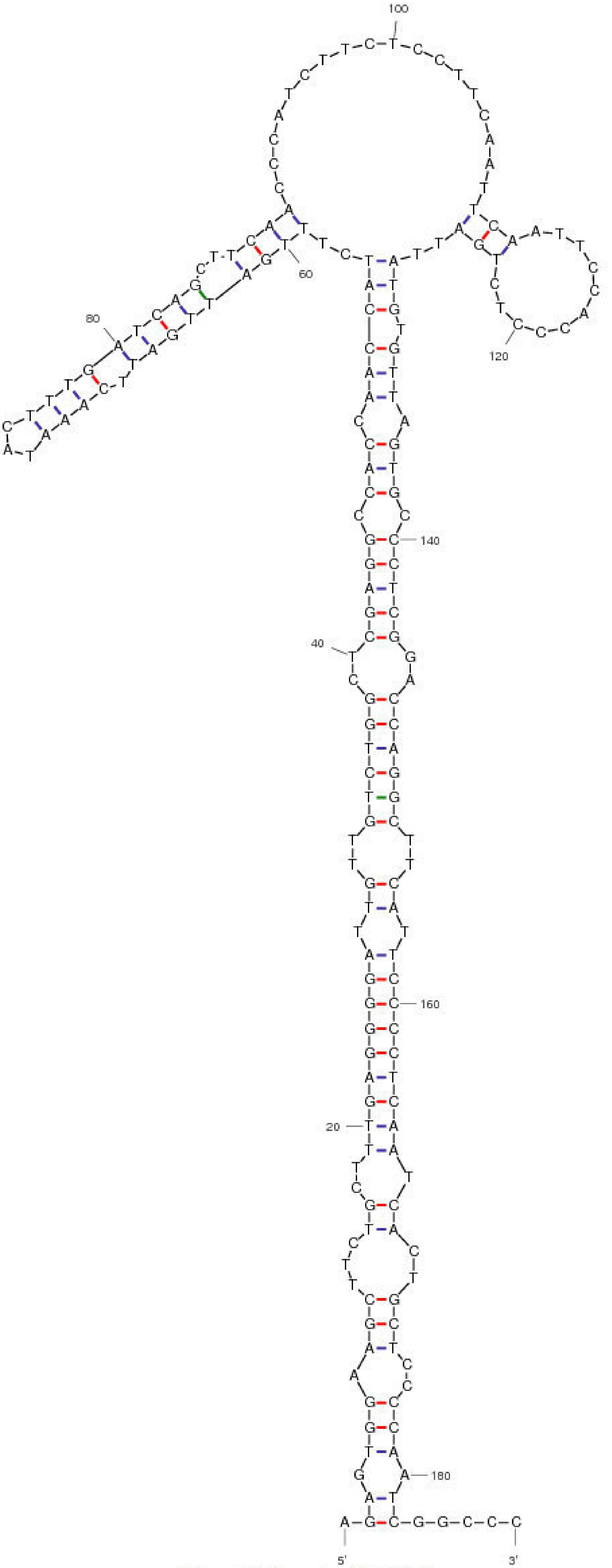

dG = -58.8 vvi-miR166d

Output of sir_graph (©)
mfold_util 4.7

Created Tue Aug 30 07:57:34 2016

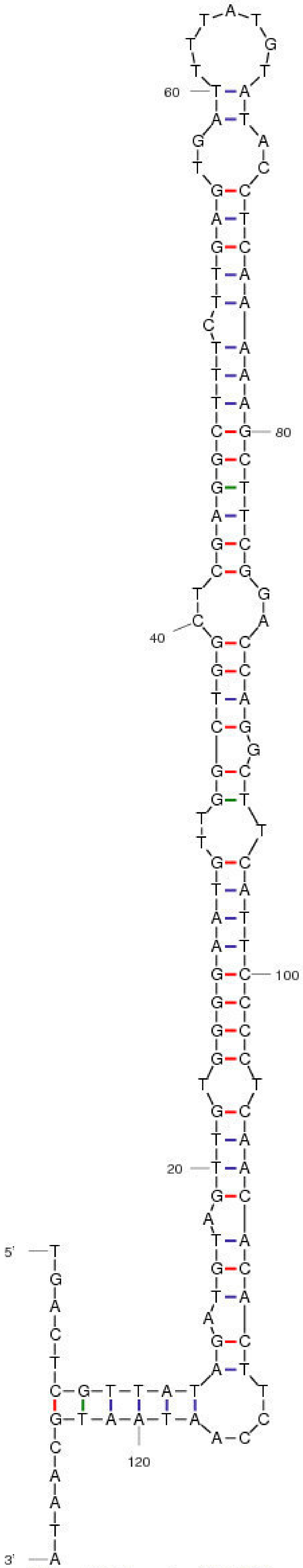

dG = -50.2 vvi-miR166f

Output of sir_graph (©)
mfold_util 4.7

Created Tue Aug 30 07:57:34 2016

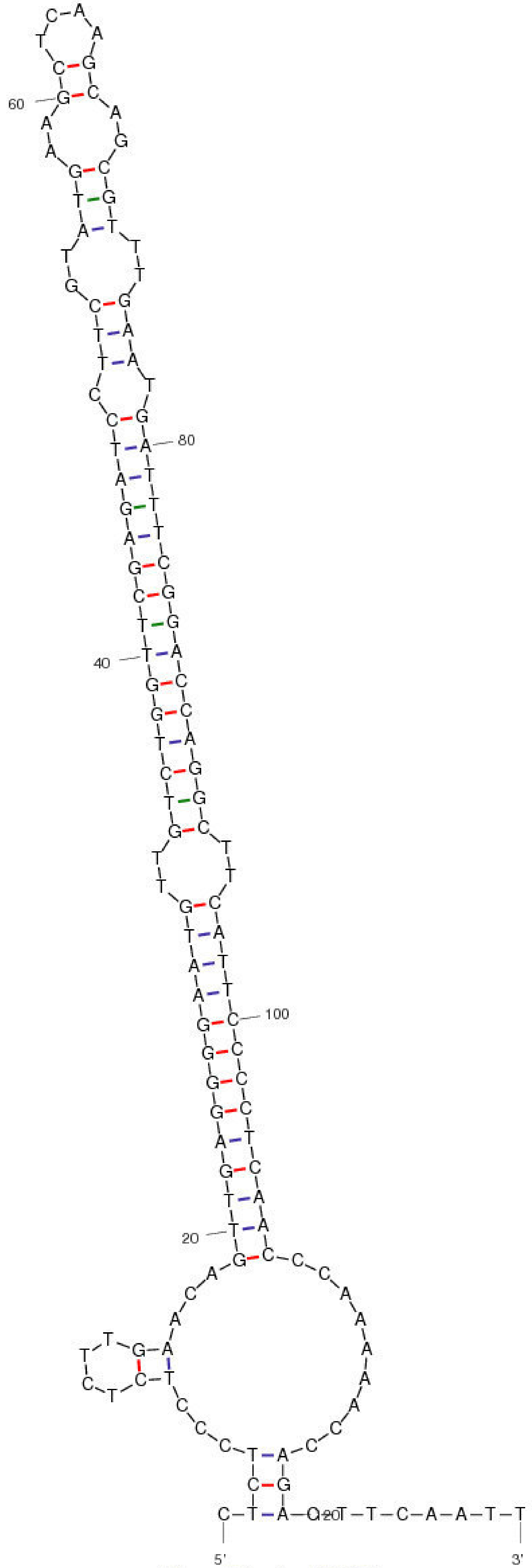

dG = -55 vvi-miR166g

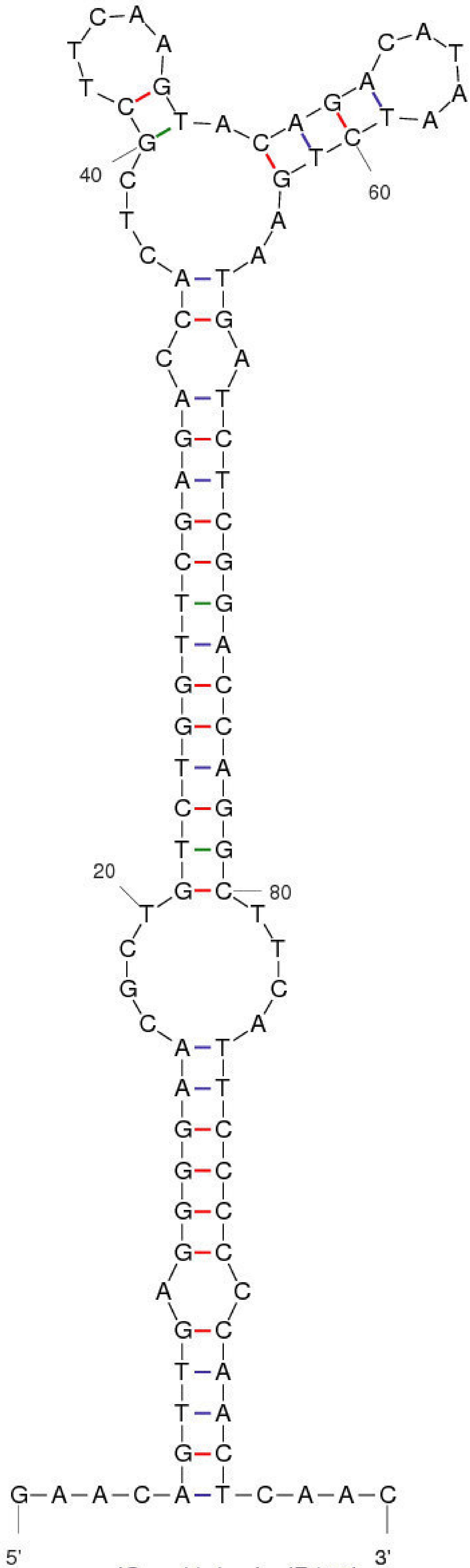

dG = -41.4 vvi-miR166h

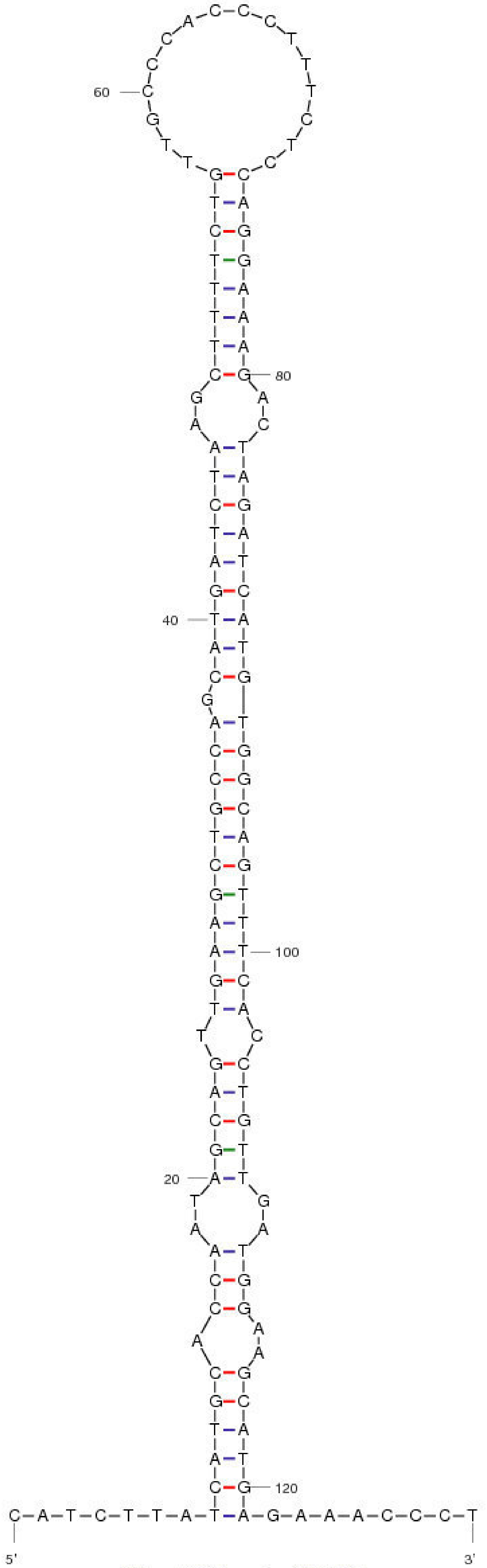

dG = -57.3 vvi-miR167b

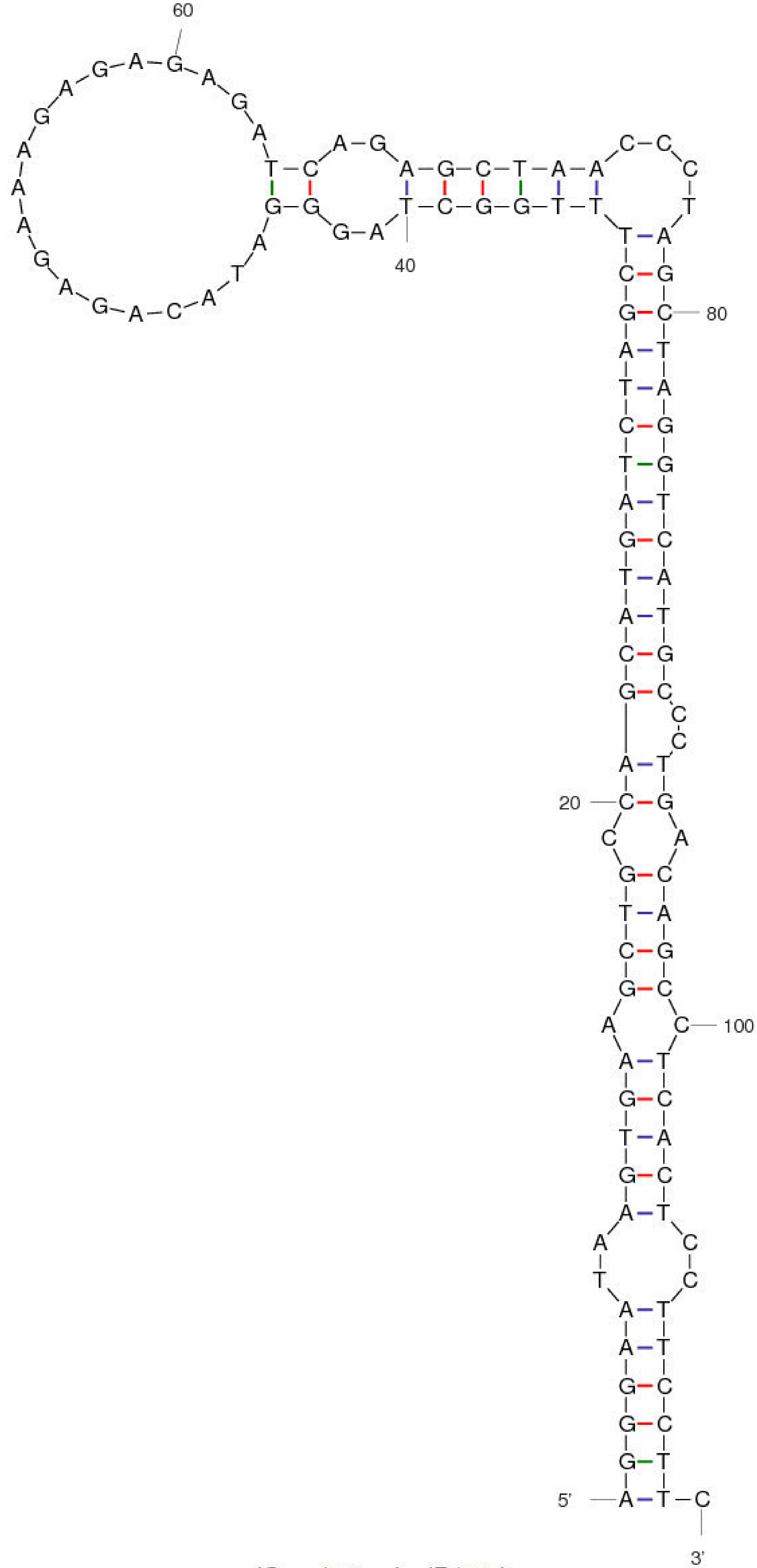

dG = -46.8 vvi-miR167d

Output of sir_graph (©)
mfold_util 4.7

Created Tue Aug 30 07:59:39 2016

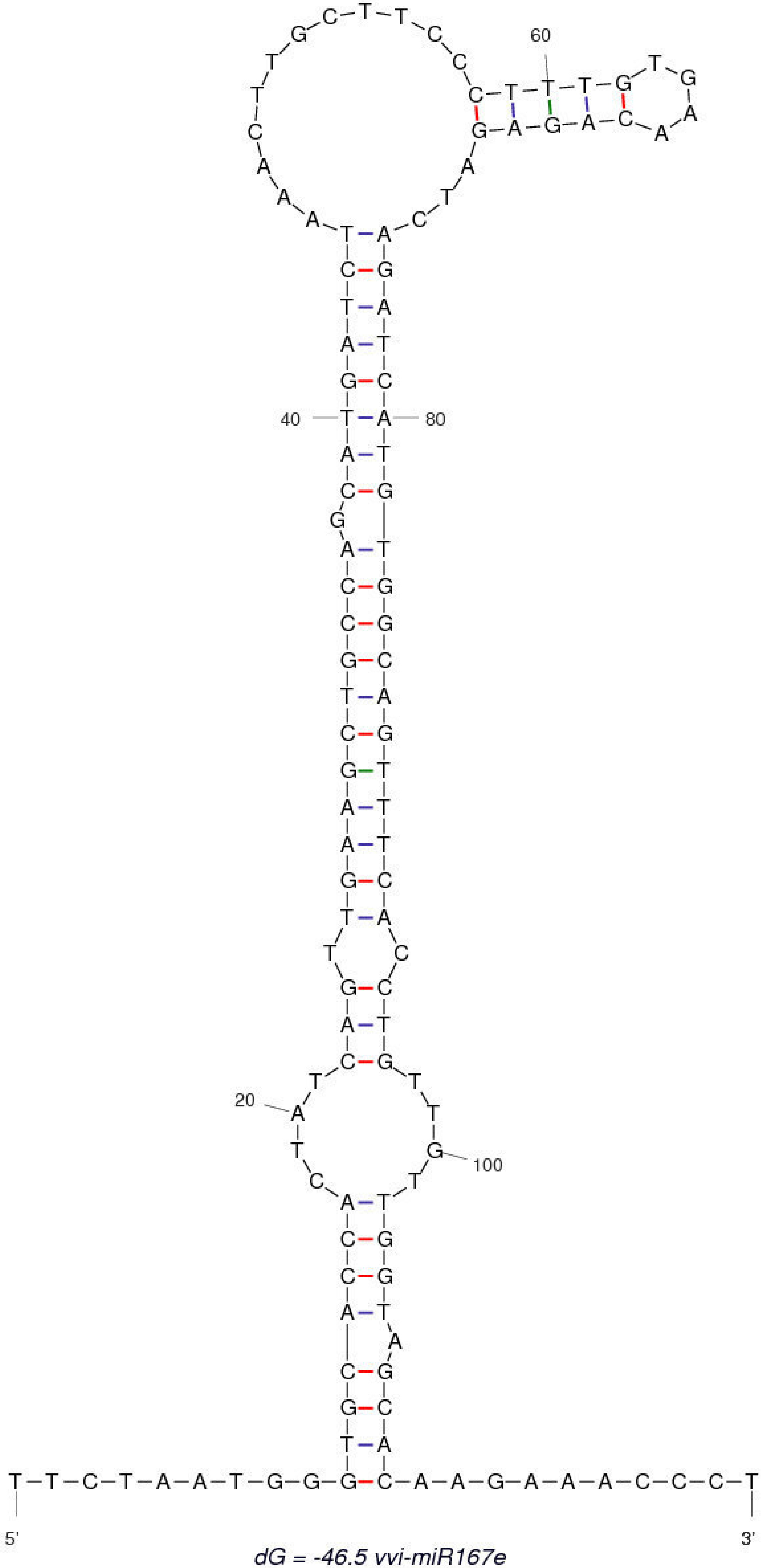

dG = -46.5 vvi-miR167e

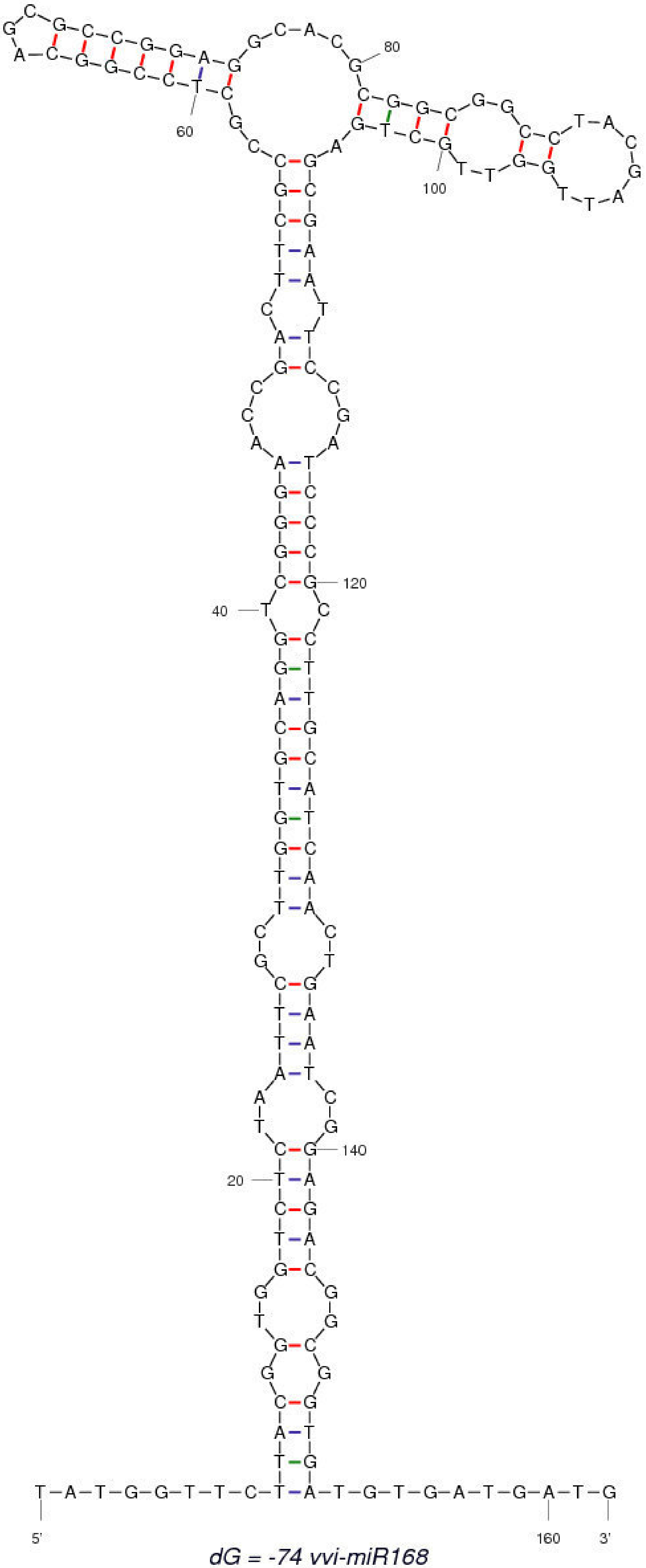

dG = -74 vvi-miR168

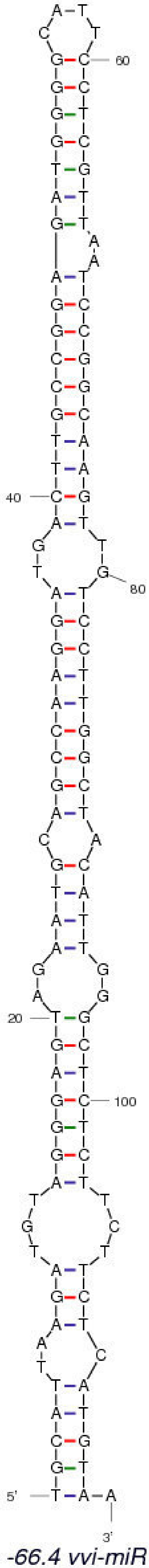

dG = -66.4 vvi-miR169c

Output of sir_graph (©)
mfold_util 4.7

Created Tue Aug 30 07:59:40 2016

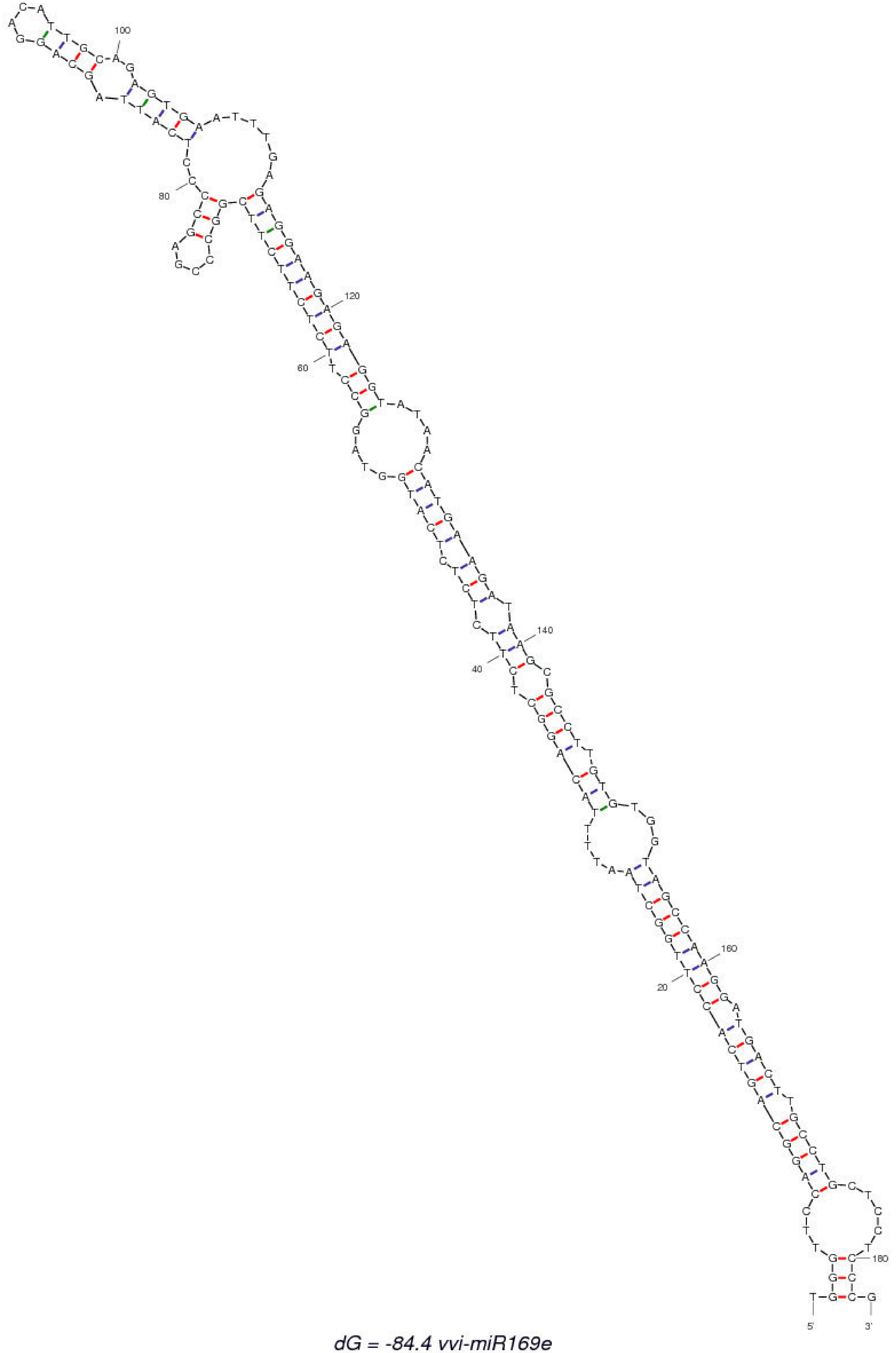

dG = -84.4 vvi-miR169e

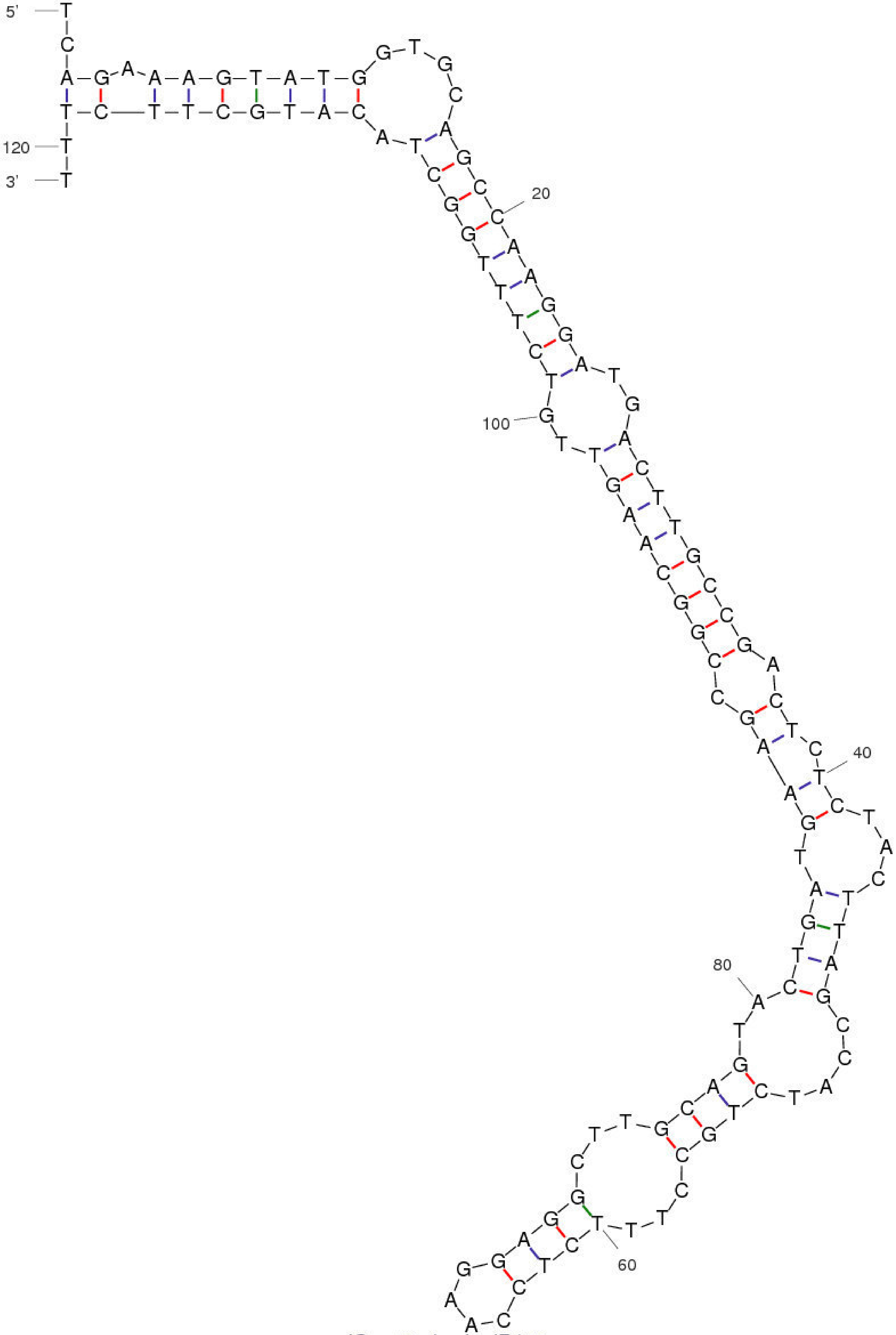

dG = -50.4 vvi-miR169g

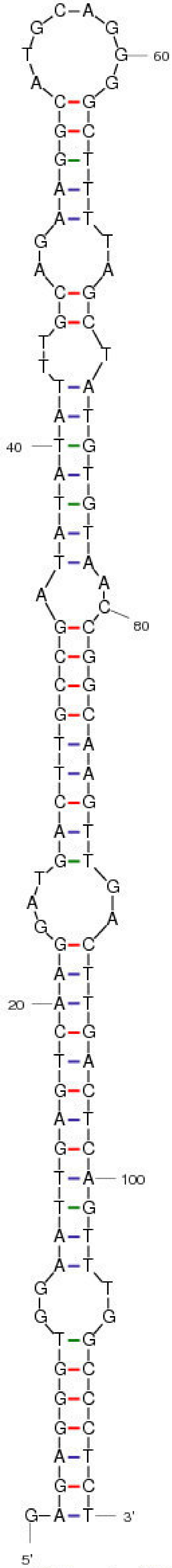

dG = -58.5 vvi-miR169r

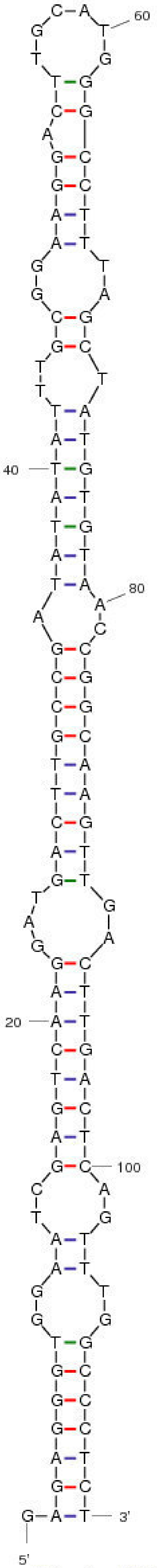

dG = -55 vvi-miR169t

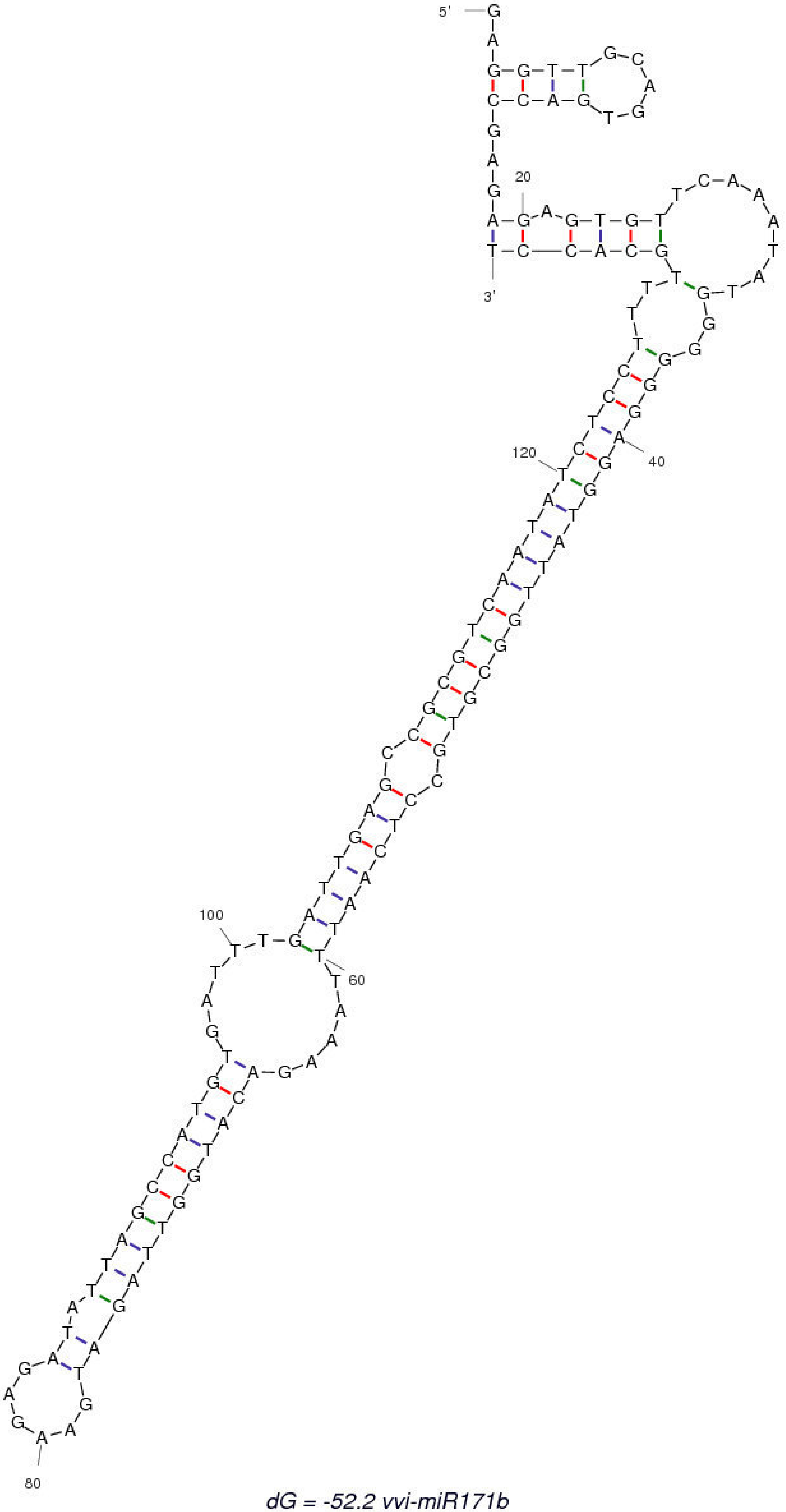

dG = -52.2 vvi-miR171b

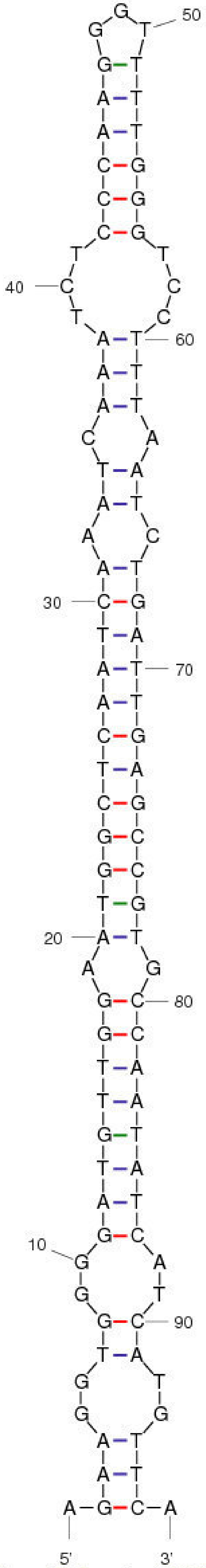

dG = -42.2 vvi-miR171i

Output of sir_graph (©)
mfold_util 4.7

Created Tue Aug 30 07:59:40 2016

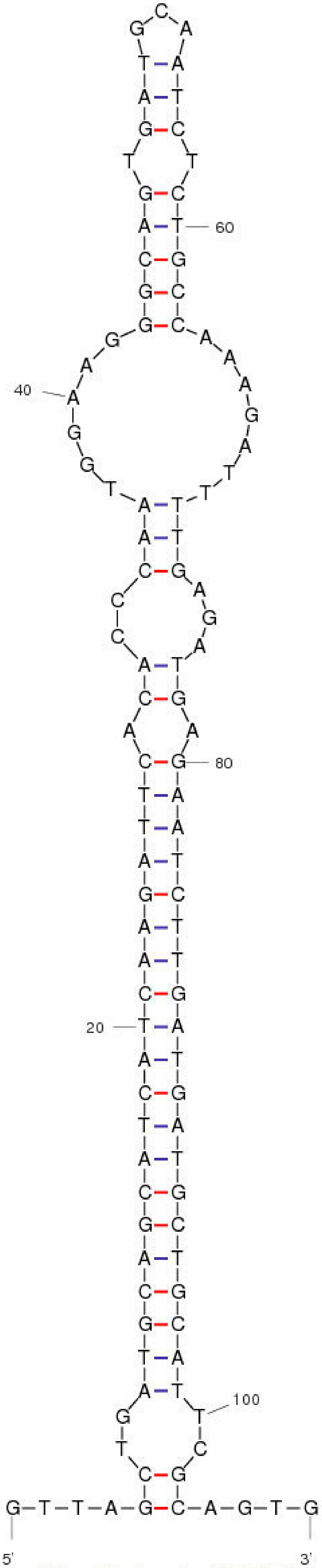

dG = -50.1 vvi-miR172d

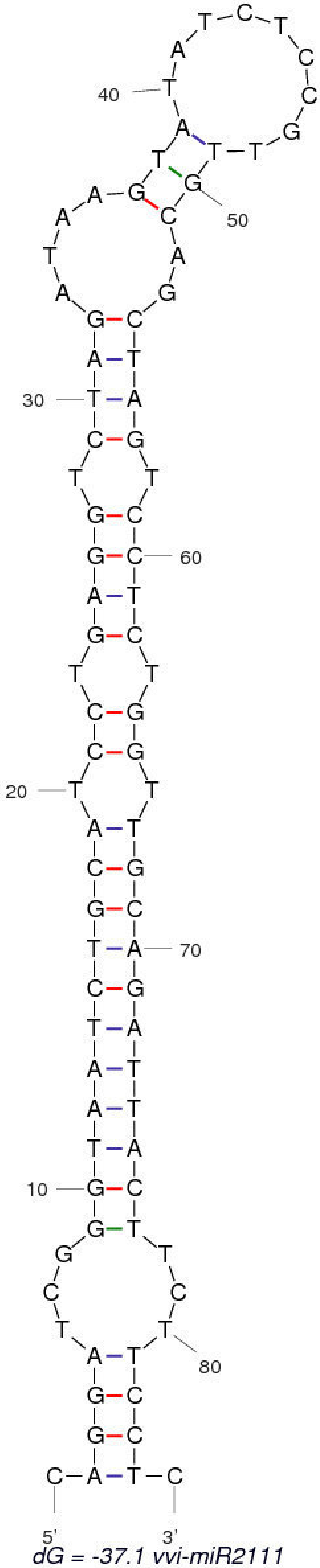

dG = -37.1 vvi-miR2111

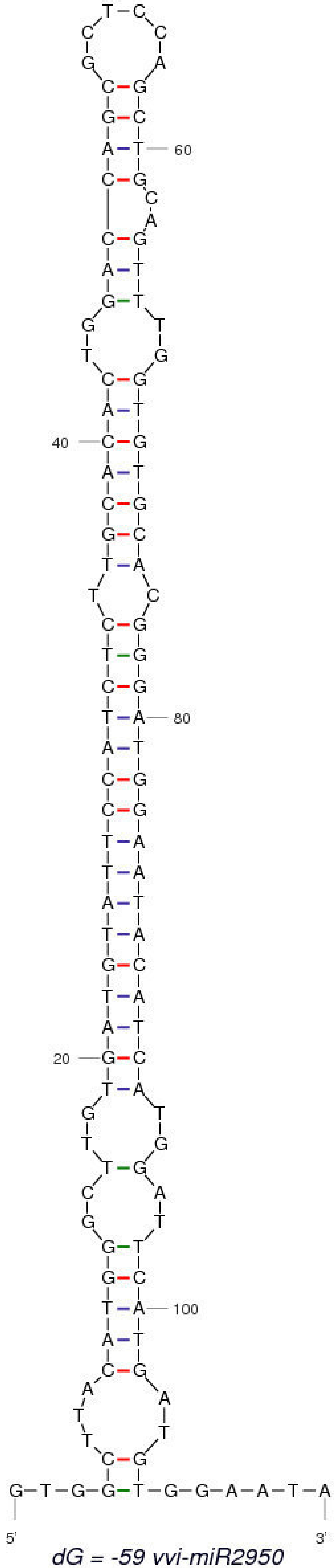

dG = -59 vvi-miR2950

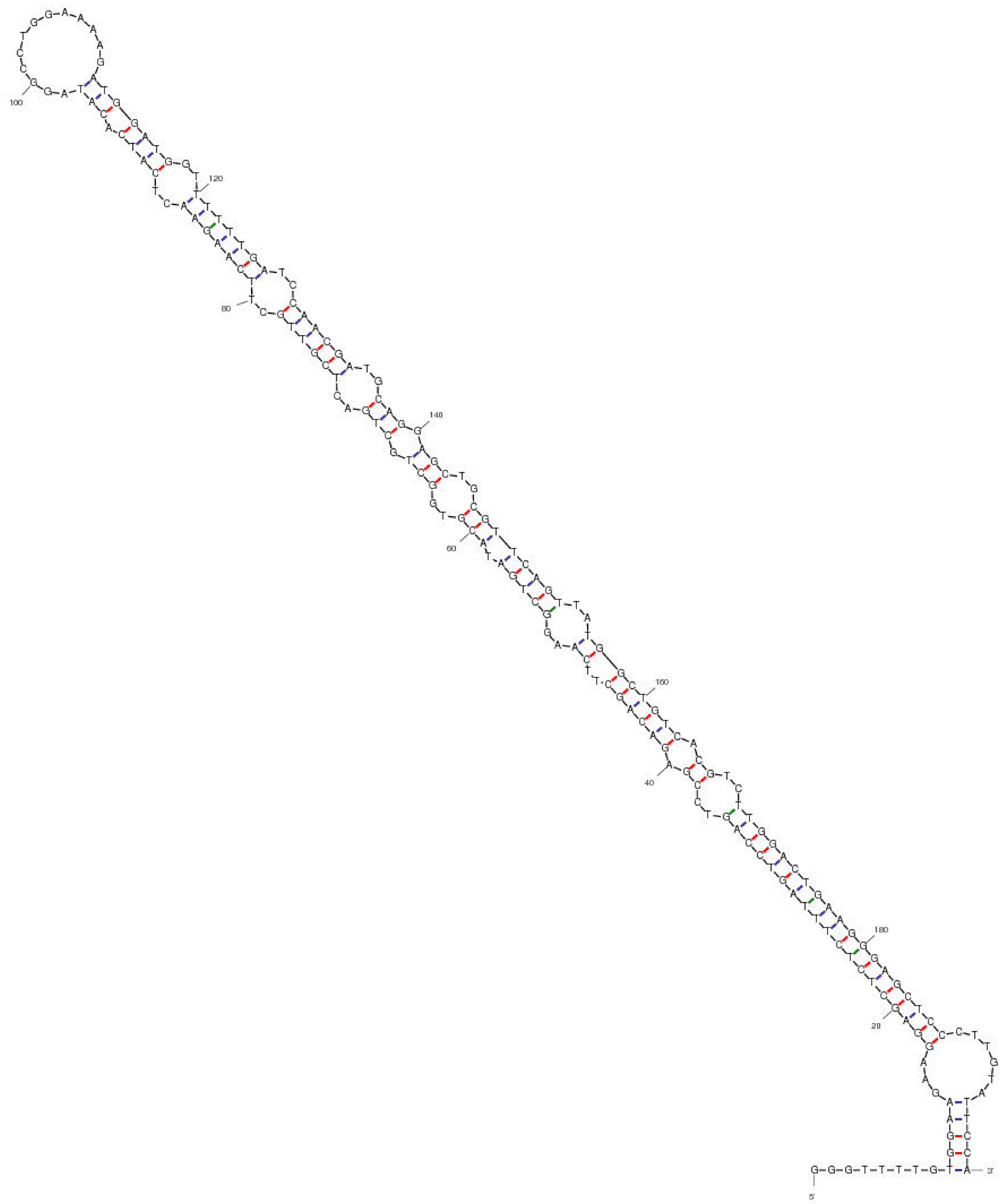

dG = -98 vvi-miR319b

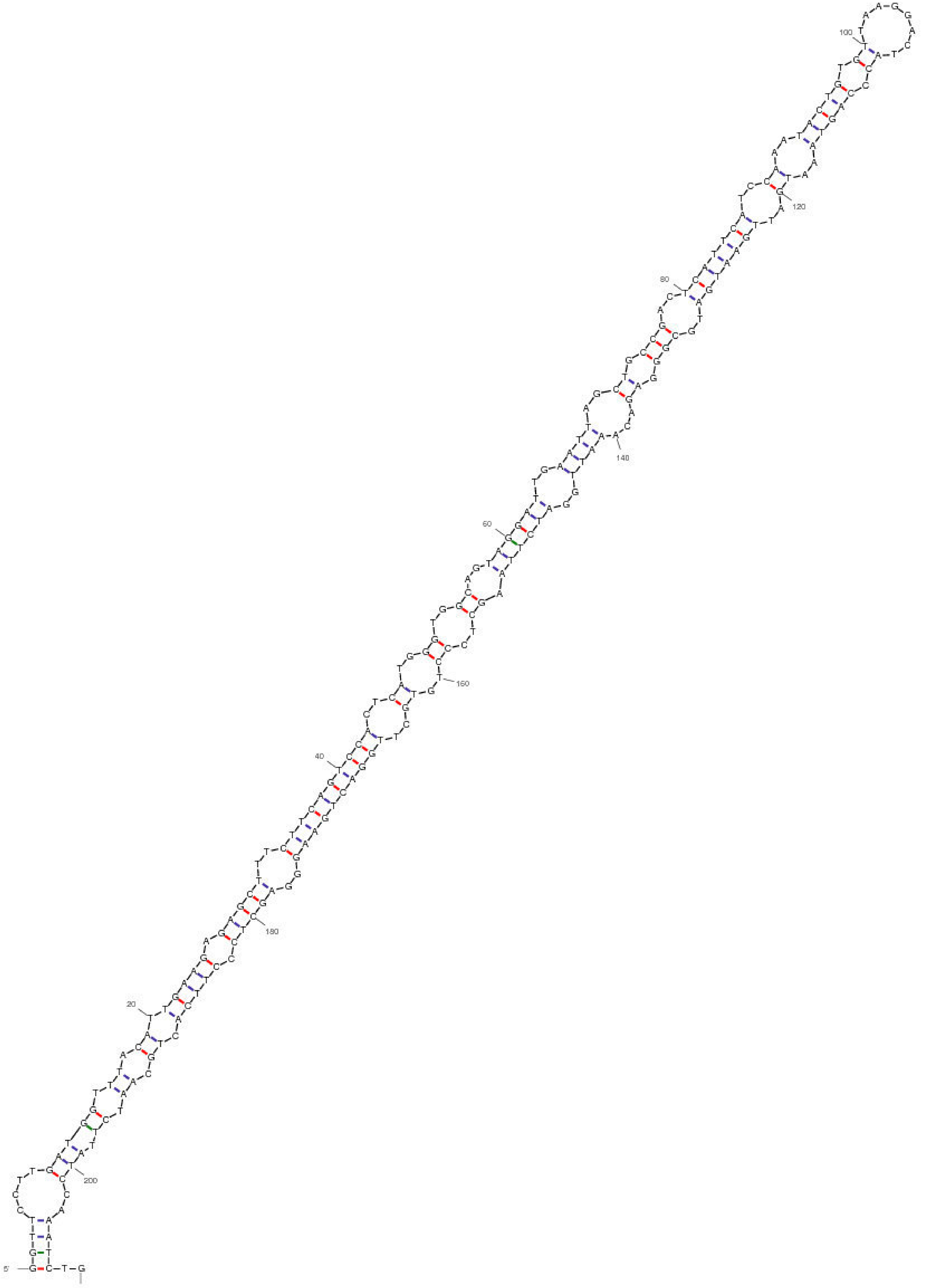

dG = -82.9 vvi-miR319c

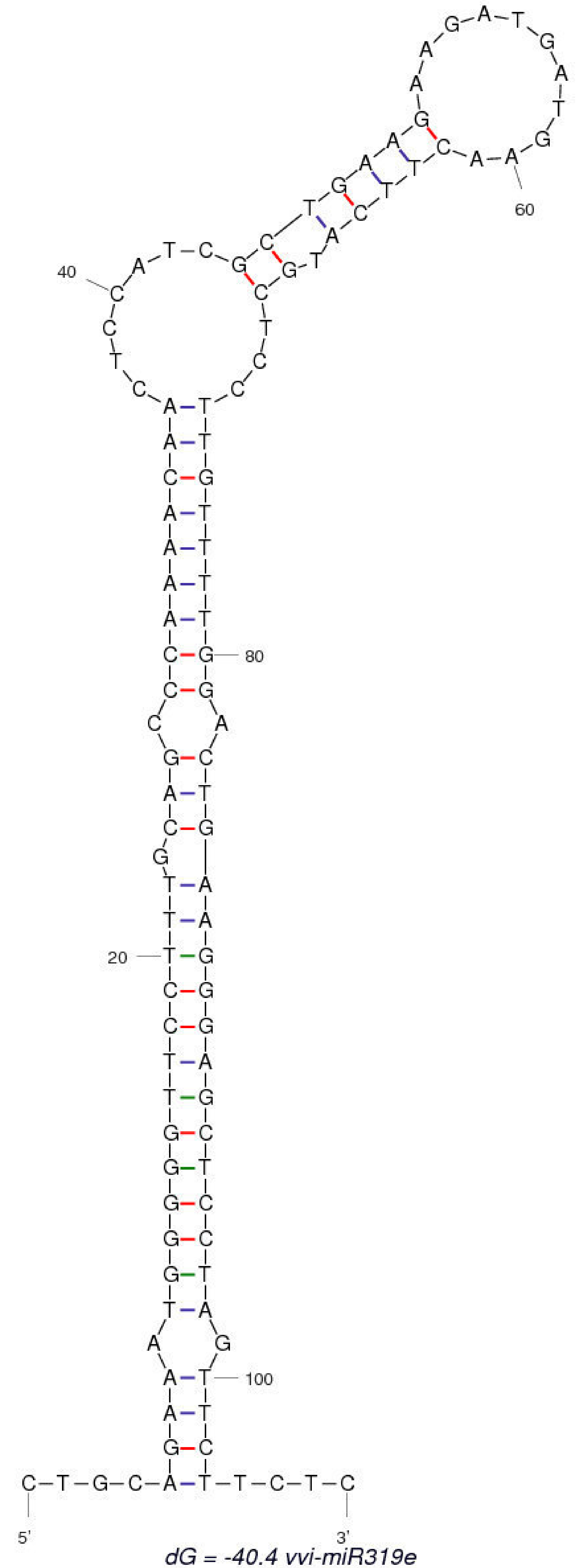

dG = -40.4 vvi-miR319e

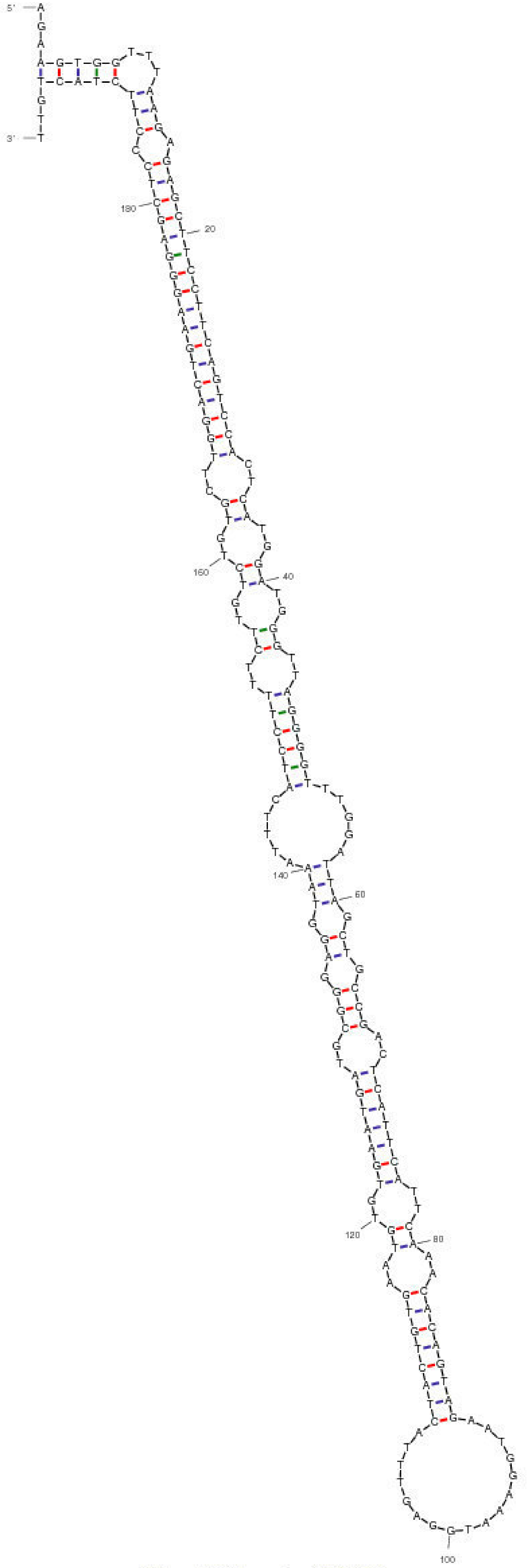

dG = -87.2 vvi-miR319f

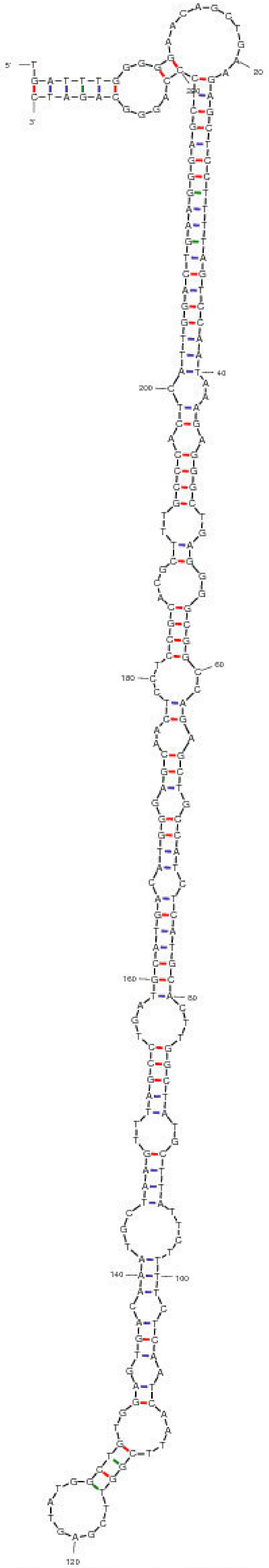

dG = -92.1 vvi-miR319g

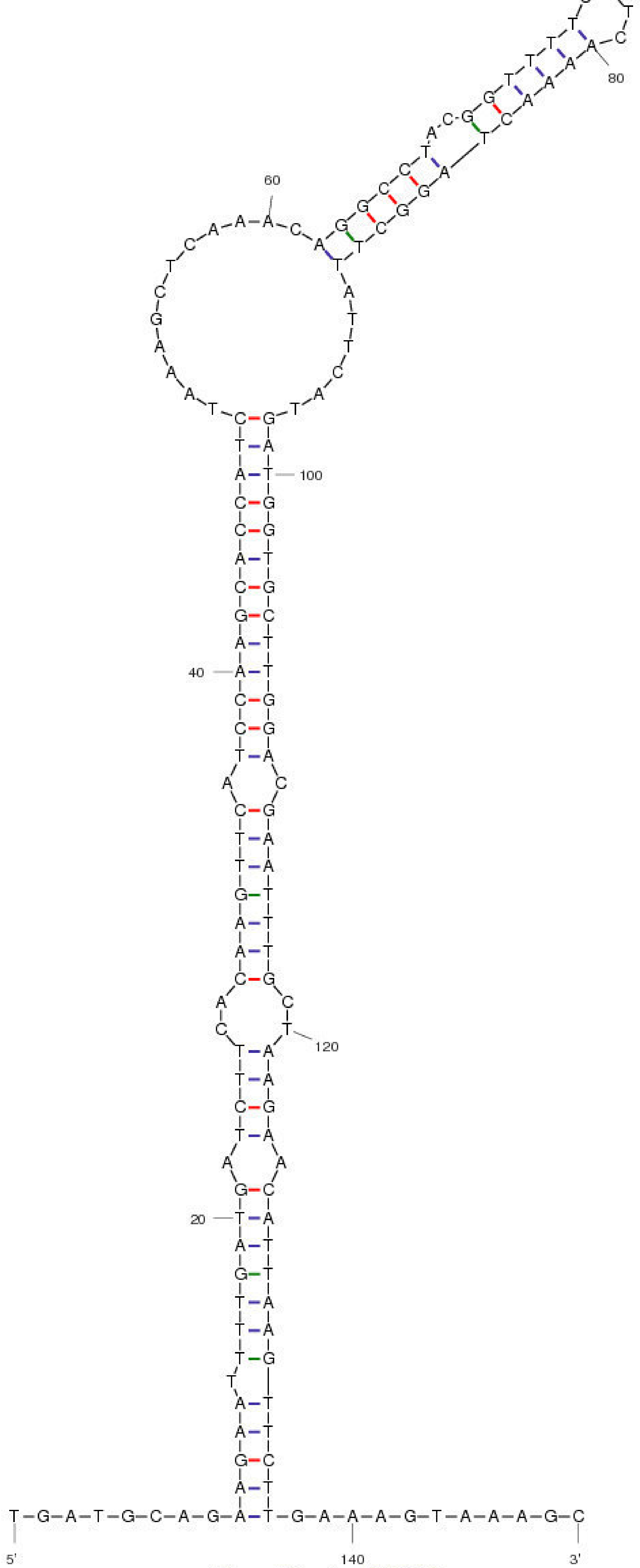

dG = -52 vvi-miR3623

Output of sir_graph (©)
mfold_util 4.7

Created Tue Aug 30 07:59:42 2016

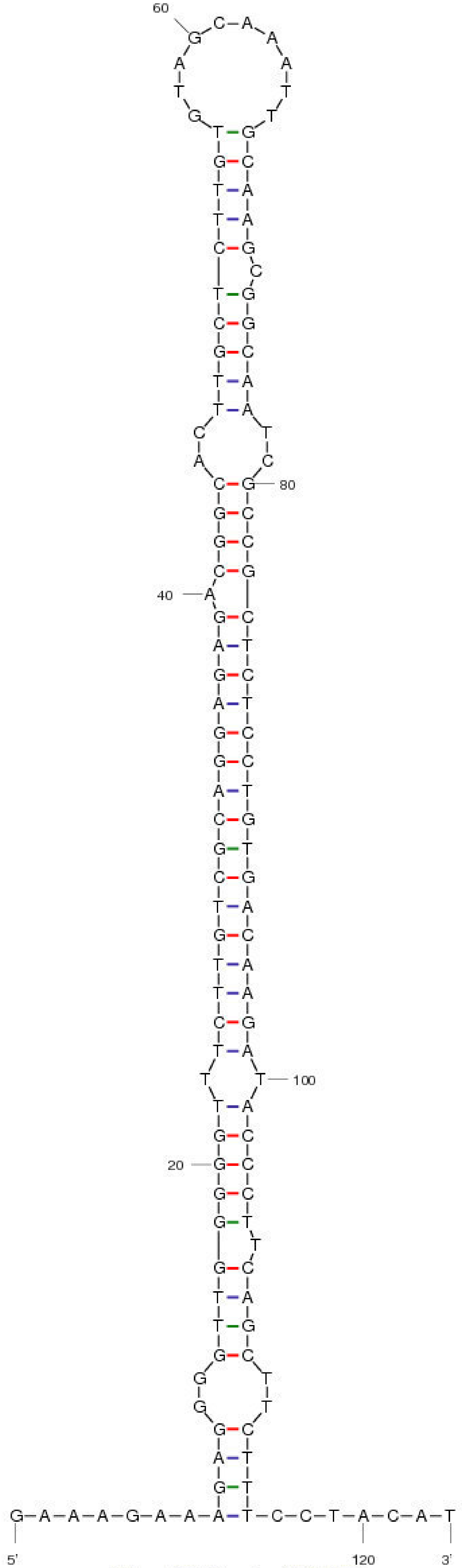

dG = -67.7 vvi-miR3627

Output of sir_graph (©)
mfold_util 4.7

Created Tue Aug 30 07:59:42 2016

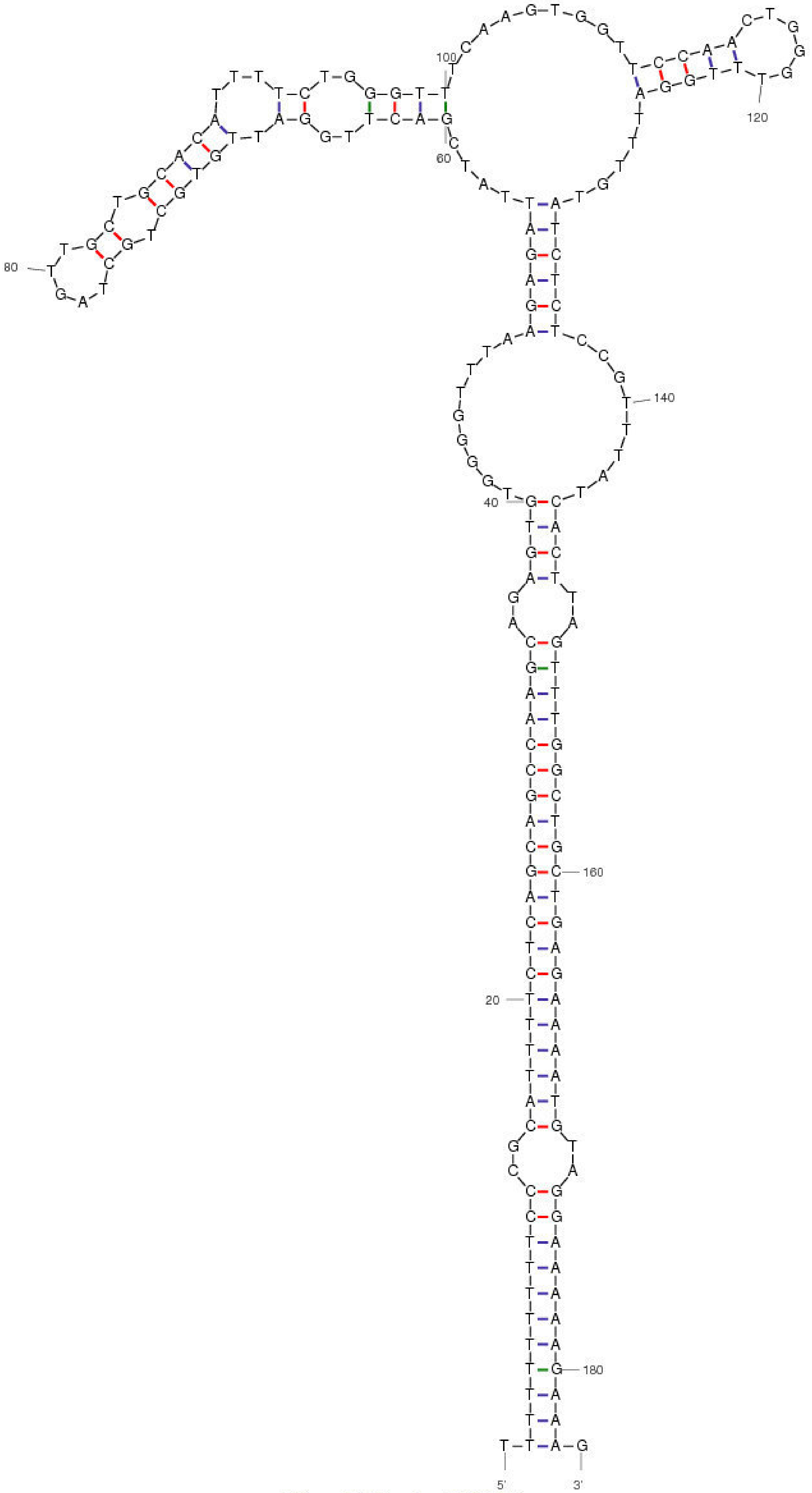

dG = -81.2 vvi-miR3629a

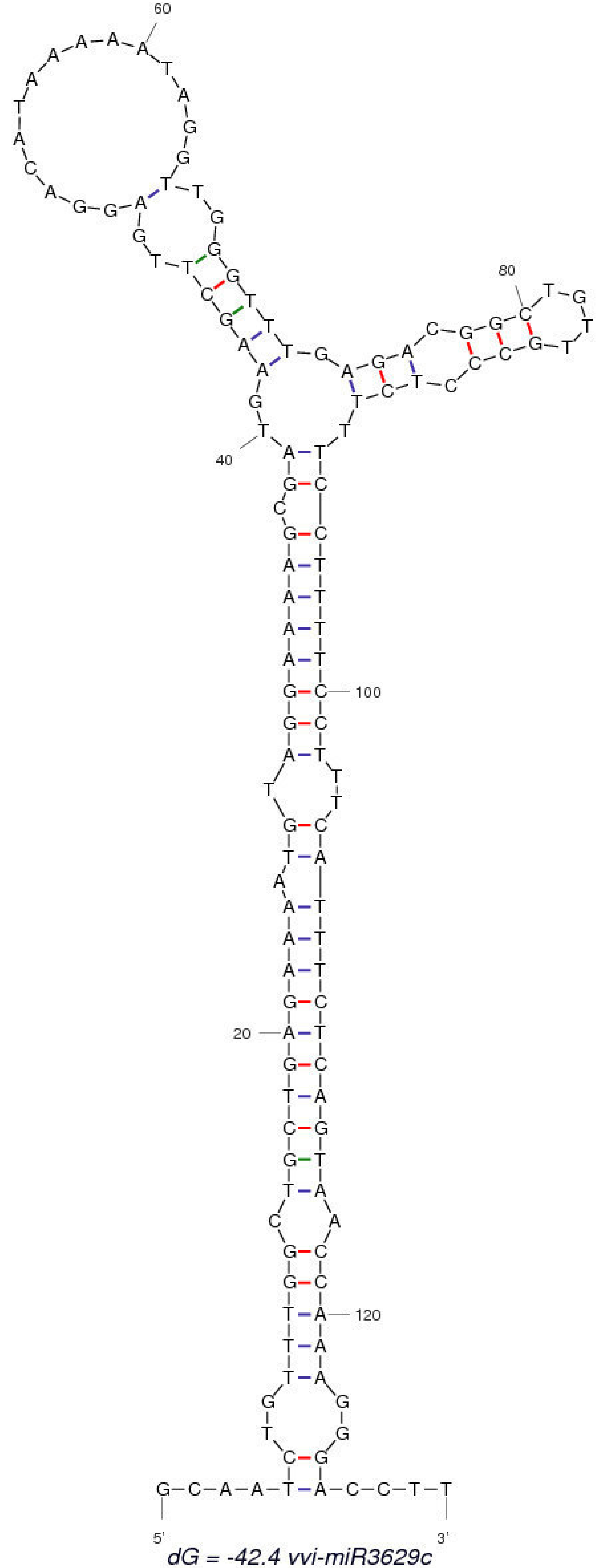

dG = -42.4 vvi-miR3629c

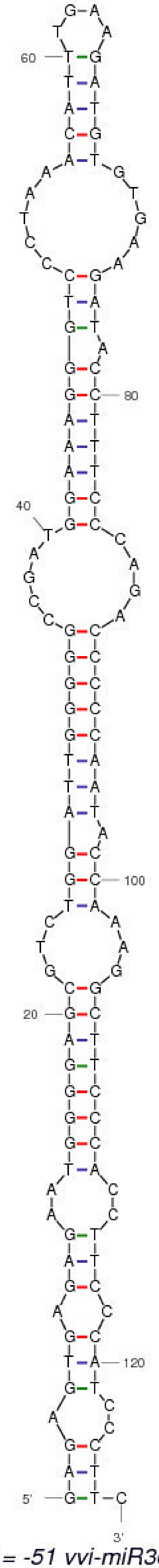

dG = -51 vvi-miR3632

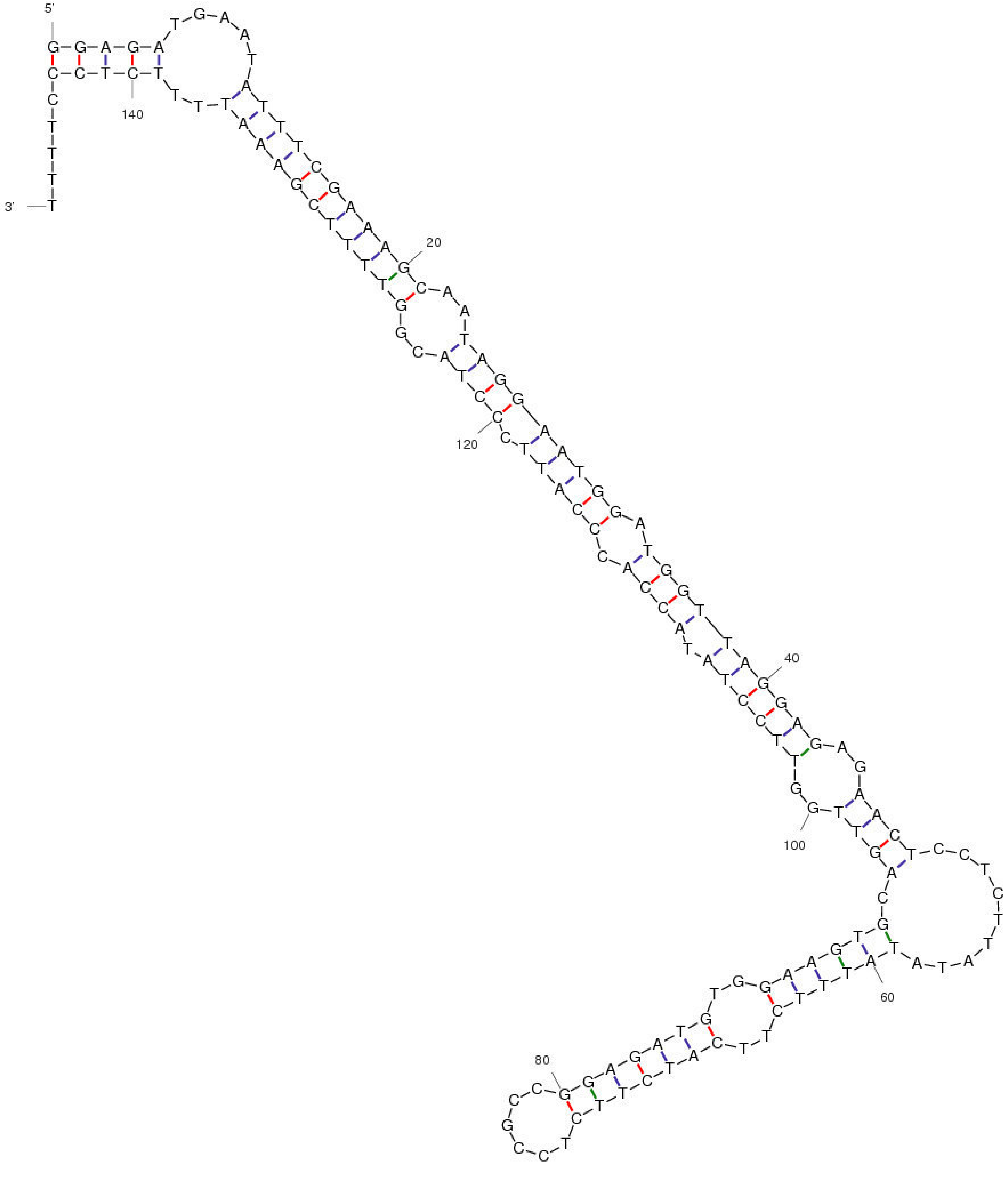

dG = -58.7 vvi-miR3633a

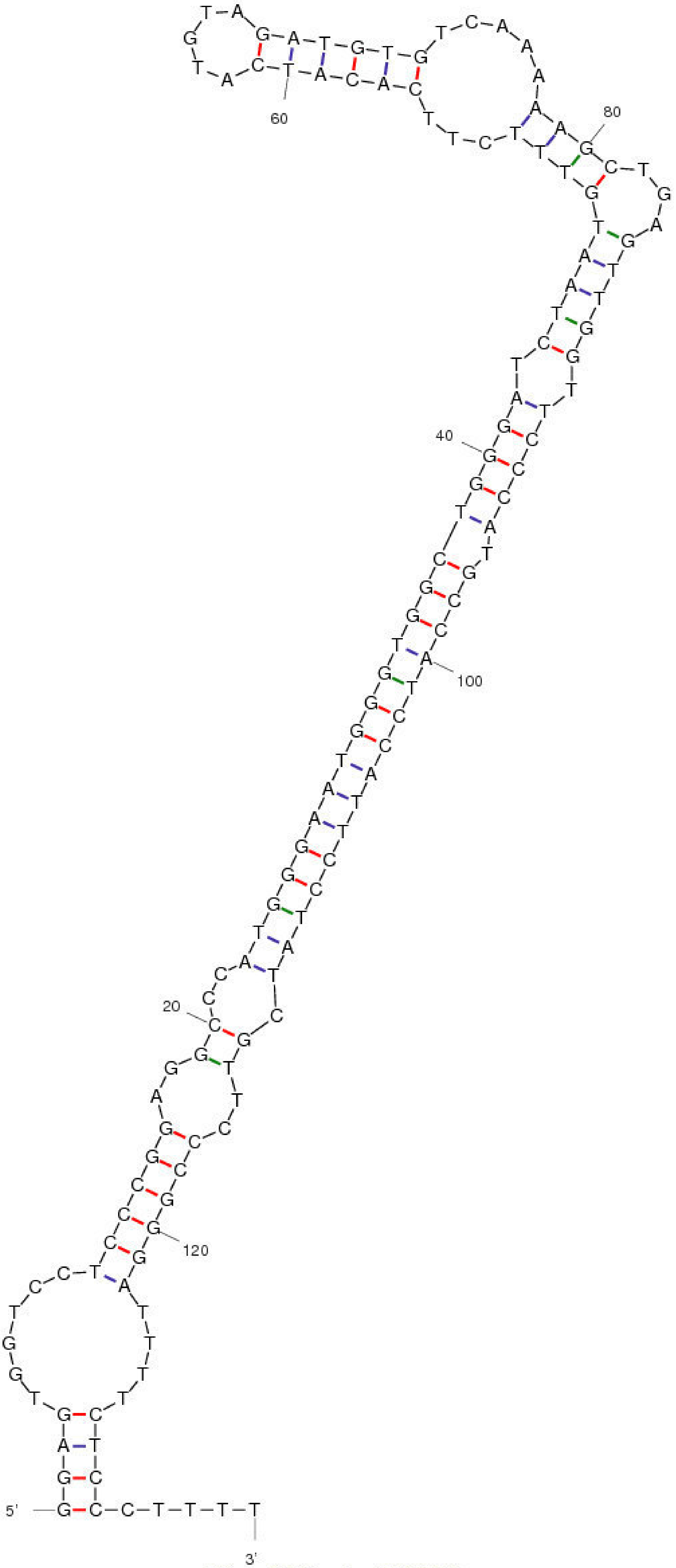

dG = -65.6 vvi-miR3633b

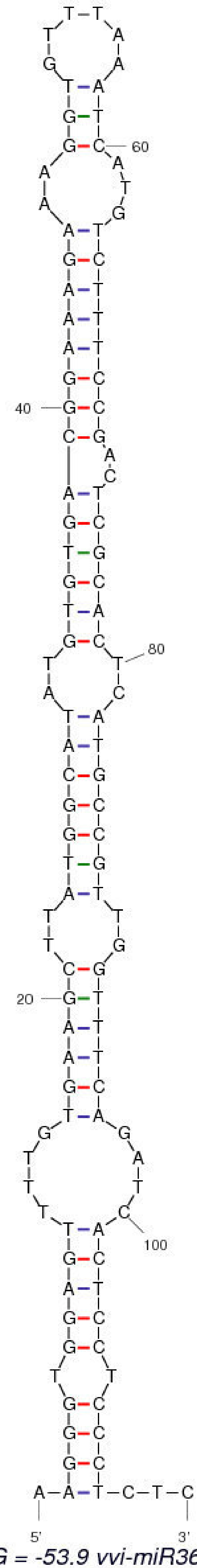

dG = -53.9 vvi-miR3634

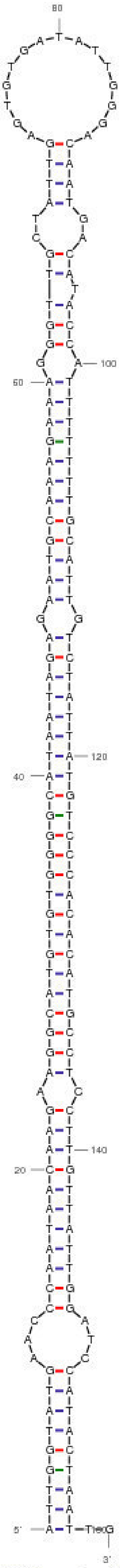

dG = -87.9 vvi-miR3635

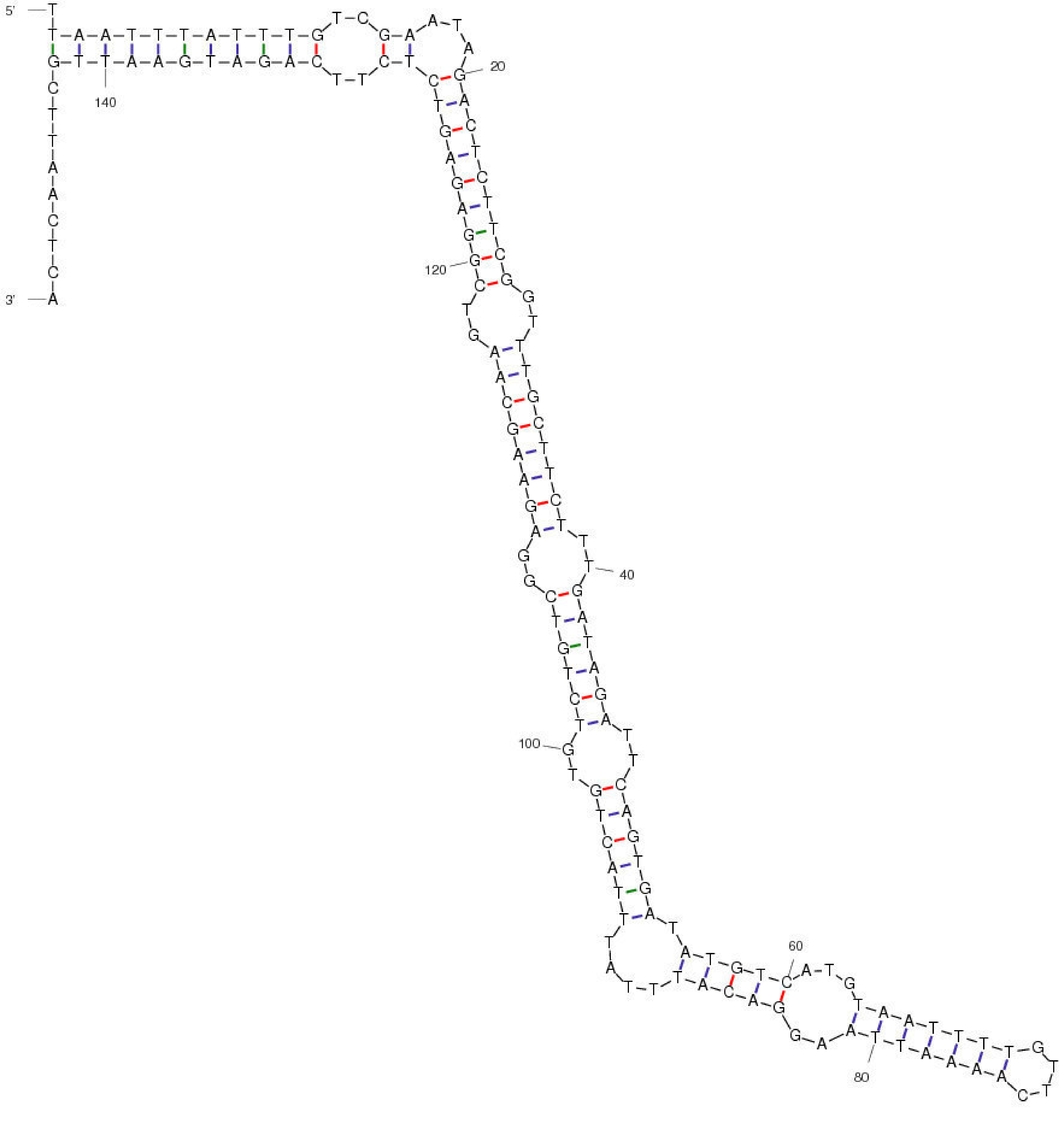

dG = -65.6 vvi-miR3636

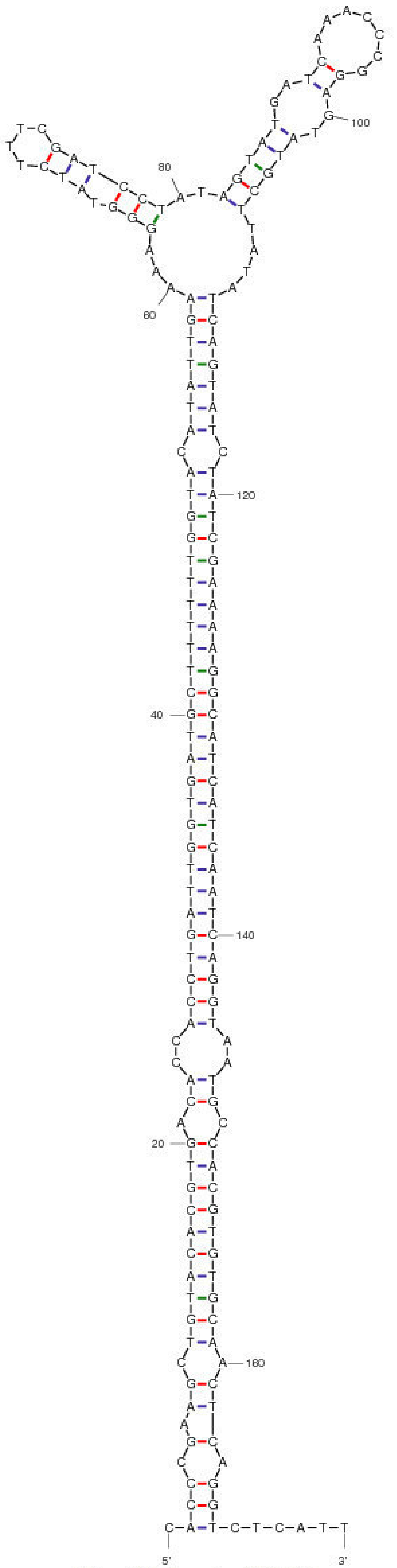

dG = -74.8 vvi-miR3640

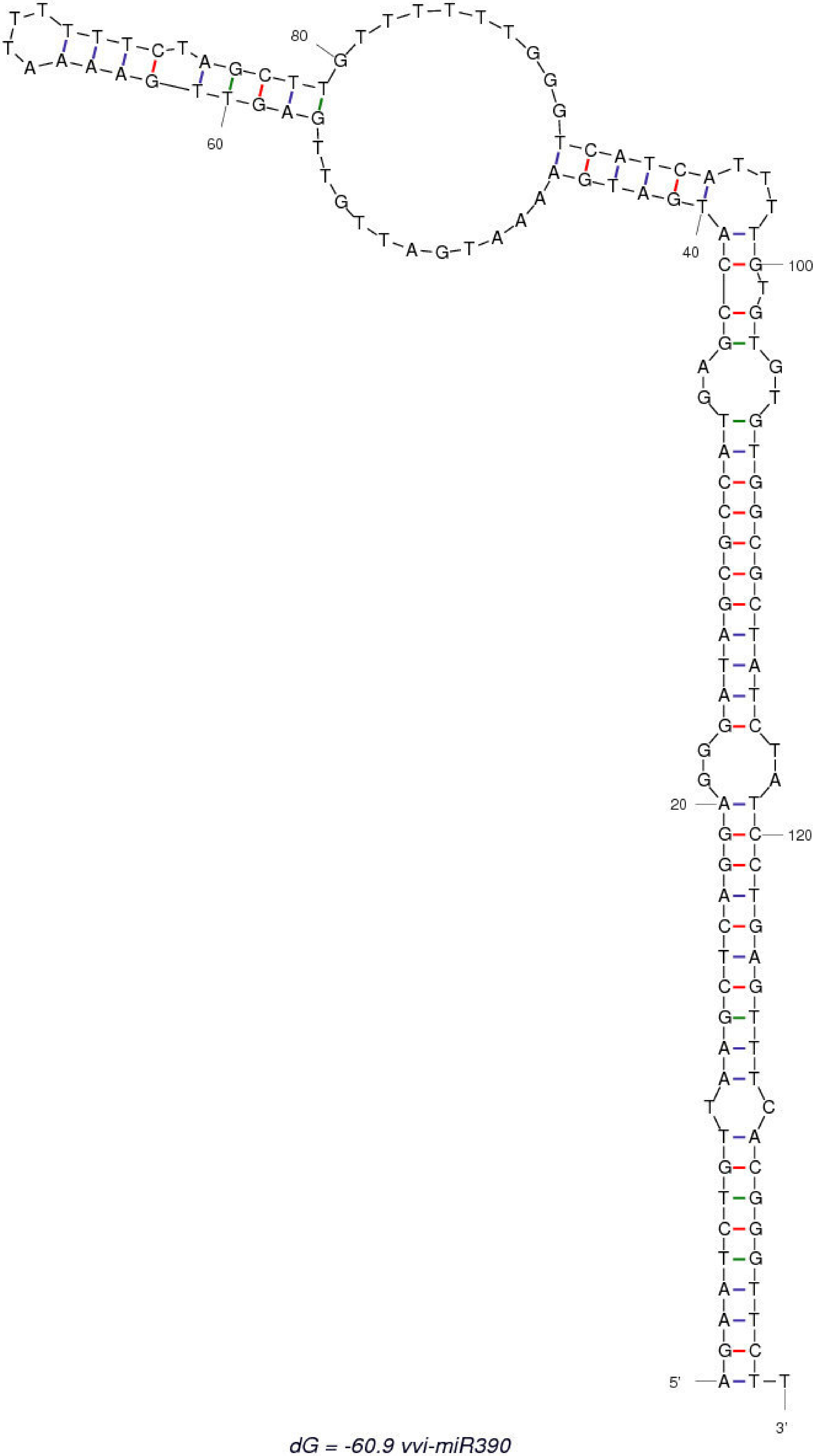

dG = -60.9 vvi-miR390

Output of sir_graph (©)
mfold_util 4.7

Created Tue Aug 30 08:01:42 2016

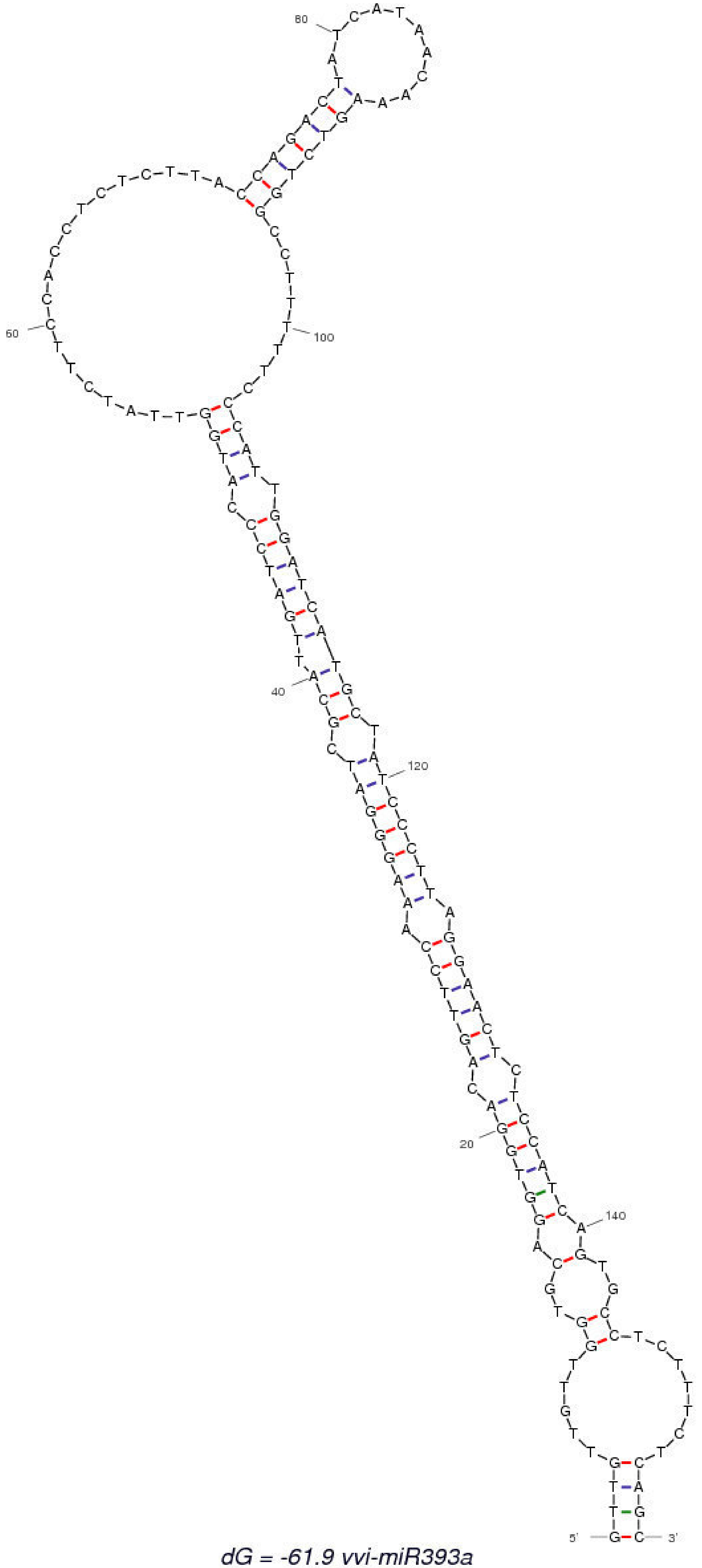

dG = -61.9 vvi-miR393a

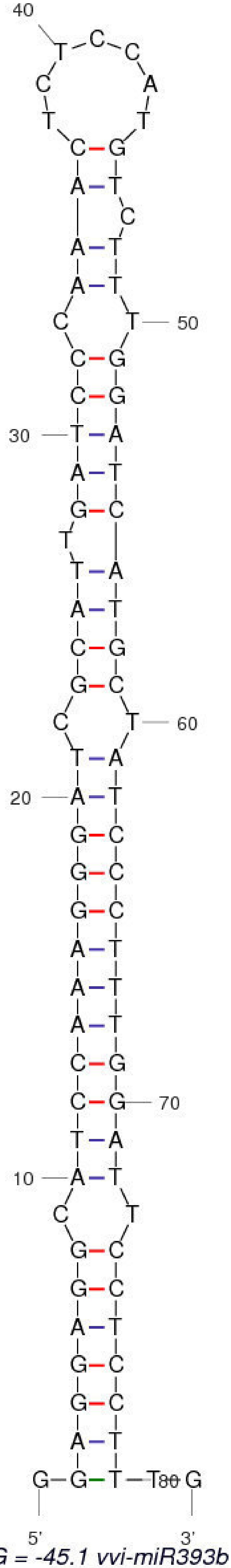

dG = -45.1 vvi-miR393b

Output of sir_graph (©)
mfold_util 4.7

Created Tue Aug 30 08:01:43 2016

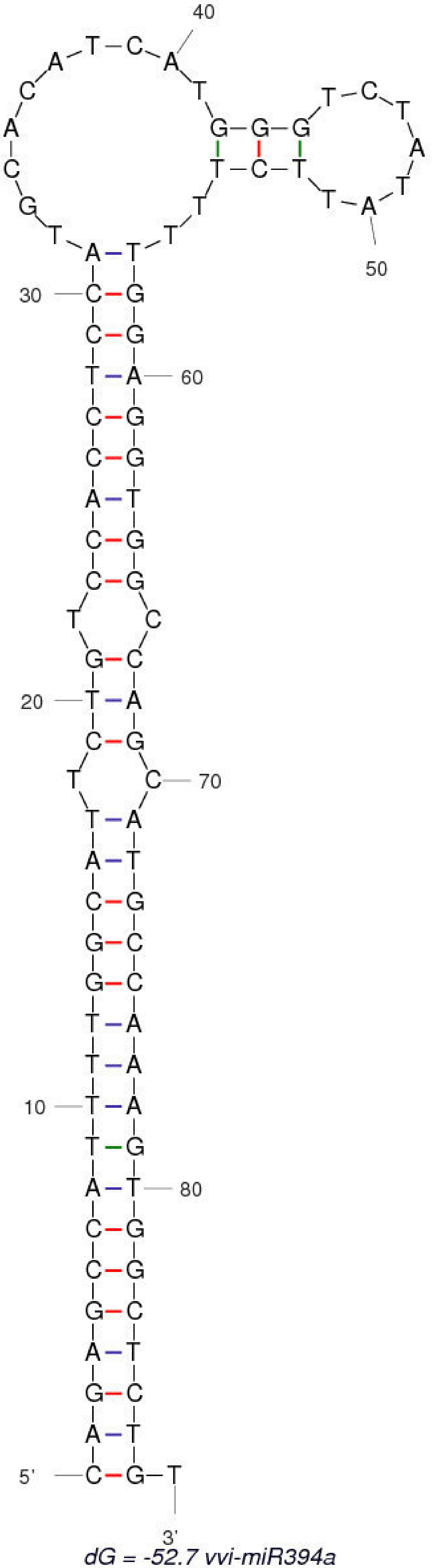

dG = -52.7 vvi-miR394a

Output of sir_graph (©)
mfold_util 4.7

Created Tue Aug 30 08:01:43 2016

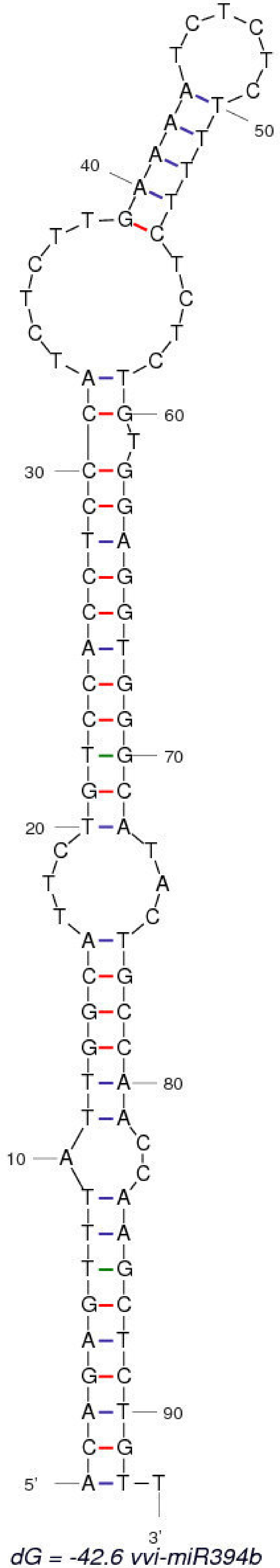

dG = -42.6 vvi-miR394b

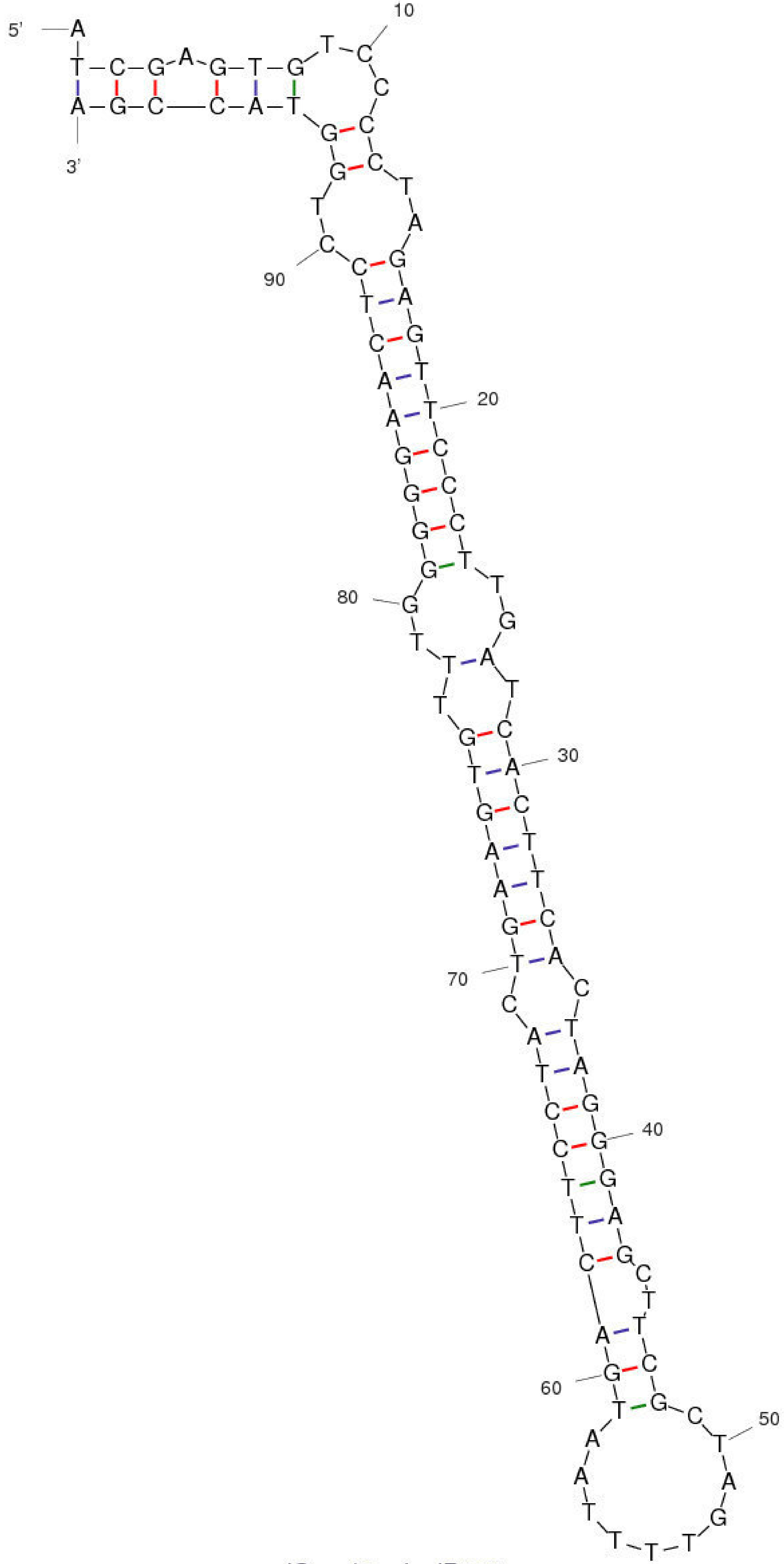

dG = -43 vvi-miR395a

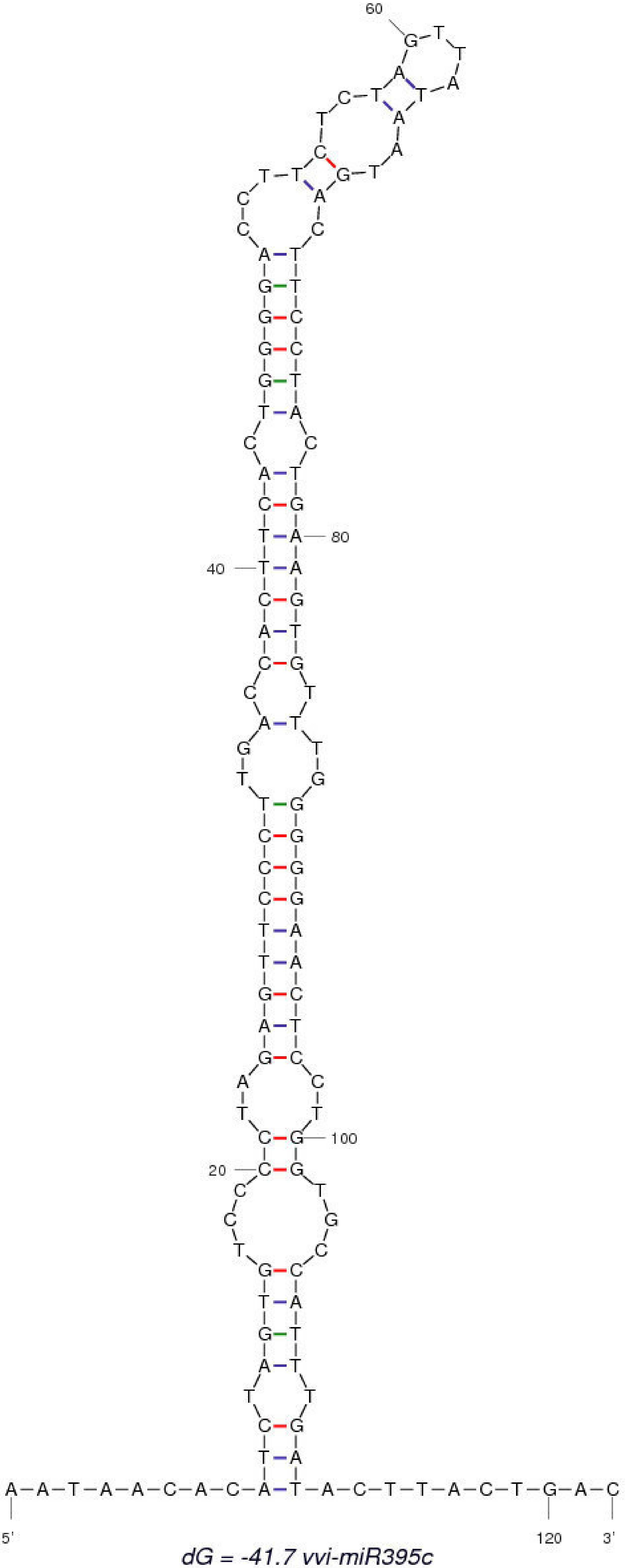

dG = -41.7 vvi-miR395c

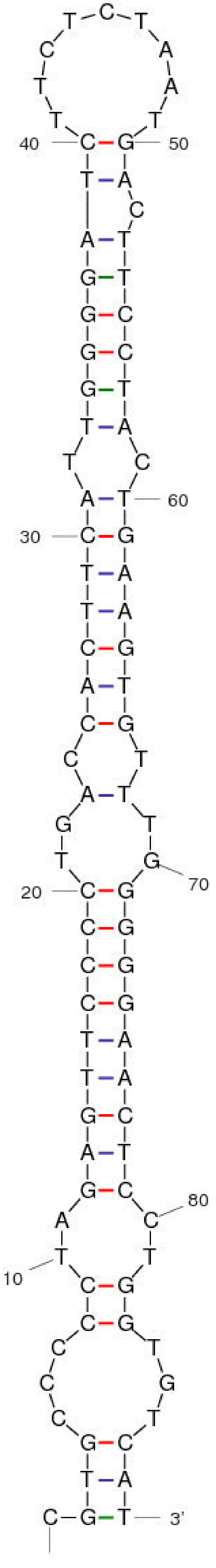

dG = -41.2 vvi-miR395d

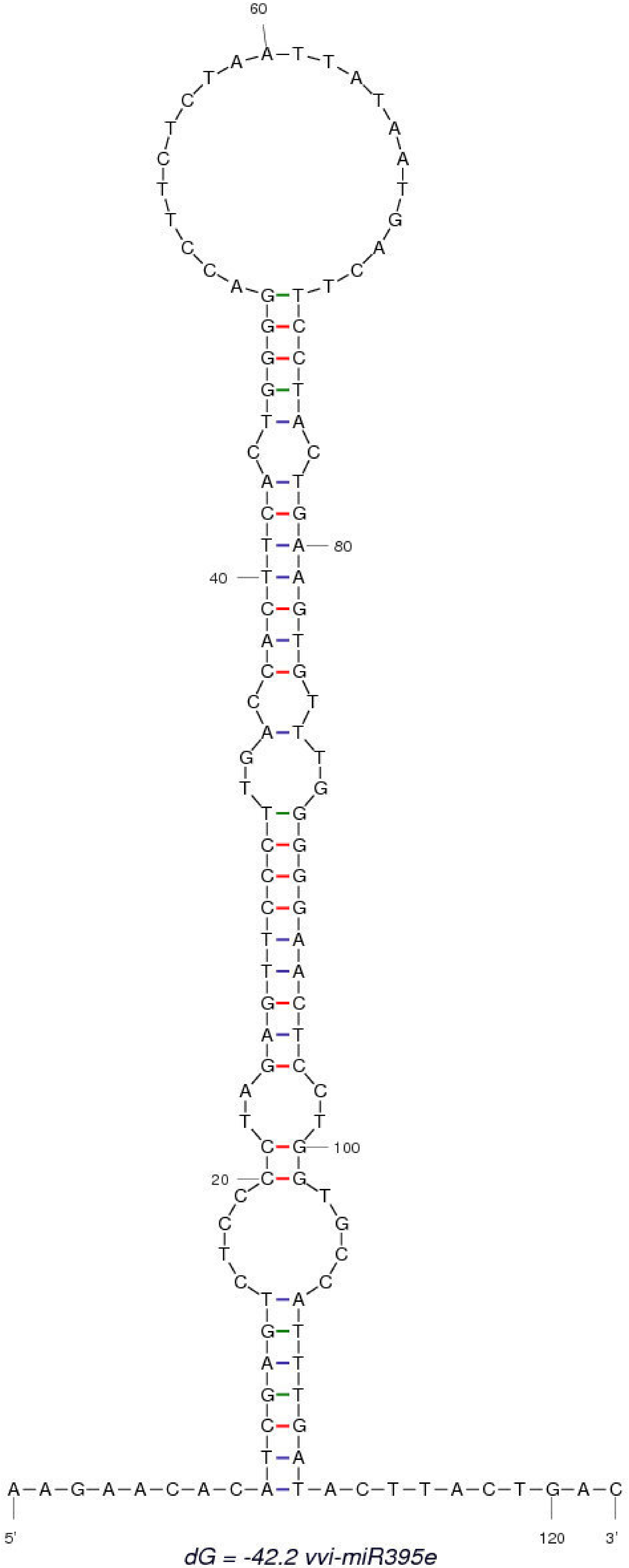

dG = -42.2 vvi-miR395e

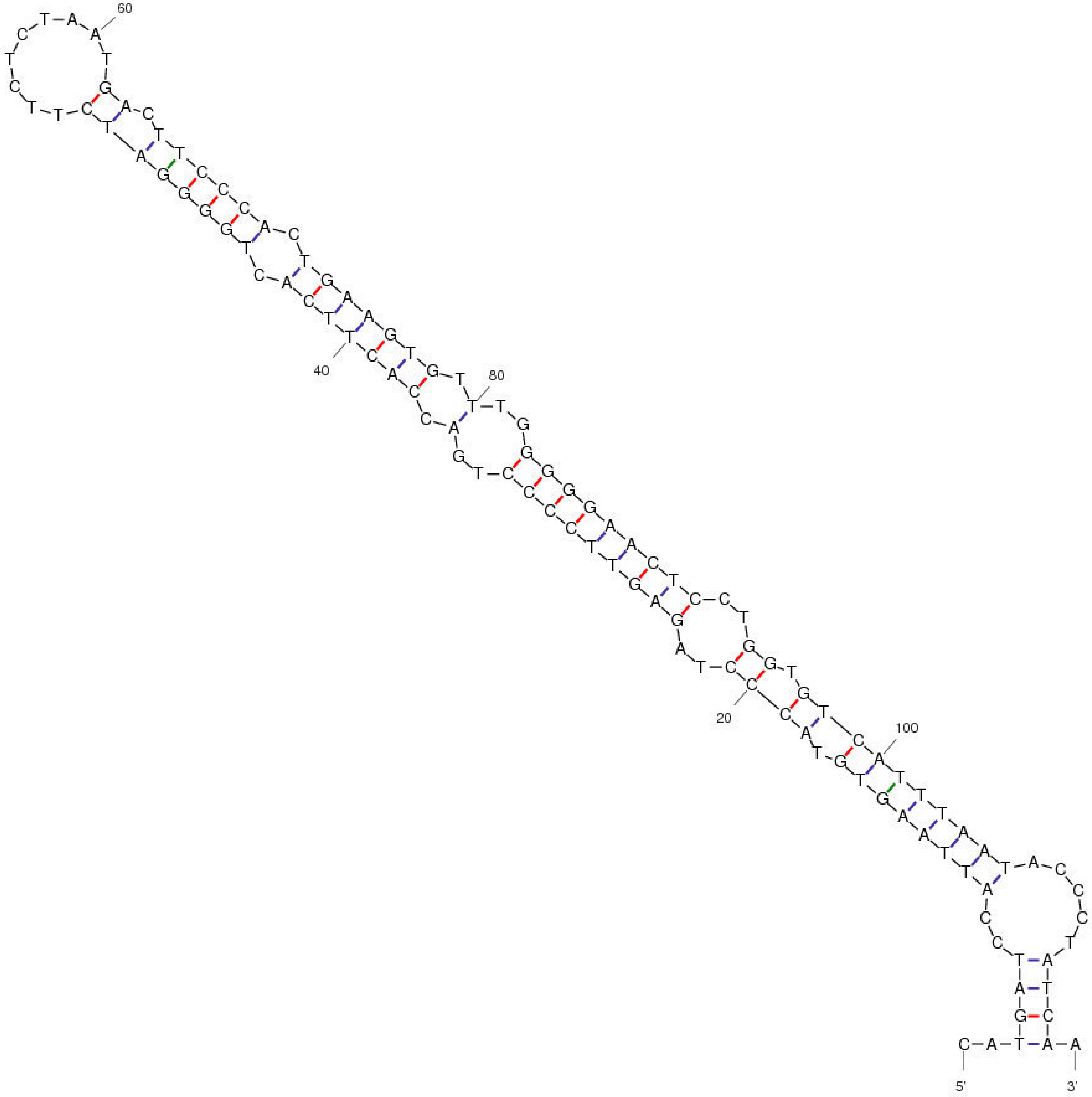

dG = -51.6 vvi-miR395f

Output of sir_graph (©)
mfold_util 4.7

Created Tue Aug 30 08:01:44 2016

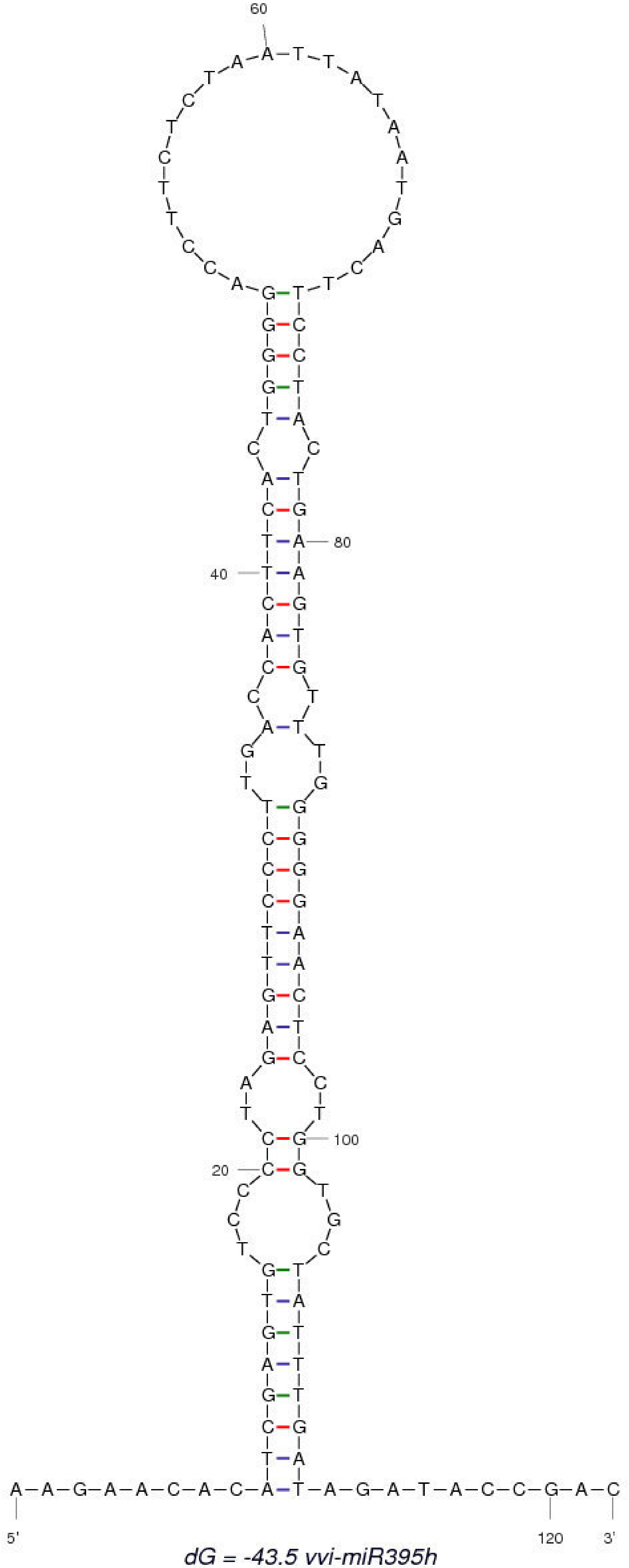

dG = -43.5 vvi-miR395h

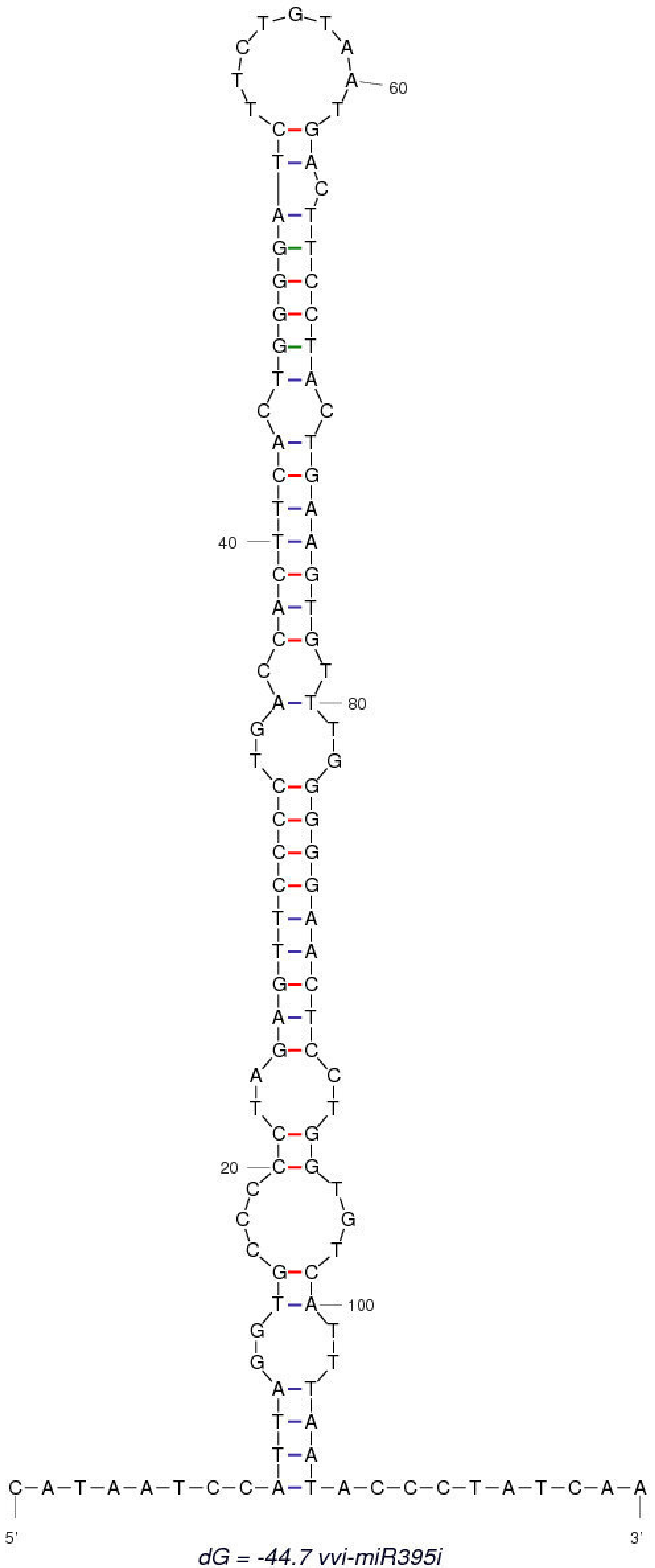

dG = -44.7 vvi-miR395i

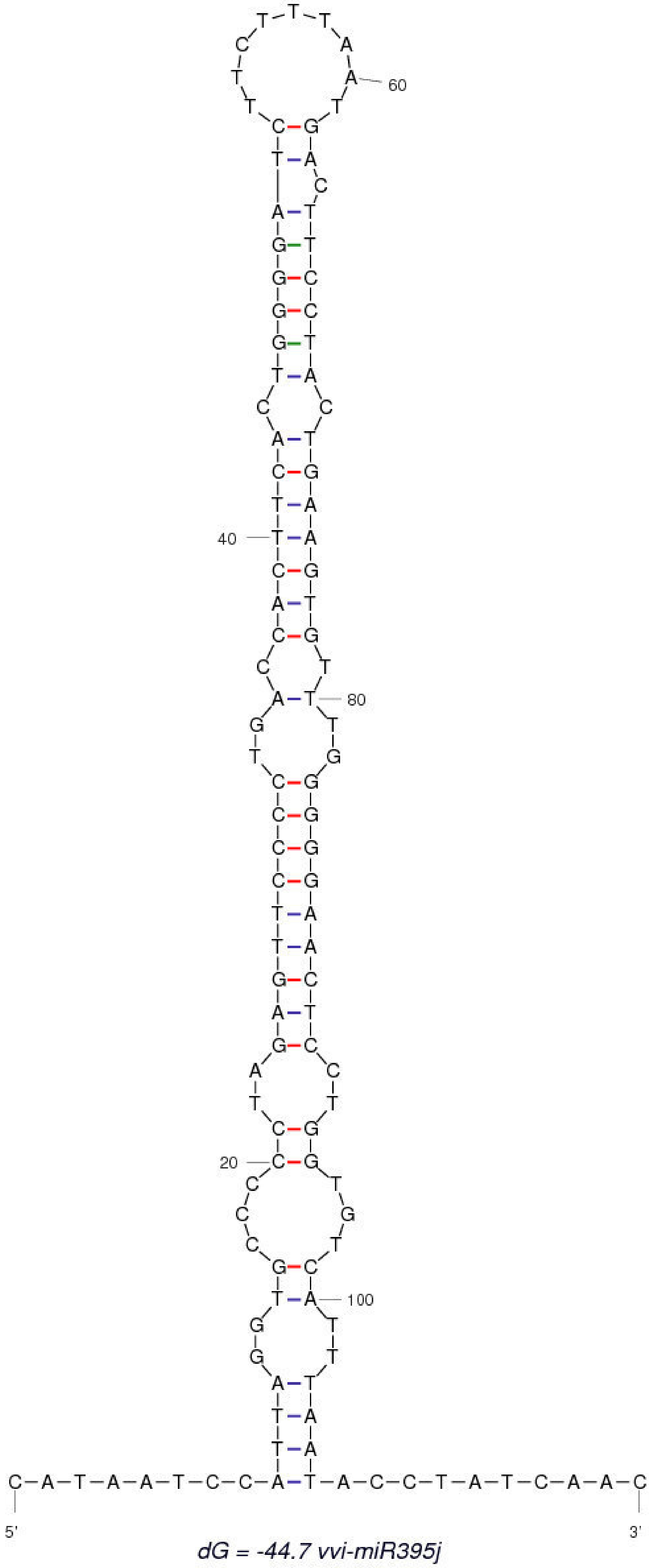

dG = -44.7 vvi-miR395j

Output of sir_graph (©)
mfold_util 4.7

Created Tue Aug 30 08:01:44 2016

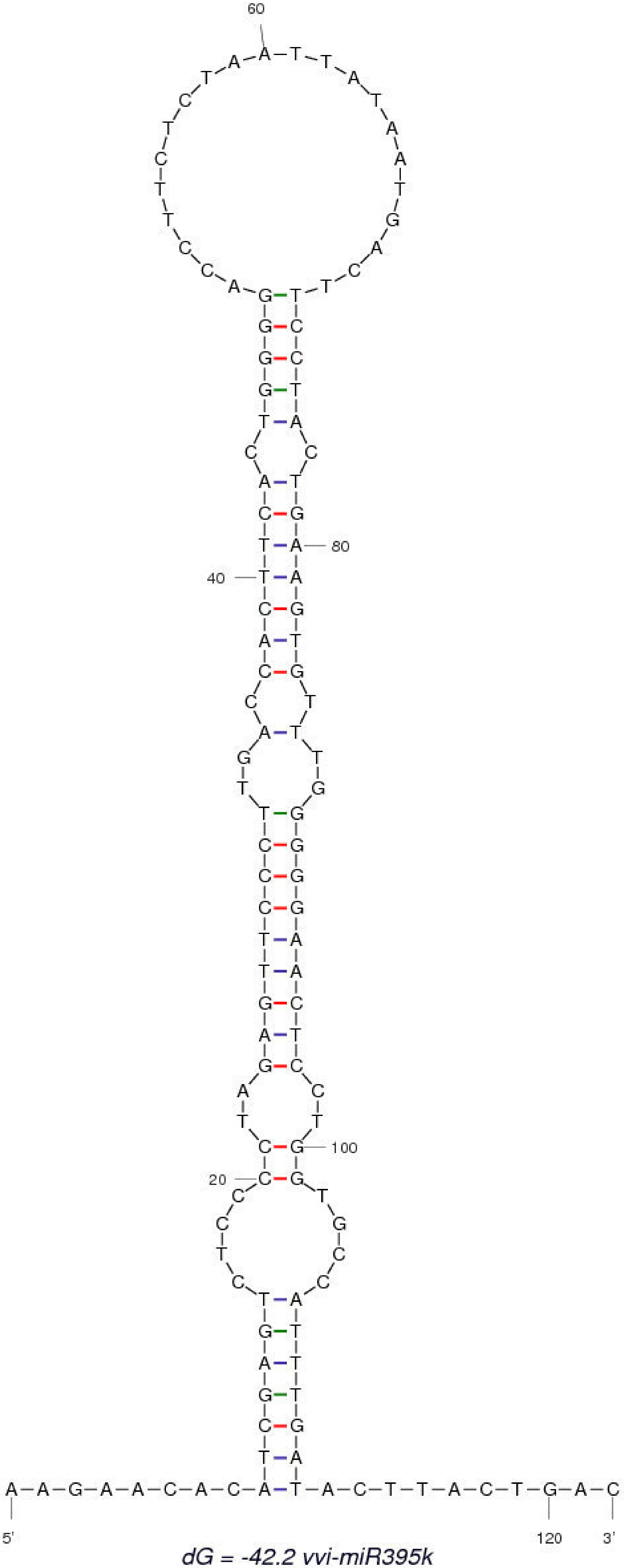

dG = -42.2 vvi-miR395k

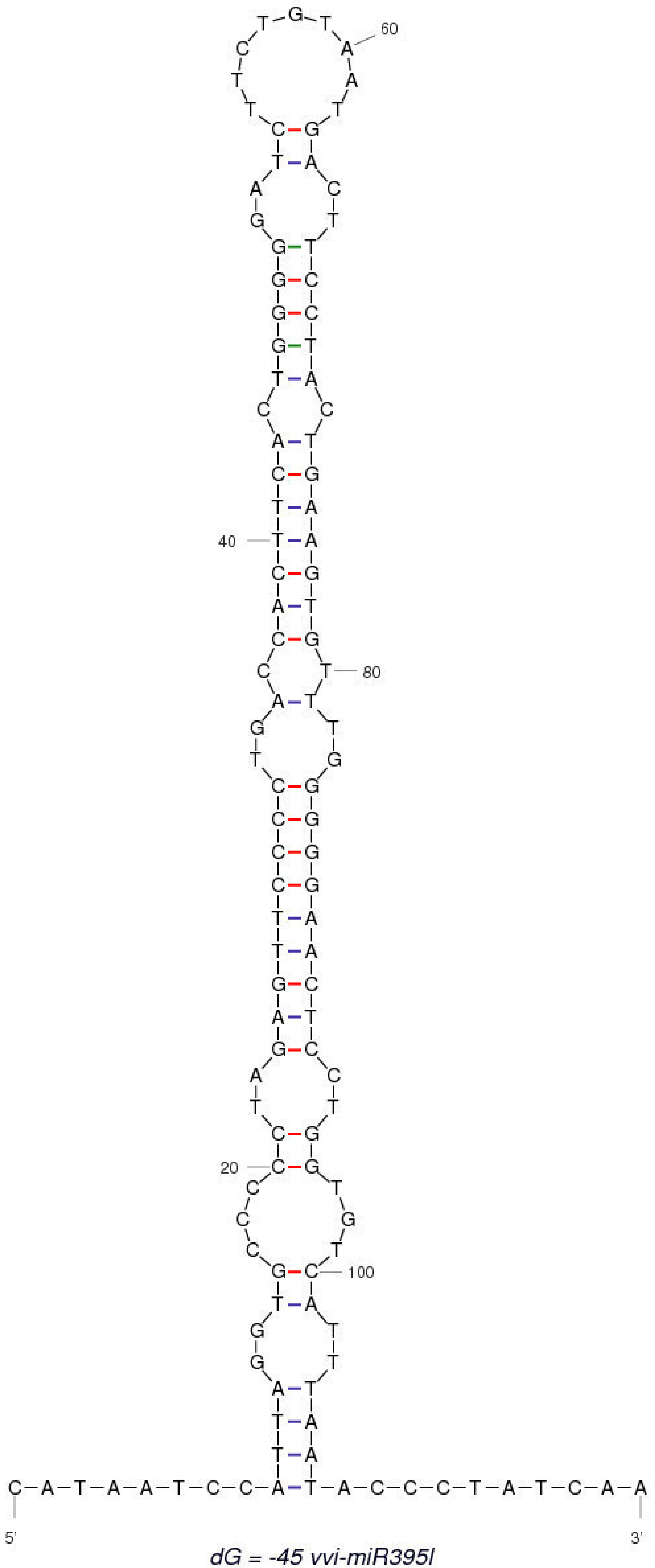

dG = -45 vvi-miR395l

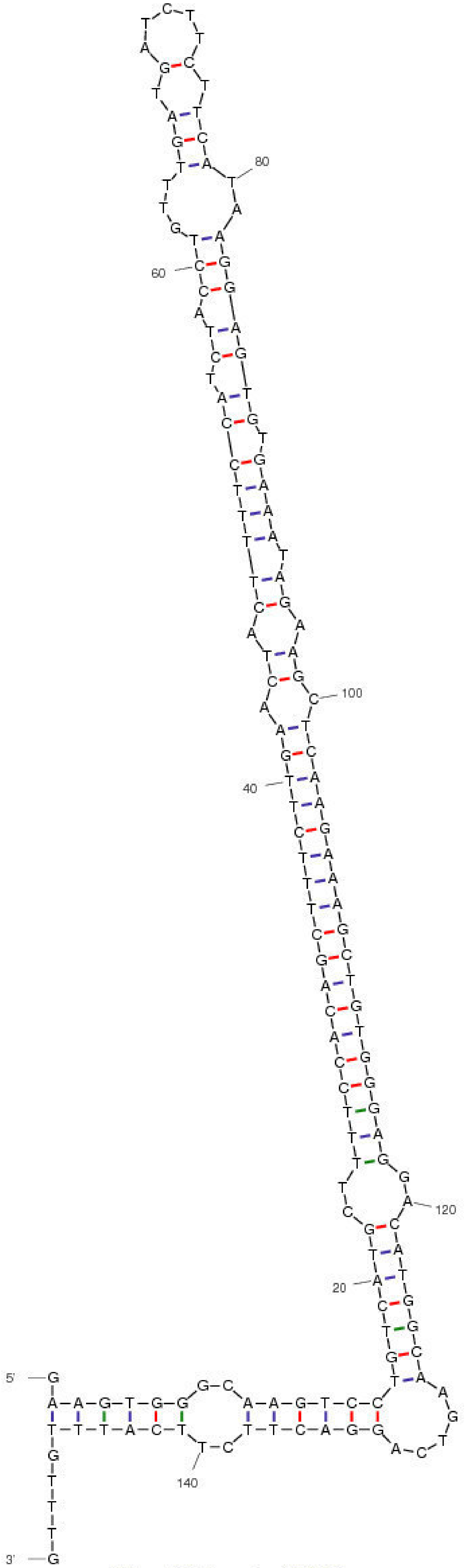

dG = -62.1 vvi-miR396a

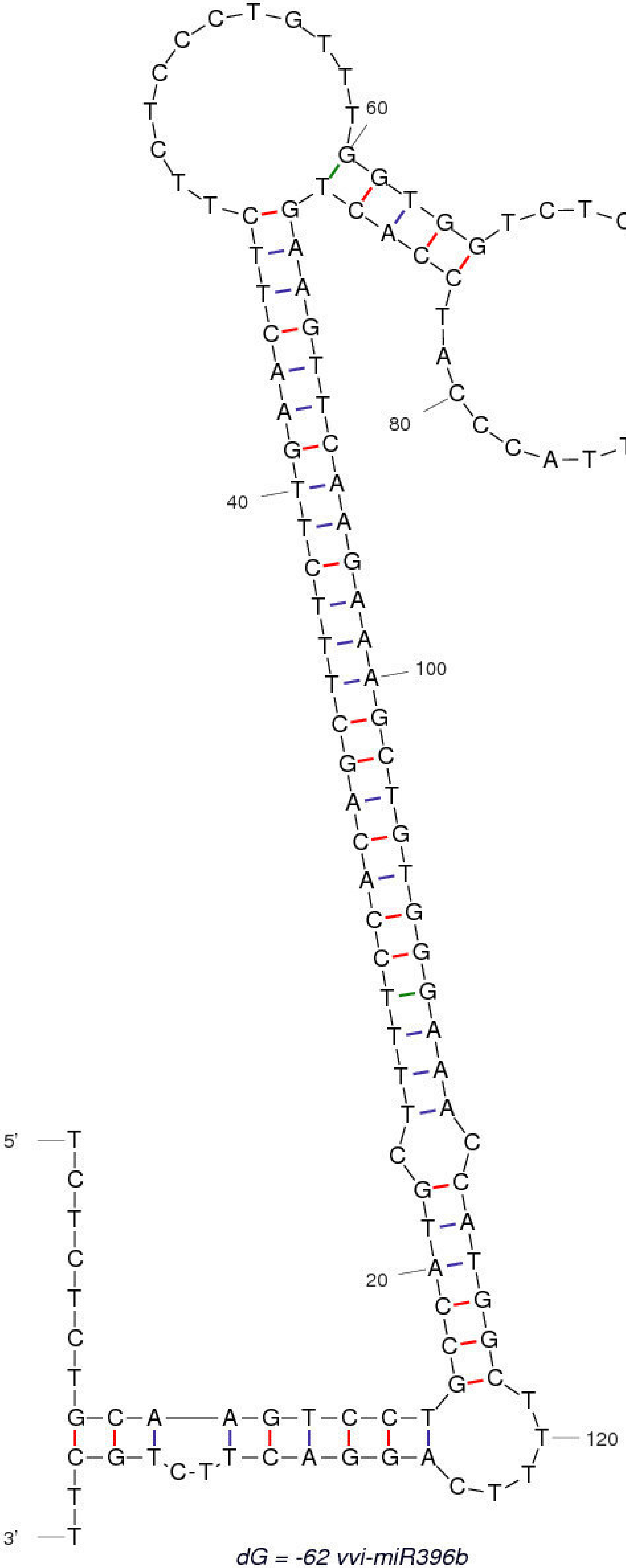

dG = -62 vvi-miR396b

Output of sir_graph (©)
mfold_util 4.7

Created Tue Aug 30 08:01:45 2016

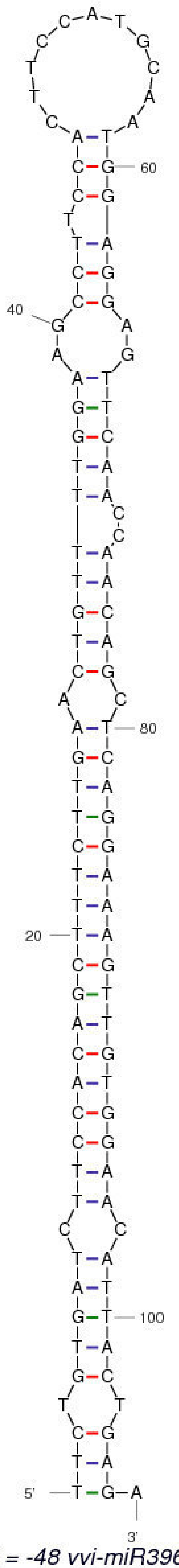

dG = -48 vvi-miR396c

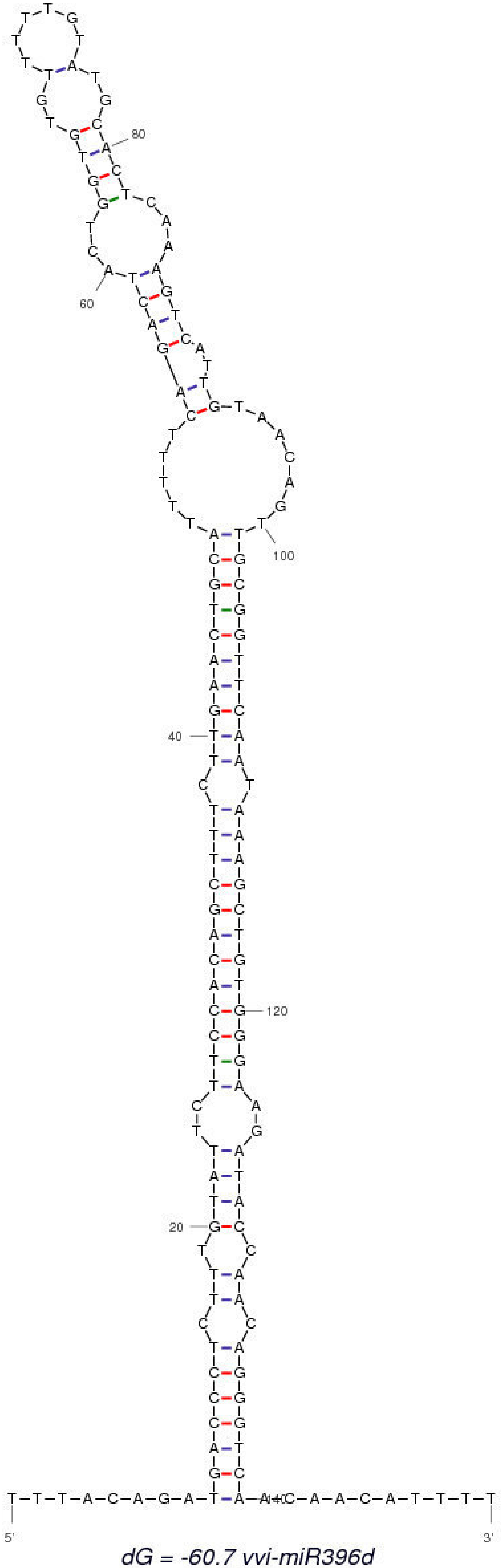

dG = -60.7 vvi-miR396d

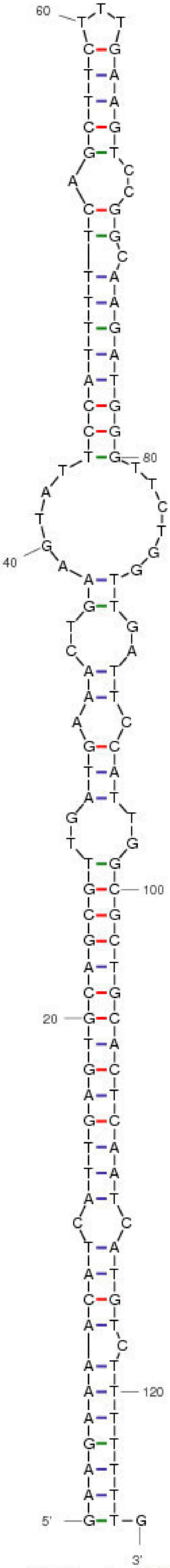

dG = -48.7 vvi-miR397a

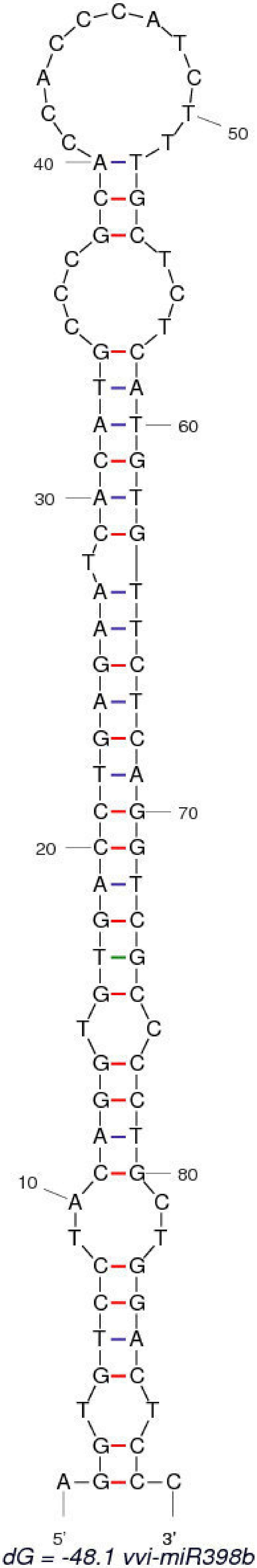

dG = -48.1 vvi-miR398b

Output of sir_graph (©)
mfold_util 4.7

Created Tue Aug 30 08:05:59 2016

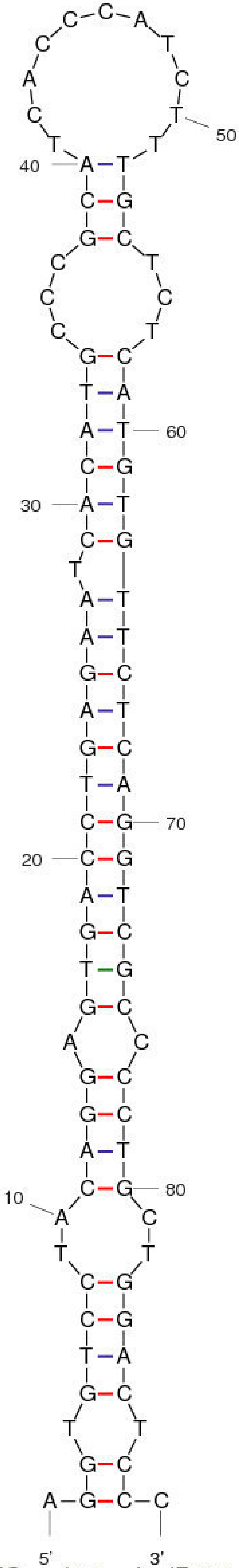

dG = -49.2 vvi-miR398c

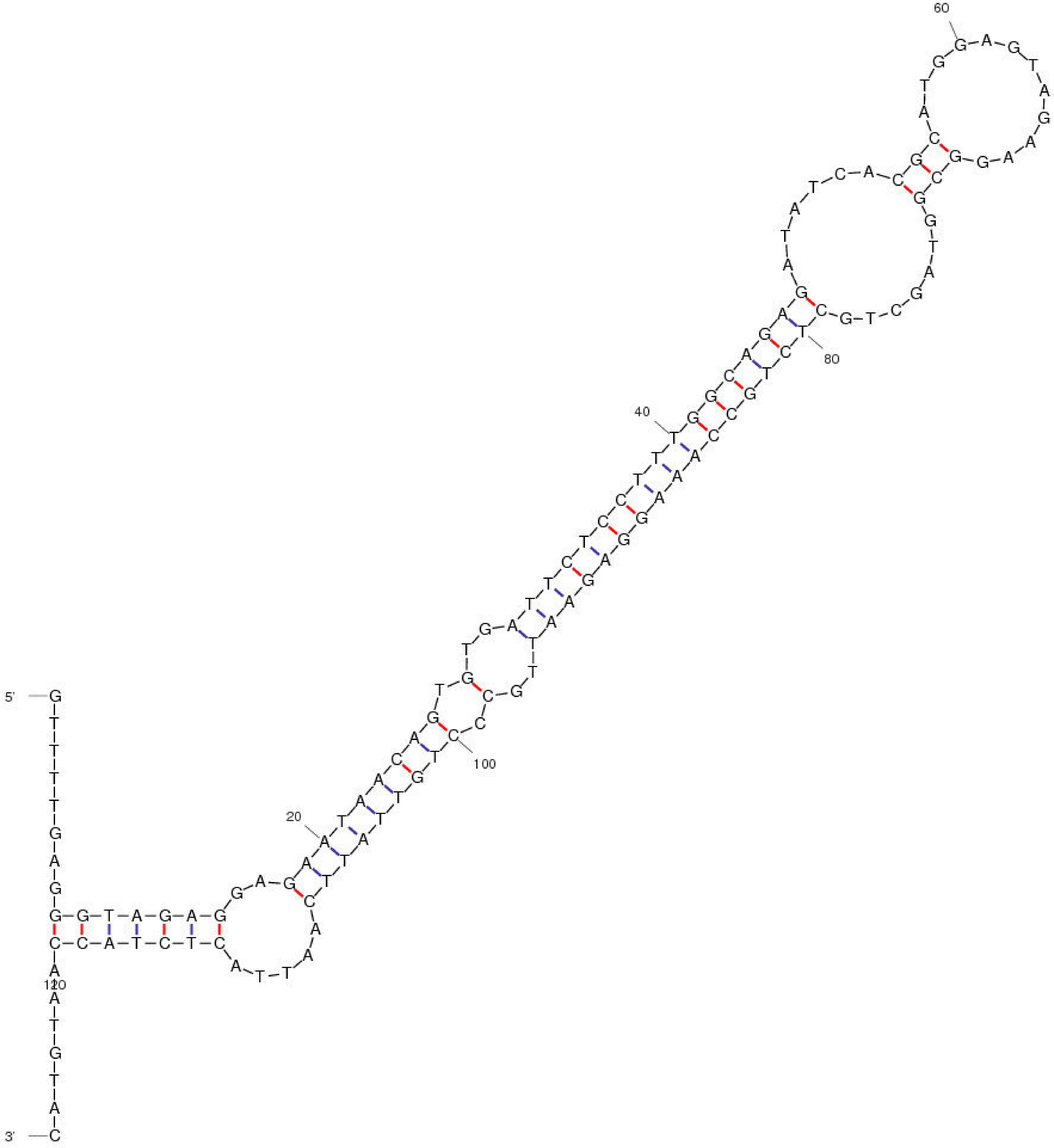

dG = -61.9 vvi-miR399a

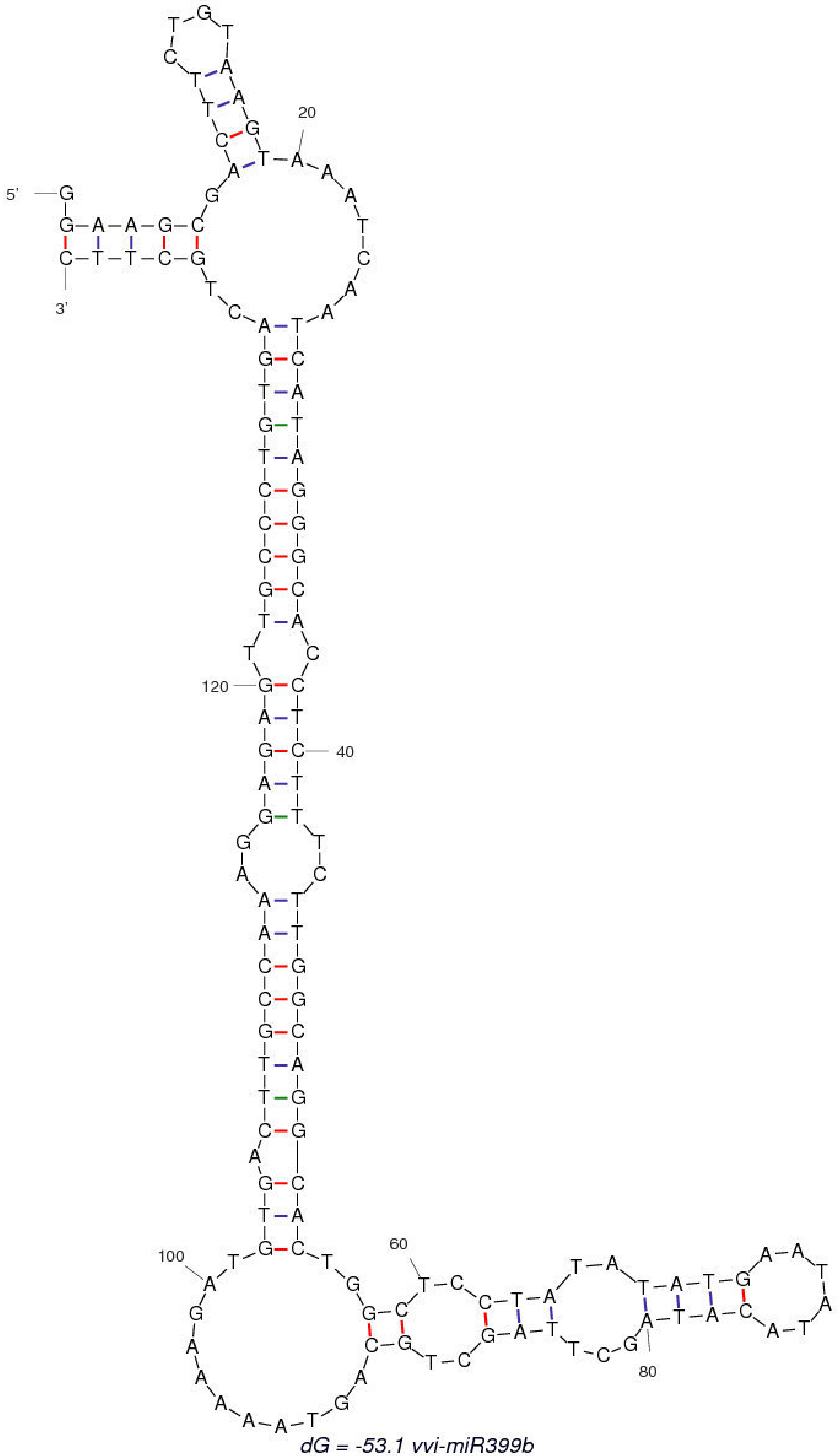

dG = -53.1 vvi-miR399b

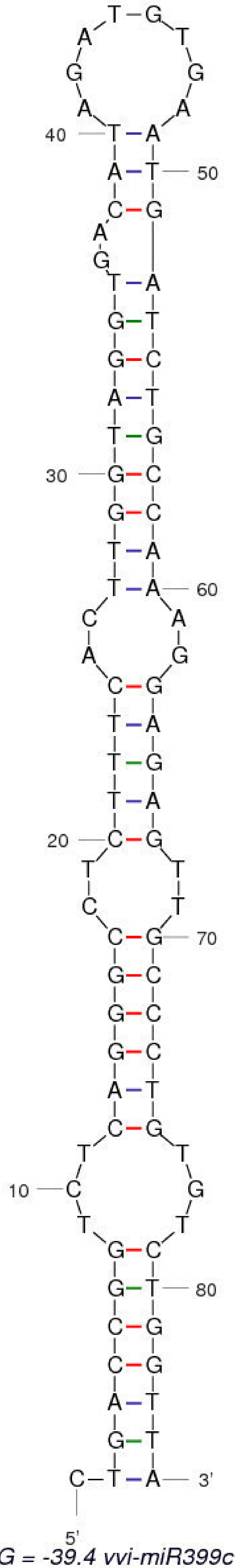

dG = -39.4 vvi-miR399c

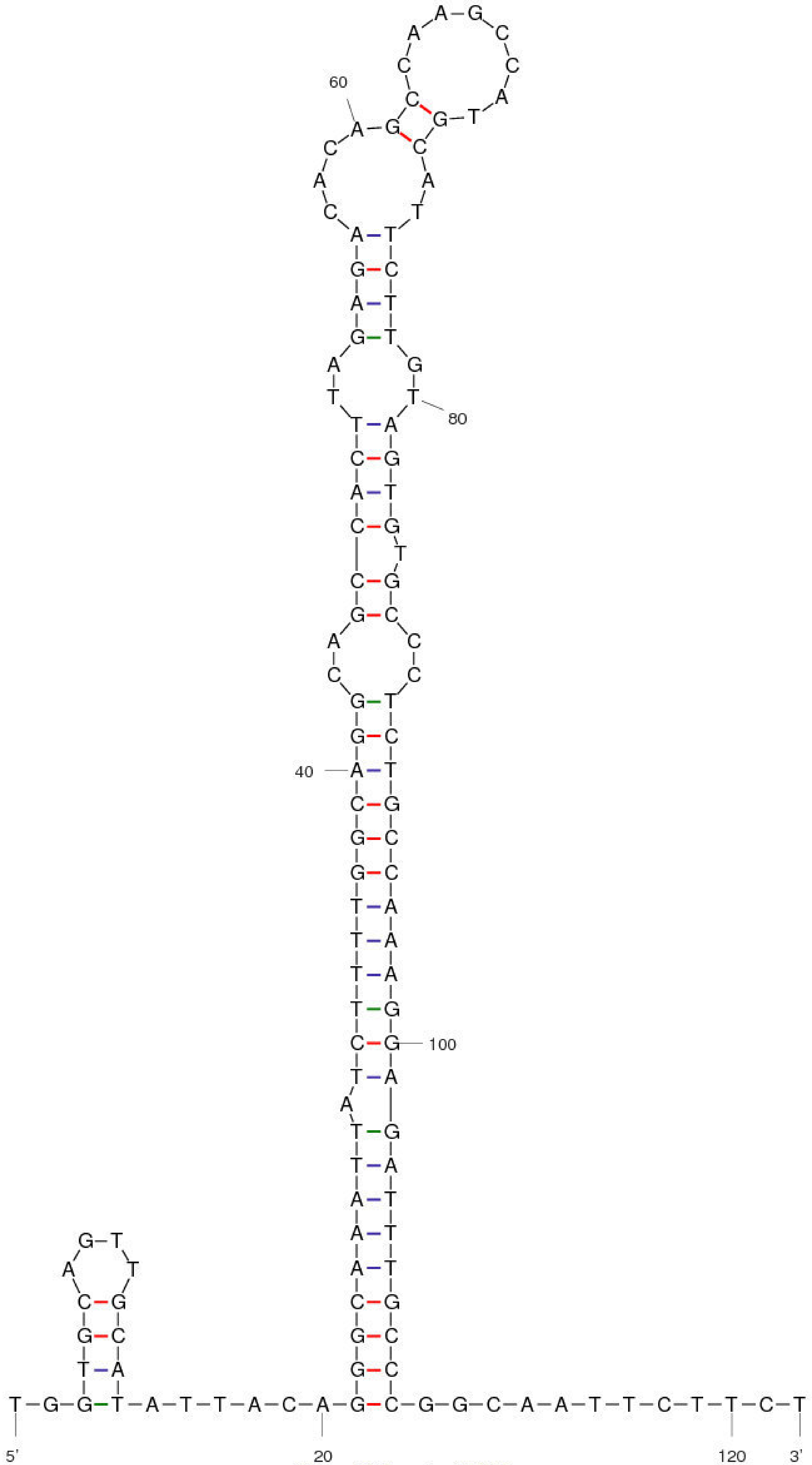

dG = -45.6 vvi-miR399e

Output of sir_graph (©)
mfold_util 4.7

Created Tue Aug 30 08:06:00 2016

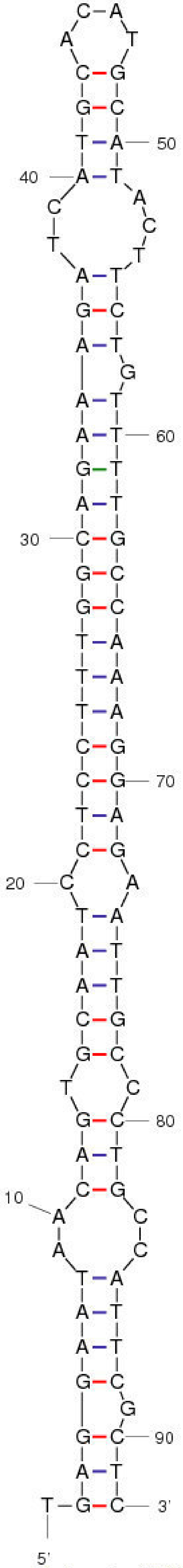

dG = -43.4 vvi-miR399h

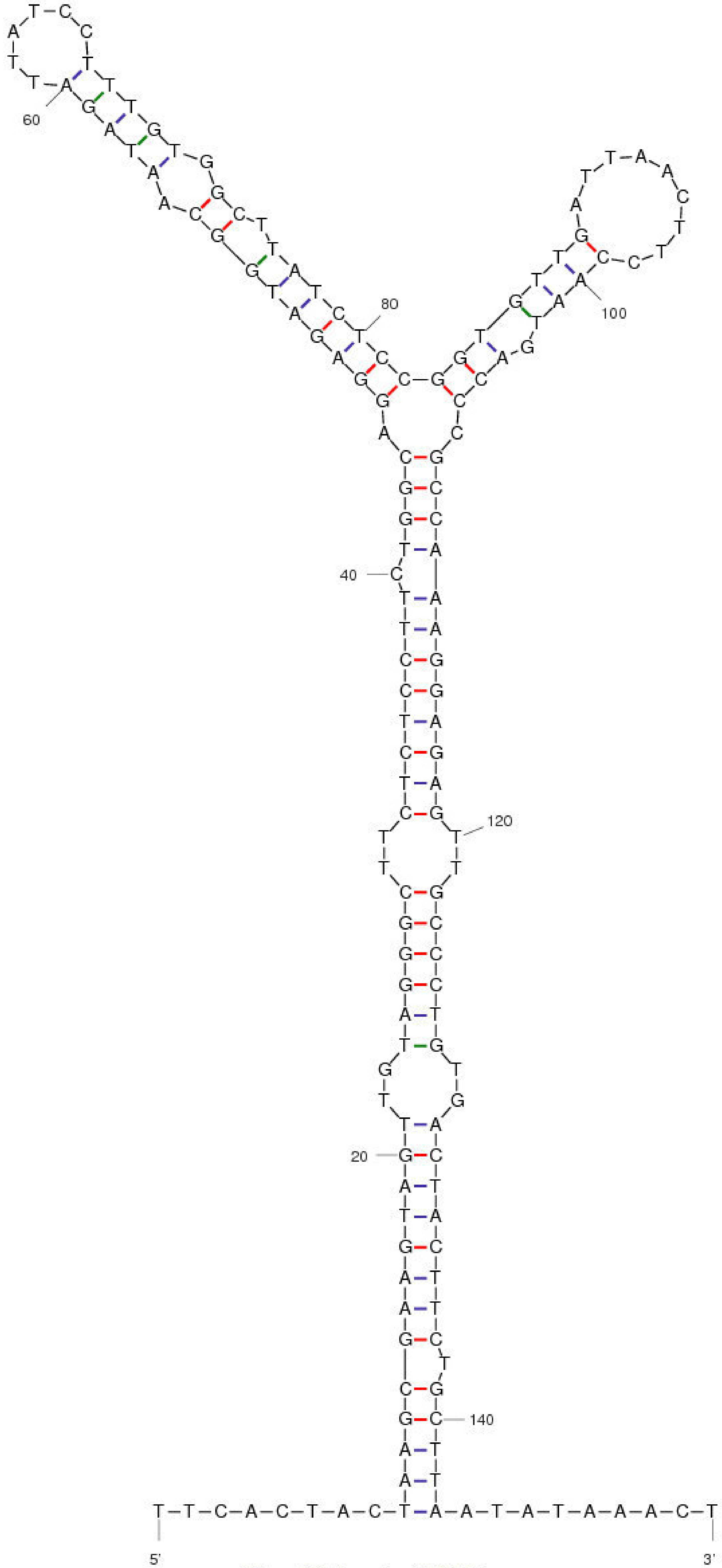

dG = -68.4 vvi-miR399i

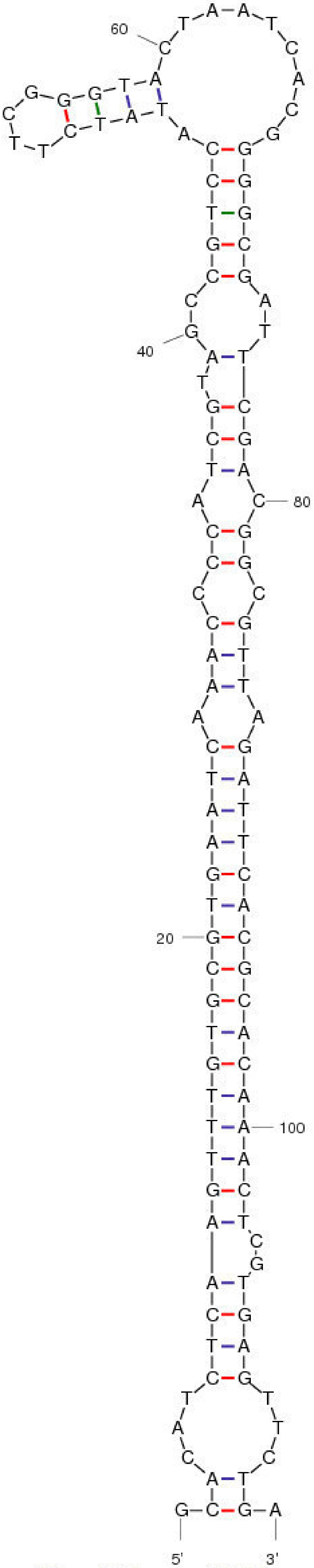

dG = -47.2 vvi-miR403a

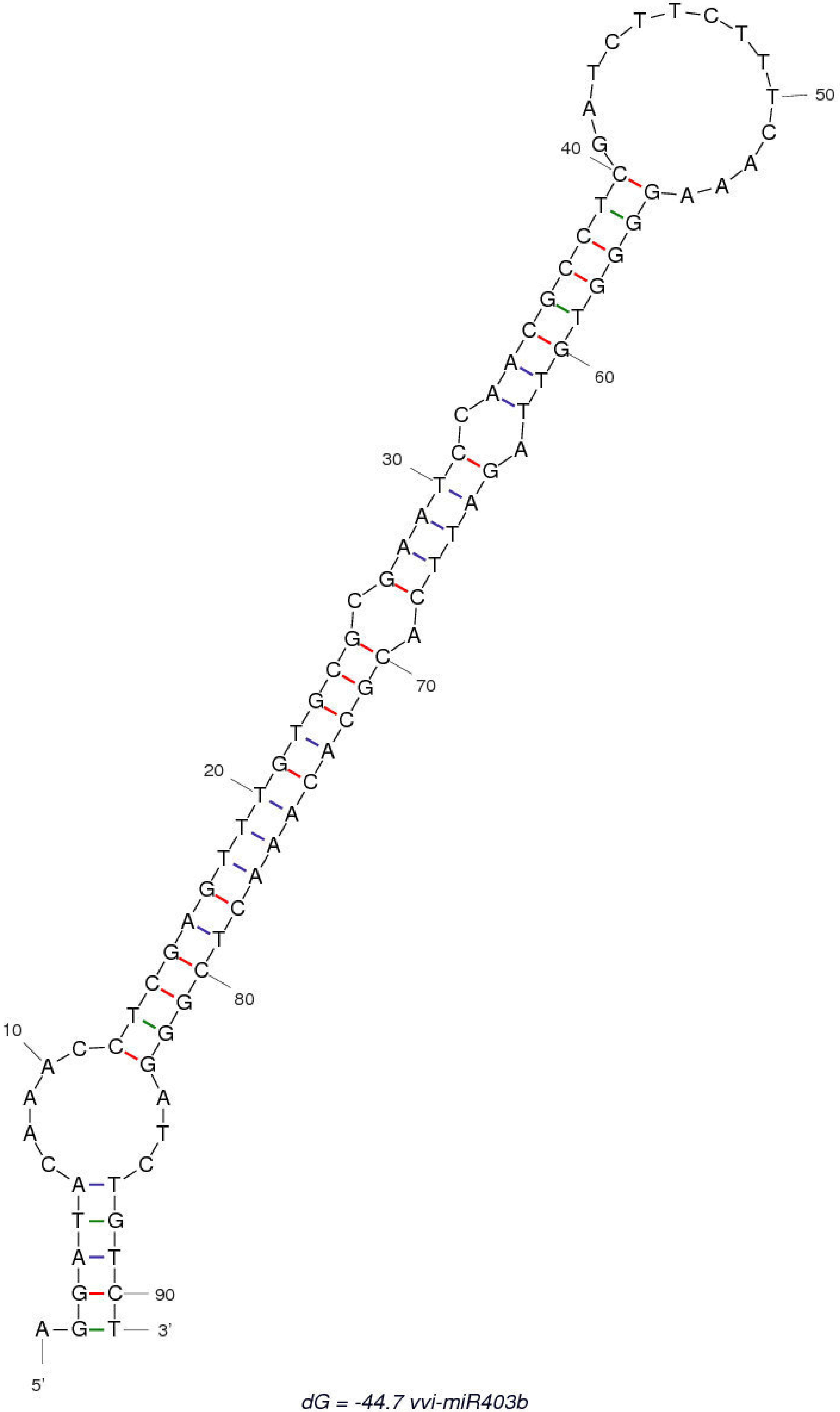

dG = -44.7 vvi-miR403b

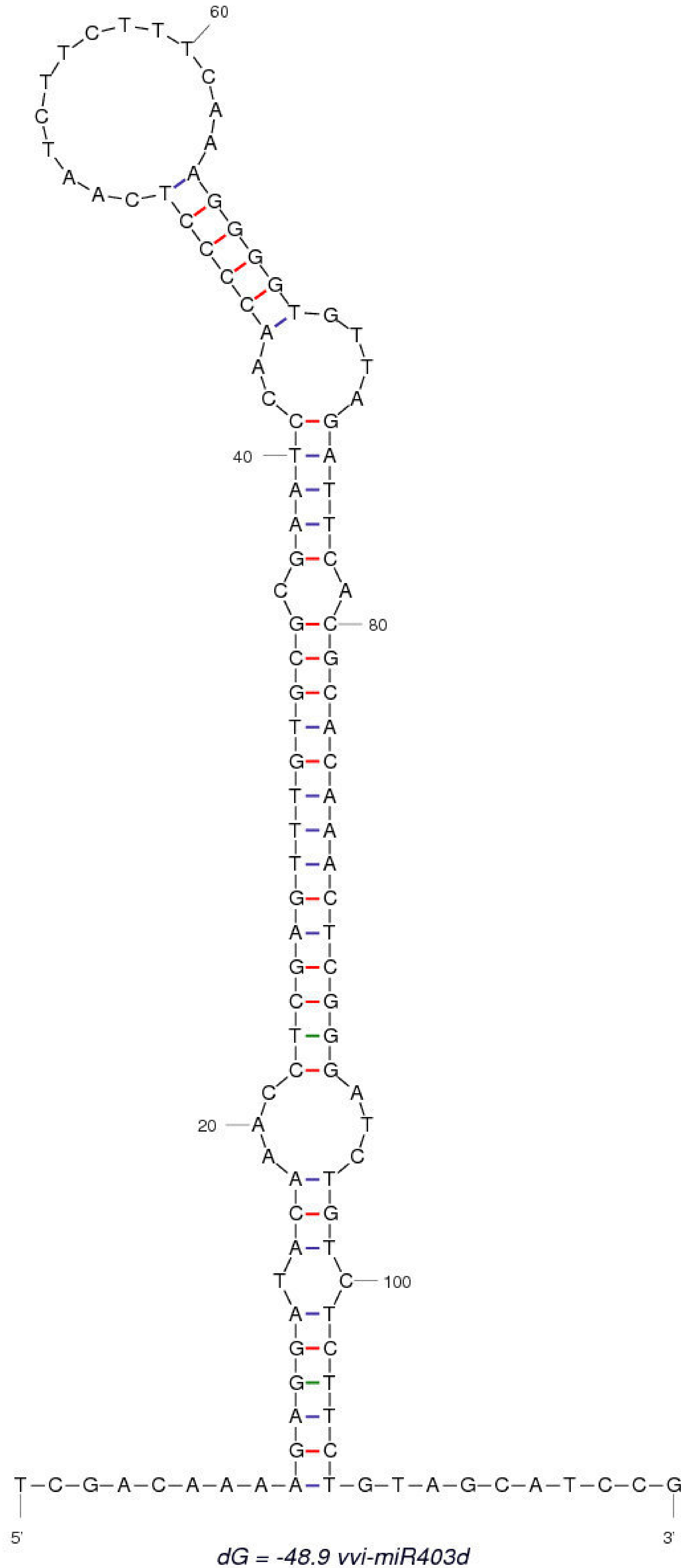

dG = -48.9 vvi-miR403d

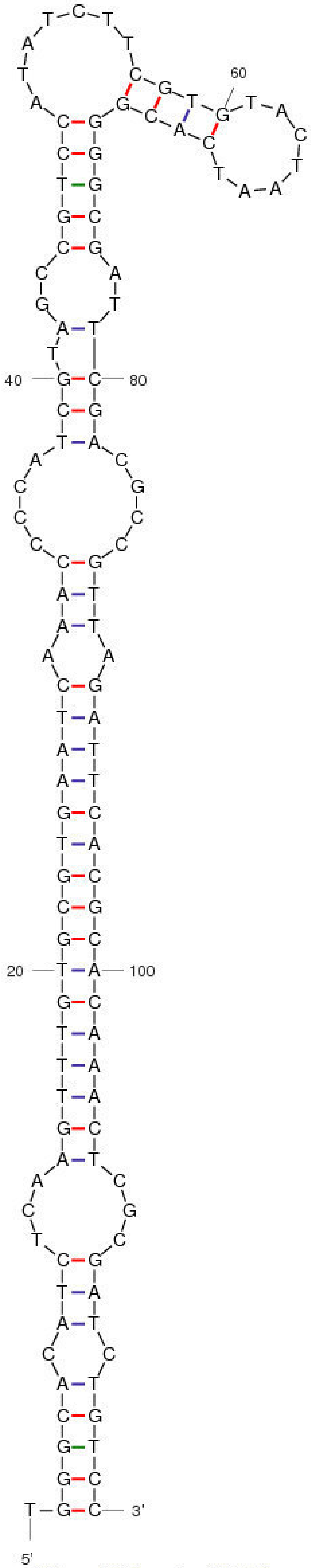

dG = -47.3 vvi-miR403e

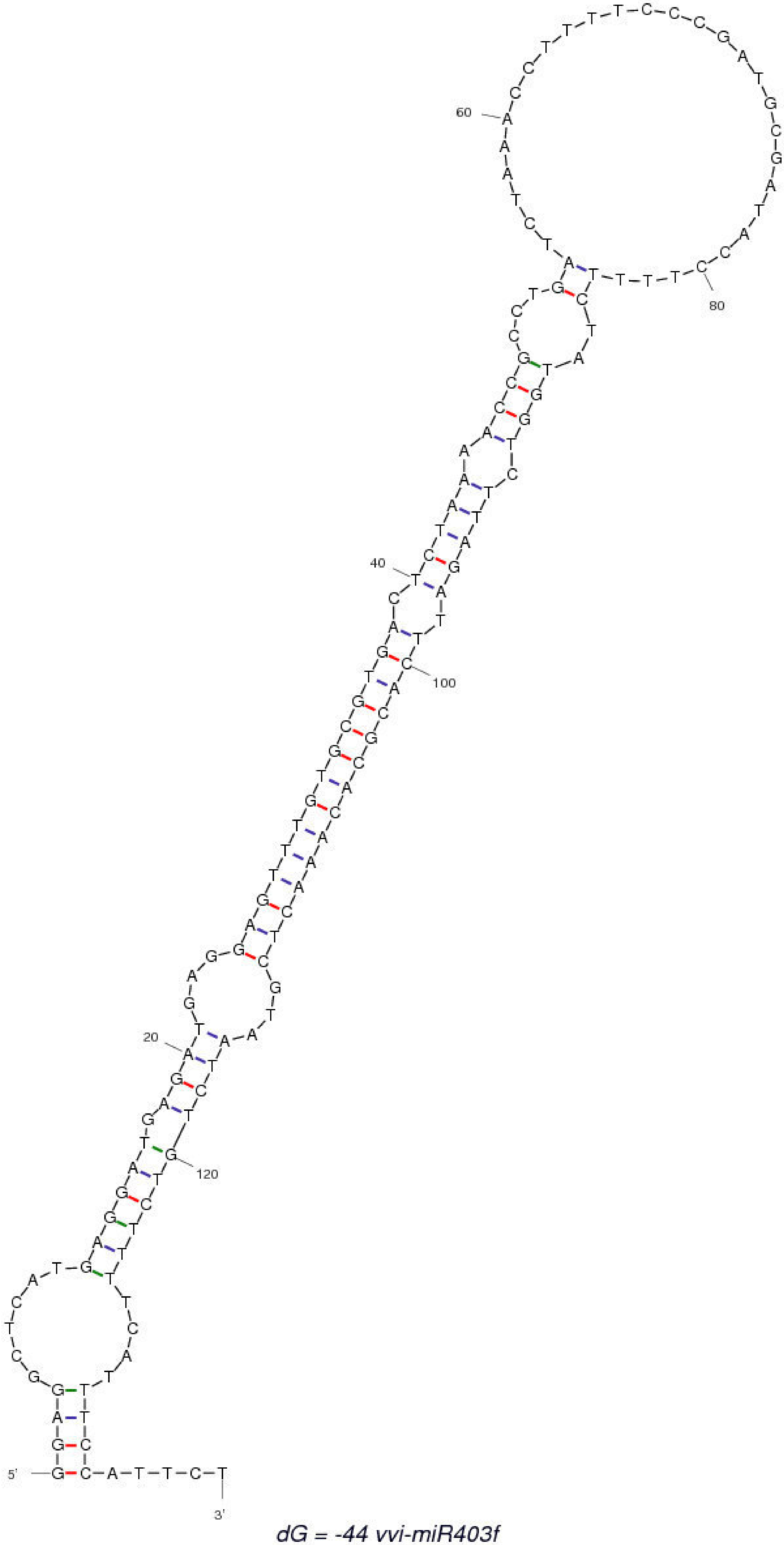

dG = -44 vvi-miR403f

Output of sir_graph (©)
mfold_util 4.7

Created Tue Aug 30 08:06:01 2016

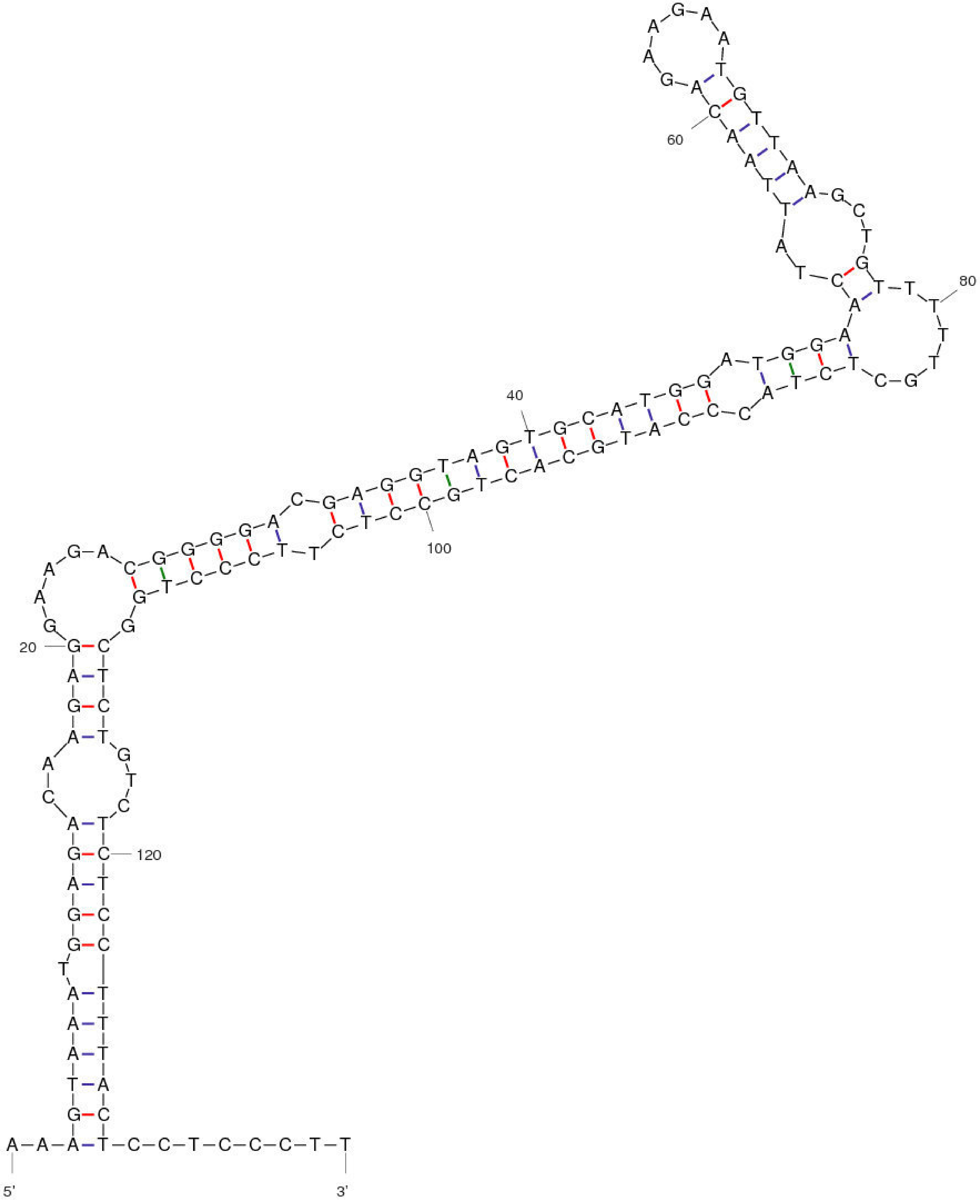

dG = -60.3 vvi-miR408

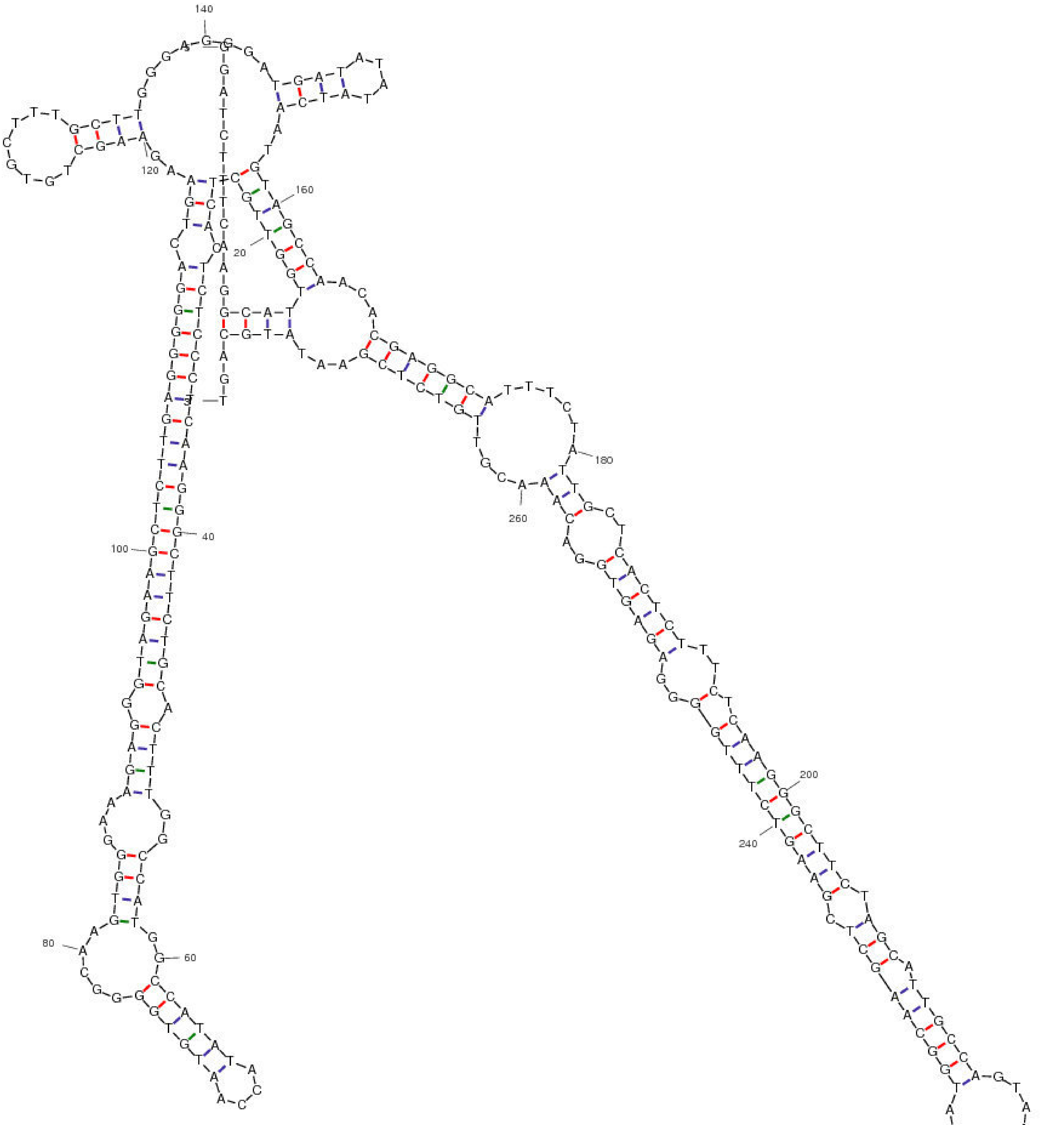

dG = -119.9 vvi-miR477b

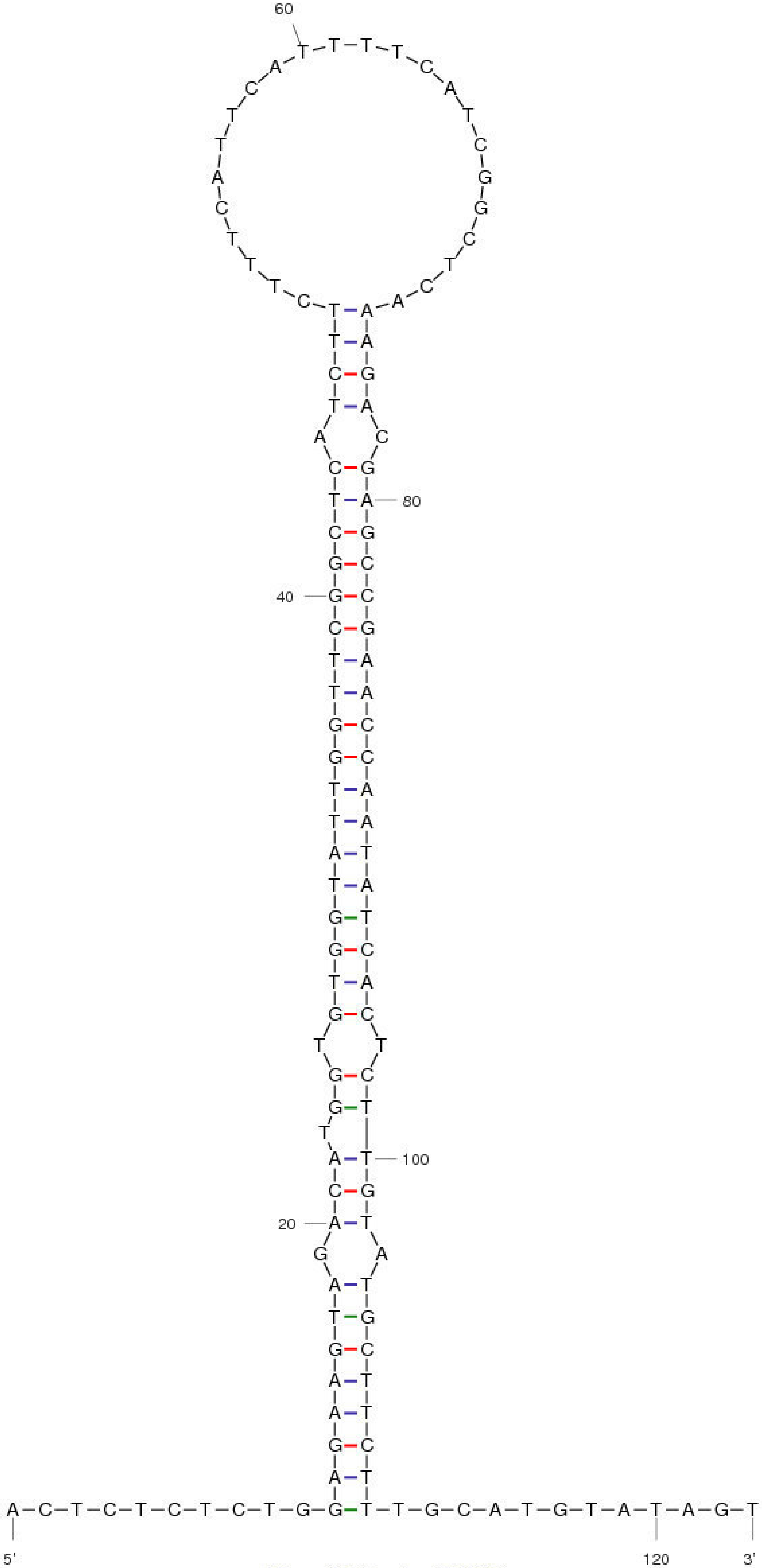

dG = -46.8 vvi-miR479

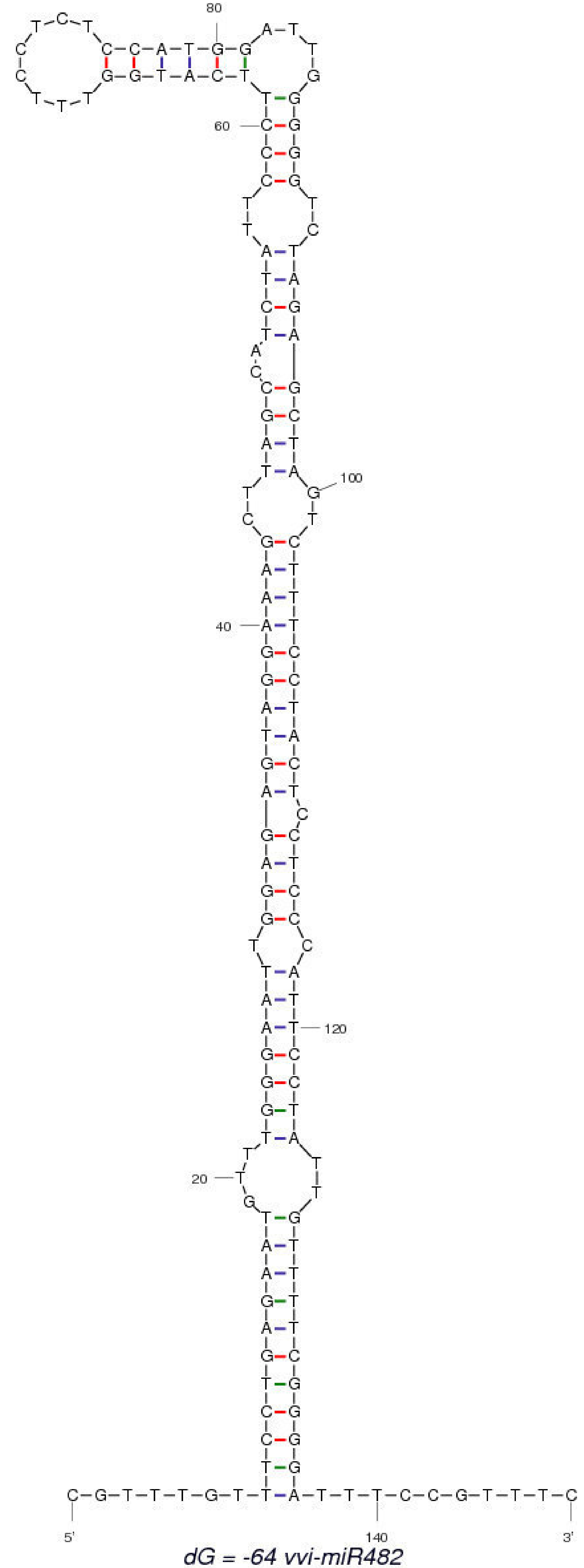

dG = -64 vvi-miR482

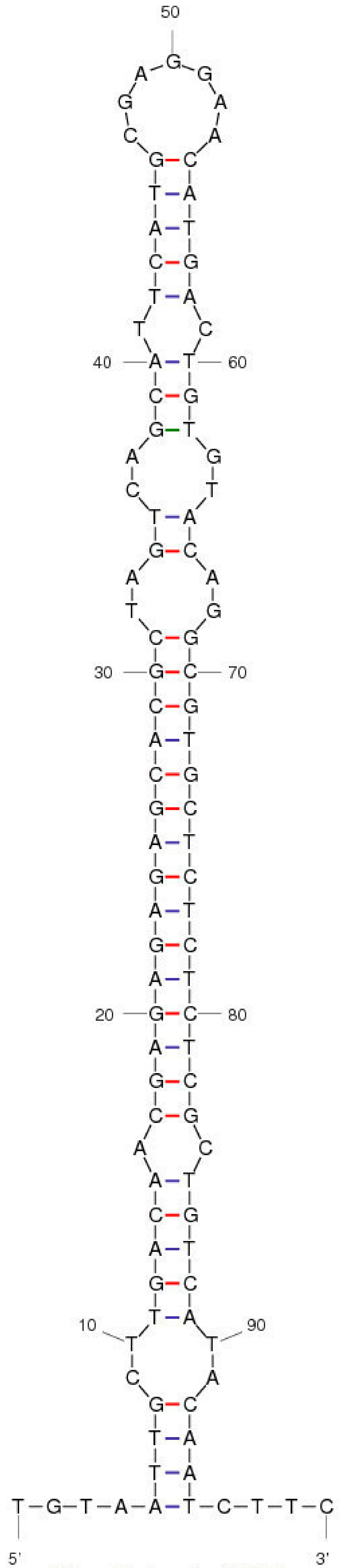

dG = -51.1 vvi-miR535a

Output of sir_graph (©)
mfold_util 4.7

Created Tue Aug 30 08:06:04 2016

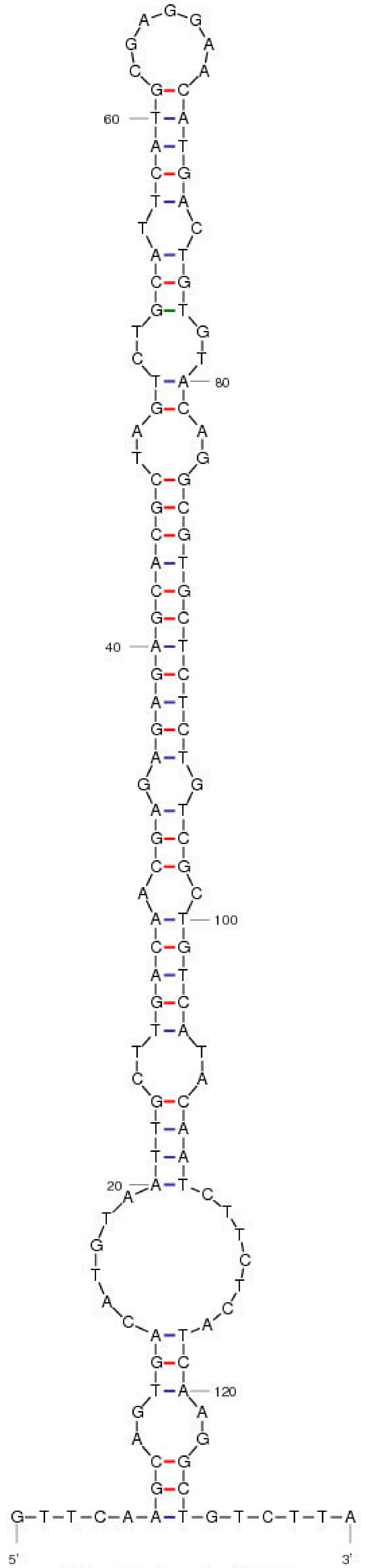

dG = -53.4 vvi-miR535c

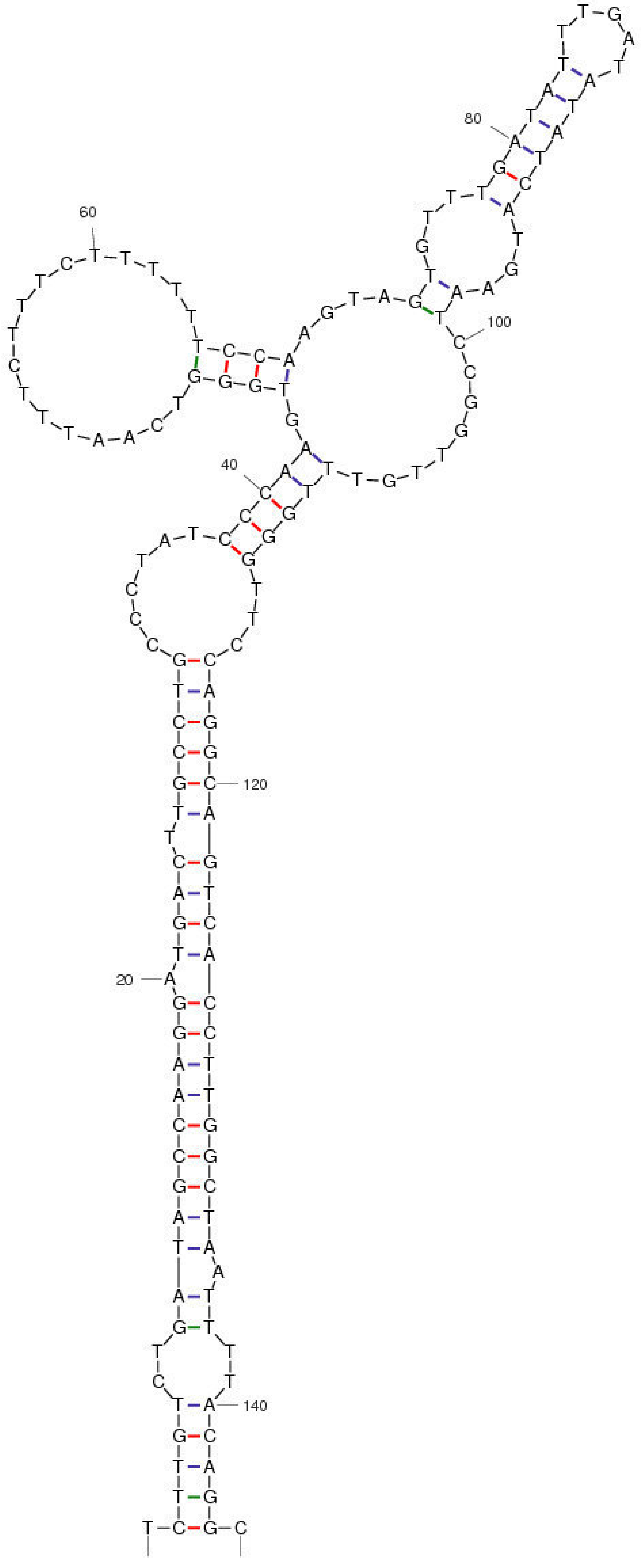

dG = -48.1 vvi-miRC169z

Output of sir_graph (©)
mfold_util 4.7

Created Tue Aug 30 08:08:13 2016

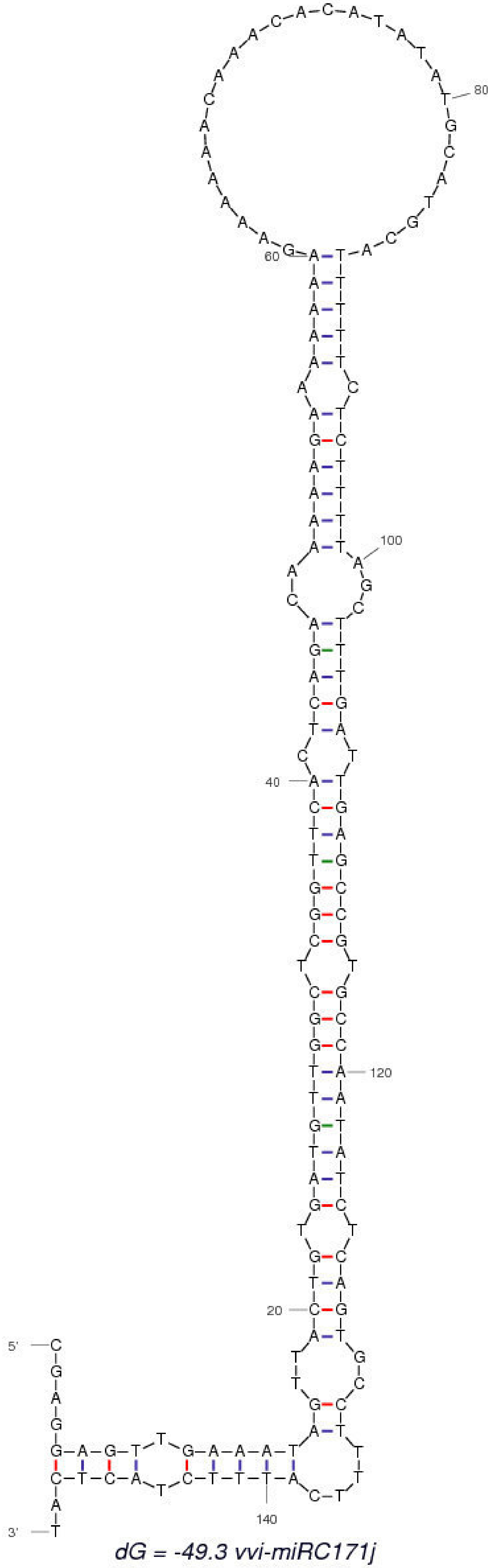

dG = -49.3 vvi-miRC171j

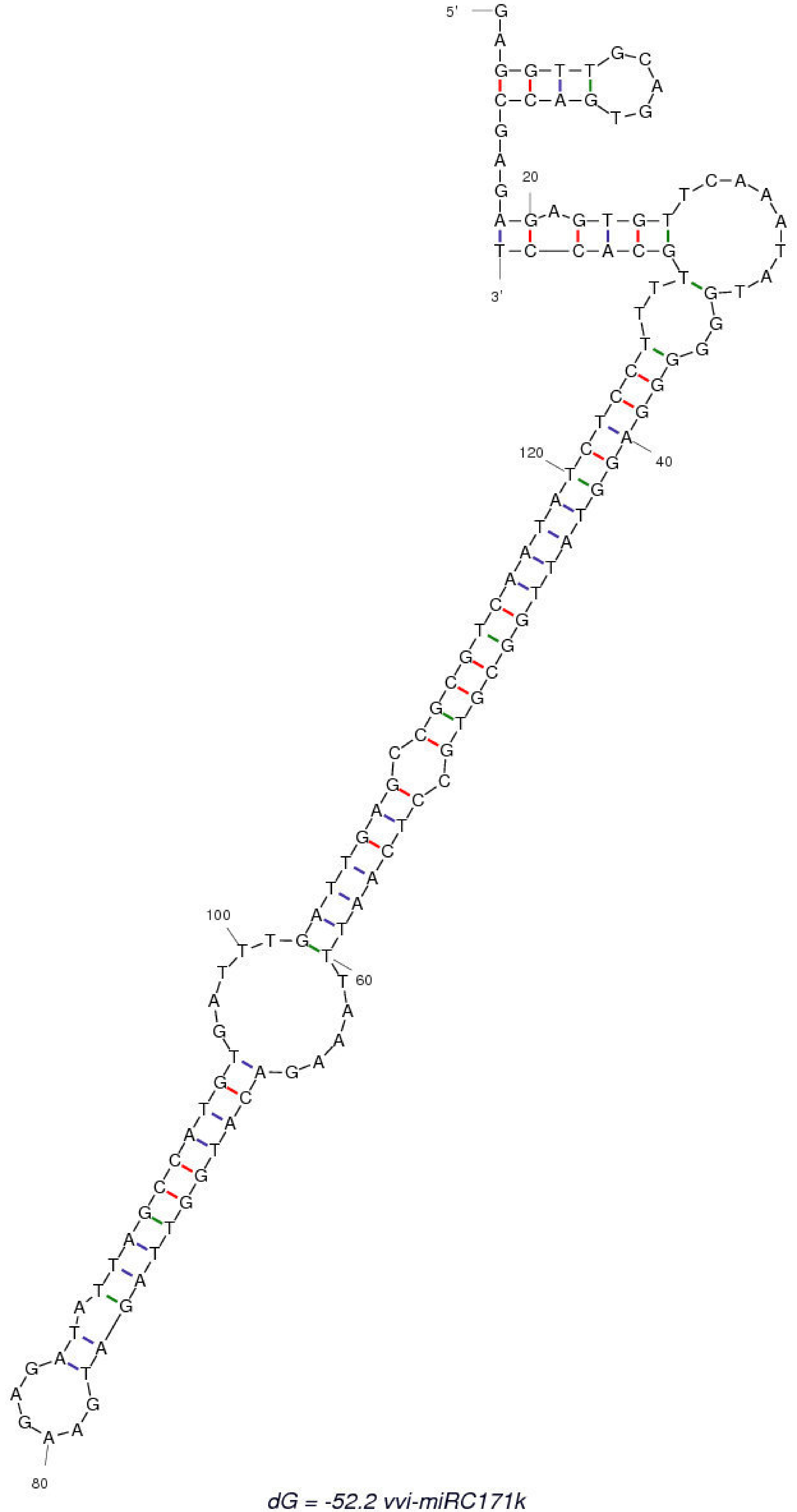

dG = -52.2 vvi-miRC171k

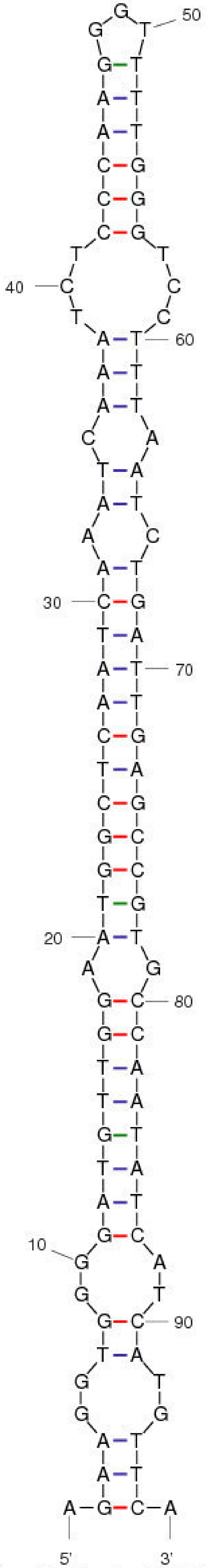

dG = -42.2 vvi-miRC171l

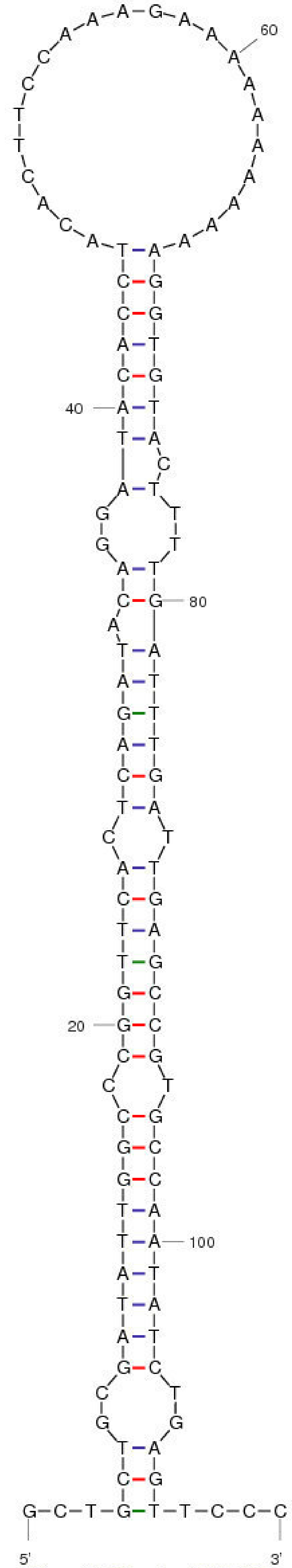

dG = -42.7 vvi-miRC171n

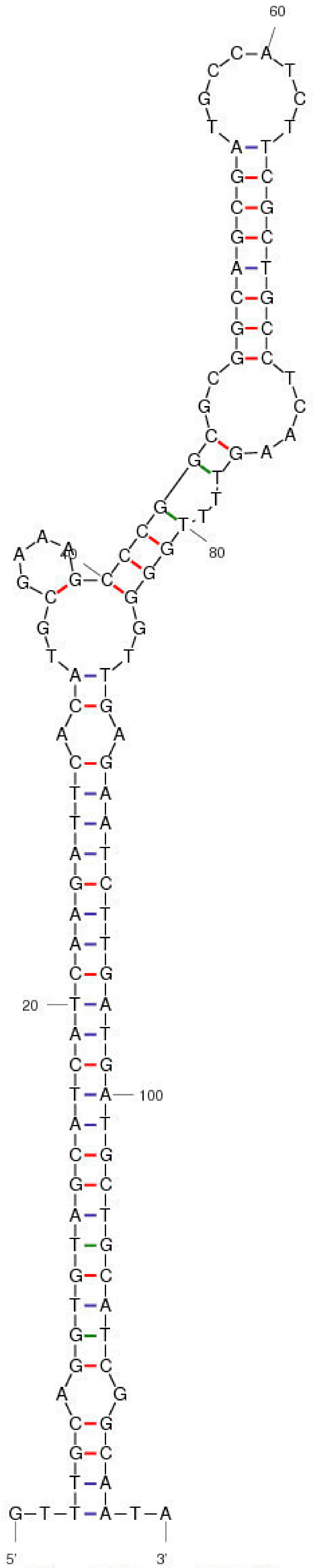

dG = -61.3 vvi-miRC172e

Output of sir_graph (©)
mfold_util 4.7

Created Tue Aug 30 08:08:14 2016

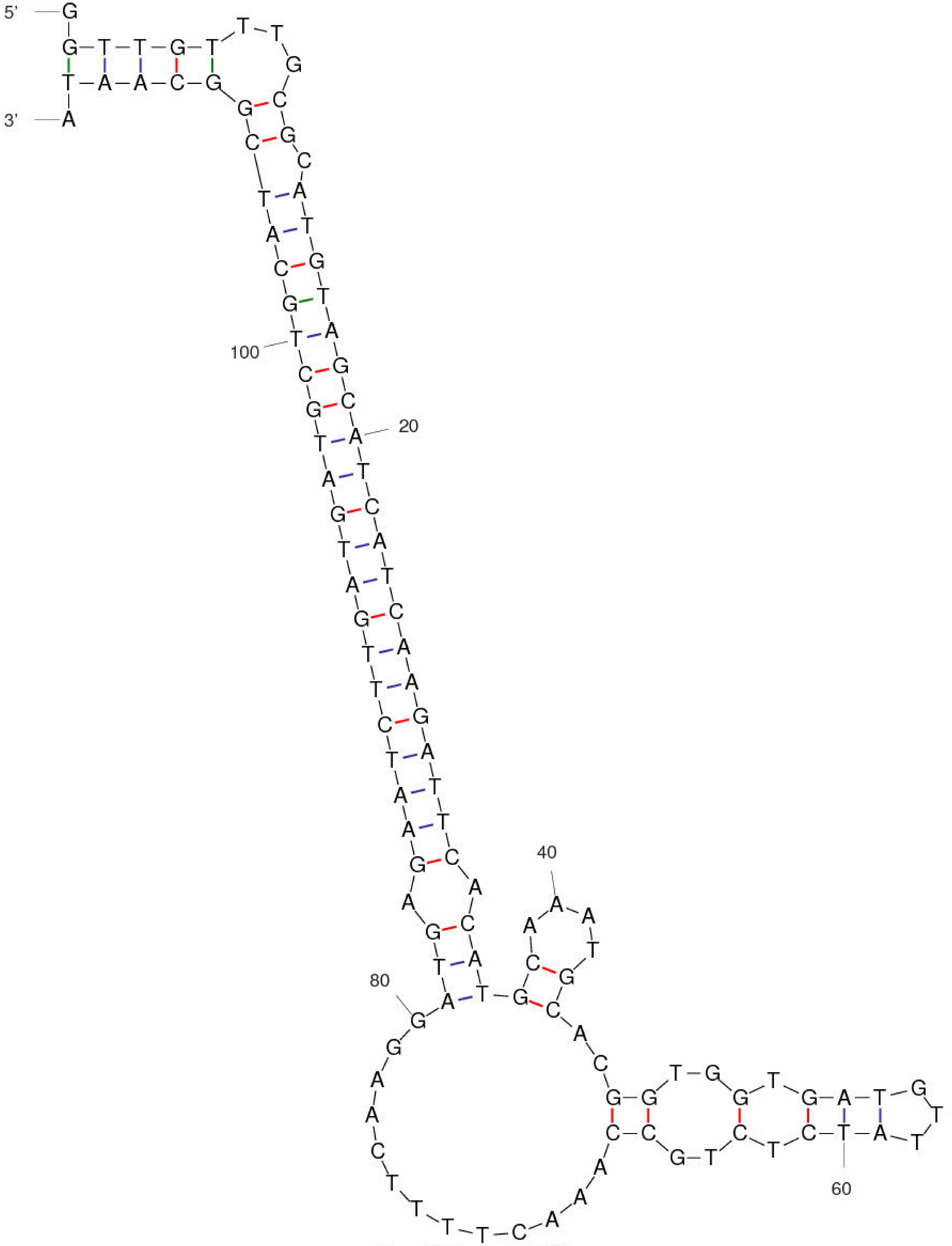

dG = -46.5 vvi-miRC172g

Output of sir_graph (©)
mfold_util 4.7

Created Tue Aug 30 08:08:14 2016

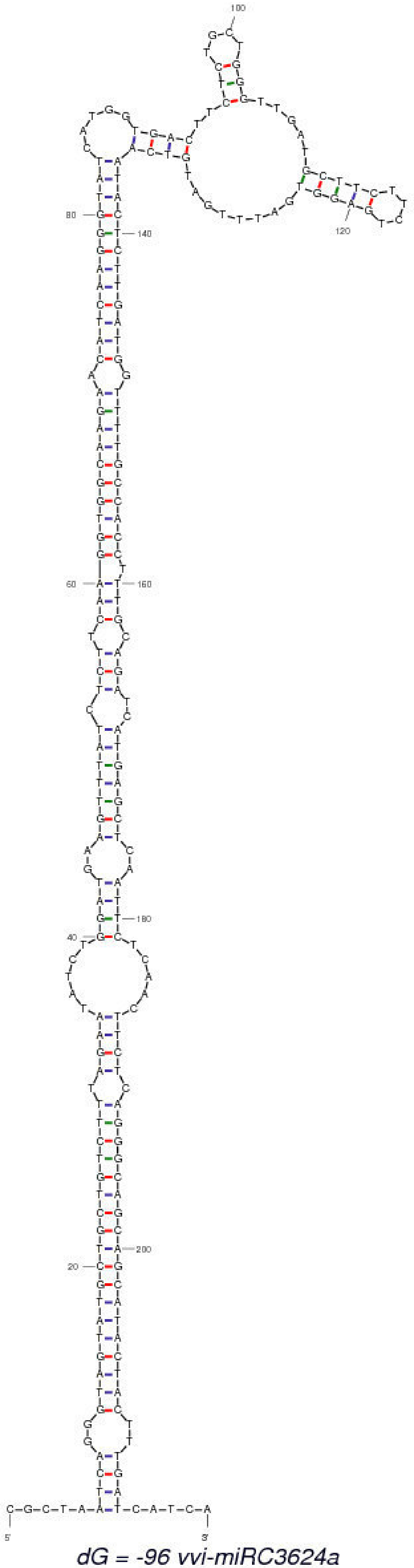

dG = -96 vvi-miRC3624a

Output of sir_graph (©)
mfold_util 4.7

Created Tue Aug 30 08:08:15 2016

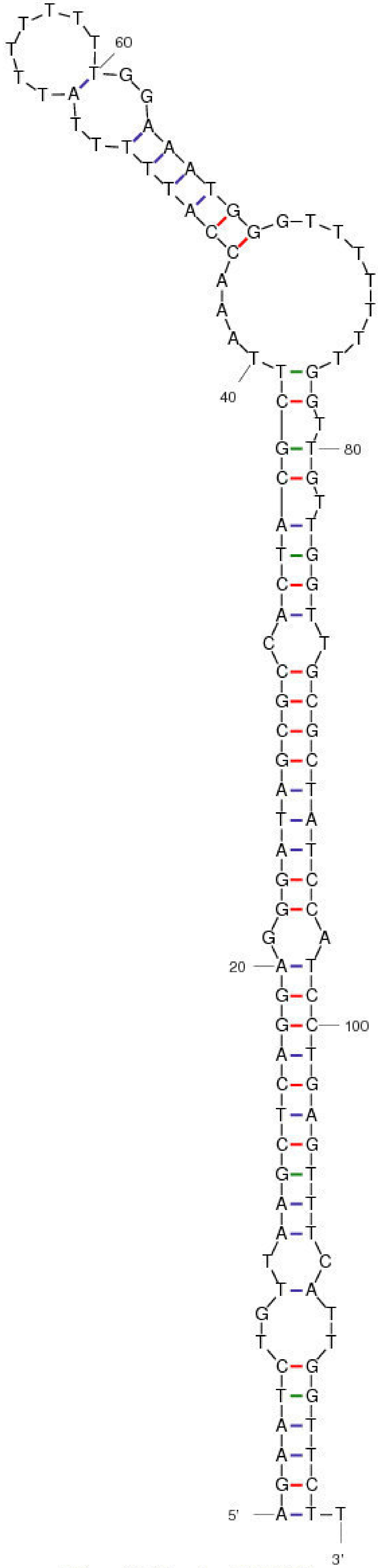

dG = -48.6 vvi-miRC390a

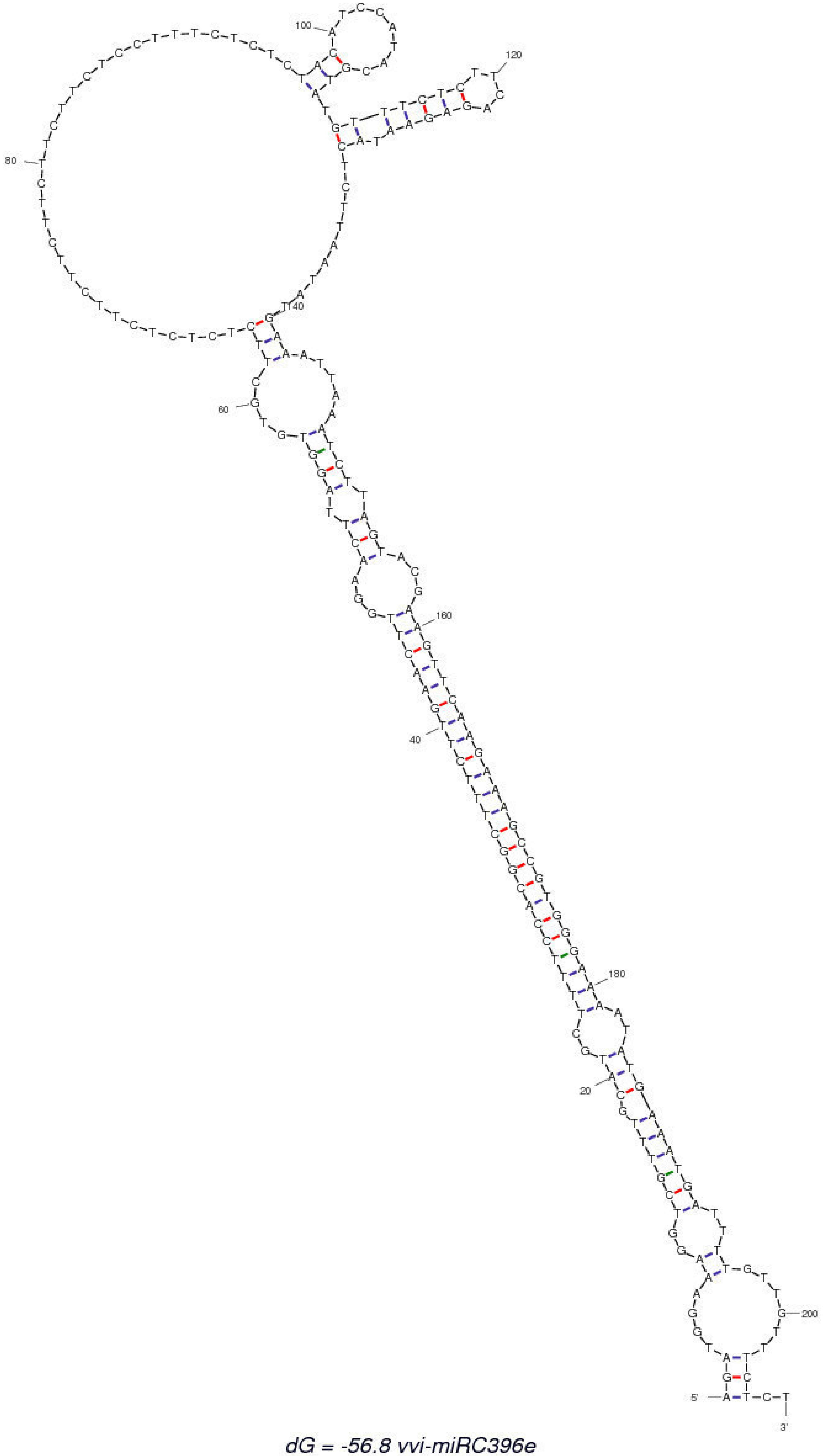

dG = -56.8 vvi-miRC396e

Output of sir_graph (©)
mfold_util 4.7

Created Tue Aug 30 08:08:15 2016

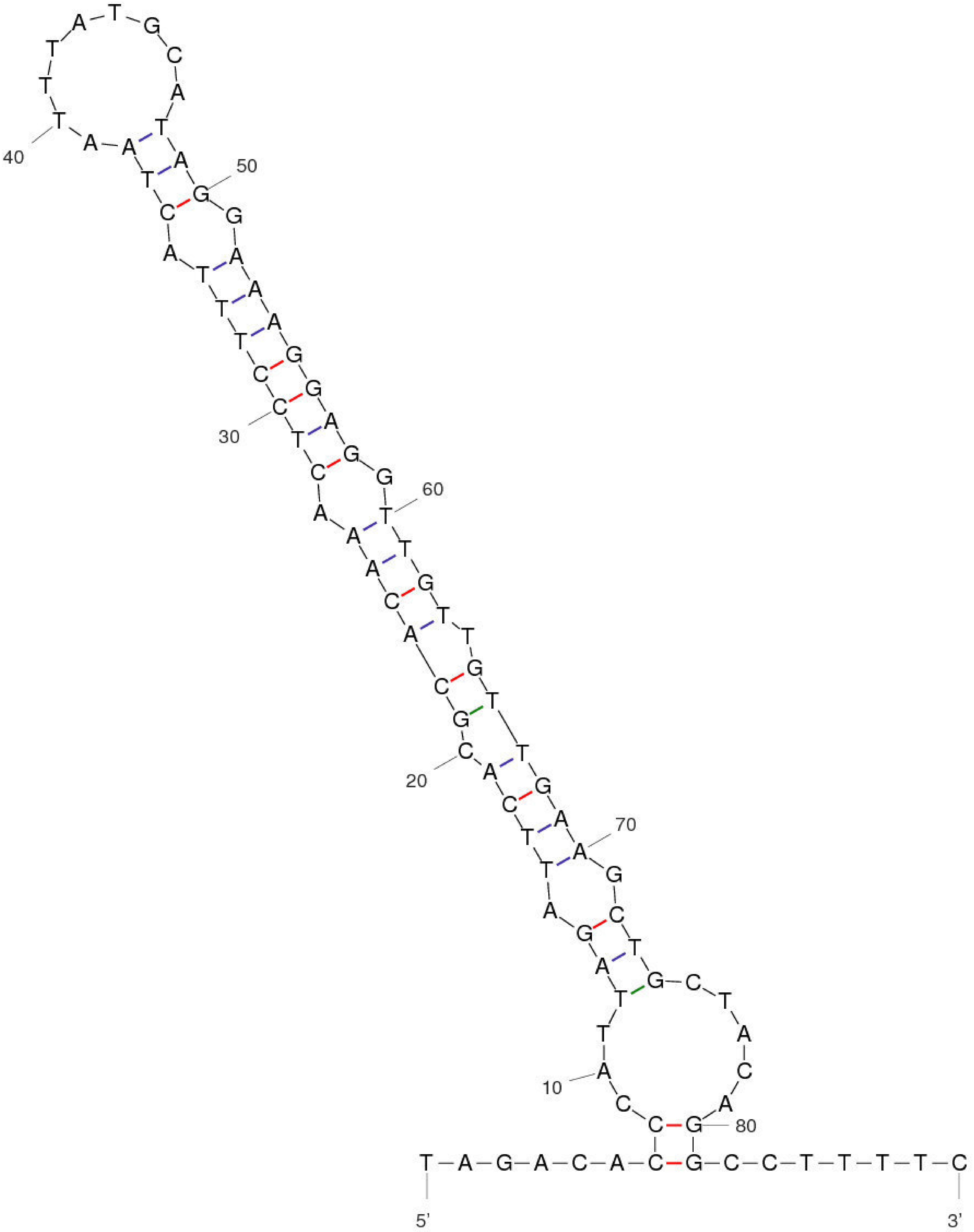

dG = -17.4 vvi-miRC403g

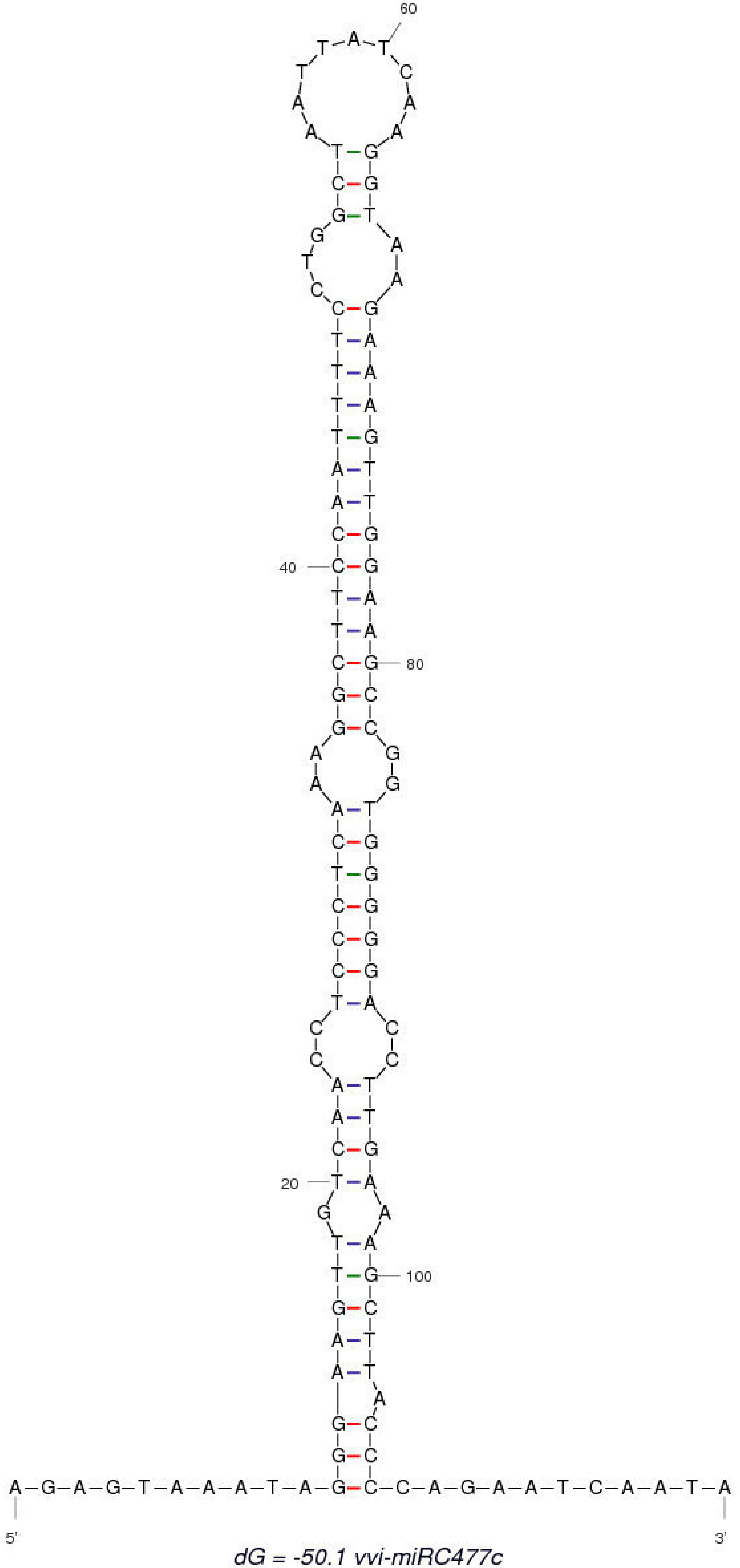

dG = -50.1 vvi-miRC477c

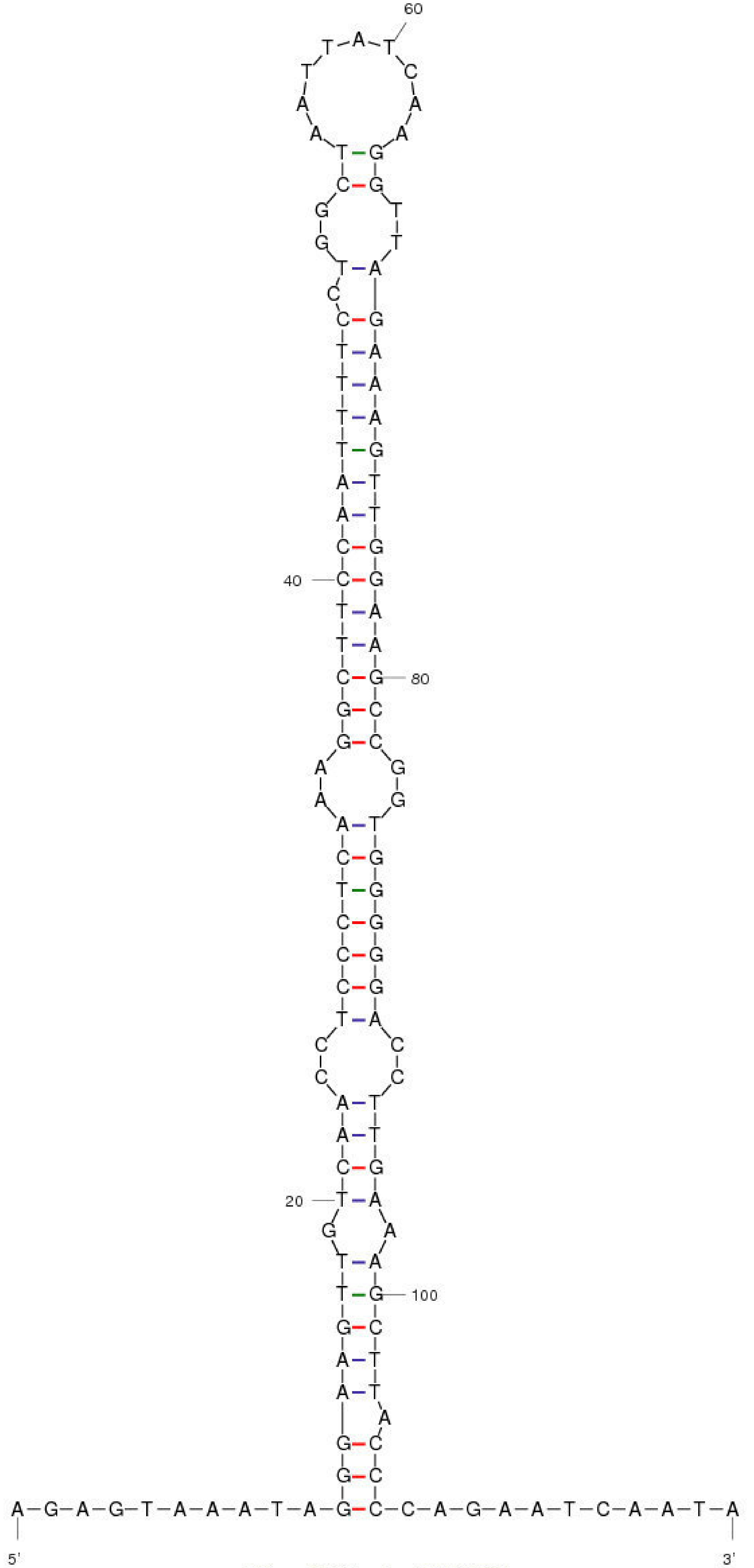

dG = -50.4 vvi-miRC477i

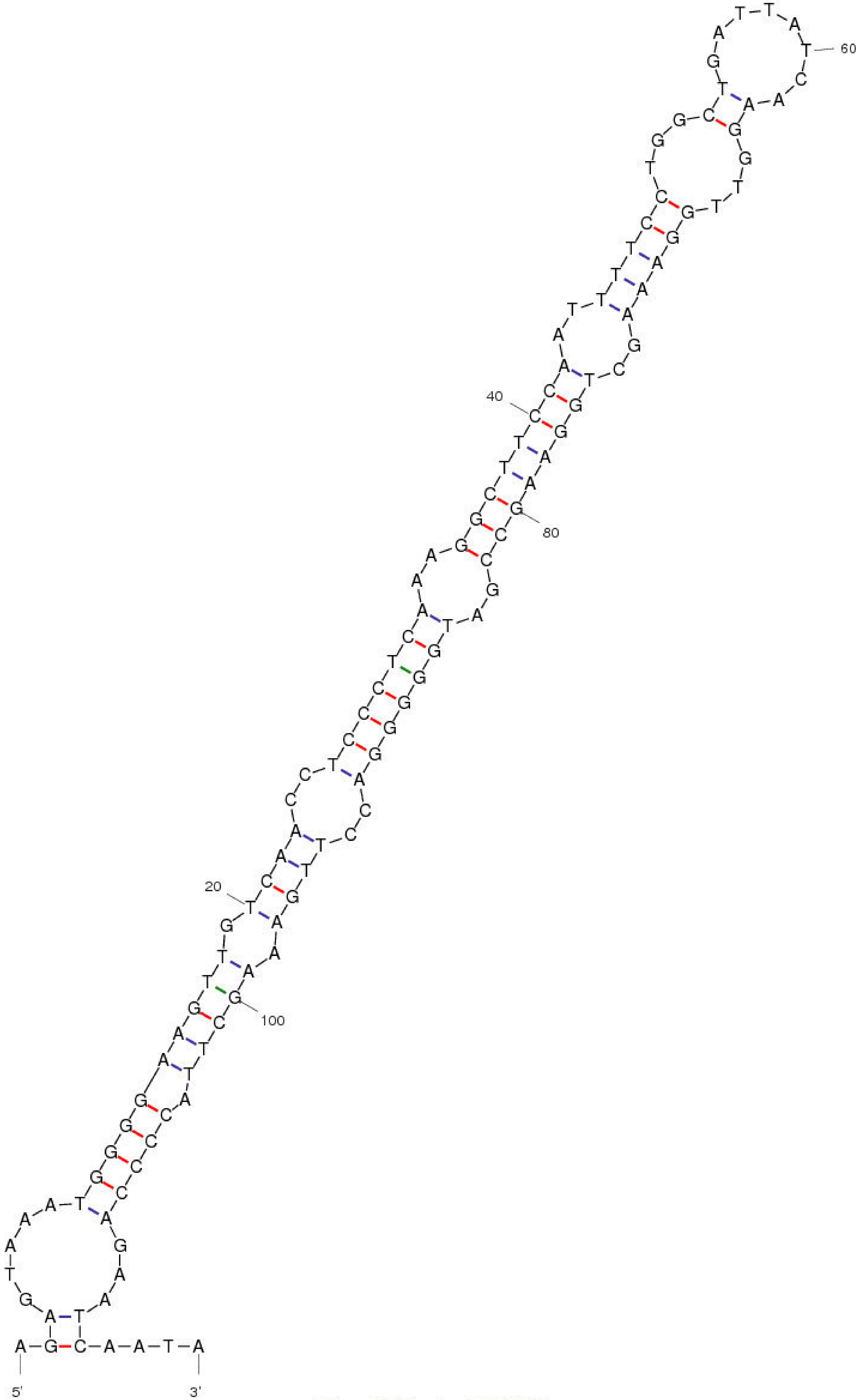

dG = -53.9 vvi-miRC477j

Output of sir_graph (©)
mfold_util 4.7

Created Tue Aug 30 08:08:16 2016

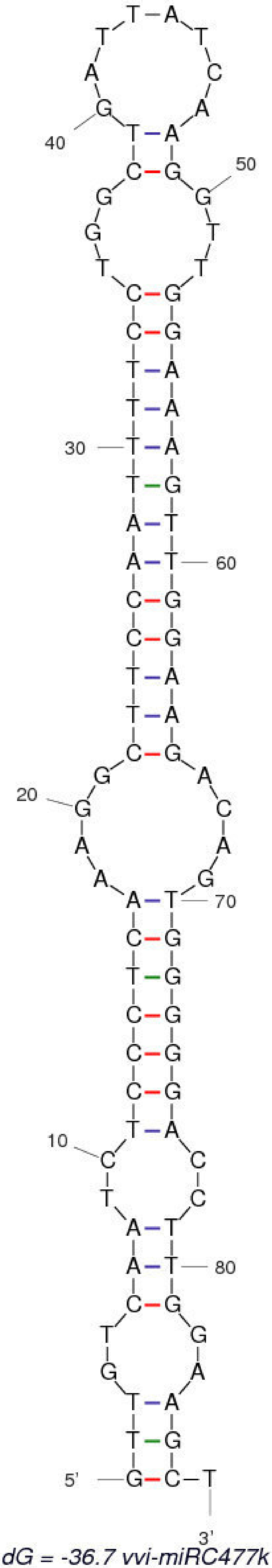

dG = -36.7 vvi-miRC477k

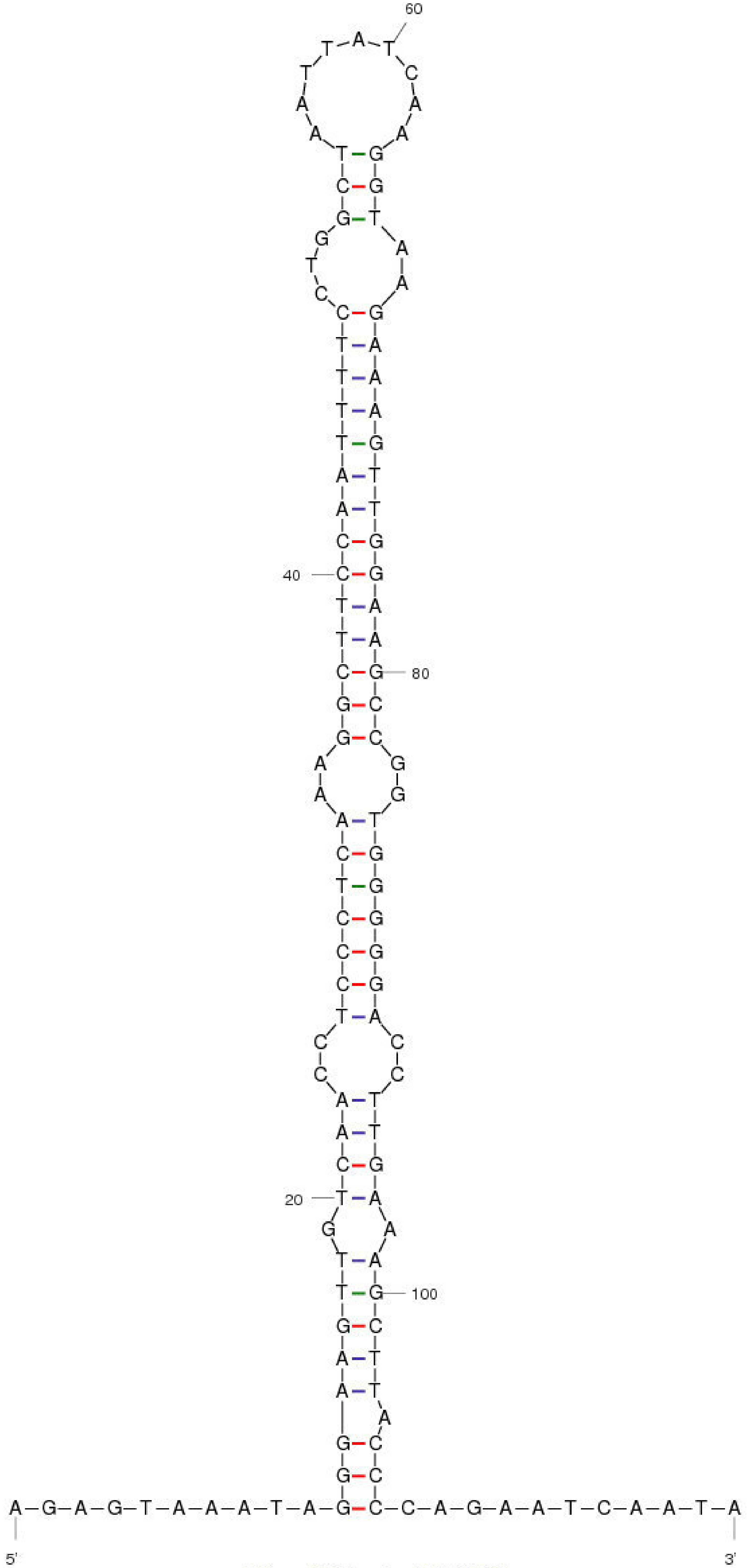

dG = -50.1 vvi-miRC477l

Output of sir_graph (©)
mfold_util 4.7

Created Tue Aug 30 08:08:16 2016

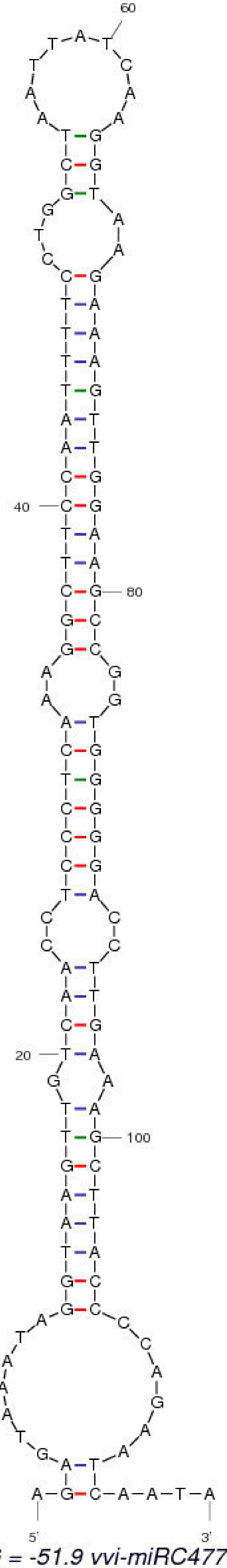

dG = -51.9 vvi-miRC477m

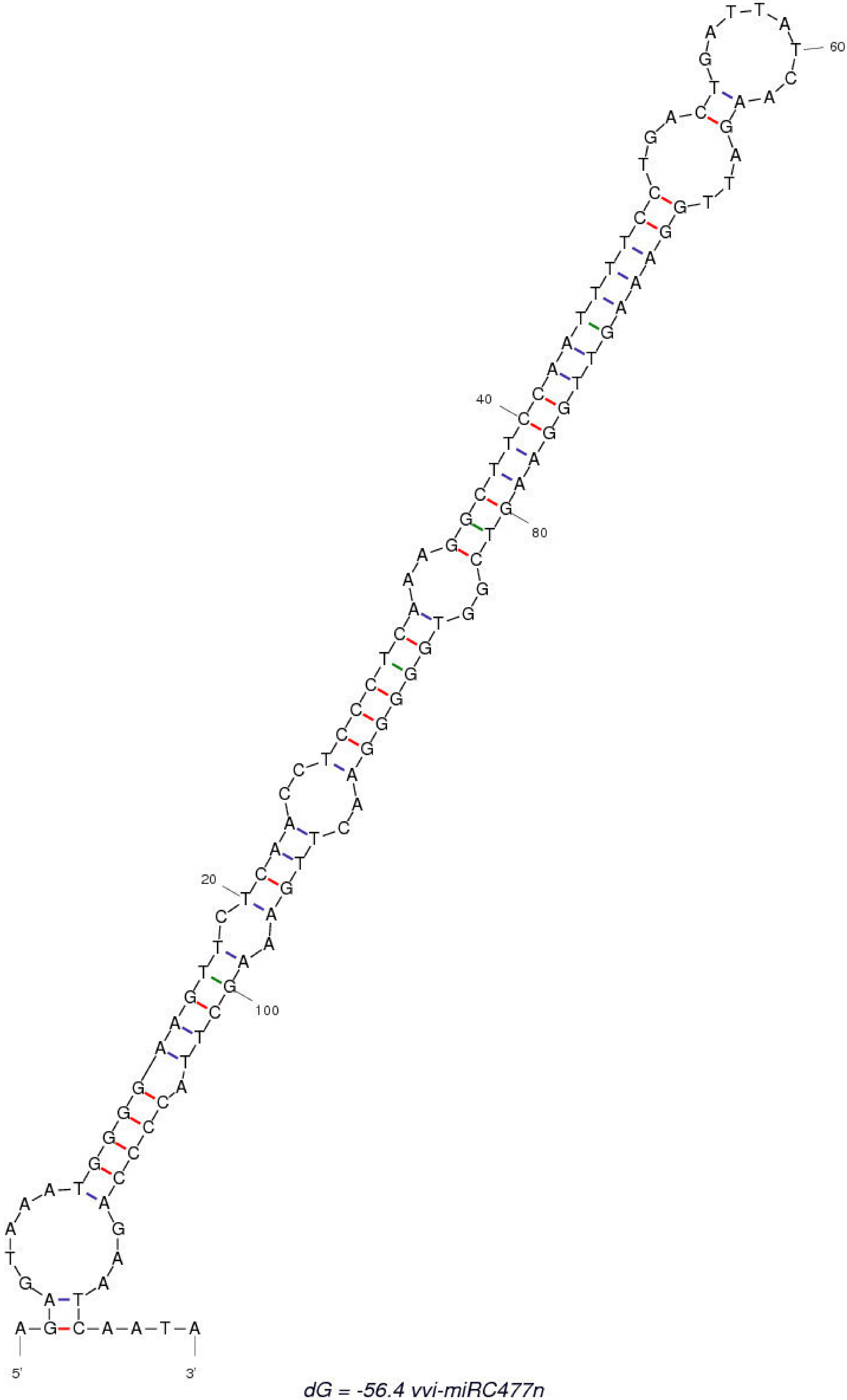

dG = -56.4 vvi-miRC477n

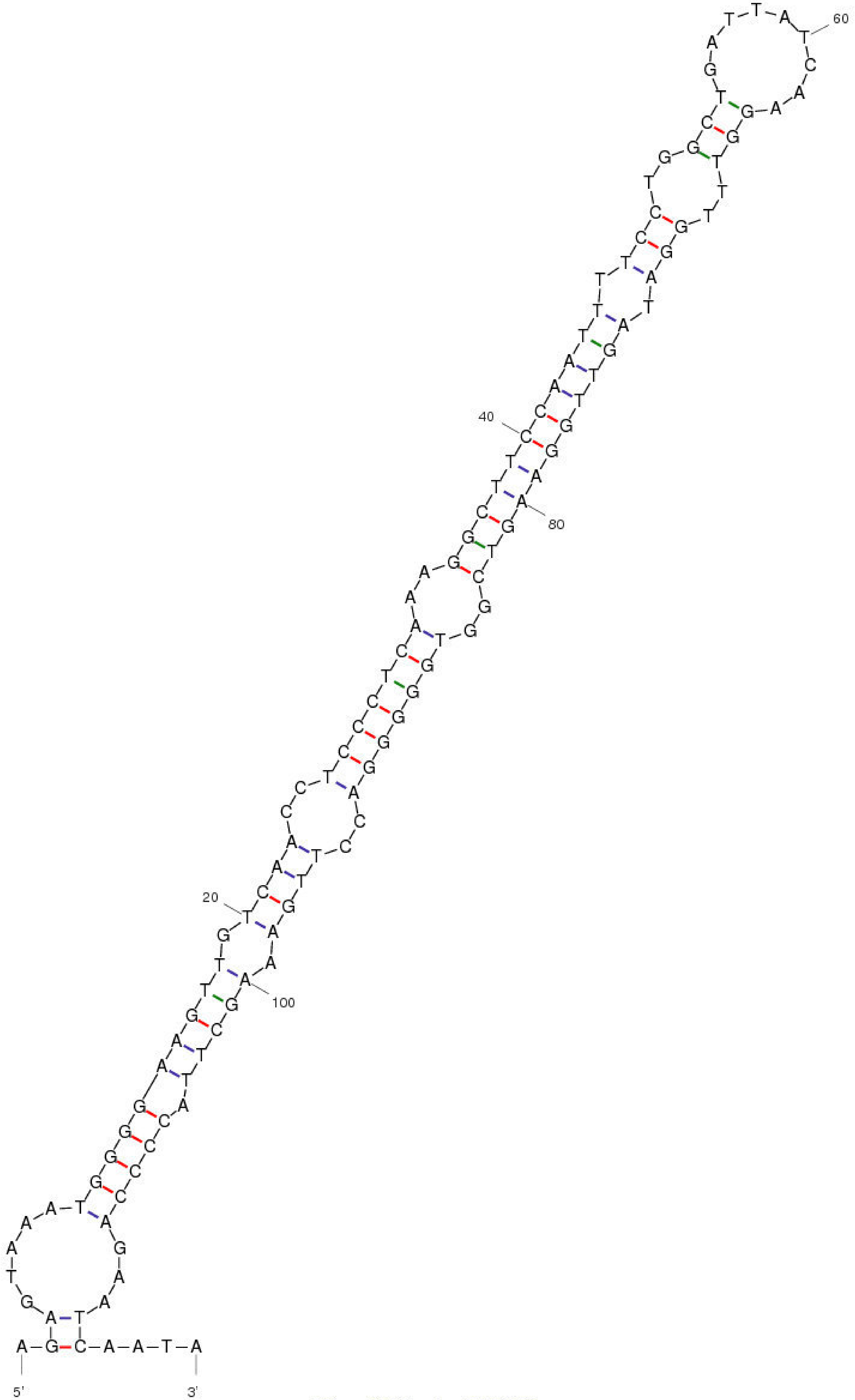

dG = -55.4 vvi-miRC477o

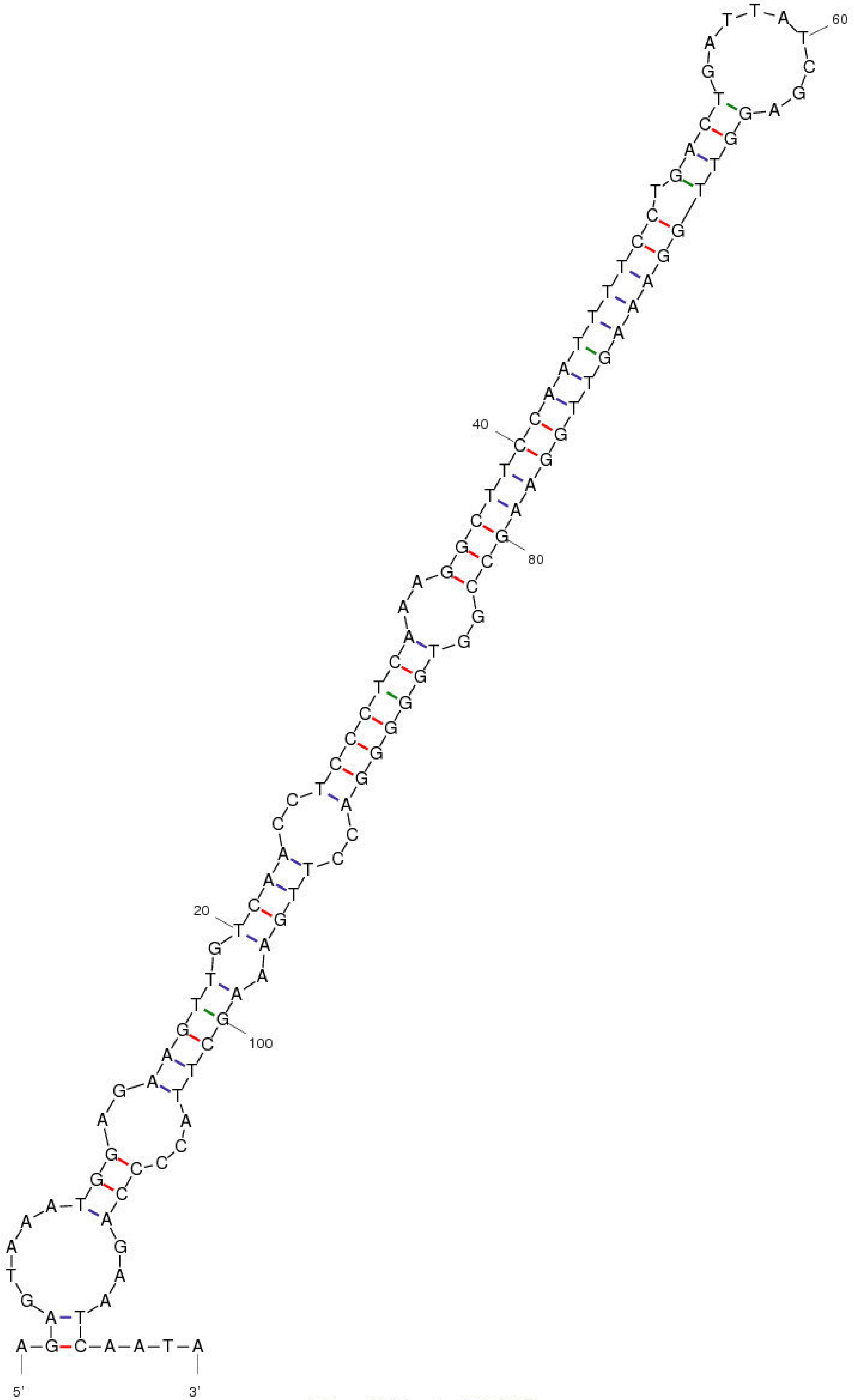

dG = -53.1 vvi-miRC477p

Output of sir_graph (©)
mfold_util 4.7

Created Tue Aug 30 08:08:17 2016

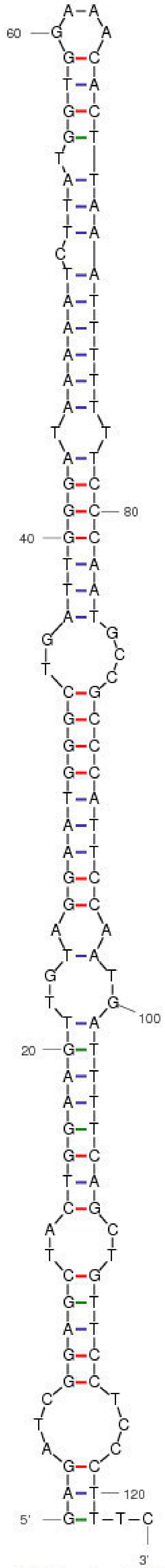

dG = -56.2 vvi-miRC482a

Output of sir_graph (©)
mfold_util 4.7

Created Tue Aug 30 08:08:17 2016

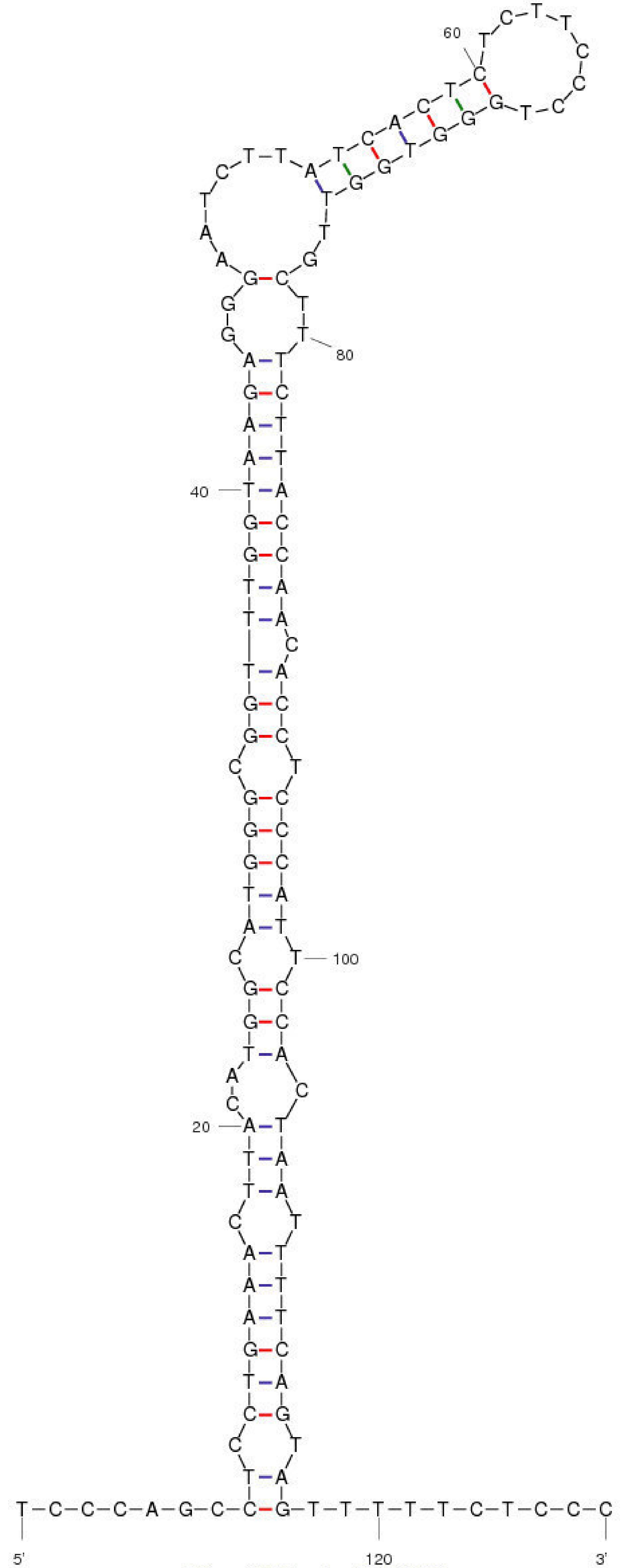

dG = -45.2 vvi-miRC482b

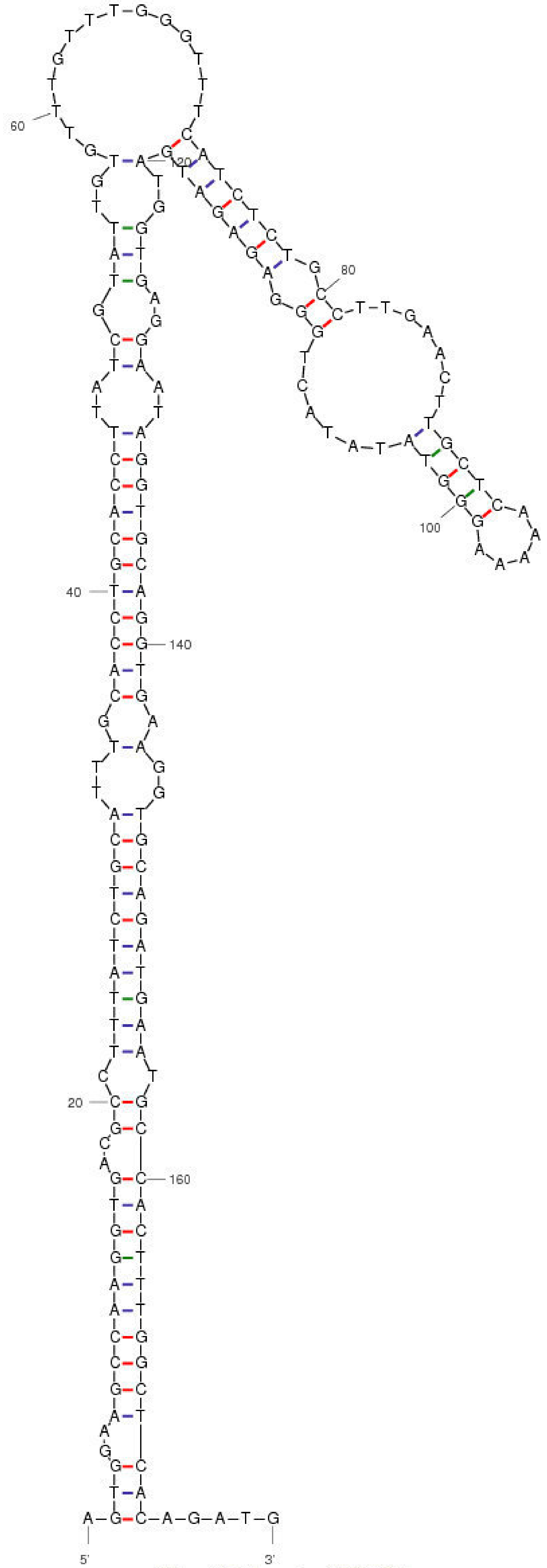

dG = -76.2 vvi-miRC530

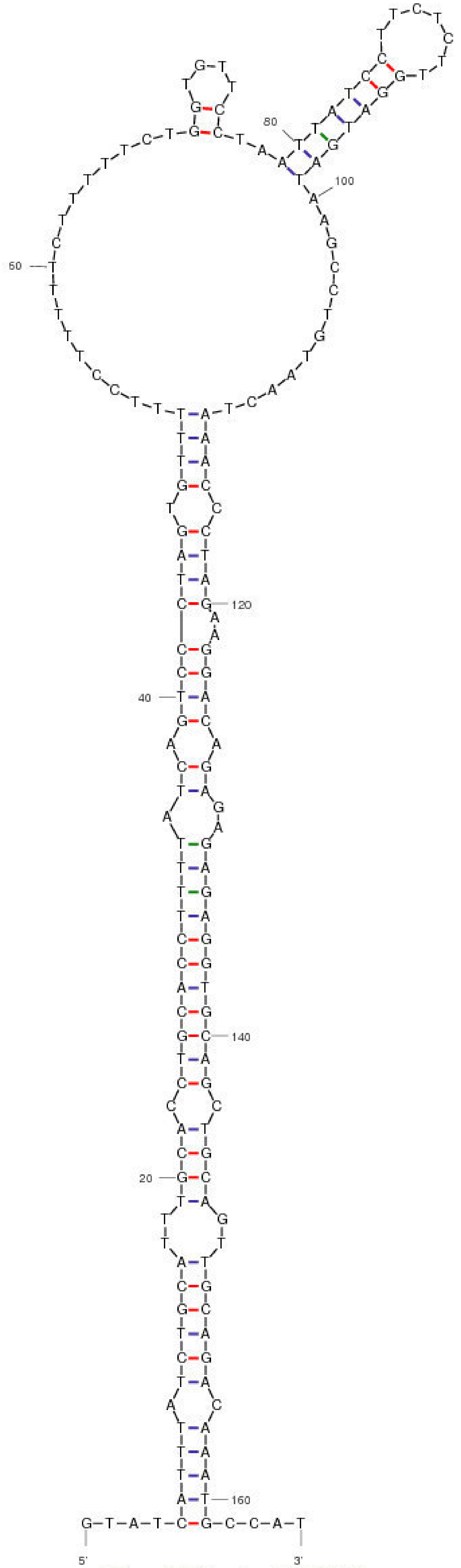

dG = -56.4 vvi-miRC530b

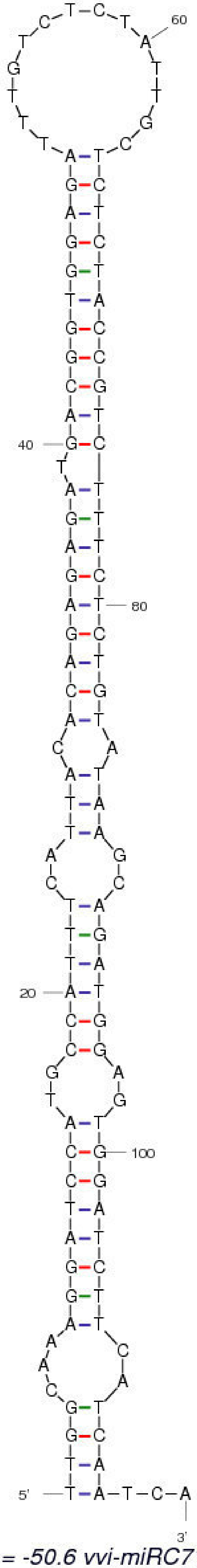

dG = -50.6 vvi-miRC7122

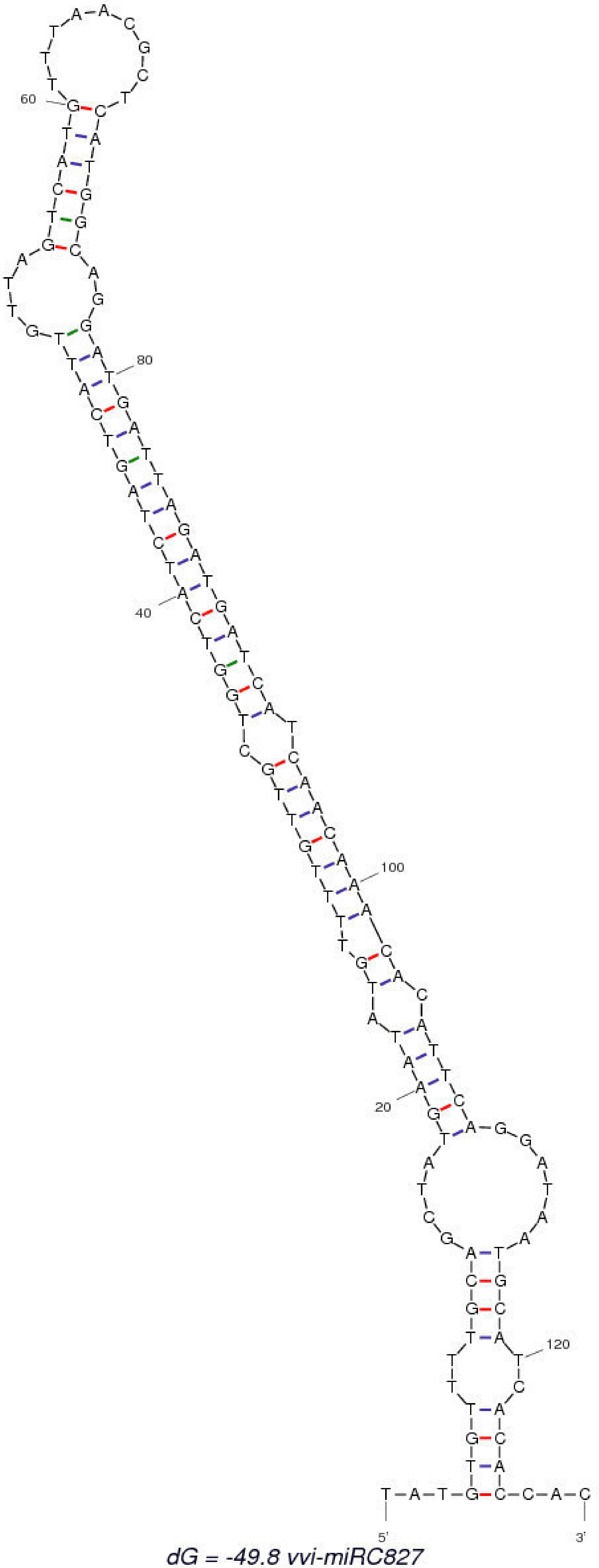

dG = -49.8 vvi-miRC827

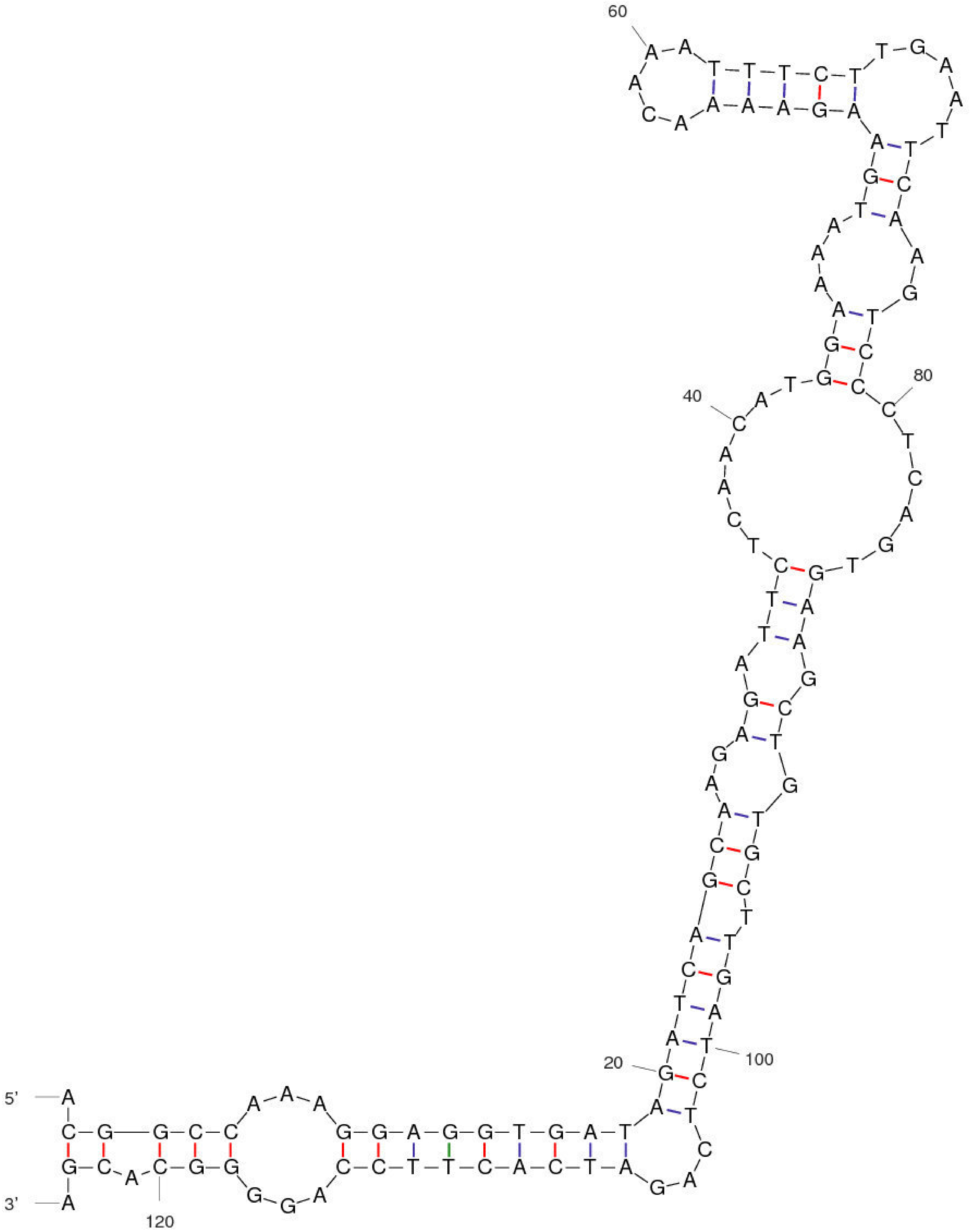

dG = -37.7 grape-m0642

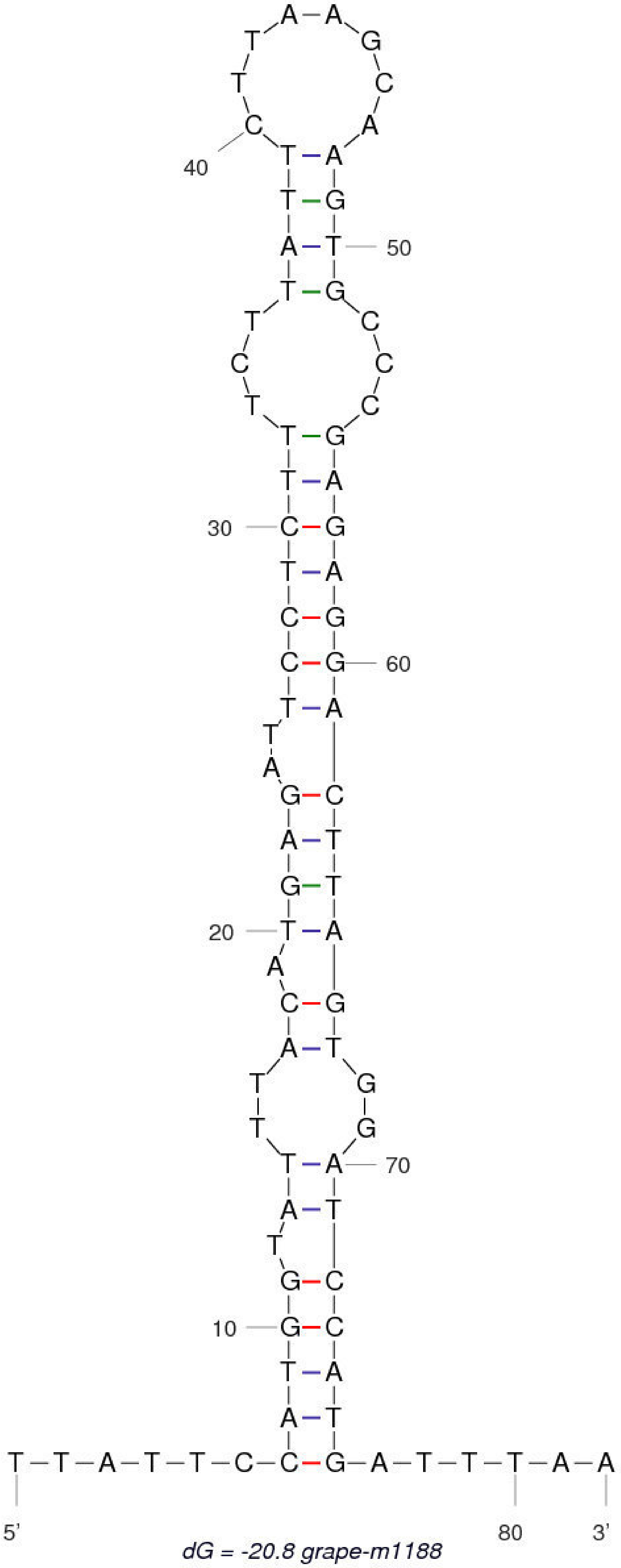

dG = -20.8 grape-m1188

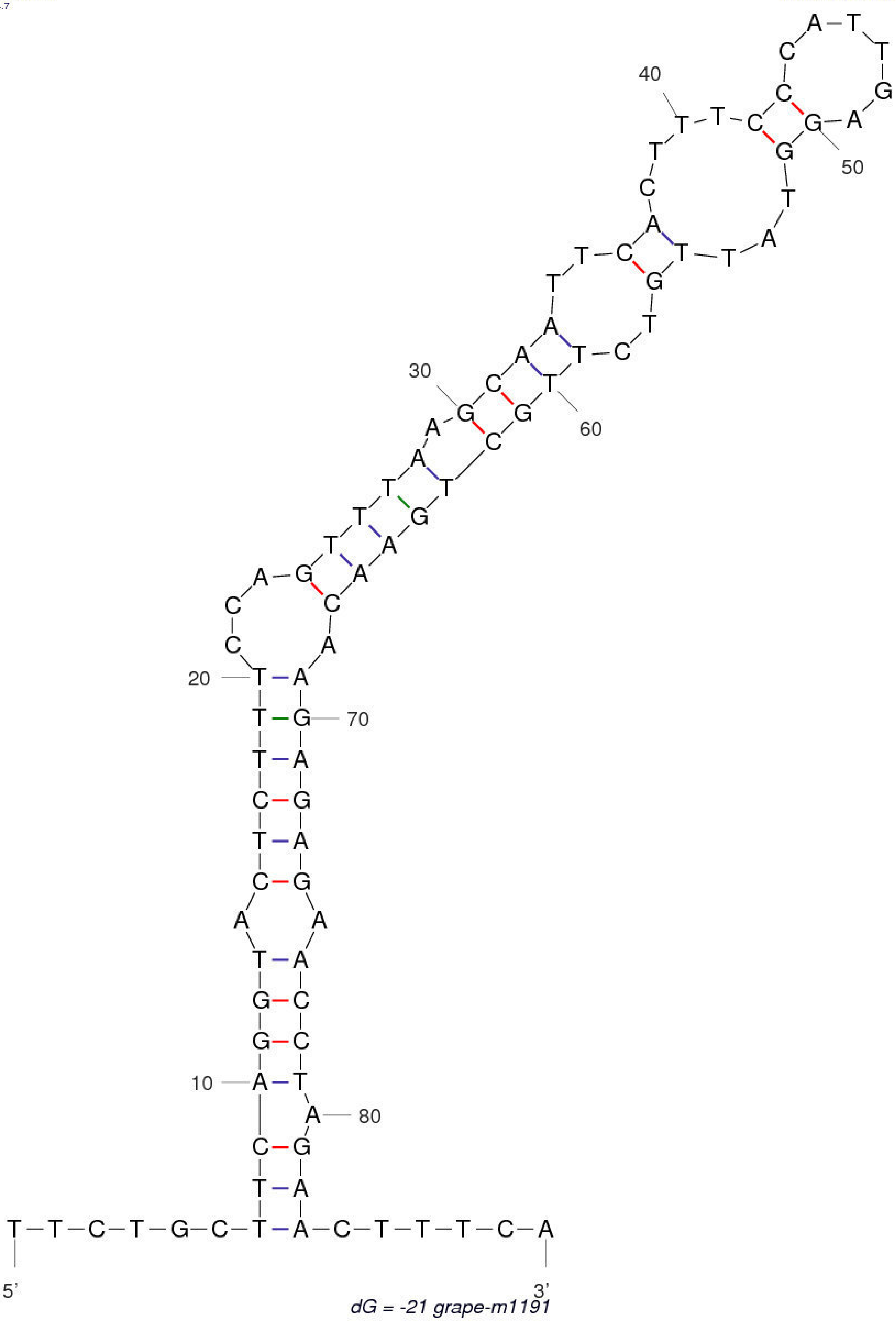

dG = -21 grape-m1191

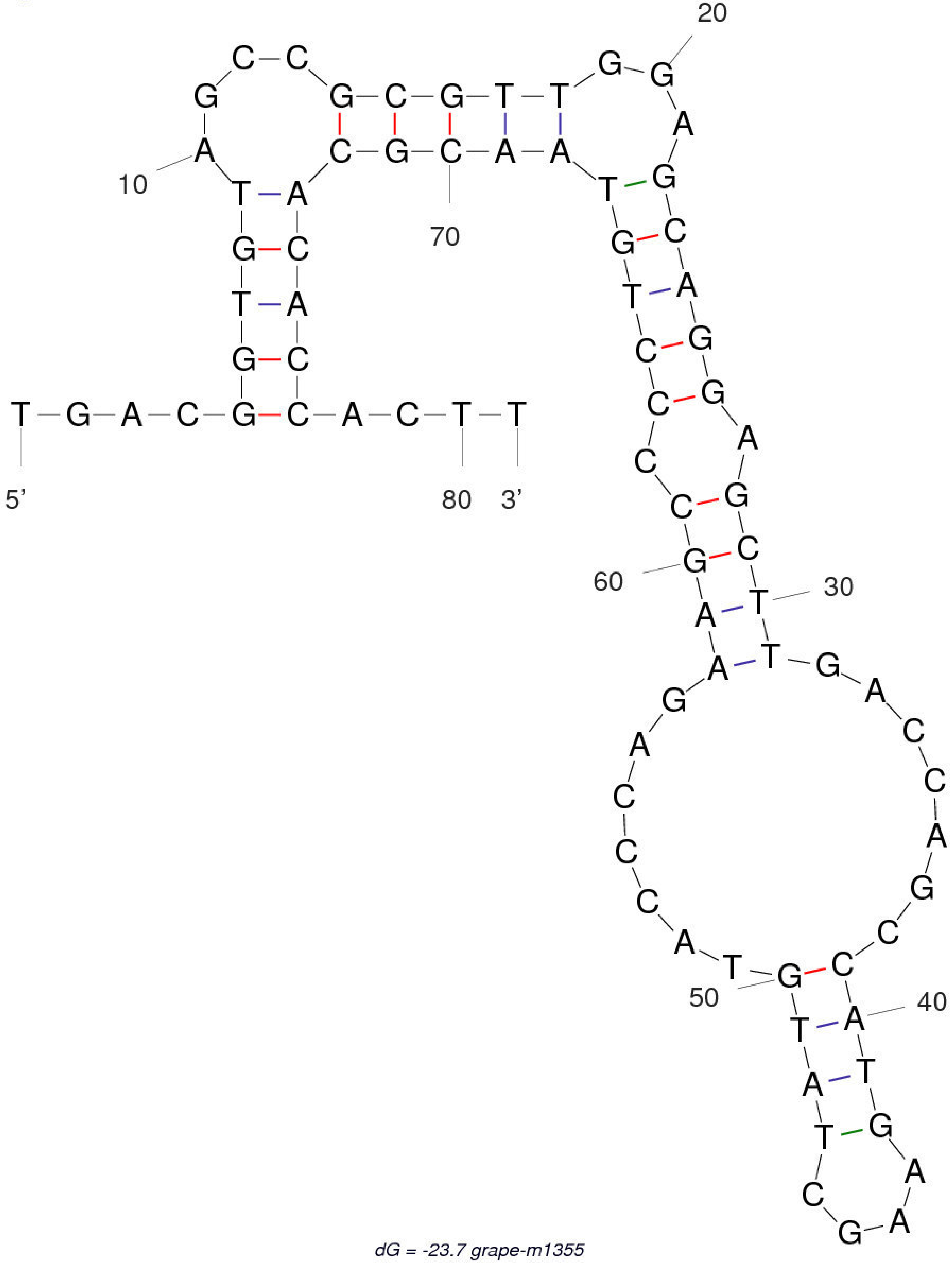

dG = -23.7 grape-m1355

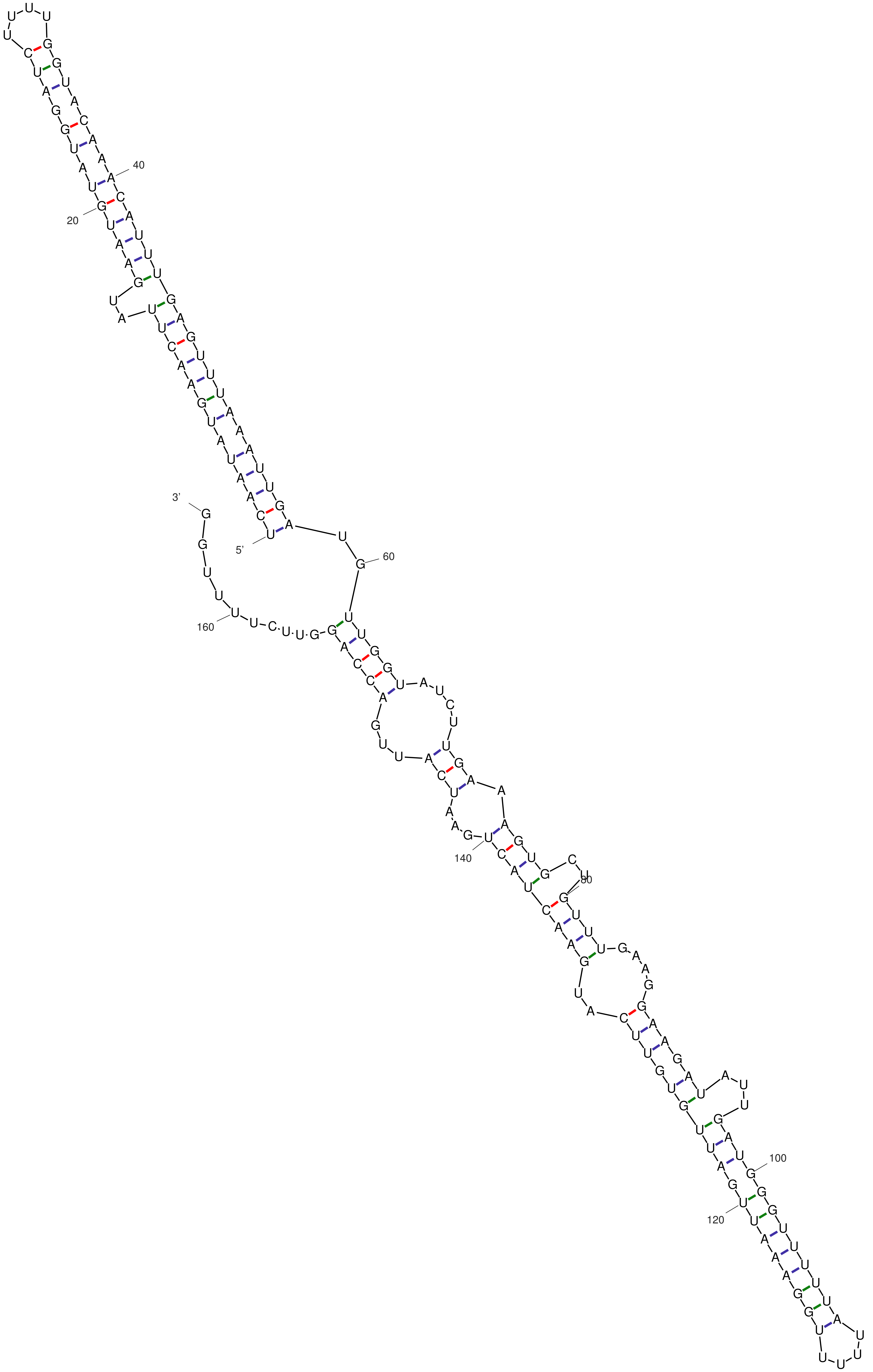

dG = -32.40 [Initially -32.40] grape-m1517

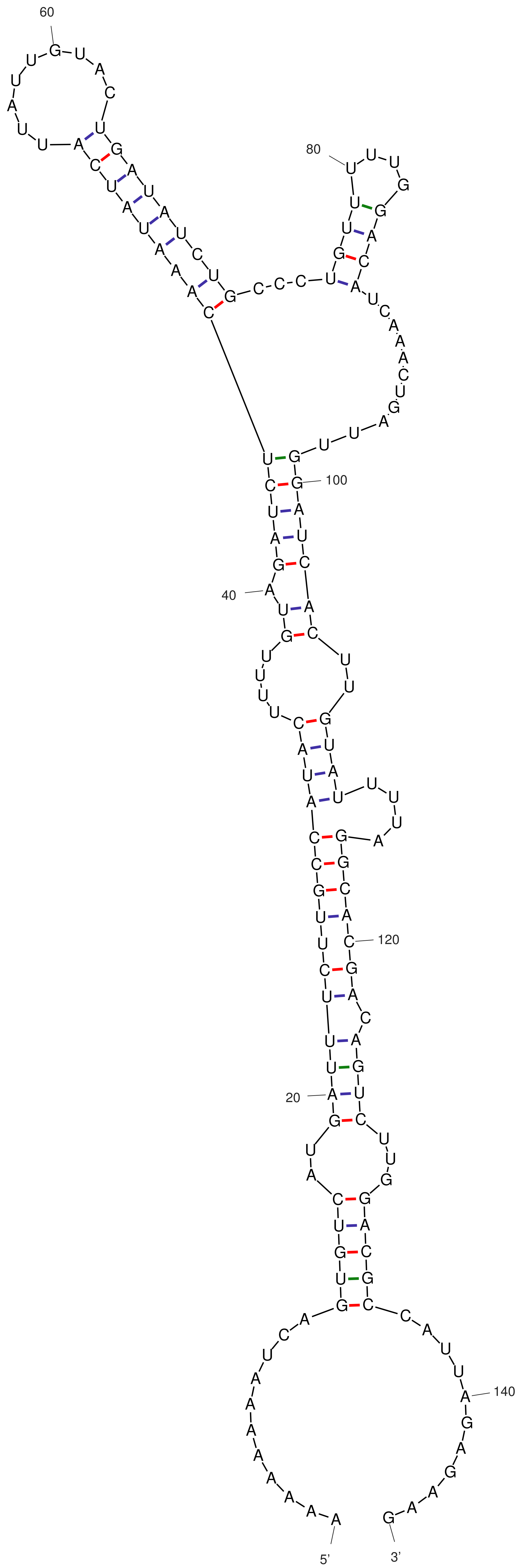

dG = -24.37 [Initially -26.60] grape-m1577

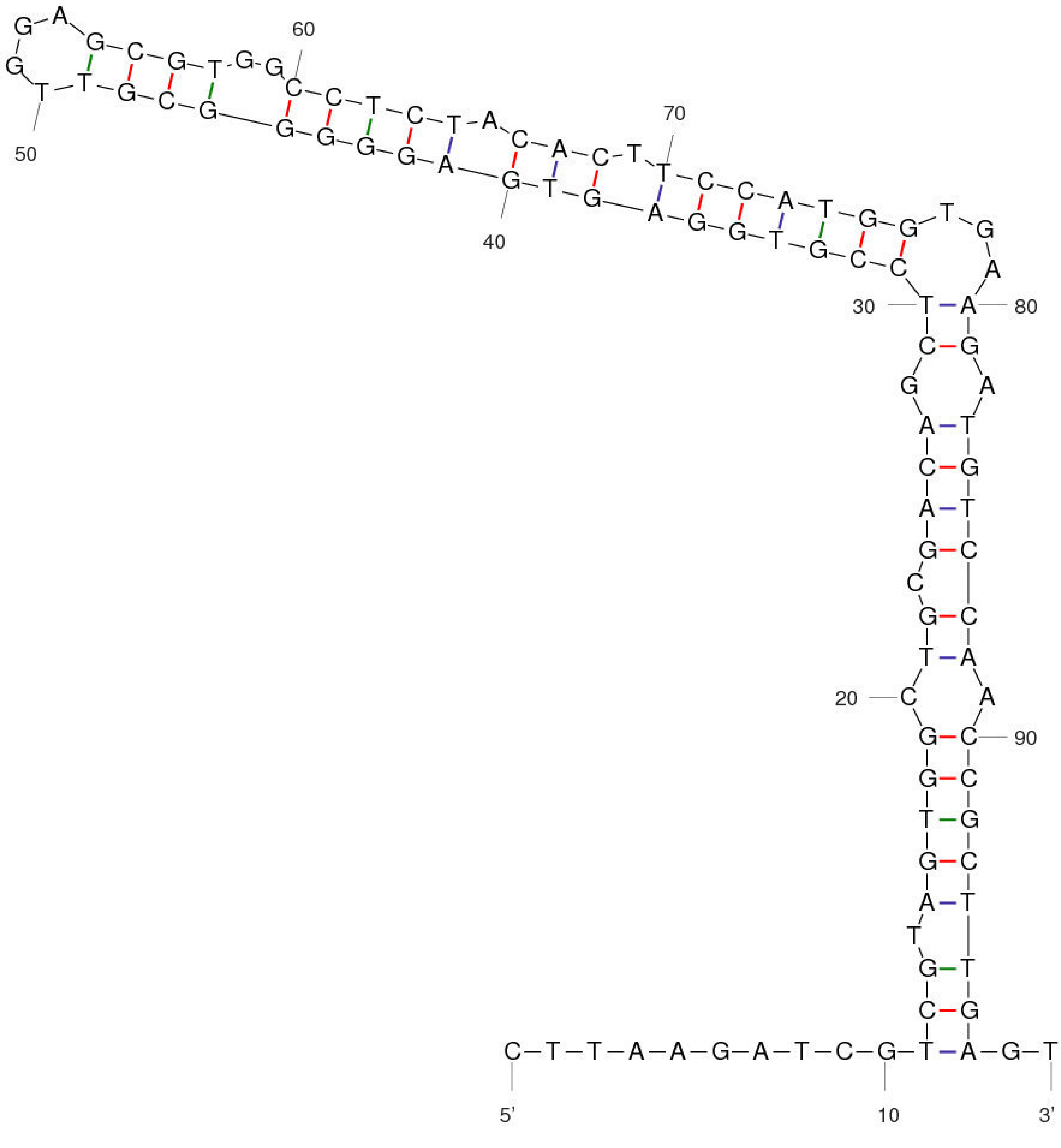

dG = -38.6 grape-m1738

Supplementary Figure 3: miRNA secondary structures of all (known and novel) *Vitis vinifera* miRNAs identified in Cabernet Sauvignon and Sangiovese derived small RNA libraries. Structures have been produced using the Mfold web server (Zuker, 2003), with default settings. For each miRNA, free energy (dG) is given. Mature miRNA sequence for each precursor can be retrieved in table 2 and 3 (main text).

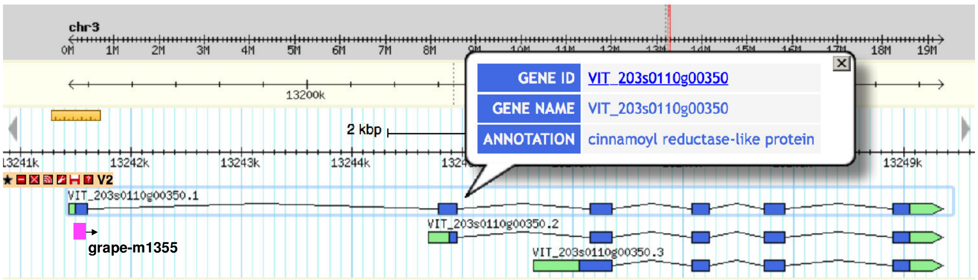

Supplementary Figure 4: Representation of the genomic region where the novel miRNA candidate grape-m1355 maps together with its target (VIT_203s0110g00350.1), and other 2 isoforms of the same target gene.

1.1 Supplementary Tables

Supplementary Table 1. Summary statistics of small RNA libraries. Sequencing was done as a pool of 6 libraries using barcode indexes.

Library[a]	Total raw reads	Total trimmed and chopped reads [b]	Genome matched reads[c]	Distinct genome matched reads[d]	Norm. Base Value
Mont_CS_ps_1	9,043,902	6,003,656	3,079,454	697,685	4M
Mont_CS_ps_2	9,237,934	2,275,836	1,310,311	118,529	4M
Mont_CS_bc_1	7,039,403	4,005,087	1,945,523	250,602	4M
Mont_CS_bc_2	10,416,121	3,245,406	1,517,101	296,213	4M
Mont_CS_19_1	12,627,603	4,816,749	1,932,814	197,822	4M
Mont_CS_19_2	11,650,542	6,832,315	3,302,841	412,701	4M
Mont_CS_hv_1	15,850,494	9,261,043	4,409,822	358,703	4M
Mont_CS_hv_2	23,644,910	4,931,988	2,104,335	138,400	4M
Mont_SG_ps_1	14,142,412	9,920,021	4,730,024	681,265	4M
Mont_SG_ps_2	10,608,981	2,153,646	929,012	180,467	4M
Mont_SG_bc_1	5,853,000	2,366,249	1,243,075	115,569	4M
Mont_SG_bc_2	13,129,001	2,316,461	967,698	121,284	4M
Mont_SG_19_1	5,378,668	3,952,750	2,021,593	241,825	4M
Mont_SG_19_2	5,200,023	2,710,081	984,885	168,108	4M
Mont_SG_hv_1	4,188,242	1,429,022	653,748	73,128	4M
Mont_SG_hv_2	21,488,514	15,977,470	8,134,871	598,594	4M
Bol_CS_ps_1	20,596,628	11,489,167	5,888,470	439,038	4M
Bol_CS_ps_2	15,345,813	12,419,568	6,468,871	1,137,809	4M

Bol_CS_bc_1	12,860,885	9,007,947	4,459,380	422,374	4M
Bol_CS_bc_2	10,062,432	8,245,210	4,432,751	701,773	4M
Bol_CS_19_1	22,379,483	11,433,286	5,424,968	235,174	4M
Bol_CS_19_2	8,792,693	5,766,958	2,846,597	302,798	4M
Bol_CS_hv_1	19,231,501	11,063,113	5,141,666	280,752	4M
Bol_CS_hv_2	11,383,442	6,650,487	3,115,091	336,957	4M
Bol_SG_ps_1	10,667,447	4,588,690	2,278,942	377,985	4M
Bol_SG_ps_2	12,708,803	7,962,286	3,358,152	827,881	4M
Bol_SG_bc_1	17,462,530	10,484,935	5,261,561	587,438	4M
Bol_SG_bc_2	35,741,881	11,126,627	7,140,324	118,071	4M
Bol_SG_19_1	16,493,965	7,234,994	3,399,949	225,402	4M
Bol_SG_19_2	14,478,682	3,703,632	1,705,007	157,304	4M
Bol_SG_hv_1	19,933,813	7,109,871	3,565,679	308,198	4M
Bol_SG_hv_2	5,167,853	3,303,106	1,535,631	177,651	4M
Ric_CS_ps_1	13,090,297	5,896,078	3,074,278	557,681	4M
Ric_CS_ps_2	10,427,663	8,392,862	3,860,244	864,982	4M
Ric_CS_bc_1	8,931,342	3,001,192	1,630,601	256,244	4M
Ric_CS_bc_2	5,641,015	2,191,257	970,282	116,796	4M
Ric_CS_19_1	11,922,030	6,153,194	2,836,288	346,934	4M
Ric_CS_19_2	61,937,671	48,403,939	21,444,997	1,151,918	4M
Ric_CS_hv_1	7,164,050	4,111,362	1,829,279	233,817	4M
Ric_CS_hv_2	45,663,782	32,514,347	14,723,974	974,003	4M
Ric_SG_ps_1	28,244,987	15,557,630	6,851,500	1,057,718	4M
Ric_SG_ps_2	35,201,415	30,300,328	13,707,899	1,351,894	4M
Ric_SG_bc_1	6,950,539	2,884,411	1,466,368	297,029	4M
Ric_SG_bc_2	13,822,016	9,139,817	4,521,724	551,155	4M
Ric_SG_19_1	13,464,075	4,448,747	2,127,072	348,519	4M
Ric_SG_19_2	13,540,517	10,662,287	4,763,877	492,888	4M
Ric_SG_hv_1	14,837,861	3,916,688	2,180,762	164,483	4M
Ric_SG_hv_2	28,373,334	14,549,095	8,673,659	265,147	4M
Summed Total	752,020,195	415,910,891	199,952,950	20,318,708	

[a]The description of small RNA libraries code are shown in Table 1.

[b] total sequences from 18 to 34 nt after trimming the adaptors

[c] Total number of sequences ranging from 18 to 34 nt which perfectly matched to the Grapevine genomic sequence, excluding sequences matched to structural RNAs (t/r/sn/snoRNAs) (GRAPE_IGGP12Xv1).

[d] Number of genome‑matched sequences which are uniquely found within the set, excluding sequences matched to structural RNAs (t/r/sn/snoRNAs).

Supplementary Table 2. Pearson correlation coefficients between library replicates using ad hoc transformed data.

Library	Pearson coefficient
Mont_CS_ps	0.854125568
Mont_CS_bc	0.9548837
Mont_CS_19	0.970957113
Mont_CS_hv	0.957031701
Mont_SG_ps	0.910242175
Mont_SG_bc	0.869691871
Mont_SG_19	0.88316824
Mont_SG_hv	0.929993472
Bol_CS_ps	0.960997846
Bol_CS_bc	0.956185352
Bol_CS_19	0.96055899
Bol_CS_hv	0.931599739
Bol_SG_ps	0.940108049
Bol_SG_bc	0.79060224
Bol_SG_19	0.918562473
Bol_SG_hv	0.970880884
Ric_CS_ps	0.961428872
Ric_CS_bc	0.923738401
Ric_CS_19	0.967545547
Ric_CS_hv	0.969727901
Ric_SG_ps	0.962939701
Ric_SG_bc	0.954586248
Ric_SG_19	0.946924113
Ric_SG_hv	0.970507578
Average	0.934041157

.

Supplementary Table 3. List of putative targets of known and novel vvi-miRNAs identified using the module *miRferno* of sPARTA software. Only targets with score ≤2.5 are listed**.**

miRNA name	Target gene	Number of target sites	miRNA sequence	Target sequence	Score	Target annotation
grape-m0642-5p	VIT_200s0291g00090	1	GAGAACGACUAGAUAGUGGAG	CUCUUUCUGAUCUAUCACCGC	2	cyclin-related protein hydrolase activity
grape-m1188-3p	VIT_200s0173g00170	1	CUAGGUGAUUCAGGAGAGCCC	GAUCCACUAAGUCCUCUCGGG	0	ribosomal protein s7 structural constituent of ribosome
grape-m1188-3p	VIT_202s0012g03140	1	CUAGGUGAUUCAGGAGAGCCC	GAUCCACUAAGUCCUCUCGGG	0	hypothetical protein
grape-m1191-3p	VIT_205s0029g00750	3	UCCAAGAGAGAACAAGUCGUU	CGGUUCUCUCUUGUUUAACAA	2.5	tmv resistance protein n-like defense response
grape-m1191-3p	VIT_205s0029g00880	1	UCCAAGAGAGAACAAGUCGUU	CGGUUCUCUCUUGUUUAACAA	2.5	tmv resistance protein n-like defense response
grape-m1191-3p	VIT_212s0028g01160	1	UCCAAGAGAGAACAAGUCGUU	GGGUCCUCUCUGGUUCAGCAA	2.5	TT12 - transparent testa dd
grape-m1191-3p	VIT_212s0034g00910	1	UCCAAGAGAGAACAAGUCGUU	AGGUUCUCUCUUAUUCAACAA	2	tmv resistance protein n-like defense response
grape-m1191-3p	VIT_212s0057g01100	3	UCCAAGAGAGAACAAGUCGUU	AGGUUCUCGCUUGUUCAGCAA	1	nudix hydrolase 2-like protein poly-ADP-ribosylation
grape-m1191-3p	VIT_214s0068g00210	1	UCCAAGAGAGAACAAGUCGUU	AGGUUCUCUGUUGUUCAGCCA	2	abc transporter family protein plant-type vacuole
grape-m1355-5p	VIT_203s0110g00350	1	UUCGAGGACGAGGUUGCGCCG	AAGCUCCUGCUCCAACGCGGC	0	cinnamoyl reductase-like protein
grape-m1355-5p	VIT_206s0004g02380	1	UUCGAGGACGAGGUUGCGCCG	AAGCUUCUUCUCCAACGUGGU	2.5	cinnamyl alcohol dehydrogenase coenzyme binding
grape-m1355-5p	VIT_213s0047g00540	1	UUCGAGGACGAGGUUGCGCCG	AAGCUCCUUCUCCAACGCGGC	1	bifunctional dihydroflavonol 4-reductase flavanone 4-reductase-like
grape-m1355-5p	VIT_213s0047g00550	1	UUCGAGGACGAGGUUGCGCCG	AAGCUCCUGCUCCAACACCGU	2.5	bifunctional dihydroflavonol 4-reductase flavanone 4-reductase-like nucleic acid binding
grape-m1355-5p	VIT_213s0047g00700	1	UUCGAGGACGAGGUUGCGCCG	AAGCUCCUGCUCCAAUGCGGC	0.5	bifunctional dihydroflavonol 4-reductase flavanone 4-reductase-like steroid
grape-m1355-5p	VIT_213s0047g00940	1	UUCGAGGACGAGGUUGCGCCG	AAGCUCCUGCUCCAACGCGGC	0	bifunctional dihydroflavonol 4-reductase flavanone 4-reductase-like nucleic acid binding
grape-m1355-5p	VIT_213s0047g00990	1	UUCGAGGACGAGGUUGCGCCG	AAGCUCUUGCUCCAACGCGGC	0.5	bifunctional dihydroflavonol 4-reductase flavanone 4-reductase-like coenzyme binding
grape-m1355-5p	VIT_213s0064g00290	1	UUCGAGGACGAGGUUGCGCCG	AAGCUCCUGCUCCAACGCGGC	0	bifunctional dihydroflavonol 4-reductase flavanone 4-reductase-like nucleic acid binding
grape-m1355-5p	VIT_213s0064g00340	1	UUCGAGGACGAGGUUGCGCCG	AAGCUCCUGCUCCAACGUGGC	0.5	phenylacetaldehyde reductase
grape-m1355-5p	VIT_213s0067g00460	1	UUCGAGGACGAGGUUGCGCCG	AAGCUCCUGCUCCAACGUGGU	1	bifunctional dihydroflavonol 4-reductase flavanone 4-reductase-like nucleic acid binding
grape-m1355-5p	VIT_213s0067g00530	1	UUCGAGGACGAGGUUGCGCCG	AAGCUCCUGCUCCAACGUGGU	1	bifunctional dihydroflavonol 4-reductase flavanone 4-reductase-like nucleic acid binding

miRNA name	Target gene	Number of target sites	miRNA sequence	Target sequence	Score	Target annotation
grape-m1355-5p	VIT_213s0101g00250	1	UUCGAGGACGAGGUUGCGCCG	AAGCUCCUGCUCCAACACCGU	2.5	bifunctional dihydroflavonol 4-reductase flavanone 4-reductase-like para-aminobenzoic acid
grape-m1517-3p	VIT_203s0063g02020	2	GACCAGUUACUAAGUCAUCAA	CUGGGCAAUGCUUCAGUGGUU	2.5	tic62 protein
grape-m1517-3p	VIT_219s0090g01770	1	GACCAGUUACUAAGUCAUCAA	CUGGUGAGUGAUUUAGUGGUU	2.5	hypothetical protein
grape-m1738-5p	VIT_208s0007g08500	1	CCUCGACAGCGUCGGUGAUGCU	GGAGCUGUCGCAGCCACUACGA	0	60s ribosomal export protein nmd3-like cytosol
vvi-miRC169z-5p	VIT_200s0125g00290	1	UCCGUUCAGUAGGAACCGAU	GGGUAAGUUGUCCUUGGCUG	2.5	protein regulation of meristem growth
vvi-miRC169z-5p	VIT_201s0146g00380	2	UCCGUUCAGUAGGAACCGAU	AGGCAAGUCAUCCUUCGCUA	1	litaf-domain-containing protein response to absence of light
vvi-miRC169z-5p	VIT_208s0007g08250	14	UCCGUUCAGUAGGAACCGAU	AGGCAAAUCAUUCUUGGCUU	2.5	nuclear transcription factor y subunit a- regulation of transcription
vvi-miRC169z-5p	VIT_209s0002g01590	3	UCCGUUCAGUAGGAACCGAU	UGGAAAGUCAUCCUUGGCUG	2.5	nuclear transcription factor y subunit a-3 regulation of transcription
vvi-miRC169z-5p	VIT_211s0016g01480	4	UCCGUUCAGUAGGAACCGAU	GGGCAAUUCAUCCUUGGCUU	2.5	nuclear transcription factor y subunit a- regulation of transcription
vvi-miRC169z-5p	VIT_213s0064g00860	3	UCCGUUCAGUAGGAACCGAU	AGGCAAUUCAUUCUUGGCUU	2.5	nuclear transcription factor y subunit a- regulation of transcription
vvi-miRC171j-3p	VIT_202s0154g00400	1	CUAUAACCGUGCCGAGUUAGU	GAUAUUGGCGCGGCUCAAUCA	0.5	gras family transcription factor regulation of transcription
vvi-miRC171j-3p	VIT_204s0023g01380	1	CUAUAACCGUGCCGAGUUAGU	GAUAUUGGCGCGGCUCAAUCA	0.5	gras family transcription factor regulation of shoot system development
vvi-miRC171j-3p	VIT_212s0059g00650	1	CUAUAACCGUGCCGAGUUAGU	GAUAUUGGUGCGGUUCAAUAA	2.5	hypothetical protein
vvi-miRC171j-3p	VIT_214s0068g01780	1	CUAUAACCGUGCCGAGUUAGU	GAUAUUGGCACGGCUCAAUCA	0	squamosa promoter-binding-like protein 16-like nucleus
vvi-miRC171j-3p	VIT_215s0048g00270	1	CUAUAACCGUGCCGAGUUAGU	GAUAUUGGCGCGGCUCAAUCA	0.5	gras family transcription factor regulation of transcription
vvi-miRC171j-3p	VIT_217s0000g01250	1	CUAUAACCGUGCCGAGUUAGU	GAUAUUGGCACGGCUCAAUCA	0	protein protein binding
vvi-miRC171j-5p	VIT_211s0118g00490	3	AGACUCACUUGGCUCGGUUGU	UCUGAGUGAACUGAGCAAACC	2.5	octicosapeptide phox domain-containing protein toxin catabolic process
vvi-miRC171k-3p	VIT_202s0154g00400	1	CCUCUAUAACUGCGCCGAGUU	AGGGAUAUUGGCGCGGCUCAA	2	gras family transcription factor regulation of transcription
vvi-miRC171k-3p	VIT_204s0023g01380	1	CCUCUAUAACUGCGCCGAGUU	GGCGAUAUUGGCGCGGCUCAA	1.5	gras family transcription factor regulation of shoot system development
vvi-miRC171k-3p	VIT_215s0048g00270	1	CCUCUAUAACUGCGCCGAGUU	AGGGAUAUUGGCGCGGCUCAA	2	gras family transcription factor regulation of transcription
vvi-miRC171k-3p	VIT_217s0000g09495	1	CCUCUAUAACUGCGCCGAGUU	UGAGAUAUUGACGAGGCUUAA	2.5	serine-threonine protein plant- protein phosphorylation
vvi-miRC171l-3p	VIT_202s0154g00400	1	CUAUAACCGUGCCGAGUUAGU	GAUAUUGGCGCGGCUCAAUCA	0.5	gras family transcription factor regulation of transcription

miRNA name	Target gene	Number of target sites	miRNA sequence	Target sequence	Score	Target annotation
vvi-miRC171l-3p	VIT_204s0023g01380	1	CUAUAACCGUGCCGAGUUAGU	GAUAUUGGCGCGGCUCAAUCA	0.5	gras family transcription factor regulation of shoot system development
vvi-miRC171l-3p	VIT_212s0059g00650	1	CUAUAACCGUGCCGAGUUAGU	GAUAUUGGUGCGGUUCAAUAA	2.5	hypothetical protein
vvi-miRC171l-3p	VIT_214s0068g01780	1	CUAUAACCGUGCCGAGUUAGU	GAUAUUGGCACGGCUCAAUCA	0	squamosa promoter-binding-like protein 16-like nucleus
vvi-miRC171l-3p	VIT_215s0048g00270	1	CUAUAACCGUGCCGAGUUAGU	GAUAUUGGCGCGGCUCAAUCA	0.5	gras family transcription factor regulation of transcription
vvi-miRC171l-3p	VIT_217s0000g01250	1	CUAUAACCGUGCCGAGUUAGU	GAUAUUGGCACGGCUCAAUCA	0	protein protein binding
vvi-miRC171n-3p	VIT_202s0154g00400	1	CUAUAACCGUGCCGAGUUAGU	GAUAUUGGCGCGGCUCAAUCA	0.5	gras family transcription factor regulation of transcription
vvi-miRC171n-3p	VIT_204s0023g01380	1	CUAUAACCGUGCCGAGUUAGU	GAUAUUGGCGCGGCUCAAUCA	0.5	gras family transcription factor regulation of shoot system development
vvi-miRC171n-3p	VIT_212s0059g00650	1	CUAUAACCGUGCCGAGUUAGU	GAUAUUGGUGCGGUUCAAUAA	2.5	hypothetical protein
vvi-miRC171n-3p	VIT_214s0068g01780	1	CUAUAACCGUGCCGAGUUAGU	GAUAUUGGCACGGCUCAAUCA	0	squamosa promoter-binding-like protein 16-like nucleus
vvi-miRC171n-3p	VIT_215s0048g00270	1	CUAUAACCGUGCCGAGUUAGU	GAUAUUGGCGCGGCUCAAUCA	0.5	gras family transcription factor regulation of transcription
vvi-miRC171n-3p	VIT_217s0000g01250	1	CUAUAACCGUGCCGAGUUAGU	GAUAUUGGCACGGCUCAAUCA	0	protein protein binding
vvi-miRC172e-3p	VIT_203s0038g03920	2	CGUCGUAGUAGUUCUAAGAGU	GCAGCACCAUCAA-AUUCUCA	2.5	glutamate receptor extracellular-glutamate-gated ion channel activity
vvi-miRC172e-3p	VIT_206s0004g03590	3	CGUCGUAGUAGUUCUAAGAGU	GCAGCAUCAUCAGGAUUCUCA	0.5	ethylene-responsive transcription factor rap2-7-like oxidation-reduction process
vvi-miRC172e-3p	VIT_206s0061g00290	1	CGUCGUAGUAGUUCUAAGAGU	GUAGCAUCAUCAAGAUUCACA	1.5	hypothetical protein
vvi-miRC172e-3p	VIT_207s0031g00220	3	CGUCGUAGUAGUUCUAAGAGU	GCAGCAUCAUCAGGAUUCCCA	1.5	transcription factor apetala2 seed development
vvi-miRC172e-3p	VIT_208s0040g03180	1	CGUCGUAGUAGUUCUAAGAGU	GCAGCAUCAUCAGGAUUCUUG	1.5	ap2 domain-containing transcription factor oxidation-reduction process
vvi-miRC172e-3p	VIT_209s0018g01300	1	CGUCGUAGUAGUUCUAAGAGU	GCAGCAUCAUCAAGAAUCGCA	2	uncharacterized protein protein heterodimerization activity
vvi-miRC172e-3p	VIT_213s0019g03550	2	CGUCGUAGUAGUUCUAAGAGU	GCAGCAUCAUCAGGAUUCCCU	2.5	ap2 domain-containing transcription factor oxidation-reduction process
vvi-miRC172g-3p	VIT_203s0038g03920	2	CGUCGUAGUAGUUCUAAGAGU	GCAGCACCAUCAA-AUUCUCA	2.5	glutamate receptor extracellular-glutamate-gated ion channel activity
vvi-miRC172g-3p	VIT_206s0004g03590	3	CGUCGUAGUAGUUCUAAGAGU	GCAGCAUCAUCAGGAUUCUCA	0.5	ethylene-responsive transcription factor rap2-7-like oxidation-reduction process
vvi-miRC172g-3p	VIT_206s0061g00290	1	CGUCGUAGUAGUUCUAAGAGU	GUAGCAUCAUCAAGAUUCACA	1.5	hypothetical protein

miRNA name	Target gene	Number of target sites	miRNA sequence	Target sequence	Score	Target annotation
vvi-miRC172g-3p	VIT_207s0031g00220	3	CGUCGUAGUAGUUCUAAGAGU	GCAGCAUCAUCAGGAUUCCCA	1.5	transcription factor apetala2 seed development
vvi-miRC172g-3p	VIT_208s0040g03180	1	CGUCGUAGUAGUUCUAAGAGU	GCAGCAUCAUCAGGAUUCUUG	1.5	ap2 domain-containing transcription factor oxidation-reduction process
vvi-miRC172g-3p	VIT_209s0018g01300	1	CGUCGUAGUAGUUCUAAGAGU	GCAGCAUCAUCAAGAAUCGCA	2	uncharacterized protein protein heterodimerization activity
vvi-miRC172g-3p	VIT_213s0019g03550	2	CGUCGUAGUAGUUCUAAGAGU	GCAGCAUCAUCAGGAUUCCCU	2.5	ap2 domain-containing transcription factor oxidation-reduction process
vvi-miRC3624a-3p	VIT_200s0194g00300	1	UCAUCAUACGACGACGGGACU	AGUAGUAUGCUGCUGCCCUGA	0	uncharacterized protein loc1002608dd metal ion transport
vvi-miRC3624a-3p	VIT_200s0194g00330	2	UCAUCAUACGACGACGGGACU	AGUAGUAUGCUGCAACCCUGA	2	proline-rich protein metal ion transport
vvi-miRC3624a-3p	VIT_200s0194g00340	2	UCAUCAUACGACGACGGGACU	AGUAGUAUGCUGCUGCCCUGA	0	hypothetical protein
vvi-miRC3624a-3p	VIT_200s0259g00070	2	UCAUCAUACGACGACGGGACU	AGUAGUAUGCUGCAGCCCUCA	2	proline-rich protein metal ion transport
vvi-miRC3624a-3p	VIT_200s0259g00100	4	UCAUCAUACGACGACGGGACU	AGUAGUAUGCUGCAGCCCUCA	2	proline-rich protein metal ion transport
vvi-miRC3624a-3p	VIT_200s0753g00010	1	UCAUCAUACGACGACGGGACU	AGUAGUAUGCUGCAACCCUGA	2	proline-rich protein metal ion transport
vvi-miRC3624a-3p	VIT_200s0753g00030	1	UCAUCAUACGACGACGGGACU	AGUAGUAUGUUGUUGCCCUGA	1	hypothetical protein
vvi-miRC3624a-3p	VIT_200s2419g00010	1	UCAUCAUACGACGACGGGACU	AGUAGUAUGCAGCUACCCUGA	2	uncharacterized protein loc1008524dd metal ion transport
vvi-miRC390a-5p	VIT_206s0004g01080	1	CCGCGAUAGGGAGGACUCGAA	GGUGUUAUUCCUCUUGAGUUU	2.5	dna (cytosine-5)-methyltransferase DNA methylation on cytosine within a CNG sequence
vvi-miRC390a-5p	VIT_210s0003g01890	1	CCGCGAUAGGGAGGACUCGAA	GGCGUUCUUCCUCCUGAGCUU	2	lrr receptor-like serine threonine-protein kinase rfk1 protein phosphorylation
vvi-miRC390a-5p	VIT_212s0059g01410	1	CCGCGAUAGGGAGGACUCGAA	GGCGAUAUCUCUCCUGAGCUU	1.5	hypothetical protein
vvi-miRC390a-5p	VIT_216s0098g01090	1	CCGCGAUAGGGAGGACUCGAA	GGCUCUAUACCUCCUGAGCUU	2	leucine-rich repeat receptor-like protein kinase pepr1 defense response
vvi-miRC396e-5p	VIT_200s0179g00260	1	UUCAAGUUCUUUCGGCACCUU	GGGGUCAAGAGAGCCGUGGAA	2.5	calcium-transporting atpase plasma membrane-type-like respiratory burst involved in defense response
vvi-miRC396e-5p	VIT_201s0026g02170	1	UUCAAGUUCUUUCGGCACCUU	AAGUUGAAGAAGGCCAUGGAA	2.5	ran gtpase binding gravitropism
vvi-miRC403g-5p	VIT_205s0077g00800	2	CUCAAACACGCACUUAGAUU	AAGUUUGUGCGUGAAUCAAA	2	gins complex subunit 4 regulation of cell cycle
vvi-miRC403g-5p	VIT_207s0005g01690	1	CUCAAACACGCACUUAGAUU	GAGUUUGUGCGUGACUCUAA	1	hypothetical protein
vvi-miRC403g-5p	VIT_210s0042g01150	1	CUCAAACACGCACUUAGAUU	GAGUUUGUGCGUGAAUCUAA	0	protein argonaute 2-like defense response to bacterium
vvi-miRC403g-5p	VIT_210s0042g01180	2	CUCAAACACGCACUUAGAUU	GAGUUUGUGCGUGAAUCUAA	0	protein argonaute 2-like defense response to bacterium
vvi-miRC403g-5p	VIT_210s0042g01200	2	CUCAAACACGCACUUAGAUU	GAGUUUGUGCGUGAAUCUAA	0	protein argonaute 2-like defense response to bacterium
vvi-miRC403g-5p	VIT_211s0052g00930	7	CUCAAACACGCACUUAGAUU	GAGCUUGUGCAUGAAUCUGA	2.5	set domain-containing protein coenzyme A
vvi-miRC403g-5p	VIT_215s0021g01190	1	CUCAAACACGCACUUAGAUU	GAGGUUGUGUGUGAAUCUAA	1.5	hypothetical protein
vvi-miRC477c-5p	VIT_201s0010g02270	1	UUUAACCUUCGGAAACUCCCU	AUAUUGGAAGCCUUUGAGGGA	1	gras family transcription factor regulation of transcription

miRNA name	Target gene	Number of target sites	miRNA sequence	Target sequence	Score	Target annotation
vvi-miRC477c-5p	VIT_217s0000g10300	1	UUUAACCUUCGGAAACUCCCU	AUAUUGGAAGCCUUUGAGGGA	1	gras family transcription factor regulation of transcription
vvi-miRC477i-5p	VIT_201s0010g02270	1	UUUAACCUUCGGAAACUCCCU	AUAUUGGAAGCCUUUGAGGGA	1	gras family transcription factor regulation of transcription
vvi-miRC477i-5p	VIT_217s0000g10300	1	UUUAACCUUCGGAAACUCCCU	AUAUUGGAAGCCUUUGAGGGA	1	gras family transcription factor regulation of transcription
vvi-miRC477j-3p	VIT_212s0028g01600	1	CCAGGGGGUAGCCGAAGGUCG	GUUCCUCCAUCUGCUUCCAGC	2.5	atp-dependent zinc metalloprotease ftsh chloroplastic-like pentose-phosphate shunt
vvi-miRC477j-5p	VIT_201s0010g02270	1	UUUAACCUUCGGAAACUCCCU	AUAUUGGAAGCCUUUGAGGGA	1	gras family transcription factor regulation of transcription
vvi-miRC477j-5p	VIT_217s0000g10300	1	UUUAACCUUCGGAAACUCCCU	AUAUUGGAAGCCUUUGAGGGA	1	gras family transcription factor regulation of transcription
vvi-miRC477k-5p	VIT_201s0010g02270	1	UUUAACCUUCGGAAACUCCCU	AUAUUGGAAGCCUUUGAGGGA	1	gras family transcription factor regulation of transcription
vvi-miRC477k-5p	VIT_217s0000g10300	1	UUUAACCUUCGGAAACUCCCU	AUAUUGGAAGCCUUUGAGGGA	1	gras family transcription factor regulation of transcription
vvi-miRC477l-5p	VIT_201s0010g02270	1	UUUAACCUUCGGAAACUCCCU	AUAUUGGAAGCCUUUGAGGGA	1	gras family transcription factor regulation of transcription
vvi-miRC477l-5p	VIT_217s0000g10300	1	UUUAACCUUCGGAAACUCCCU	AUAUUGGAAGCCUUUGAGGGA	1	gras family transcription factor regulation of transcription
vvi-miRC477m-5p	VIT_201s0010g02270	1	UUUAACCUUCGGAAACUCCCU	AUAUUGGAAGCCUUUGAGGGA	1	gras family transcription factor regulation of transcription
vvi-miRC477m-5p	VIT_217s0000g10300	1	UUUAACCUUCGGAAACUCCCU	AUAUUGGAAGCCUUUGAGGGA	1	gras family transcription factor regulation of transcription
vvi-miRC477n-3p	VIT_202s0087g00760	1	CAAGGGGGUGGCUGAAGGUUG	GUUCUUCCACUGCCUUCCAAC	2.5	pentatricopeptide repeat-containing protein mitochondrion
vvi-miRC477n-3p	VIT_218s0001g12680	3	CAAGGGGGUGGCUGAAGGUUG	GUUUCCCCGCCGACUUCCAAC	1	hypothetical protein
vvi-miRC477n-5p	VIT_201s0010g02270	1	UUUAACCUUCGGAAACUCCCU	AUAUUGGAAGCCUUUGAGGGA	1	gras family transcription factor regulation of transcription
vvi-miRC477n-5p	VIT_217s0000g10300	1	UUUAACCUUCGGAAACUCCCU	AUAUUGGAAGCCUUUGAGGGA	1	gras family transcription factor regulation of transcription
vvi-miRC477o-3p	VIT_218s0001g12680	3	CCAGGGGGUGGCUGAAGGUUG	GUUUCCCCGCCGACUUCCAAC	2	hypothetical protein
vvi-miRC477o-5p	VIT_201s0010g02270	1	UUUAACCUUCGGAAACUCCCU	AUAUUGGAAGCCUUUGAGGGA	1	gras family transcription factor regulation of transcription

miRNA name	Target gene	Number of target sites	miRNA sequence	Target sequence	Score	Target annotation
vvi-miRC477o-5p	VIT_217s0000g10300	1	UUUAACCUUCGGAAACUCCCU	AUAUUGGAAGCCUUUGAGGGA	1	gras family transcription factor regulation of transcription
vvi-miRC477p-5p	VIT_201s0010g02270	1	UUUAACCUUCGGAAACUCCCU	AUAUUGGAAGCCUUUGAGGGA	1	gras family transcription factor regulation of transcription
vvi-miRC477p-5p	VIT_217s0000g10300	1	UUUAACCUUCGGAAACUCCCU	AUAUUGGAAGCCUUUGAGGGA	1	gras family transcription factor regulation of transcription
vvi-miRC482a-3p	VIT_212s0034g01700	1	AACCUUACCCGCCGUAACCCUU	UGGGUAUGGGCGGUAUUGGGAA	2.5	disease resistance rpp13-like protein 1-like defense response
vvi-miRC482a-3p	VIT_217s0000g10080	3	AACCUUACCCGCCGUAACCCUU	UUCGAGUGGGCGGCAAUGGGAA	2.5	domain-containing protein os01g09054dd DNA binding
vvi-miRC482b-3p	VIT_209s0002g03790	1	CCUUACCCUCCACAACCAUUCU	GGAAUGGGAGGUGUUGGGAAAA	2	probable disease resistance protein at5g63020-like defense response
vvi-miRC482b-3p	VIT_209s0002g04890	1	CCUUACCCUCCACAACCAUUCU	GGAAUGGGGGGUGUUGGCAAAA	2.5	disease resistance protein rps5-like nucleoside-triphosphatase activity
vvi-miRC482b-3p	VIT_209s0002g04950	1	CCUUACCCUCCACAACCAUUCU	GGAAUGGGGGGUGUUGGCAAAA	2.5	disease resistance protein rps5-like nucleoside-triphosphatase activity
vvi-miRC482b-3p	VIT_209s0002g04980	1	CCUUACCCUCCACAACCAUUCU	GGAAUGGGGGGUGUUGGCAAAA	2.5	disease resistance protein rps5-like nucleoside-triphosphatase activity
vvi-miRC482b-3p	VIT_209s0002g05040	1	CCUUACCCUCCACAACCAUUCU	GGAAUGGGGGGUGUUGGCAAAA	2.5	disease resistance protein rps5-like nucleoside-triphosphatase activity
vvi-miRC482b-3p	VIT_209s0002g05920	4	CCUUACCCUCCACAACCAUUCU	GGAAUGGGGGGUGUUGGCAAAA	2.5	disease resistance protein rps5-like nucleoside-triphosphatase activity
vvi-miRC482b-3p	VIT_209s0018g00480	1	CCUUACCCUCCACAACCAUUCU	GGGAUGGGAGGCGUUGGGAAGA	2.5	probable disease resistance protein at5g63020-like plant-type hypersensitive response
vvi-miRC482b-3p	VIT_213s0047g00095	1	CCUUACCCUCCACAACCAUUCU	GGGAUGGGUGGAGUUGGUAAGA	2.5	hypothetical protein
vvi-miRC482b-3p	VIT_213s0064g01165	1	CCUUACCCUCCACAACCAUUCU	GGAGUGGGAGGUGUUGAUAGGA	2	anthocyanin 5-aromatic acyltransferase-like
vvi-miRC530-3p	VIT_200s0267g00060	1	AGACGUGGAAGUGGACGUGGA	UCUUCACCUUCACCU-CACCU	2.5	hypothetical protein
vvi-miRC530-3p	VIT_201s0011g03410	4	AGACGUGGAAGUGGACGUGGA	ACUGCACCUGCACCUGCACCU	2	uv excision repair protein proteasome binding
vvi-miRC530-3p	VIT_201s0011g03410	8	AGACGUGGAAGUGGACGUGGA	CCUGCACCUGCACCUGCACCU	2	uv excision repair protein proteasome binding
vvi-miRC530-3p	VIT_213s0067g01550	1	AGACGUGGAAGUGGACGUGGA	UCUUCACCUUCACCUUCACCU	2	probable lrr receptor-like serine threonine-protein kinase mrh1-like protein phosphorylation
vvi-miRC530-3p	VIT_216s0013g00610	1	AGACGUGGAAGUGGACGUGGA	UCUGCAUCUUUAUUUGCAUCU	2.5	mago nashi-like protein nucleolus
vvi-miRC530-3p	VIT_218s0001g01800	1	AGACGUGGAAGUGGACGUGGA	UCUGCAUCUGCAUCUGCAUCU	2.5	mrna splicing mRNA binding
vvi-miRC530-3p	VIT_218s0086g00180	1	AGACGUGGAAGUGGACGUGGA	UCUUCACCUUCACCUUCACCU	2	protein drug transmembrane transport
vvi-miRC530-5p	VIT_202s0012g02510	1	UCCACGUCCACGUUUACGUCU	AGGU-CAGGUGCAAAUGCAGG	2	protein zinc ion binding
vvi-miRC530-5p	VIT_205s0020g04860	6	UCCACGUCCACGUUUACGUCU	AGGUGCAGGUGCAAAUGCAGG	0.5	protein zinc ion binding
vvi-miRC530-5p	VIT_206s0080g00400	1	UCCACGUCCACGUUUACGUCU	AGGUACAGGUGCAGGUGCAGA	2	ribosomal protein l1 translation
vvi-miRC530-5p	VIT_206s0080g00400	1	UCCACGUCCACGUUUACGUCU	AGGUGCAGGUGCAGAUGCAGG	1	ribosomal protein l1 translation
vvi-miRC530-5p	VIT_210s0116g00140	1	UCCACGUCCACGUUUACGUCU	AGGUGGAGAUGCAAAUGCAGG	2.5	subtilisin-like protease-like identical protein binding

miRNA name	Target gene	Number of target sites	miRNA sequence	Target sequence	Score	Target annotation
vvi-miRC530b-5p	VIT_202s0012g02510	1	UCCACGUCCACGUUUACGUCU	AGGU-CAGGUGCAAAUGCAGG	2	protein zinc ion binding
vvi-miRC530b-5p	VIT_205s0020g04860	6	UCCACGUCCACGUUUACGUCU	AGGUGCAGGUGCAAAUGCAGG	0.5	protein zinc ion binding
vvi-miRC530b-5p	VIT_206s0080g00400	1	UCCACGUCCACGUUUACGUCU	AGGUACAGGUGCAGGUGCAGA	2	ribosomal protein l1 translation
vvi-miRC530b-5p	VIT_206s0080g00400	1	UCCACGUCCACGUUUACGUCU	AGGUGCAGGUGCAGAUGCAGG	1	ribosomal protein l1 translation
vvi-miRC530b-5p	VIT_210s0116g00140	1	UCCACGUCCACGUUUACGUCU	AGGUGGAGAUGCAAAUGCAGG	2.5	subtilisin-like protease-like identical protein binding
vvi-miRC7122-3p	VIT_214s0030g00640	2	CGAAUAUGUCUCUUUCUGCCA	GCUUAUAUA-AGAAAGAUGGU	2.5	DNAJ heat shock n-terminal domain-containing protein protein folding
vvi-miRC827-3p	VIT_202s0025g04540	2	ACAAACAACUACUAGUAGAUU	UGUUCGUUGAUGGUCAUCUAA	1.5	spx domain-containing membrane protein vacuolar membrane
vvi-miRC827-3p	VIT_204s0008g00910	4	ACAAACAACUACUAGUAGAUU	UGUUUG-UGAUCAUCAUCUAA	2.5	histone deacetylase 2 histone deacetylase activity
vvi-miR156b-3p	VIT_200s0358g00010	1	CACGAGUGAGAGAAGACAGU	GUGCUCUCUCUCGUCUGUCA	2	hypothetical protein
vvi-miR156b-3p	VIT_201s0010g03710	6	CACGAGUGAGAGAAGACAGU	GUGCUCUCUCUCUUCUGUCA	1	squamosa promoter-binding-like protein 2 regulation of biological process
vvi-miR156b-3p	VIT_201s0010g03910	3	CACGAGUGAGAGAAGACAGU	GUGCUCUCUCUCUUCUGUCA	1	squamosa promoter-binding-like protein 13-like nucleus
vvi-miR156b-3p	VIT_201s0011g00130	2	CACGAGUGAGAGAAGACAGU	GUGCUCUCUCUCUUCUGUCA	1	squamosa promoter-binding-like protein 6-like DNA binding
vvi-miR156b-3p	VIT_204s0008g00960	2	CACGAGUGAGAGAAGACAGU	GUGCUCACUCUCUUCUGUCA	0	calcineurin b-like protein dd multidimensional cell growth
vvi-miR156b-3p	VIT_204s0008g02640	2	CACGAGUGAGAGAAGACAGU	GUGCUCUCUCUCUUCUGUCA	1	plastid-lipid-associated protein 8 photosynthesis
vvi-miR156b-3p	VIT_208s0007g06270	1	CACGAGUGAGAGAAGACAGU	GUGCUCUCUCUCUUCUGUCA	1	squamosa promoter-binding-like protein 9-like nucleus
vvi-miR156b-3p	VIT_209s0002g04110	2	CACGAGUGAGAGAAGACAGU	GUGGUCACUCUUUGCUGUCA	2.5	peroxisome assembly protein cytosol
vvi-miR156b-3p	VIT_211s0065g00170	7	CACGAGUGAGAGAAGACAGU	GUGCUCUCUCUCUUCUGUCA	1	squamosa promoter-binding-like protein 12-like RNA binding
vvi-miR156b-3p	VIT_212s0028g03350	1	CACGAGUGAGAGAAGACAGU	AUGCUCUCUCUCUUCUGUCA	2	squamosa promoter binding 4 regulation of transcription
vvi-miR156b-3p	VIT_214s0068g01780	1	CACGAGUGAGAGAAGACAGU	GUGCUUUCUCUCUUCUGUCA	2.5	squamosa promoter-binding-like protein 16-like nucleus
vvi-miR156b-3p	VIT_214s0108g00380	1	CACGAGUGAGAGAAGACAGU	GUACUCACUCUCUUCUGUCA	1	probable receptor-like protein kinase at5g15080-like ATP binding
vvi-miR156b-3p	VIT_215s0021g02290	1	CACGAGUGAGAGAAGACAGU	GUGCUCUCUCUCUUCUGUCA	1	squamosa promoter-binding-like protein 7-like transcription

miRNA name	Target gene	Number of target sites	miRNA sequence	Target sequence	Score	Target annotation
vvi-miR156b-3p	VIT_217s0000g01260	1	CACGAGUGAGAGAAGACAGU	GUGCUCUCUCUCUUCUGUCA	1	squamosa promoter-binding-like protein dd DNA binding
vvi-miR156b-3p	VIT_217s0000g05020	3	CACGAGUGAGAGAAGACAGU	GUGCUCUCUCUCUUCUGUCA	1	squamosa promoter-binding-like protein 6-like DNA binding
vvi-miR156b-3p	VIT_217s0000g08780	5	CACGAGUGAGAGAAGACAGU	UUGCUCAUUUUCUUCUGUUA	2.5	uncharacterized protein chloroplast
vvi-miR156b-3p	VIT_219s0014g02350	1	CACGAGUGAGAGAAGACAGU	UUGCUCCCUCUCUUCUGUCA	2	squamosa promoter binding 4 regulation of transcription
vvi-miR156b-3p	VIT_219s0090g01180	2	CACGAGUGAGAGAAGACAGU	AUGCUCACUCUUUUCUGUCA	1.5	brain protein dd chaperone binding
vvi-miR156c-5p	VIT_200s0358g00010	1	CACGAGUGAGAGAAGACAGU	GUGCUCUCUCUCGUCUGUCA	2	hypothetical protein
vvi-miR156c-5p	VIT_201s0010g03710	6	CACGAGUGAGAGAAGACAGU	GUGCUCUCUCUCUUCUGUCA	1	squamosa promoter-binding-like protein 2 regulation of biological process
vvi-miR156c-5p	VIT_201s0010g03910	3	CACGAGUGAGAGAAGACAGU	GUGCUCUCUCUCUUCUGUCA	1	squamosa promoter-binding-like protein 13-like nucleus
vvi-miR156c-5p	VIT_201s0011g00130	2	CACGAGUGAGAGAAGACAGU	GUGCUCUCUCUCUUCUGUCA	1	squamosa promoter-binding-like protein 6-like DNA binding
vvi-miR156c-5p	VIT_204s0008g00960	2	CACGAGUGAGAGAAGACAGU	GUGCUCACUCUCUUCUGUCA	0	calcineurin b-like protein dd multidimensional cell growth
vvi-miR156c-5p	VIT_204s0008g02640	2	CACGAGUGAGAGAAGACAGU	GUGCUCUCUCUCUUCUGUCA	1	plastid-lipid-associated protein 8 photosynthesis
vvi-miR156c-5p	VIT_208s0007g06270	1	CACGAGUGAGAGAAGACAGU	GUGCUCUCUCUCUUCUGUCA	1	squamosa promoter-binding-like protein 9-like nucleus
vvi-miR156c-5p	VIT_209s0002g04110	2	CACGAGUGAGAGAAGACAGU	GUGGUCACUCUUUGCUGUCA	2.5	peroxisome assembly protein cytosol
vvi-miR156c-5p	VIT_211s0065g00170	7	CACGAGUGAGAGAAGACAGU	GUGCUCUCUCUCUUCUGUCA	1	squamosa promoter-binding-like protein 12-like RNA binding
vvi-miR156c-5p	VIT_212s0028g03350	1	CACGAGUGAGAGAAGACAGU	AUGCUCUCUCUCUUCUGUCA	2	squamosa promoter binding 4 regulation of transcription
vvi-miR156c-5p	VIT_214s0068g01780	1	CACGAGUGAGAGAAGACAGU	GUGCUUUCUCUCUUCUGUCA	2.5	squamosa promoter-binding-like protein 16-like nucleus
vvi-miR156c-5p	VIT_214s0108g00380	1	CACGAGUGAGAGAAGACAGU	GUACUCACUCUCUUCUGUCA	1	probable receptor-like protein kinase at5g15080-like ATP binding
vvi-miR156c-5p	VIT_215s0021g02290	1	CACGAGUGAGAGAAGACAGU	GUGCUCUCUCUCUUCUGUCA	1	squamosa promoter-binding-like protein 7-like transcription
vvi-miR156c-5p	VIT_217s0000g01260	1	CACGAGUGAGAGAAGACAGU	GUGCUCUCUCUCUUCUGUCA	1	squamosa promoter-binding-like protein dd DNA binding
vvi-miR156c-5p	VIT_217s0000g05020	3	CACGAGUGAGAGAAGACAGU	GUGCUCUCUCUCUUCUGUCA	1	squamosa promoter-binding-like protein 6-like DNA binding
vvi-miR156c-5p	VIT_217s0000g08780	5	CACGAGUGAGAGAAGACAGU	UUGCUCAUUUUCUUCUGUUA	2.5	uncharacterized protein chloroplast
vvi-miR156c-5p	VIT_219s0014g02350	1	CACGAGUGAGAGAAGACAGU	UUGCUCCCUCUCUUCUGUCA	2	squamosa promoter binding 4 regulation of transcription
vvi-miR156c-5p	VIT_219s0090g01180	2	CACGAGUGAGAGAAGACAGU	AUGCUCACUCUUUUCUGUCA	1.5	brain protein dd chaperone binding
vvi-miR156d-5p	VIT_200s0358g00010	1	CACGAGUGAGAGAAGACAGU	GUGCUCUCUCUCGUCUGUCA	2	hypothetical protein

miRNA name	Target gene	Number of target sites	miRNA sequence	Target sequence	Score	Target annotation
vvi-miR156d-5p	VIT_201s0010g03710	6	CACGAGUGAGAGAAGACAGU	GUGCUCUCUCUCUUCUGUCA	1	squamosa promoter-binding-like protein 2 regulation of biological process
vvi-miR156d-5p	VIT_201s0010g03910	3	CACGAGUGAGAGAAGACAGU	GUGCUCUCUCUCUUCUGUCA	1	squamosa promoter-binding-like protein 13-like nucleus
vvi-miR156d-5p	VIT_201s0011g00130	2	CACGAGUGAGAGAAGACAGU	GUGCUCUCUCUCUUCUGUCA	1	squamosa promoter-binding-like protein 6-like DNA binding
vvi-miR156d-5p	VIT_204s0008g00960	2	CACGAGUGAGAGAAGACAGU	GUGCUCACUCUCUUCUGUCA	0	calcineurin b-like protein dd multidimensional cell growth
vvi-miR156d-5p	VIT_204s0008g02640	2	CACGAGUGAGAGAAGACAGU	GUGCUCUCUCUCUUCUGUCA	1	plastid-lipid-associated protein 8 photosynthesis
vvi-miR156d-5p	VIT_208s0007g06270	1	CACGAGUGAGAGAAGACAGU	GUGCUCUCUCUCUUCUGUCA	1	squamosa promoter-binding-like protein 9-like nucleus
vvi-miR156d-5p	VIT_209s0002g04110	2	CACGAGUGAGAGAAGACAGU	GUGGUCACUCUUUGCUGUCA	2.5	peroxisome assembly protein cytosol
vvi-miR156d-5p	VIT_211s0065g00170	7	CACGAGUGAGAGAAGACAGU	GUGCUCUCUCUCUUCUGUCA	1	squamosa promoter-binding-like protein 12-like RNA binding
vvi-miR156d-5p	VIT_212s0028g03350	1	CACGAGUGAGAGAAGACAGU	AUGCUCUCUCUCUUCUGUCA	2	squamosa promoter binding 4 regulation of transcription
vvi-miR156d-5p	VIT_214s0068g01780	1	CACGAGUGAGAGAAGACAGU	GUGCUUUCUCUCUUCUGUCA	2.5	squamosa promoter-binding-like protein 16-like nucleus
vvi-miR156d-5p	VIT_214s0108g00380	1	CACGAGUGAGAGAAGACAGU	GUACUCACUCUCUUCUGUCA	1	probable receptor-like protein kinase at5g15080-like ATP binding
vvi-miR156d-5p	VIT_215s0021g02290	1	CACGAGUGAGAGAAGACAGU	GUGCUCUCUCUCUUCUGUCA	1	squamosa promoter-binding-like protein 7-like transcription
vvi-miR156d-5p	VIT_217s0000g01260	1	CACGAGUGAGAGAAGACAGU	GUGCUCUCUCUCUUCUGUCA	1	squamosa promoter-binding-like protein dd DNA binding
vvi-miR156d-5p	VIT_217s0000g05020	3	CACGAGUGAGAGAAGACAGU	GUGCUCUCUCUCUUCUGUCA	1	squamosa promoter-binding-like protein 6-like DNA binding
vvi-miR156d-5p	VIT_217s0000g08780	5	CACGAGUGAGAGAAGACAGU	UUGCUCAUUUUCUUCUGUUA	2.5	uncharacterized protein chloroplast
vvi-miR156d-5p	VIT_219s0014g02350	1	CACGAGUGAGAGAAGACAGU	UUGCUCCCUCUCUUCUGUCA	2	squamosa promoter binding 4 regulation of transcription
vvi-miR156d-5p	VIT_219s0090g01180	2	CACGAGUGAGAGAAGACAGU	AUGCUCACUCUUUUCUGUCA	1.5	brain protein dd chaperone binding
vvi-miR156e-5p	VIT_201s0010g03710	6	CACGAGUGAGAGGAGACAGU	GUGCUCUCUCUCUUCUGUCA	1.5	squamosa promoter-binding-like protein 2 regulation of biological process
vvi-miR156e-5p	VIT_201s0010g03910	3	CACGAGUGAGAGGAGACAGU	GUGCUCUCUCUCUUCUGUCA	1.5	squamosa promoter-binding-like protein 13-like nucleus

miRNA name	Target gene	Number of target sites	miRNA sequence	Target sequence	Score	Target annotation
vvi-miR156e-5p	VIT_201s0011g00130	2	CACGAGUGAGAGGAGACAGU	GUGCUCUCUCUCUUCUGUCA	1.5	squamosa promoter-binding-like protein 6-like DNA binding
vvi-miR156e-5p	VIT_204s0008g00960	2	CACGAGUGAGAGGAGACAGU	GUGCUCACUCUCUUCUGUCA	0.5	calcineurin b-like protein dd multidimensional cell growth
vvi-miR156e-5p	VIT_204s0008g02640	2	CACGAGUGAGAGGAGACAGU	GUGCUCUCUCUCCUCUGUCA	1	plastid-lipid-associated protein 8 photosynthesis
vvi-miR156e-5p	VIT_205s0020g02110	1	CACGAGUGAGAGGAGACAGU	GAGCUCACUCUCCUUUGACA	2.5	peroxidase dd heme binding
vvi-miR156e-5p	VIT_208s0007g06270	1	CACGAGUGAGAGGAGACAGU	GUGCUCUCUCUCUUCUGUCA	1.5	squamosa promoter-binding-like protein 9-like nucleus
vvi-miR156e-5p	VIT_211s0065g00170	7	CACGAGUGAGAGGAGACAGU	GUGCUCUCUCUCUUCUGUCA	1.5	squamosa promoter-binding-like protein 12-like RNA binding
vvi-miR156e-5p	VIT_212s0028g03350	1	CACGAGUGAGAGGAGACAGU	AUGCUCUCUCUCUUCUGUCA	2.5	squamosa promoter binding 4 regulation of transcription
vvi-miR156e-5p	VIT_214s0108g00380	1	CACGAGUGAGAGGAGACAGU	GUACUCACUCUCUUCUGUCA	1.5	probable receptor-like protein kinase at5g15080-like ATP binding
vvi-miR156e-5p	VIT_215s0021g02290	1	CACGAGUGAGAGGAGACAGU	GUGCUCUCUCUCUUCUGUCA	1.5	squamosa promoter-binding-like protein 7-like transcription
vvi-miR156e-5p	VIT_217s0000g01260	1	CACGAGUGAGAGGAGACAGU	GUGCUCUCUCUCUUCUGUCA	1.5	squamosa promoter-binding-like protein dd DNA binding
vvi-miR156e-5p	VIT_217s0000g05020	3	CACGAGUGAGAGGAGACAGU	GUGCUCUCUCUCUUCUGUCA	1.5	squamosa promoter-binding-like protein 6-like DNA binding
vvi-miR156e-5p	VIT_219s0014g02350	1	CACGAGUGAGAGGAGACAGU	UUGCUCCCUCUCUUCUGUCA	2.5	squamosa promoter binding 4 regulation of transcription
vvi-miR156e-5p	VIT_219s0090g01180	2	CACGAGUGAGAGGAGACAGU	AUGCUCACUCUUUUCUGUCA	2	brain protein dd chaperone binding
vvi-miR156f-5p	VIT_201s0010g03710	6	CACGAGAGAUAGAAGACAGUU	GUGCUCUCUCUCUUCUGUCAA	1	squamosa promoter-binding-like protein 2 regulation of biological process
vvi-miR156f-5p	VIT_201s0010g03910	3	CACGAGAGAUAGAAGACAGUU	GUGCUCUCUCUCUUCUGUCAU	2	squamosa promoter-binding-like protein 13-like nucleus
vvi-miR156f-5p	VIT_201s0011g00130	2	CACGAGAGAUAGAAGACAGUU	GUGCUCUCUCUCUUCUGUCAG	1.5	squamosa promoter-binding-like protein 6-like DNA binding
vvi-miR156f-5p	VIT_208s0007g06270	1	CACGAGAGAUAGAAGACAGUU	GUGCUCUCUCUCUUCUGUCAA	1	squamosa promoter-binding-like protein 9-like nucleus
vvi-miR156f-5p	VIT_210s0003g00050	2	CACGAGAGAUAGAAGACAGUU	AUGCUCUCUAUCUCCUGUCAA	2	squamosa promoter binding-like protein metal ion binding
vvi-miR156f-5p	VIT_211s0065g00170	7	CACGAGAGAUAGAAGACAGUU	GUGCUCUCUCUCUUCUGUCAA	1	squamosa promoter-binding-like protein 12-like RNA binding
vvi-miR156f-5p	VIT_214s0068g01780	1	CACGAGAGAUAGAAGACAGUU	GUGCUUUCUCUCUUCUGUCAU	2.5	squamosa promoter-binding-like protein 16-like nucleus
vvi-miR156f-5p	VIT_215s0021g02290	1	CACGAGAGAUAGAAGACAGUU	GUGCUCUCUCUCUUCUGUCAU	2	squamosa promoter-binding-like protein 7-like transcription
vvi-miR156f-5p	VIT_215s0046g01230	1	CACGAGAGAUAGAAGACAGUU	GUGCUCUCUGUUUUGUGUUAA	2.5	hypothetical protein

miRNA name	Target gene	Number of target sites	miRNA sequence	Target sequence	Score	Target annotation
vvi-miR156f-5p	VIT_216s0039g02000	2	CACGAGAGAUAGAAGACAGUU	GUGCUUUCUUUCUUCCGUCAA	2.5	peptidyl-trna hydrolase ii-like protein cytoplasmic membrane-bounded vesicle
vvi-miR156f-5p	VIT_217s0000g01260	1	CACGAGAGAUAGAAGACAGUU	GUGCUCUCUCUCUUCUGUCAU	2	squamosa promoter-binding-like protein dd DNA binding
vvi-miR156f-5p	VIT_217s0000g05020	3	CACGAGAGAUAGAAGACAGUU	GUGCUCUCUCUCUUCUGUCAU	2	squamosa promoter-binding-like protein 6-like DNA binding
vvi-miR156g-5p	VIT_201s0010g03710	6	CACGAGAGAUAGAAGACAGUU	GUGCUCUCUCUCUUCUGUCAA	1	squamosa promoter-binding-like protein 2 regulation of biological process
vvi-miR156g-5p	VIT_201s0010g03910	3	CACGAGAGAUAGAAGACAGUU	GUGCUCUCUCUCUUCUGUCAU	2	squamosa promoter-binding-like protein 13-like nucleus
vvi-miR156g-5p	VIT_201s0011g00130	2	CACGAGAGAUAGAAGACAGUU	GUGCUCUCUCUCUUCUGUCAG	1.5	squamosa promoter-binding-like protein 6-like DNA binding
vvi-miR156g-5p	VIT_208s0007g06270	1	CACGAGAGAUAGAAGACAGUU	GUGCUCUCUCUCUUCUGUCAA	1	squamosa promoter-binding-like protein 9-like nucleus
vvi-miR156g-5p	VIT_210s0003g00050	2	CACGAGAGAUAGAAGACAGUU	AUGCUCUCUAUCUCCUGUCAA	2	squamosa promoter binding-like protein metal ion binding
vvi-miR156g-5p	VIT_211s0065g00170	7	CACGAGAGAUAGAAGACAGUU	GUGCUCUCUCUCUUCUGUCAA	1	squamosa promoter-binding-like protein 12-like RNA binding
vvi-miR156g-5p	VIT_214s0068g01780	1	CACGAGAGAUAGAAGACAGUU	GUGCUUUCUCUCUUCUGUCAU	2.5	squamosa promoter-binding-like protein 16-like nucleus
vvi-miR156g-5p	VIT_215s0021g02290	1	CACGAGAGAUAGAAGACAGUU	GUGCUCUCUCUCUUCUGUCAU	2	squamosa promoter-binding-like protein 7-like transcription
vvi-miR156g-5p	VIT_215s0046g01230	1	CACGAGAGAUAGAAGACAGUU	GUGCUCUCUGUUUUGUGUUAA	2.5	hypothetical protein
vvi-miR156g-5p	VIT_216s0039g02000	2	CACGAGAGAUAGAAGACAGUU	GUGCUUUCUUUCUUCCGUCAA	2.5	peptidyl-trna hydrolase ii-like protein cytoplasmic membrane-bounded vesicle
vvi-miR156g-5p	VIT_217s0000g01260	1	CACGAGAGAUAGAAGACAGUU	GUGCUCUCUCUCUUCUGUCAU	2	squamosa promoter-binding-like protein dd DNA binding
vvi-miR156g-5p	VIT_217s0000g05020	3	CACGAGAGAUAGAAGACAGUU	GUGCUCUCUCUCUUCUGUCAU	2	squamosa promoter-binding-like protein 6-like DNA binding
vvi-miR156i-3p	VIT_201s0010g03710	6	CACGAGAGAUAGAAGACAGUU	GUGCUCUCUCUCUUCUGUCAA	1	squamosa promoter-binding-like protein 2 regulation of biological process
vvi-miR156i-3p	VIT_201s0010g03910	3	CACGAGAGAUAGAAGACAGUU	GUGCUCUCUCUCUUCUGUCAU	2	squamosa promoter-binding-like protein 13-like nucleus
vvi-miR156i-3p	VIT_201s0011g00130	2	CACGAGAGAUAGAAGACAGUU	GUGCUCUCUCUCUUCUGUCAG	1.5	squamosa promoter-binding-like protein 6-like DNA binding

miRNA name	Target gene	Number of target sites	miRNA sequence	Target sequence	Score	Target annotation
vvi-miR156i-3p	VIT_208s0007g06270	1	CACGAGAGAUAGAAGACAGUU	GUGCUCUCUCUCUUCUGUCAA	1	squamosa promoter-binding-like protein 9-like nucleus
vvi-miR156i-3p	VIT_210s0003g00050	2	CACGAGAGAUAGAAGACAGUU	AUGCUCUCUAUCUCCUGUCAA	2	squamosa promoter binding-like protein metal ion binding
vvi-miR156i-3p	VIT_211s0065g00170	7	CACGAGAGAUAGAAGACAGUU	GUGCUCUCUCUCUUCUGUCAA	1	squamosa promoter-binding-like protein 12-like RNA binding
vvi-miR156i-3p	VIT_214s0068g01780	1	CACGAGAGAUAGAAGACAGUU	GUGCUUUCUCUCUUCUGUCAU	2.5	squamosa promoter-binding-like protein 16-like nucleus
vvi-miR156i-3p	VIT_215s0021g02290	1	CACGAGAGAUAGAAGACAGUU	GUGCUCUCUCUCUUCUGUCAU	2	squamosa promoter-binding-like protein 7-like transcription
vvi-miR156i-3p	VIT_215s0046g01230	1	CACGAGAGAUAGAAGACAGUU	GUGCUCUCUGUUUUGUGUUAA	2.5	hypothetical protein
vvi-miR156i-3p	VIT_216s0039g02000	2	CACGAGAGAUAGAAGACAGUU	GUGCUUUCUUUCUUCCGUCAA	2.5	peptidyl-trna hydrolase ii-like protein cytoplasmic membrane-bounded vesicle
vvi-miR156i-3p	VIT_217s0000g01260	1	CACGAGAGAUAGAAGACAGUU	GUGCUCUCUCUCUUCUGUCAU	2	squamosa promoter-binding-like protein dd DNA binding
vvi-miR156i-3p	VIT_217s0000g05020	3	CACGAGAGAUAGAAGACAGUU	GUGCUCUCUCUCUUCUGUCAU	2	squamosa promoter-binding-like protein 6-like DNA binding
vvi-miR159c-3p	VIT_212s0059g01070	2	AUCUCGAGGGAAGUUAGGUUU	UAGAGCUCCUUGCAAUACAAA	2.5	condensin complex subunit 2-like microtubule cytoskeleton organization
vvi-miR159c-3p	VIT_213s0067g01630	1	AUCUCGAGGGAAGUUAGGUUU	UGGAGCUCCCUUCACUCCAAU	2.5	transcription factor gamyb-like flower development
vvi-miR159c-3p	VIT_219s0014g01700	1	AUCUCGAGGGAAGUUAGGUUU	UAGAGCCCCCUUCAAACCAAA	2	hypothetical protein
vvi-miR159c-5p	VIT_201s0011g06470	5	GAUAACCUGAAGUUCCUCGAG	CUAUUGGCCUACAAGGAGCUC	2	probable NAD(P)H-dependent oxidoreductase
vvi-miR162-3p	VIT_217s0000g04470	1	GACCUACGUCUCCAAAUAGCU	CUGGAUGCAGCGGUUCAUCGA	2	hypothetical protein
vvi-miR162-5p	VIT_217s0000g04470	1	CUAGCUACUUGGCGACGUAGG	GGUCGAUAAACCUCUGCAUCC	2.5	hypothetical protein
vvi-miR164a-5p	VIT_203s0038g04270	6	ACGUGCACGGGACGAAGAGGU	UGCACAUGCCCUGCUUCUGCA	2	ankyrin repeat domain protein folding
vvi-miR164a-5p	VIT_209s0002g00760	5	ACGUGCACGGGACGAAGAGGU	AGCAUGUGCCCUGCUUCUCCA	1.5	uncharacterized protein heme binding
vvi-miR164a-5p	VIT_217s0000g06400	3	ACGUGCACGGGACGAAGAGGU	CUCACGUGCCCUGCUUCUCCA	2	nac domain ipr0034dd sequence-specific DNA binding transcription factor activity
vvi-miR164a-5p	VIT_219s0014g02200	1	ACGUGCACGGGACGAAGAGGU	AGCACGUGCCCUGGUUCUCCA	2	no apical meristem gynoecium development
vvi-miR164a-5p	VIT_219s0027g00230	2	ACGUGCACGGGACGAAGAGGU	AGCAAGUGCCCUGCUUCUCCG	2.5	nac domain protein regulation of transcription
vvi-miR164c-3p	VIT_201s0137g00270	1	CUACCCCUUCUCCCCGUGUAC	GAUGGGGAGGAGAGGAACAUG	2.5	rna recognition motif-containing protein nucleic acid binding
vvi-miR164c-5p	VIT_203s0038g04270	6	ACGUGCACGGGACGAAGAGGU	UGCACAUGCCCUGCUUCUGCA	2	ankyrin repeat domain protein folding
vvi-miR164c-5p	VIT_209s0002g00760	5	ACGUGCACGGGACGAAGAGGU	AGCAUGUGCCCUGCUUCUCCA	1.5	uncharacterized protein heme binding
vvi-miR164c-5p	VIT_217s0000g06400	3	ACGUGCACGGGACGAAGAGGU	CUCACGUGCCCUGCUUCUCCA	2	nac domain ipr0034dd sequence-specific DNA binding transcription factor activity
vvi-miR164c-5p	VIT_219s0014g02200	1	ACGUGCACGGGACGAAGAGGU	AGCACGUGCCCUGGUUCUCCA	2	no apical meristem gynoecium development
vvi-miR164c-5p	VIT_219s0027g00230	2	ACGUGCACGGGACGAAGAGGU	AGCAAGUGCCCUGCUUCUCCG	2.5	nac domain protein regulation of transcription
vvi-miR164d-5p	VIT_203s0038g04270	6	ACGUGCACGGGACGAAGAGGU	UGCACAUGCCCUGCUUCUGCA	2	ankyrin repeat domain protein folding

miRNA name	Target gene	Number of target sites	miRNA sequence	Target sequence	Score	Target annotation
vvi-miR164d-5p	VIT_209s0002g00760	5	ACGUGCACGGGACGAAGAGGU	AGCAUGUGCCCUGCUUCUCCA	1.5	uncharacterized protein heme binding
vvi-miR164d-5p	VIT_217s0000g06400	3	ACGUGCACGGGACGAAGAGGU	CUCACGUGCCCUGCUUCUCCA	2	nac domain ipr0034dd sequence-specific DNA binding transcription factor activity
vvi-miR164d-5p	VIT_219s0014g02200	1	ACGUGCACGGGACGAAGAGGU	AGCACGUGCCCUGGUUCUCCA	2	no apical meristem gynoecium development
vvi-miR164d-5p	VIT_219s0027g00230	2	ACGUGCACGGGACGAAGAGGU	AGCAAGUGCCCUGCUUCUCCG	2.5	nac domain protein regulation of transcription
vvi-miR166a-3p	VIT_218s0075g00480	7	CCUUACUUCGGACCAGGCUCU	GGAAUGAAGUAUGGUCCGAGA	2.5	tmv resistance protein n-like defense response
vvi-miR166a-5p	VIT_214s0060g01210	1	CUAGAACCUAGUUUGGAGUAA	GAUUUUGGAUCAAAACCCAUU	2.5	bzip transcription factor positive regulation of seed maturation
vvi-miR166b-5p	VIT_209s0002g03740	1	CUCCUUACUUCGGACCAGGCU	CUGGAAUGAAGCCUGGUCCGG	2.5	homeobox-leucine zipper protein athb-dd cellular response to nitrogen starvation
vvi-miR166c-3p	VIT_209s0002g03740	1	CCCCUUACUUCGGACCAGGCU	CUGGAAUGAAGCCUGGUCCGG	2.5	homeobox-leucine zipper protein athb-dd cellular response to nitrogen starvation
vvi-miR166c-5p	VIT_214s0108g00480	4	GGAGCUCGGUCUGUUGUAAGG	CCUUGAGCUGGGCAACAUUCU	2.5	transcription factor bhlhdd cellular response to iron ion starvation
vvi-miR166d-3p	VIT_209s0002g03740	1	CCCCUUACUUCGGACCAGGCU	CUGGAAUGAAGCCUGGUCCGG	2.5	homeobox-leucine zipper protein athb-dd cellular response to nitrogen starvation
vvi-miR166e-3p	VIT_209s0002g03740	1	CCCCUUACUUCGGACCAGGCU	CUGGAAUGAAGCCUGGUCCGG	2.5	homeobox-leucine zipper protein athb-dd cellular response to nitrogen starvation
vvi-miR166e-5p	VIT_214s0108g00480	4	GGAGCUCGGUCUGUUGUAAGG	CCUUGAGCUGGGCAACAUUCU	2.5	transcription factor bhlhdd cellular response to iron ion starvation
vvi-miR166f-3p	VIT_209s0002g03740	1	CCCCUUACUUCGGACCAGGCU	CUGGAAUGAAGCCUGGUCCGG	2.5	homeobox-leucine zipper protein athb-dd cellular response to nitrogen starvation
vvi-miR166g-3p	VIT_209s0002g03740	1	CCCCUUACUUCGGACCAGGCU	CUGGAAUGAAGCCUGGUCCGG	2.5	homeobox-leucine zipper protein athb-dd cellular response to nitrogen starvation
vvi-miR166g-5p	VIT_205s0102g00770	2	AGAGCUUGGUCUGUUGUAAGG	UCUCGAACCAGAUAACUGUCC	2.5	lipid binding nucleus
vvi-miR166h-3p	VIT_209s0002g03740	1	CCCCUUACUUCGGACCAGGCU	CUGGAAUGAAGCCUGGUCCGG	2.5	homeobox-leucine zipper protein athb-dd cellular response to nitrogen starvation
vvi-miR167b-5p	VIT_206s0080g00260	1	UCUAGUACGACCGUCGAAGU	AGAUCAUGCU-GCAGCUGCA	2.5	poly protein ADP-ribosylation
vvi-miR167b-5p	VIT_208s0007g02340	2	UCUAGUACGACCGUCGAAGU	AGAUUAUGCUGGCUGCUUCA	1.5	protein cytosol
vvi-miR167d-5p	VIT_206s0080g00260	1	UCUAGUACGACCGUCGAAGU	AGAUCAUGCU-GCAGCUGCA	2.5	poly protein ADP-ribosylation
vvi-miR167d-5p	VIT_208s0007g02340	2	UCUAGUACGACCGUCGAAGU	AGAUUAUGCUGGCUGCUUCA	1.5	protein cytosol
vvi-miR167e-5p	VIT_206s0080g00260	1	UCUAGUACGACCGUCGAAGU	AGAUCAUGCU-GCAGCUGCA	2.5	poly protein ADP-ribosylation
vvi-miR167e-5p	VIT_208s0007g02340	2	UCUAGUACGACCGUCGAAGU	AGAUUAUGCUGGCUGCUUCA	1.5	protein cytosol

miRNA name	Target gene	Number of target sites	miRNA sequence	Target sequence	Score	Target annotation
vvi-miR168-3p	VIT_208s0007g07890	1	UAAGUCAACUACGUUCCGCCC	GUUUAGGUGAUGCAAGGCGGG	2	pentatricopeptide repeat-containing protein mitochondrial-like exo-poly-alpha-galacturonosidase activity
vvi-miR169e-5p	VIT_200s0125g00290	1	UCCGUUCAGUAGGAACCGAU	GGGUAAGUUGUCCUUGGCUG	2.5	protein regulation of meristem growth
vvi-miR169e-5p	VIT_201s0146g00380	2	UCCGUUCAGUAGGAACCGAU	AGGCAAGUCAUCCUUCGCUA	1	litaf-domain-containing protein response to absence of light
vvi-miR169e-5p	VIT_208s0007g08250	14	UCCGUUCAGUAGGAACCGAU	AGGCAAAUCAUUCUUGGCUU	2.5	nuclear transcription factor y subunit a- regulation of transcription
vvi-miR169e-5p	VIT_209s0002g01590	3	UCCGUUCAGUAGGAACCGAU	UGGAAAGUCAUCCUUGGCUG	2.5	nuclear transcription factor y subunit a-3 regulation of transcription
vvi-miR169e-5p	VIT_211s0016g01480	4	UCCGUUCAGUAGGAACCGAU	GGGCAAUUCAUCCUUGGCUU	2.5	nuclear transcription factor y subunit a- regulation of transcription
vvi-miR169e-5p	VIT_213s0064g00860	3	UCCGUUCAGUAGGAACCGAU	AGGCAAUUCAUUCUUGGCUU	2.5	nuclear transcription factor y subunit a- regulation of transcription
vvi-miR169g-5p	VIT_209s0002g01590	3	AGCCGUUCAGUAGGAACCGAC	UUGGAAAGUCAUCCUUGGCUG	1.5	nuclear transcription factor y subunit a-3 regulation of transcription
vvi-miR169r-3p	VIT_216s0050g00390	1	UGACUCAGUUCAGUUGAACGG	ACUGAGUCAACUCAACUUGGC	2	amp dependent cytosol
vvi-miR169r-5p	VIT_208s0032g01190	1	GCCGUUCAGUAGGAACUGAGU	UGGUAAUUCAUCCUUGGCUCA	2.5	nuclear transcription factor y subunit a-dd regulation of transcription
vvi-miR169r-5p	VIT_211s0016g00450	1	GCCGUUCAGUAGGAACUGAGU	GGACAAGUCAUUCUUGACUCA	2.5	eukaryotic translation initiation factor 3 subunit k eukaryotic translation initiation factor 3 complex
vvi-miR169t-3p	VIT_216s0050g00390	1	UGACUCAGUUCAGUUGAACGG	ACUGAGUCAACUCAACUUGGC	2	amp dependent cytosol
vvi-miR171b-3p	VIT_202s0154g00400	1	CCUCUAUAACUGCGCCGAGUU	AGGGAUAUUGGCGCGGCUCAA	2	gras family transcription factor regulation of transcription
vvi-miR171b-3p	VIT_204s0023g01380	1	CCUCUAUAACUGCGCCGAGUU	GGCGAUAUUGGCGCGGCUCAA	1.5	gras family transcription factor regulation of shoot system development
vvi-miR171b-3p	VIT_215s0048g00270	1	CCUCUAUAACUGCGCCGAGUU	AGGGAUAUUGGCGCGGCUCAA	2	gras family transcription factor regulation of transcription
vvi-miR171b-3p	VIT_217s0000g09495	1	CCUCUAUAACUGCGCCGAGUU	UGAGAUAUUGACGAGGCUUAA	2.5	serine-threonine protein plant- protein phosphorylation
vvi-miR171i-3p	VIT_202s0154g00400	1	CUAUAACCGUGCCGAGUUAGU	GAUAUUGGCGCGGCUCAAUCA	0.5	gras family transcription factor regulation of transcription
vvi-miR171i-3p	VIT_204s0023g01380	1	CUAUAACCGUGCCGAGUUAGU	GAUAUUGGCGCGGCUCAAUCA	0.5	gras family transcription factor regulation of shoot system development
vvi-miR171i-3p	VIT_212s0059g00650	1	CUAUAACCGUGCCGAGUUAGU	GAUAUUGGUGCGGUUCAAUAA	2.5	hypothetical protein
vvi-miR171i-3p	VIT_214s0068g01780	1	CUAUAACCGUGCCGAGUUAGU	GAUAUUGGCACGGCUCAAUCA	0	squamosa promoter-binding-like protein 16-like nucleus
vvi-miR171i-3p	VIT_215s0048g00270	1	CUAUAACCGUGCCGAGUUAGU	GAUAUUGGCGCGGCUCAAUCA	0.5	gras family transcription factor regulation of transcription
vvi-miR171i-3p	VIT_217s0000g01250	1	CUAUAACCGUGCCGAGUUAGU	GAUAUUGGCACGGCUCAAUCA	0	protein protein binding

miRNA name	Target gene	Number of target sites	miRNA sequence	Target sequence	Score	Target annotation
vvi-miR172d-3p	VIT_203s0038g03920	2	CGUCGUAGUAGUUCUAAGAGU	GCAGCACCAUCAA-AUUCUCA	2.5	glutamate receptor extracellular-glutamate-gated ion channel activity
vvi-miR172d-3p	VIT_206s0004g03590	3	CGUCGUAGUAGUUCUAAGAGU	GCAGCAUCAUCAGGAUUCUCA	0.5	AP2/ERF domain ethylene-responsive transcription factor rap2-7-like
vvi-miR172d-3p	VIT_206s0061g00290	1	CGUCGUAGUAGUUCUAAGAGU	GUAGCAUCAUCAAGAUUCACA	1.5	hypothetical protein
vvi-miR172d-3p	VIT_207s0031g00220	3	CGUCGUAGUAGUUCUAAGAGU	GCAGCAUCAUCAGGAUUCCCA	1.5	transcription factor apetala2 seed development
vvi-miR172d-3p	VIT_208s0040g03180	1	CGUCGUAGUAGUUCUAAGAGU	GCAGCAUCAUCAGGAUUCUUG	1.5	ap2 domain-containing transcription factor oxidation-reduction process
vvi-miR172d-3p	VIT_209s0018g01300	1	CGUCGUAGUAGUUCUAAGAGU	GCAGCAUCAUCAAGAAUCGCA	2	uncharacterized protein protein heterodimerization activity
vvi-miR172d-3p	VIT_213s0019g03550	2	CGUCGUAGUAGUUCUAAGAGU	GCAGCAUCAUCAGGAUUCCCU	2.5	ap2 domain-containing transcription factor oxidation-reduction process
vvi-miR2111-3p	VIT_208s0007g01270	1	UCAUUAGACGUUGGUCUCCUG	AGUAAUCUGCAACCAGAGGAC	0	dynein light chain flagellar outer arm Rho guanyl-nucleotide exchange factor activity
vvi-miR2111-3p	VIT_209s0002g02550	6	UCAUUAGACGUUGGUCUCCUG	AGUAAU-UGCAACCAGAGGAU	2	hypothetical protein
vvi-miR2111-3p	VIT_210s0071g00980	3	UCAUUAGACGUUGGUCUCCUG	AGUAAUCAGCAAGCAGAGGAU	2.5	uncharacterized protein protein desumoylation
vvi-miR2111-5p	VIT_208s0007g01270	1	AUCUGGAGUCCUACGUCUAAU	UAGACCUCAGGAUGCAGAUUA	0	dynein light chain flagellar outer arm Rho guanyl-nucleotide exchange factor activity
vvi-miR2950-5p	VIT_204s0008g03190	1	AGGUCACACGUUCUCUACCUU	UUCAGUGUGCAAAAGAUGGAA	1.5	hypothetical protein
vvi-miR2950-5p	VIT_206s0004g00860	1	AGGUCACACGUUCUCUACCUU	UCUAGUUUGCAAGAGAUGGCA	2.5	f-box kelch-repeat protein cytoplasm
vvi-miR2950-5p	VIT_213s0158g00130	1	AGGUCACACGUUCUCUACCUU	UCCAGUGUG-AAGACAUGGAA	2.5	lactoylglutathione lyase cytoplasm
vvi-miR2950-5p	VIT_219s0014g01860	6	AGGUCACACGUUCUCUACCUU	UCCAGUGUGCAAAAGAUGGGA	1.5	rna exonuclease 4-like nucleic acid binding
vvi-miR319b-3p	VIT_202s0025g00870	1	CCCUCGAGGGAAGUCAGGUU	GGGAGCUCCCUUCACUGCAA	2	hypothetical protein
vvi-miR319b-3p	VIT_211s0016g05010	1	CCCUCGAGGGAAGUCAGGUU	AGGAGCUCCCUUCAGUCCAA	1	metallothiol transferase fosb cytoplasm
vvi-miR319b-3p	VIT_213s0067g01630	1	CCCUCGAGGGAAGUCAGGUU	UGGAGCUCCCUUCACUCCAA	2	transcription factor gamyb-like flower development
vvi-miR319c-3p	VIT_202s0025g00870	1	CCCUCGAGGGAAGUCAGGUU	GGGAGCUCCCUUCACUGCAA	2	hypothetical protein
vvi-miR319c-3p	VIT_211s0016g05010	1	CCCUCGAGGGAAGUCAGGUU	AGGAGCUCCCUUCAGUCCAA	1	metallothiol transferase fosb cytoplasm
vvi-miR319c-3p	VIT_213s0067g01630	1	CCCUCGAGGGAAGUCAGGUU	UGGAGCUCCCUUCACUCCAA	2	transcription factor gamyb-like flower development
vvi-miR319c-5p	VIT_200s0566g00010	1	CUCACCUGACUUCUUUCGAGA	GAGUCGACUCAAGAAAGCUCU	2	upf03dd protein chloroplastic-like peroxiredoxin activity
vvi-miR319c-5p	VIT_200s1308g00020	3	CUCACCUGACUUCUUUCGAGA	GAGUCGACUCAAGAAAGCUCU	2	upf03dd protein chloroplastic-like peroxiredoxin activity
vvi-miR319e-3p	VIT_211s0016g05010	1	UCCUCGAGGGAAGUCAGGUUU	AGGAGCUCCCUUCAGUCCAAA	0	metallothiol transferase fosb cytoplasm

miRNA name	Target gene	Number of target sites	miRNA sequence	Target sequence	Score	Target annotation
vvi-miR319f-3p	VIT_202s0025g00870	1	CCCUCGAGGGAAGUCAGGUU	GGGAGCUCCCUUCACUGCAA	2	hypothetical protein
vvi-miR319f-3p	VIT_211s0016g05010	1	CCCUCGAGGGAAGUCAGGUU	AGGAGCUCCCUUCAGUCCAA	1	metallothiol transferase fosb cytoplasm
vvi-miR319f-3p	VIT_213s0067g01630	1	CCCUCGAGGGAAGUCAGGUU	UGGAGCUCCCUUCACUCCAA	2	transcription factor gamyb-like flower development
vvi-miR319g-3p	VIT_202s0025g00870	1	CCCUCGAGGGAAGUCAGGUU	GGGAGCUCCCUUCACUGCAA	2	hypothetical protein
vvi-miR319g-3p	VIT_211s0016g05010	1	CCCUCGAGGGAAGUCAGGUU	AGGAGCUCCCUUCAGUCCAA	1	metallothiol transferase fosb cytoplasm
vvi-miR319g-3p	VIT_213s0067g01630	1	CCCUCGAGGGAAGUCAGGUU	UGGAGCUCCCUUCACUCCAA	2	transcription factor gamyb-like flower development
vvi-miR3623-5p	VIT_200s0288g00010	1	ACCACGAACCUACUUGAACACU	UGGUGCUUGGAUGAGCUUGAUA	2.5	tmv resistance protein n-like defense response
vvi-miR3623-5p	VIT_200s0288g00040	1	ACCACGAACCUACUUGAACACU	UGGUGCUUGGAUGAGCUUGAUA	2.5	tmv resistance protein n-like defense response
vvi-miR3623-5p	VIT_218s0001g06340	1	ACCACGAACCUACUUGAACACU	UGGUGCUUGGAGGAGCUGGUGA	2.5	tmv resistance protein n-like defense response
vvi-miR3623-5p	VIT_218s0041g01620	1	ACCACGAACCUACUUGAACACU	UGGUGCUUGGAUGAACUCGUCA	2	tmv resistance protein n-like defense response
vvi-miR3623-5p	VIT_218s0041g01650	1	ACCACGAACCUACUUGAACACU	UGGUGCUUGGAUGAACUCGUCA	2	tmv resistance protein n-like defense response
vvi-miR3623-5p	VIT_218s0041g02180	2	ACCACGAACCUACUUGAACACU	UGGUGCUUGGAGGAGCUUGUGA	1.5	protein da1-related 1-like ovule morphogenesis
vvi-miR3627-5p	VIT_211s0052g00320	6	UCACGGCAGAGAGGACGCUGUU	GGUGUCAUCUCUCCUGCGACAG	2.5	calcium-transporting atpase plasma membrane-type-like inflorescence morphogenesis
vvi-miR3629a-3p	VIT_200s0198g00160	1	GGAUGUAAAAGAGUCGUCGGU	CCAAUAUUUUCUCAACAGCCA	2.5	chromosome transmission fidelity protein dd homolog nucleoside-triphosphatase activity
vvi-miR3629a-3p	VIT_201s0011g04410	1	GGAUGUAAAAGAGUCGUCGGU	CCUACAUUUUGUCGGCAGCCA	1.5	sec-independent protein translocase protein abscisic acid
vvi-miR3629a-3p	VIT_201s0127g00895	1	GGAUGUAAAAGAGUCGUCGGU	CCUACAUUUUCUCAGUAACCA	1.5	endoplasmic oxidoreductin-1 endoplasmic reticulum membrane
vvi-miR3629a-3p	VIT_203s0091g01030	2	GGAUGUAAAAGAGUCGUCGGU	CCUACGUUUUCUCUGCAACCA	2.5	telomere repeat binding factor 1 glucuronoxylan
vvi-miR3629a-3p	VIT_204s0008g04480	2	GGAUGUAAAAGAGUCGUCGGU	CCUACAUUUU-UCAGCAACCA	2.5	ring finger and chy zinc finger domain-containing protein nucleus
vvi-miR3629a-3p	VIT_204s0008g04490	1	GGAUGUAAAAGAGUCGUCGGU	CCUACGUUUUCUCAGCGGCCA	1	hypothetical protein
vvi-miR3629a-3p	VIT_204s0044g01620	3	GGAUGUAAAAGAGUCGUCGGU	CUUAUAUUUUCUUAGCAGCCA	1.5	copine (calcium-dependent phospholipid-binding protein) family zinc ion binding
vvi-miR3629a-3p	VIT_206s0004g00450	2	GGAUGUAAAAGAGUCGUCGGU	UCUACAUUUUCUCAGCAGCCA	0.5	rna polymerase ii transcription mediator regulation of transcription from RNA polymerase II promoter
vvi-miR3629a-3p	VIT_207s0031g01230	2	GGAUGUAAAAGAGUCGUCGGU	UCCUCAUUUUCUCAGCAGCCA	2.5	60s ribosomal protein ldd structural constituent of ribosome
vvi-miR3629a-3p	VIT_208s0007g06820	1	GGAUGUAAAAGAGUCGUCGGU	CCUACAUUUUCUCGGUAGACA	2	kinesin motor protein microtubule-based movement
vvi-miR3629a-3p	VIT_208s0007g06820	2	GGAUGUAAAAGAGUCGUCGGU	GCUGCAUUUUCUCAGCAACCA	2.5	kinesin motor protein microtubule-based movement
vvi-miR3629a-3p	VIT_209s0002g03240	5	GGAUGUAAAAGAGUCGUCGGU	UCUACACUUUCUCAGCAACCA	2.5	uncharacterized protein mitochondrion
vvi-miR3629a-3p	VIT_209s0054g00440	1	GGAUGUAAAAGAGUCGUCGGU	CCUCAAUUUUCUCAGCAGCCA	2	gata transcription factor dd sequence-specific DNA binding transcription factor activity

miRNA name	Target gene	Number of target sites	miRNA sequence	Target sequence	Score	Target annotation
vvi-miR3629a-3p	VIT_211s0016g03380	1	GGAUGUAAAAGAGUCGUCGGU	CCUGCACUUUCUCAGCAACCA	2.5	probable glycosyltransferase at5g03795-like glucuronosyl-N-acetylglucosaminyl-proteoglycan 4-alpha-N-acetylglucosaminyltransferase activity
vvi-miR3629a-3p	VIT_211s0016g04820	5	GGAUGUAAAAGAGUCGUCGGU	CCGACAUUUUCUCAGCAGCCA	1	tudor-like rna-binding protein RNA binding
vvi-miR3629a-3p	VIT_211s0065g00340	2	GGAUGUAAAAGAGUCGUCGGU	UCCACAUUUUCUCAGCAGCCA	1.5	hua enhancer 2 sugar mediated signaling pathway
vvi-miR3629a-3p	VIT_212s0035g01020	1	GGAUGUAAAAGAGUCGUCGGU	CUUACUUUUUCUCAGCAGCCG	2	60s ribosomal protein nucleolus
vvi-miR3629a-3p	VIT_213s0019g04380	1	GGAUGUAAAAGAGUCGUCGGU	UCUACAUUUUCUUGGCACCCA	2.5	protein developmental growth
vvi-miR3629a-3p	VIT_213s0047g01200	1	GGAUGUAAAAGAGUCGUCGGU	CCUCCAUUUUCUCAGCAACCA	2	obtusifoliol 14alpha-demethylase methionine
vvi-miR3629a-3p	VIT_213s0064g00580	4	GGAUGUAAAAGAGUCGUCGGU	CCUACAUUUUCUCGGCAGCCA	0.5	dd kda peptidyl-prolyl isomerase protein folding
vvi-miR3629a-3p	VIT_213s0073g00550	1	GGAUGUAAAAGAGUCGUCGGU	CCUGCAUUUUCUCAGCAACUA	2	lysosomal beta glucosidase-like anchored to membrane
vvi-miR3629a-3p	VIT_214s0060g01430	3	GGAUGUAAAAGAGUCGUCGGU	UUUAUAUUUUCUCAGCAGCCA	1.5	uncharacterized protein single-organism cellular process
vvi-miR3629a-3p	VIT_214s0060g01580	1	GGAUGUAAAAGAGUCGUCGGU	CCUGCAUUUUCUCGGCAACCG	2.5	protein plasma membrane
vvi-miR3629a-3p	VIT_214s0066g00830	2	GGAUGUAAAAGAGUCGUCGGU	CCUGCAUUUUCUCACCAACCA	2.5	sirohydrochlorin ferrochelatase metal ion binding
vvi-miR3629a-3p	VIT_215s0046g03190	1	GGAUGUAAAAGAGUCGUCGGU	CCUGCAUUUUCUCAGCUGCCA	1.5	r2r3-myb transcription response to jasmonic acid stimulus
vvi-miR3629a-3p	VIT_216s0039g01420	1	GGAUGUAAAAGAGUCGUCGGU	CCUCCAUUUUCUUGGCAGCCA	2	nucleotide-diphospho-sugar transferases superfamily protein pollen germination
vvi-miR3629a-3p	VIT_216s0039g02430	2	GGAUGUAAAAGAGUCGUCGGU	CCUGCAAUUUCUCAGCAACCA	2.5	uncharacterized protein single-organism cellular process
vvi-miR3629a-3p	VIT_216s0050g01780	1	GGAUGUAAAAGAGUCGUCGGU	CUUCCAUUUUCUCAGCGGCCA	2	probable membrane-associated kinase regulator 2-like response to stimulus
vvi-miR3629a-3p	VIT_216s0098g01870	6	GGAUGUAAAAGAGUCGUCGGU	CCUACAUUUUUUCAGCAGCCA	0.5	calcineurin b-like protein 4 hypotonic salinity response
vvi-miR3629a-3p	VIT_216s0098g01880	3	GGAUGUAAAAGAGUCGUCGGU	CCUACAUUUUCUAAGCAACCA	2	dipeptidyl peptidase cytosol
vvi-miR3629a-3p	VIT_217s0000g01150	1	GGAUGUAAAAGAGUCGUCGGU	CCUACAUUUUCUCAGCAGCCA	0	curved dna-binding protein protein folding
vvi-miR3629a-5p	VIT_203s0038g02970	2	GAACCGACGACUCUUUUACGC	UUUGGUUGCUGAGAAAAUGCA	2	serine threonine-protein kinase atr-like telomere maintenance in response to DNA damage
vvi-miR3629a-5p	VIT_204s0008g02000	1	GAACCGACGACUCUUUUACGC	UUUGGUUGCUGAGAAAAGGCG	2	uncharacterized protein nucleus
vvi-miR3629a-5p	VIT_205s0020g02030	2	GAACCGACGACUCUUUUACGC	UUUGGUUGCUGAGAUAAUGCG	2	dehydroquinate dehydratase shikimate dehydrogenase cytosol
vvi-miR3629a-5p	VIT_206s0061g00700	1	GAACCGACGACUCUUUUACGC	UUUGGGUGCUGAGAAAAUGCG	1.5	auxin-regulated expressed plasma membrane

miRNA name	Target gene	Number of target sites	miRNA sequence	Target sequence	Score	Target annotation
vvi-miR3629a-5p	VIT_207s0104g00880	2	GAACCGACGACUCUUUUACGC	UUUGGCUGCUGAGAAACUGUG	2	histidine phosphotransfer protein cytoplasm
vvi-miR3629a-5p	VIT_210s0003g02690	3	GAACCGACGACUCUUUUACGC	UUUGGUUGCUGAGAAAACGUG	2.5	scp1-like small phosphatase 4b CTD phosphatase activity
vvi-miR3629a-5p	VIT_211s0103g00550	4	GAACCGACGACUCUUUUACGC	UUUGGUUGGUGAGAAAAUGUG	2.5	subtilisin-like protease-like plant extracellular matrix
vvi-miR3629a-5p	VIT_212s0059g00900	2	GAACCGACGACUCUUUUACGC	UUUGGUUGCUGAGAAAAUGGG	2	hypothetical protein
vvi-miR3629a-5p	VIT_212s0059g00920	1	GAACCGACGACUCUUUUACGC	UUUGGUUGCUGAGAAAAUGGG	2	pentatricopeptide repeat-containing protein mitochondrion
vvi-miR3629a-5p	VIT_212s0059g01160	1	GAACCGACGACUCUUUUACGC	UUUGGUUGCUGAGAAAGUGCU	2.5	cupin-like protein
vvi-miR3629a-5p	VIT_213s0064g01230	2	GAACCGACGACUCUUUUACGC	UUUGGCUGCUGAGAAAUUGCA	2.5	u6 snrna-associated sm-like protein lsm7 nucleus
vvi-miR3629a-5p	VIT_214s0030g01280	1	GAACCGACGACUCUUUUACGC	UUUGGUUGCUGAGAAAAUGCG	1	double-stranded rna-binding protein 2-like RNA binding
vvi-miR3629a-5p	VIT_214s0060g01580	1	GAACCGACGACUCUUUUACGC	UUUGGUUGCUGAGAAAAUGUG	1.5	protein plasma membrane
vvi-miR3629a-5p	VIT_215s0021g00090	1	GAACCGACGACUCUUUUACGC	UUUGGUUGCUGAGAAAAUGCA	2	sister chromatid cohesion protein dcc1-like nucleus
vvi-miR3629a-5p	VIT_215s0024g00820	1	GAACCGACGACUCUUUUACGC	UUUGGCUGCUGAGAAAAUGUG	1	protein response to abscisic acid stimulus
vvi-miR3629a-5p	VIT_216s0050g02440	2	GAACCGACGACUCUUUUACGC	UUUGGUUGCUGAGAAAGUGUG	2	protein dimerization DNA binding
vvi-miR3629a-5p	VIT_217s0000g01160	2	GAACCGACGACUCUUUUACGC	UUUGGUUGCUGAGAAAAGGUG	2.5	nodulin-like protein mitochondrion
vvi-miR3629a-5p	VIT_217s0000g01860	1	GAACCGACGACUCUUUUACGC	UUUGGUUGCUGAUAAAAUGUG	2.5	gaga-binding transcriptional activator glucan endo-1
vvi-miR3629a-5p	VIT_217s0000g05820	1	GAACCGACGACUCUUUUACGC	UUUGGUUGCUGAGAAAAUGUG	1.5	ubiquitin-conjugating enzyme negative regulation of flower development
vvi-miR3629a-5p	VIT_217s0000g06880	2	GAACCGACGACUCUUUUACGC	UUUGGUUGCUGAGAAAGUGUG	2	heparanase-like protein 1-like plant-type cell wall
vvi-miR3629a-5p	VIT_218s0001g14070	5	GAACCGACGACUCUUUUACGC	CAUGGCUGCUGGGAAAGUGUG	2.5	hypothetical protein
vvi-miR3629a-5p	VIT_219s0014g02260	1	GAACCGACGACUCUUUUACGC	UUUGGUUGCUGAGAAAUUGUG	2.5	hypothetical protein
vvi-miR3629a-5p	VIT_219s0090g00630	5	GAACCGACGACUCUUUUACGC	UUUGGUUGCUGAGAAAAUGCU	2	tetraacyldisaccharide 4 -kinase family protein lipid A
vvi-miR3629a-5p	VIT_219s0090g01230	3	GAACCGACGACUCUUUUACGC	CUUGGCUGCUAACAAAAUGCG	2	microtubule-associated proteins 65-1 cortical microtubule organization
vvi-miR3629a-5p	VIT_219s0090g01230	3	GAACCGACGACUCUUUUACGC	UUUGGCUGCUGAGAAAAUGUG	1	microtubule-associated proteins 65-1 cortical microtubule organization
vvi-miR3629a-5p	VIT_219s0090g01230	3	GAACCGACGACUCUUUUACGC	UUUGGUUGCUGAGAAAAUGCA	2	microtubule-associated proteins 65-1 cortical microtubule organization
vvi-miR3629c-5p	VIT_200s0198g00160	1	GGAUGUAAAAGAGUCGUCGGU	CCAAUAUUUUCUCAACAGCCA	2.5	chromosome transmission fidelity protein dd homolog nucleoside-triphosphatase activity
vvi-miR3629c-5p	VIT_201s0011g04410	1	GGAUGUAAAAGAGUCGUCGGU	CCUACAUUUUGUCGGCAGCCA	1.5	sec-independent protein translocase protein abscisic acid
vvi-miR3629c-5p	VIT_201s0127g00895	1	GGAUGUAAAAGAGUCGUCGGU	CCUACAUUUUCUCAGUAACCA	1.5	endoplasmic oxidoreductin-1 endoplasmic reticulum membrane

miRNA name	Target gene	Number of target sites	miRNA sequence	Target sequence	Score	Target annotation
vvi-miR3629c-5p	VIT_203s0091g01030	2	GGAUGUAAAAGAGUCGUCGGU	CCUACGUUUUCUCUGCAACCA	2.5	telomere repeat binding factor 1 glucuronoxylan
vvi-miR3629c-5p	VIT_204s0008g04480	2	GGAUGUAAAAGAGUCGUCGGU	CCUACAUUUU-UCAGCAACCA	2.5	ring finger and chy zinc finger domain-containing protein nucleus
vvi-miR3629c-5p	VIT_204s0008g04490	1	GGAUGUAAAAGAGUCGUCGGU	CCUACGUUUUCUCAGCGGCCA	1	hypothetical protein
vvi-miR3629c-5p	VIT_204s0044g01620	3	GGAUGUAAAAGAGUCGUCGGU	CUUAUAUUUUCUUAGCAGCCA	1.5	copine (calcium-dependent phospholipid-binding protein) family zinc ion binding
vvi-miR3629c-5p	VIT_206s0004g00450	2	GGAUGUAAAAGAGUCGUCGGU	UCUACAUUUUCUCAGCAGCCA	0.5	rna polymerase ii transcription mediator regulation of transcription from RNA polymerase II promoter
vvi-miR3629c-5p	VIT_207s0031g01230	2	GGAUGUAAAAGAGUCGUCGGU	UCCUCAUUUUCUCAGCAGCCA	2.5	60s ribosomal protein ldd structural constituent of ribosome
vvi-miR3629c-5p	VIT_208s0007g06820	1	GGAUGUAAAAGAGUCGUCGGU	CCUACAUUUUCUCGGUAGACA	2	kinesin motor protein microtubule-based movement
vvi-miR3629c-5p	VIT_208s0007g06820	2	GGAUGUAAAAGAGUCGUCGGU	GCUGCAUUUUCUCAGCAACCA	2.5	kinesin motor protein microtubule-based movement
vvi-miR3629c-5p	VIT_209s0002g03240	5	GGAUGUAAAAGAGUCGUCGGU	UCUACACUUUCUCAGCAACCA	2.5	uncharacterized protein mitochondrion
vvi-miR3629c-5p	VIT_209s0054g00440	1	GGAUGUAAAAGAGUCGUCGGU	CCUCAAUUUUCUCAGCAGCCA	2	gata transcription factor dd sequence-specific DNA binding transcription factor activity
vvi-miR3629c-5p	VIT_211s0016g03380	1	GGAUGUAAAAGAGUCGUCGGU	CCUGCACUUUCUCAGCAACCA	2.5	probable glycosyltransferase at5g03795-like glucuronosyl-N-acetylglucosaminyl-proteoglycan 4-alpha-N-acetylglucosaminyltransferase activity
vvi-miR3629c-5p	VIT_211s0016g04820	5	GGAUGUAAAAGAGUCGUCGGU	CCGACAUUUUCUCAGCAGCCA	1	tudor-like rna-binding protein RNA binding
vvi-miR3629c-5p	VIT_211s0065g00340	2	GGAUGUAAAAGAGUCGUCGGU	UCCACAUUUUCUCAGCAGCCA	1.5	hua enhancer 2 sugar mediated signaling pathway
vvi-miR3629c-5p	VIT_212s0035g01020	1	GGAUGUAAAAGAGUCGUCGGU	CUUACUUUUUCUCAGCAGCCG	2	60s ribosomal protein nucleolus
vvi-miR3629c-5p	VIT_213s0019g04380	1	GGAUGUAAAAGAGUCGUCGGU	UCUACAUUUUCUUGGCACCCA	2.5	protein developmental growth
vvi-miR3629c-5p	VIT_213s0047g01200	1	GGAUGUAAAAGAGUCGUCGGU	CCUCCAUUUUCUCAGCAACCA	2	obtusifoliol 14alpha-demethylase methionine
vvi-miR3629c-5p	VIT_213s0064g00580	4	GGAUGUAAAAGAGUCGUCGGU	CCUACAUUUUCUCGGCAGCCA	0.5	dd kda peptidyl-prolyl isomerase protein folding
vvi-miR3629c-5p	VIT_213s0073g00550	1	GGAUGUAAAAGAGUCGUCGGU	CCUGCAUUUUCUCAGCAACUA	2	lysosomal beta glucosidase-like anchored to membrane
vvi-miR3629c-5p	VIT_214s0060g01430	3	GGAUGUAAAAGAGUCGUCGGU	UUUAUAUUUUCUCAGCAGCCA	1.5	uncharacterized protein single-organism cellular process
vvi-miR3629c-5p	VIT_214s0060g01580	1	GGAUGUAAAAGAGUCGUCGGU	CCUGCAUUUUCUCGGCAACCG	2.5	protein plasma membrane
vvi-miR3629c-5p	VIT_214s0066g00830	2	GGAUGUAAAAGAGUCGUCGGU	CCUGCAUUUUCUCACCAACCA	2.5	sirohydrochlorin ferrochelatase metal ion binding

miRNA name	Target gene	Number of target sites	miRNA sequence	Target sequence	Score	Target annotation
vvi-miR3629c-5p	VIT_215s0046g03190	1	GGAUGUAAAAGAGUCGUCGGU	CCUGCAUUUUCUCAGCUGCCA	1.5	r2r3-myb transcription response to jasmonic acid stimulus
vvi-miR3629c-5p	VIT_216s0039g01420	1	GGAUGUAAAAGAGUCGUCGGU	CCUCCAUUUUCUUGGCAGCCA	2	nucleotide-diphospho-sugar transferases superfamily protein pollen germination
vvi-miR3629c-5p	VIT_216s0039g02430	2	GGAUGUAAAAGAGUCGUCGGU	CCUGCAAUUUCUCAGCAACCA	2.5	uncharacterized protein single-organism cellular process
vvi-miR3629c-5p	VIT_216s0050g01780	1	GGAUGUAAAAGAGUCGUCGGU	CUUCCAUUUUCUCAGCGGCCA	2	probable membrane-associated kinase regulator 2-like response to stimulus
vvi-miR3629c-5p	VIT_216s0098g01870	6	GGAUGUAAAAGAGUCGUCGGU	CCUACAUUUUUUCAGCAGCCA	0.5	calcineurin b-like protein 4 hypotonic salinity response
vvi-miR3629c-5p	VIT_216s0098g01880	3	GGAUGUAAAAGAGUCGUCGGU	CCUACAUUUUCUAAGCAACCA	2	dipeptidyl peptidase cytosol
vvi-miR3629c-5p	VIT_217s0000g01150	1	GGAUGUAAAAGAGUCGUCGGU	CCUACAUUUUCUCAGCAGCCA	0	curved DNA-binding protein protein folding
vvi-miR3632-3p	VIT_211s0016g05350	1	AACCAUAACCCCCAGACCCUUU	UUGGUAUUCGGGGUCUGGGAAA	1	protein Rab GTPase activator activity
vvi-miR3632-3p	VIT_213s0064g00550	1	AACCAUAACCCCCAGACCCUUU	UUGGUAUUGGGGGUUUAGGUAA	2.5	disease resistance protein rga4-like defense response
vvi-miR3632-3p	VIT_213s0067g00790	1	AACCAUAACCCCCAGACCCUUU	AUGGUAUGGGGGGUCUGGGAAA	2	disease resistance protein defense response
vvi-miR3632-3p	VIT_213s0067g00830	4	AACCAUAACCCCCAGACCCUUU	AUGGUAUGGGGGGUCUGGGAAA	2	probable disease resistance rpp8-like protein 2-like defense response
vvi-miR3633a-3p	VIT_214s0036g00100	1	AUCCCUUACCCACCAUAUCCUU	UGGGGGAUGGGUGGUGUGGGGA	2.5	disease resistance protein at4g27190-like defense response
vvi-miR3633a-5p	VIT_201s0026g02650	8	GAGAGGAUUGGUAGGUAAGG	CUGUCCUAACCAUCCAUUUU	2	kh domain-containing protein at4g18375-like response to abscisic acid stimulus
vvi-miR3633a-5p	VIT_205s0051g00590	2	GAGAGGAUUGGUAGGUAAGG	CUCUCUUCACCAUCCACUCC	2.5	pectate lyase extracellular region
vvi-miR3633a-5p	VIT_207s0031g01910	1	GAGAGGAUUGGUAGGUAAGG	CUUUCCUGACCAUCCAUACU	2.5	protein nucleus
vvi-miR3633a-5p	VIT_213s0074g00440	4	GAGAGGAUUGGUAGGUAAGG	CUCUCCUCCCCAUCCAUUUC	2.5	methyltransferase-like protein 13-like
vvi-miR3633a-5p	VIT_218s0001g07660	6	GAGAGGAUUGGUAGGUAAGG	CUCUCCCAAUCCUCCAUUCC	2.5	hydroxyproline-rich glycoprotein family protein RNA binding
vvi-miR3633b-5p	VIT_205s0020g04900	2	UCUAGGGUCGGUGGGUAAGG	AGAACUUAGCCACCUAUUCC	2.5	n-carbamoyl-l-amino acid hydrolase allantoin catabolic process
vvi-miR3633b-5p	VIT_210s0003g03540	1	UCUAGGGUCGGUGGGUAAGG	AGAUUUCAGCCACCCAUUAC	2	beta-amyrin synthase
vvi-miR3633b-5p	VIT_212s0034g02400	1	UCUAGGGUCGGUGGGUAAGG	GGAUCCCA-CCAUCCAUUCC	2.5	disease resistance protein at3g14460-like defense response
vvi-miR3633b-5p	VIT_212s0034g02440	1	UCUAGGGUCGGUGGGUAAGG	AGAUCUCA-CCAUCCAUUCC	2.5	disease resistance protein at3g14460-like defense response
vvi-miR3633b-5p	VIT_212s0034g02470	1	UCUAGGGUCGGUGGGUAAGG	AGAUCCCA-CCAUCCAUUCC	2	disease resistance protein at3g14460-like defense response
vvi-miR3633b-5p	VIT_216s0050g00050	1	UCUAGGGUCGGUGGGUAAGG	AGACCCCAGCUACCCUUUCC	2.5	myb transcription factor DNA binding
vvi-miR3633b-5p	VIT_217s0000g00950	2	UCUAGGGUCGGUGGGUAAGG	AGAUUCCAGCCACCAAUACC	2.5	hydroxycinnamoyl shikimate quinate hydroxycinnamoyltransferase-like protein nitrate transport

miRNA name	Target gene	Number of target sites	miRNA sequence	Target sequence	Score	Target annotation
vvi-miR3634-3p	VIT_219s0085g00430	1	UGCCGUACUCACGCUCAGCCUUU	AUGGCAUGAGUG-GAGUCGGAAA	2	hypothetical protein
vvi-miR3634-3p	VIT_219s0085g00470	1	UGCCGUACUCACGCUCAGCCUUU	AUGGCAUGAGUG-GAGUCGGAAA	2	hypothetical protein
vvi-miR3635-3p	VIT_217s0000g05850	5	CUCCGUACACACCCUGUAUUA	GAGGCAUGUGUGGGACAUAAU	0	abc transporter retinal flippase subfamily plastid
vvi-miR3635-3p	VIT_218s0041g02230	1	CUCCGUACACACCCUGUAUUA	GAGGCAUGUGUGGGACAUAAU	0	hypothetical protein
vvi-miR390-5p	VIT_206s0004g01080	1	CCGCGAUAGGGAGGACUCGAA	GGUGUUAUUCCUCUUGAGUUU	2.5	dna (cytosine-5)-methyltransferase DNA methylation on cytosine within a CNG sequence
vvi-miR390-5p	VIT_210s0003g01890	1	CCGCGAUAGGGAGGACUCGAA	GGCGUUCUUCCUCCUGAGCUU	2	lrr receptor-like serine threonine-protein kinase rfk1 protein phosphorylation
vvi-miR390-5p	VIT_212s0059g01410	1	CCGCGAUAGGGAGGACUCGAA	GGCGAUAUCUCUCCUGAGCUU	1.5	hypothetical protein
vvi-miR390-5p	VIT_216s0098g01090	1	CCGCGAUAGGGAGGACUCGAA	GGCUCUAUACCUCCUGAGCUU	2	leucine-rich repeat receptor-like protein kinase pepr1 defense response
vvi-miR393a-5p	VIT_206s0061g00780	1	CUAGUUACGCUAGGGAAACCU	GAACAAUGUGAUCUCUUUGGA	2	protein sensitive to proton rhizotoxicity 1-like nitrate transport
vvi-miR393a-5p	VIT_218s0001g02460	1	CUAGUUACGCUAGGGAAACCU	GAUCAGUGC-AUUCCUUUGGA	2.5	uncharacterized protein loc1002579dd isoform 2 membrane
vvi-miR393b-5p	VIT_206s0061g00780	1	CUAGUUACGCUAGGGAAACCU	GAACAAUGUGAUCUCUUUGGA	2	protein sensitive to proton rhizotoxicity 1-like nitrate transport
vvi-miR393b-5p	VIT_218s0001g02460	1	CUAGUUACGCUAGGGAAACCU	GAUCAGUGC-AUUCCUUUGGA	2.5	uncharacterized protein loc1002579dd isoform 2 membrane
vvi-miR394a-5p	VIT_201s0010g03730	2	CCUCCACCUGUCUUACGGUU	GGAGGUUGACAGAAUGCCAA	1	f-box family protein regulation of auxin mediated signaling pathway
vvi-miR394a-5p	VIT_202s0025g03200	1	CCUCCACCUGUCUUACGGUU	GGAAGUGGACAGAGUGCUGA	2.5	dehydration-responsive family protein pollen exine formation
vvi-miR394a-5p	VIT_204s0008g02510	3	CCUCCACCUGUCUUACGGUU	GGAGGAGGAGAGAAUGCCAA	2	g-type lectin s-receptor-like serine threonine-protein kinase at1g34300-like myosin light chain kinase activity
vvi-miR394a-5p	VIT_204s0008g02520	2	CCUCCACCUGUCUUACGGUU	GGAGGAGGAGAGAAUGCCAA	2	g-type lectin s-receptor-like serine threonine-protein kinase at1g34300-like myosin light chain kinase activity
vvi-miR394a-5p	VIT_205s0049g00850	1	CCUCCACCUGUCUUACGGUU	GGAGGUGGCCGGAAUGGCAA	2.5	activating signal cointegrator 1-like mitochondrion
vvi-miR394b-5p	VIT_201s0010g03730	2	CCUCCACCUGUCUUACGGUU	GGAGGUUGACAGAAUGCCAA	1	f-box family protein regulation of auxin mediated signaling pathway
vvi-miR394b-5p	VIT_202s0025g03200	1	CCUCCACCUGUCUUACGGUU	GGAAGUGGACAGAGUGCUGA	2.5	dehydration-responsive family protein pollen exine formation

miRNA name	Target gene	Number of target sites	miRNA sequence	Target sequence	Score	Target annotation
vvi-miR394b-5p	VIT_204s0008g02510	3	CCUCCACCUGUCUUACGGUU	GGAGGAGGAGAGAAUGCCAA	2	g-type lectin s-receptor-like serine threonine-protein kinase at1g34300-like myosin light chain kinase activity
vvi-miR394b-5p	VIT_204s0008g02520	2	CCUCCACCUGUCUUACGGUU	GGAGGAGGAGAGAAUGCCAA	2	g-type lectin s-receptor-like serine threonine-protein kinase at1g34300-like myosin light chain kinase activity
vvi-miR394b-5p	VIT_205s0049g00850	1	CCUCCACCUGUCUUACGGUU	GGAGGUGGCCGGAAUGGCAA	2.5	activating signal cointegrator 1-like mitochondrion
vvi-miR395a-3p	VIT_205s0020g04210	1	CUCAAGGGGGUUUGUGAAGUC	GAGUUCCUCCAAACUCUUCAU	2.5	atp sulfurylase water transport
vvi-miR395a-3p	VIT_207s0031g00940	2	CUCAAGGGGGUUUGUGAAGUC	AAGUUCUCCCAAACACUUCAA	2.5	sulfate bicarbonate oxalate exchanger and transporter sat-1 integral to membrane
vvi-miR395a-3p	VIT_212s0059g00760	4	CUCAAGGGGGUUUGUGAAGUC	GAGUUCCCCUACAAACUUCAG	2.5	prhdd protein phosphoadenylyl-sulfate reductase (thioredoxin) activity
vvi-miR395a-3p	VIT_218s0001g04890	1	CUCAAGGGGGUUUGUGAAGUC	GAGCUCUCCCAAACACUUCAG	1.5	low affinity sulfate transporter 3-like integral to membrane
vvi-miR395c-3p	VIT_205s0020g04210	1	CUCAAGGGGGUUUGUGAAGUC	GAGUUCCUCCAAACUCUUCAU	2.5	atp sulfurylase water transport
vvi-miR395c-3p	VIT_207s0031g00940	2	CUCAAGGGGGUUUGUGAAGUC	AAGUUCUCCCAAACACUUCAA	2.5	sulfate bicarbonate oxalate exchanger and transporter sat-1 integral to membrane
vvi-miR395c-3p	VIT_212s0059g00760	4	CUCAAGGGGGUUUGUGAAGUC	GAGUUCCCCUACAAACUUCAG	2.5	prhdd protein phosphoadenylyl-sulfate reductase (thioredoxin) activity
vvi-miR395c-3p	VIT_218s0001g04890	1	CUCAAGGGGGUUUGUGAAGUC	GAGCUCUCCCAAACACUUCAG	1.5	low affinity sulfate transporter 3-like integral to membrane
vvi-miR395c-5p	VIT_214s0066g00910	1	GUCACUUCACCAGUUCCCUUG	UGGUGAAGUGUUCAAGGGAAC	2	protein plasma membrane
vvi-miR395d-3p	VIT_205s0020g04210	1	CUCAAGGGGGUUUGUGAAGUC	GAGUUCCUCCAAACUCUUCAU	2.5	atp sulfurylase water transport
vvi-miR395d-3p	VIT_207s0031g00940	2	CUCAAGGGGGUUUGUGAAGUC	AAGUUCUCCCAAACACUUCAA	2.5	sulfate bicarbonate oxalate exchanger and transporter sat-1 integral to membrane
vvi-miR395d-3p	VIT_212s0059g00760	4	CUCAAGGGGGUUUGUGAAGUC	GAGUUCCCCUACAAACUUCAG	2.5	prhdd protein phosphoadenylyl-sulfate reductase (thioredoxin) activity
vvi-miR395d-3p	VIT_218s0001g04890	1	CUCAAGGGGGUUUGUGAAGUC	GAGCUCUCCCAAACACUUCAG	1.5	low affinity sulfate transporter 3-like integral to membrane
vvi-miR395e-3p	VIT_205s0020g04210	1	CUCAAGGGGGUUUGUGAAGUC	GAGUUCCUCCAAACUCUUCAU	2.5	atp sulfurylase water transport
vvi-miR395e-3p	VIT_207s0031g00940	2	CUCAAGGGGGUUUGUGAAGUC	AAGUUCUCCCAAACACUUCAA	2.5	sulfate bicarbonate oxalate exchanger and transporter sat-1 integral to membrane
vvi-miR395e-3p	VIT_212s0059g00760	4	CUCAAGGGGGUUUGUGAAGUC	GAGUUCCCCUACAAACUUCAG	2.5	prhdd protein phosphoadenylyl-sulfate reductase (thioredoxin) activity
vvi-miR395e-3p	VIT_218s0001g04890	1	CUCAAGGGGGUUUGUGAAGUC	GAGCUCUCCCAAACACUUCAG	1.5	low affinity sulfate transporter 3-like integral to membrane
vvi-miR395e-5p	VIT_214s0066g00910	1	GUCACUUCACCAGUUCCCUUG	UGGUGAAGUGUUCAAGGGAAC	2	protein plasma membrane
vvi-miR395f-3p	VIT_205s0020g04210	1	CUCAAGGGGGUUUGUGAAGUC	GAGUUCCUCCAAACUCUUCAU	2.5	atp sulfurylase water transport

miRNA name	Target gene	Number of target sites	miRNA sequence	Target sequence	Score	Target annotation
vvi-miR395f-3p	VIT_207s0031g00940	2	CUCAAGGGGGUUUGUGAAGUC	AAGUUCUCCCAAACACUUCAA	2.5	sulfate bicarbonate oxalate exchanger and transporter sat-1 integral to membrane
vvi-miR395f-3p	VIT_212s0059g00760	4	CUCAAGGGGGUUUGUGAAGUC	GAGUUCCCCUACAAACUUCAG	2.5	prhdd protein phosphoadenylyl-sulfate reductase (thioredoxin) activity
vvi-miR395f-3p	VIT_218s0001g04890	1	CUCAAGGGGGUUUGUGAAGUC	GAGCUCUCCCAAACACUUCAG	1.5	low affinity sulfate transporter 3-like integral to membrane
vvi-miR395h-3p	VIT_205s0020g04210	1	CUCAAGGGGGUUUGUGAAGUC	GAGUUCCUCCAAACUCUUCAU	2.5	atp sulfurylase water transport
vvi-miR395h-3p	VIT_207s0031g00940	2	CUCAAGGGGGUUUGUGAAGUC	AAGUUCUCCCAAACACUUCAA	2.5	sulfate bicarbonate oxalate exchanger and transporter sat-1 integral to membrane
vvi-miR395h-3p	VIT_212s0059g00760	4	CUCAAGGGGGUUUGUGAAGUC	GAGUUCCCCUACAAACUUCAG	2.5	prhdd protein phosphoadenylyl-sulfate reductase (thioredoxin) activity
vvi-miR395h-3p	VIT_218s0001g04890	1	CUCAAGGGGGUUUGUGAAGUC	GAGCUCUCCCAAACACUUCAG	1.5	low affinity sulfate transporter 3-like integral to membrane
vvi-miR395h-5p	VIT_214s0066g00910	1	GUCACUUCACCAGUUCCCUUG	UGGUGAAGUGUUCAAGGGAAC	2	protein plasma membrane
vvi-miR395i-3p	VIT_205s0020g04210	1	CUCAAGGGGGUUUGUGAAGUC	GAGUUCCUCCAAACUCUUCAU	2.5	atp sulfurylase water transport
vvi-miR395i-3p	VIT_207s0031g00940	2	CUCAAGGGGGUUUGUGAAGUC	AAGUUCUCCCAAACACUUCAA	2.5	sulfate bicarbonate oxalate exchanger and transporter sat-1 integral to membrane
vvi-miR395i-3p	VIT_212s0059g00760	4	CUCAAGGGGGUUUGUGAAGUC	GAGUUCCCCUACAAACUUCAG	2.5	prhdd protein phosphoadenylyl-sulfate reductase (thioredoxin) activity
vvi-miR395i-3p	VIT_218s0001g04890	1	CUCAAGGGGGUUUGUGAAGUC	GAGCUCUCCCAAACACUUCAG	1.5	low affinity sulfate transporter 3-like integral to membrane
vvi-miR395j-3p	VIT_205s0020g04210	1	CUCAAGGGGGUUUGUGAAGUC	GAGUUCCUCCAAACUCUUCAU	2.5	atp sulfurylase water transport
vvi-miR395j-3p	VIT_207s0031g00940	2	CUCAAGGGGGUUUGUGAAGUC	AAGUUCUCCCAAACACUUCAA	2.5	sulfate bicarbonate oxalate exchanger and transporter sat-1 integral to membrane
vvi-miR395j-3p	VIT_212s0059g00760	4	CUCAAGGGGGUUUGUGAAGUC	GAGUUCCCCUACAAACUUCAG	2.5	prhdd protein phosphoadenylyl-sulfate reductase (thioredoxin) activity
vvi-miR395j-3p	VIT_218s0001g04890	1	CUCAAGGGGGUUUGUGAAGUC	GAGCUCUCCCAAACACUUCAG	1.5	low affinity sulfate transporter 3-like integral to membrane
vvi-miR395k-3p	VIT_205s0020g04210	1	CUCAAGGGGGUUUGUGAAGUC	GAGUUCCUCCAAACUCUUCAU	2.5	atp sulfurylase water transport
vvi-miR395k-3p	VIT_207s0031g00940	2	CUCAAGGGGGUUUGUGAAGUC	AAGUUCUCCCAAACACUUCAA	2.5	sulfate bicarbonate oxalate exchanger and transporter sat-1 integral to membrane
vvi-miR395k-3p	VIT_212s0059g00760	4	CUCAAGGGGGUUUGUGAAGUC	GAGUUCCCCUACAAACUUCAG	2.5	prhdd protein phosphoadenylyl-sulfate reductase (thioredoxin) activity
vvi-miR395k-3p	VIT_218s0001g04890	1	CUCAAGGGGGUUUGUGAAGUC	GAGCUCUCCCAAACACUUCAG	1.5	low affinity sulfate transporter 3-like integral to membrane

miRNA name	Target gene	Number of target sites	miRNA sequence	Target sequence	Score	Target annotation
vvi-miR395k-5p	VIT_214s0066g00910	1	GUCACUUCACCAGUUCCCUUG	UGGUGAAGUGUUCAAGGGAAC	2	protein plasma membrane
vvi-miR395l-3p	VIT_205s0020g04210	1	CUCAAGGGGGUUUGUGAAGUC	GAGUUCCUCCAAACUCUUCAU	2.5	atp sulfurylase water transport
vvi-miR395l-3p	VIT_207s0031g00940	2	CUCAAGGGGGUUUGUGAAGUC	AAGUUCUCCCAAACACUUCAA	2.5	sulfate bicarbonate oxalate exchanger and transporter sat-1 integral to membrane
vvi-miR395l-3p	VIT_212s0059g00760	4	CUCAAGGGGGUUUGUGAAGUC	GAGUUCCCCUACAAACUUCAG	2.5	prhdd protein phosphoadenylyl-sulfate reductase (thioredoxin) activity
vvi-miR395l-3p	VIT_218s0001g04890	1	CUCAAGGGGGUUUGUGAAGUC	GAGCUCUCCCAAACACUUCAG	1.5	low affinity sulfate transporter 3-like integral to membrane
vvi-miR396a-5p	VIT_202s0012g02250	7	AUCAAGUUCUUUCGACACCUU	UGGUUUGAGAAAGUUGUGGAA	2	transcription factor hbp-1a sequence-specific DNA binding transcription factor activity
vvi-miR396b-5p	VIT_207s0191g00220	2	UUCAAGUUCUUUCGACACCUU	AAACUCAAGGAAGCUGUGGAA	2.5	hypothetical protein
vvi-miR396b-5p	VIT_211s0016g05580	5	UUCAAGUUCUUUCGACACCUU	AAGUUCAAGAAAACUGUGGUA	2	methyl binding domain protein nucleus
vvi-miR396c-5p	VIT_202s0012g02250	7	GUCAAGUUCUUUCGACACCUU	UGGUUUGAGAAAGUUGUGGAA	2.5	transcription factor hbp-1a sequence-specific DNA binding transcription factor activity
vvi-miR396c-5p	VIT_204s0008g00915	1	GUCAAGUUCUUUCGACACCUU	CAGUUCAGGAAGGUUUUGGAA	2.5	receptor-like protein kinase hsl1-like plasma membrane
vvi-miR396d-3p	VIT_215s0024g00350	1	GAAGGGUGUCGAAAUAACUUG	CUUUCCAUAGUUUUGUUGAAC	2	tata box binding protein associated factor-like protein helicase activity
vvi-miR396d-5p	VIT_202s0012g02250	7	UCAAGUUCUUUCGACACCUU	GGUUUGAGAAAGUUGUGGAA	2	transcription factor hbp-1a sequence-specific DNA binding transcription factor activity
vvi-miR396d-5p	VIT_202s0033g01260	1	UCAAGUUCUUUCGACACCUU	UGUUCAAGAAGGCUGUGGCA	2.5	uncharacterized protein loc1002569dd oxidation-reduction process
vvi-miR396d-5p	VIT_204s0008g00915	1	UCAAGUUCUUUCGACACCUU	AGUUCAGGAAGGUUUUGGAA	2.5	receptor-like protein kinase hsl1-like plasma membrane
vvi-miR396d-5p	VIT_206s0009g02740	1	UCAAGUUCUUUCGACACCUU	AGUUCAAGAAAGCUUUGAAG	2.5	pentatricopeptide repeat-containing protein chloroplastic-like protein binding
vvi-miR396d-5p	VIT_207s0191g00220	2	UCAAGUUCUUUCGACACCUU	AACUCAAGGAAGCUGUGGAA	2.5	hypothetical protein
vvi-miR396d-5p	VIT_211s0016g05580	5	UCAAGUUCUUUCGACACCUU	AGUUCAAGAAAACUGUGGUA	2	methyl binding domain protein nucleus
vvi-miR397a-5p	VIT_204s0023g01960	7	GUAGUUGCGACGUGAGUUACU	CAUCAAUGCUGCUCUCAACGA	2.5	laccase (diphenol oxidase)-like protein copper ion binding
vvi-miR397a-5p	VIT_204s0069g00950	2	GUAGUUGCGACGUGAGUUACU	UGUCAAUGCUGCACUCAAUGA	1.5	laccase (diphenol oxidase)-like protein copper ion binding
vvi-miR397a-5p	VIT_206s0004g03760	2	GUAGUUGCGACGUGAGUUACU	CAUCAAUGCGGCACUCAAUGA	1.5	protein copper ion binding
vvi-miR397a-5p	VIT_206s0004g04050	2	GUAGUUGCGACGUGAGUUACU	GAUCAACGCAGCGCUCAAUGA	2.5	protein copper ion binding
vvi-miR397a-5p	VIT_206s0004g06090	1	GUAGUUGCGACGUGAGUUACU	CAUCAACGCUGCGCUGAAUGA	1.5	laccase 1a copper ion binding
vvi-miR397a-5p	VIT_208s0007g00600	2	GUAGUUGCGACGUGAGUUACU	GAUCAACGCUGCACUCAACGA	2	laccase 110b copper ion binding
vvi-miR397a-5p	VIT_208s0007g00670	1	GUAGUUGCGACGUGAGUUACU	AAUCAAUGCUGCACUCAAUGA	1.5	laccase 110c response to water deprivation
vvi-miR397a-5p	VIT_208s0007g00680	1	GUAGUUGCGACGUGAGUUACU	GAUCAAUGCAGCACUCAAUGA	2.5	laccase 110c response to water deprivation
vvi-miR397a-5p	VIT_208s0007g01550	1	GUAGUUGCGACGUGAGUUACU	AAUUAACGCUGCACUCAAUAA	2.5	laccase 110a copper ion binding
vvi-miR397a-5p	VIT_208s0007g01910	2	GUAGUUGCGACGUGAGUUACU	CAUUAACGCUGCACUCAAUGA	0.5	laccase 1a glucuronoxylan

miRNA name	Target gene	Number of target sites	miRNA sequence	Target sequence	Score	Target annotation
vvi-miR397a-5p	VIT_208s0007g06460	5	GUAGUUGCGACGUGAGUUACU	UGUCAAUGCUGCACUCAACGA	2.5	laccase (diphenol oxidase)-like protein copper ion binding
vvi-miR397a-5p	VIT_208s0040g01790	5	GUAGUUGCGACGUGAGUUACU	CGUCAAUGCUGCACUCAAUGA	1	laccase (diphenol oxidase)-like protein copper ion binding
vvi-miR397a-5p	VIT_213s0019g01920	1	GUAGUUGCGACGUGAGUUACU	GAUCAAUGCCGCACUCAAUGA	2.5	laccase-dd hydroquinone:oxygen oxidoreductase activity
vvi-miR397a-5p	VIT_213s0019g01930	1	GUAGUUGCGACGUGAGUUACU	GAUCAACGCUGCACUCAAUGA	1	laccase-17-like [Vitis vinifera] hydroquinone:oxygen oxidoreductase activity
vvi-miR397a-5p	VIT_213s0019g01940	1	GUAGUUGCGACGUGAGUUACU	GAUCAAUGCUGCACUCAAUGA	1.5	laccase-dd hydroquinone:oxygen oxidoreductase activity
vvi-miR397a-5p	VIT_213s0019g02150	1	GUAGUUGCGACGUGAGUUACU	GAUCAAUGCUGCACUCAAUGA	1.5	laccase-dd hydroquinone:oxygen oxidoreductase activity
vvi-miR397a-5p	VIT_213s0019g02160	1	GUAGUUGCGACGUGAGUUACU	GAUCAAUGCUGCACUCAAUGA	1.5	laccase-dd hydroquinone:oxygen oxidoreductase activity
vvi-miR399a-3p	VIT_200s0265g00070	2	GUCCCGUUAAGAGGAAACCGU	UAGGGCAAAUCUCCUUUGGCA	1.5	probable ubiquitin-conjugating enzyme e2 24-like postreplication repair
vvi-miR399a-5p	VIT_200s0131g00355	1	AGACGGUUUCCUCUUAGUGUG	UUUGCCAAAUGAGAGUCGCAC	2.5	low quality protein: annexin d4-like response to abscisic acid stimulus
vvi-miR399b-3p	VIT_200s0265g00070	2	GUCCCGUUGAGAGGAAACCGU	UAGGGCAAAUCUCCUUUGGCA	1.5	probable ubiquitin-conjugating enzyme e2 24-like postreplication repair
vvi-miR399b-3p	VIT_213s0067g03280	1	GUCCCGUUGAGAGGAAACCGU	CUGGGCAACUCUUCUUUGGCU	2.5	high affinity inorganic phosphate transporter phosphate ion transport
vvi-miR399c-3p	VIT_200s0265g00070	2	GUCCCGUUGAGAGGAAACCGU	UAGGGCAAAUCUCCUUUGGCA	1.5	probable ubiquitin-conjugating enzyme e2 24-like postreplication repair
vvi-miR399c-3p	VIT_213s0067g03280	1	GUCCCGUUGAGAGGAAACCGU	CUGGGCAACUCUUCUUUGGCU	2.5	high affinity inorganic phosphate transporter phosphate ion transport
vvi-miR399e-3p	VIT_200s0265g00070	2	GGCCCGUUUAGAGGAAACCGU	AGGGGCAAAUCUCCUUUGGCA	2	probable ubiquitin-conjugating enzyme e2 24-like postreplication repair
vvi-miR399e-3p	VIT_200s0265g00070	2	GGCCCGUUUAGAGGAAACCGU	GGGGGCAAAUCUCCUUUGGCA	2	probable ubiquitin-conjugating enzyme e2 24-like postreplication repair
vvi-miR399e-3p	VIT_200s0265g00070	2	GGCCCGUUUAGAGGAAACCGU	GUGGGCAAAUCUCCUUUGGCA	2.5	probable ubiquitin-conjugating enzyme e2 24-like postreplication repair
vvi-miR399e-3p	VIT_200s0265g00070	2	GGCCCGUUUAGAGGAAACCGU	UAGGGCAAAUCUCCUUUGGCA	2.5	probable ubiquitin-conjugating enzyme e2 24-like postreplication repair

miRNA name	Target gene	Number of target sites	miRNA sequence	Target sequence	Score	Target annotation
vvi-miR399e-3p	VIT_200s0265g00070	2	GGCCCGUUUAGAGGAAACCGU	UUGGGCAAAUCUCCUUUGGCA	1	probable ubiquitin-conjugating enzyme e2 24-like postreplication repair
vvi-miR399h-3p	VIT_200s0265g00070	2	GUCCCGUUAAGAGGAAACCGU	UAGGGCAAAUCUCCUUUGGCA	1.5	probable ubiquitin-conjugating enzyme e2 24-like postreplication repair
vvi-miR399i-3p	VIT_200s0265g00070	2	GUCCCGUUGAGAGGAAACCGC	UAGGGCAAAUCUCCUUUGGCA	2.5	probable ubiquitin-conjugating enzyme e2 24-like postreplication repair
vvi-miR399i-3p	VIT_213s0067g03280	1	GUCCCGUUGAGAGGAAACCGC	CUGGGCAACUCUUCUUUGGCU	2.5	high affinity inorganic phosphate transporter phosphate ion transport
vvi-miR399i-3p	VIT_218s0166g00230	1	GUCCCGUUGAGAGGAAACCGC	CAGGGCAGC-CUCUUUUGGCG	2.5	g-type lectin s-receptor-like serine threonine-protein kinase rlk1-like transmembrane receptor protein tyrosine kinase activity
vvi-miR403a-3p	VIT_205s0077g00800	2	GCUCAAACACGCACUUAGAUU	CAAGUUUGUGCGUGAAUCAAA	2	gins complex subunit 4 regulation of cell cycle
vvi-miR403a-3p	VIT_207s0005g01690	1	GCUCAAACACGCACUUAGAUU	GGAGUUUGUGCGUGACUCUAA	2	hypothetical protein
vvi-miR403a-3p	VIT_210s0042g01150	1	GCUCAAACACGCACUUAGAUU	GGAGUUUGUGCGUGAAUCUAA	1	protein argonaute 2-like defense response to bacterium
vvi-miR403a-3p	VIT_210s0042g01180	2	GCUCAAACACGCACUUAGAUU	GGAGUUUGUGCGUGAAUCUAA	1	protein argonaute 2-like defense response to bacterium
vvi-miR403a-3p	VIT_210s0042g01200	2	GCUCAAACACGCACUUAGAUU	GGAGUUUGUGCGUGAAUCUAA	1	protein argonaute 2-like defense response to bacterium
vvi-miR403b-3p	VIT_205s0077g00800	2	GCUCAAACACGCACUUAGAUU	CAAGUUUGUGCGUGAAUCAAA	2	gins complex subunit 4 regulation of cell cycle
vvi-miR403b-3p	VIT_207s0005g01690	1	GCUCAAACACGCACUUAGAUU	GGAGUUUGUGCGUGACUCUAA	2	hypothetical protein
vvi-miR403b-3p	VIT_210s0042g01150	1	GCUCAAACACGCACUUAGAUU	GGAGUUUGUGCGUGAAUCUAA	1	protein argonaute 2-like defense response to bacterium
vvi-miR403b-3p	VIT_210s0042g01180	2	GCUCAAACACGCACUUAGAUU	GGAGUUUGUGCGUGAAUCUAA	1	protein argonaute 2-like defense response to bacterium
vvi-miR403b-3p	VIT_210s0042g01200	2	GCUCAAACACGCACUUAGAUU	GGAGUUUGUGCGUGAAUCUAA	1	protein argonaute 2-like defense response to bacterium
vvi-miR403d-3p	VIT_205s0077g00800	2	GCUCAAACACGCACUUAGAUU	CAAGUUUGUGCGUGAAUCAAA	2	gins complex subunit 4 regulation of cell cycle
vvi-miR403d-3p	VIT_207s0005g01690	1	GCUCAAACACGCACUUAGAUU	GGAGUUUGUGCGUGACUCUAA	2	hypothetical protein
vvi-miR403d-3p	VIT_210s0042g01150	1	GCUCAAACACGCACUUAGAUU	GGAGUUUGUGCGUGAAUCUAA	1	protein argonaute 2-like defense response to bacterium
vvi-miR403d-3p	VIT_210s0042g01180	2	GCUCAAACACGCACUUAGAUU	GGAGUUUGUGCGUGAAUCUAA	1	protein argonaute 2-like defense response to bacterium
vvi-miR403d-3p	VIT_210s0042g01200	2	GCUCAAACACGCACUUAGAUU	GGAGUUUGUGCGUGAAUCUAA	1	protein argonaute 2-like defense response to bacterium
vvi-miR403e-3p	VIT_205s0077g00800	2	GCUCAAACACGCACUUAGAUU	CAAGUUUGUGCGUGAAUCAAA	2	gins complex subunit 4 regulation of cell cycle
vvi-miR403e-3p	VIT_207s0005g01690	1	GCUCAAACACGCACUUAGAUU	GGAGUUUGUGCGUGACUCUAA	2	hypothetical protein
vvi-miR403e-3p	VIT_210s0042g01150	1	GCUCAAACACGCACUUAGAUU	GGAGUUUGUGCGUGAAUCUAA	1	protein argonaute 2-like defense response to bacterium

miRNA name	Target gene	Number of target sites	miRNA sequence	Target sequence	Score	Target annotation
vvi-miR403e-3p	VIT_210s0042g01180	2	GCUCAAACACGCACUUAGAUU	GGAGUUUGUGCGUGAAUCUAA	1	protein argonaute 2-like defense response to bacterium
vvi-miR403e-3p	VIT_210s0042g01200	2	GCUCAAACACGCACUUAGAUU	GGAGUUUGUGCGUGAAUCUAA	1	protein argonaute 2-like defense response to bacterium
vvi-miR403f-3p	VIT_205s0077g00800	2	GCUCAAACACGCACUUAGAUU	CAAGUUUGUGCGUGAAUCAAA	2	gins complex subunit 4 regulation of cell cycle
vvi-miR403f-3p	VIT_207s0005g01690	1	GCUCAAACACGCACUUAGAUU	GGAGUUUGUGCGUGACUCUAA	2	hypothetical protein
vvi-miR403f-3p	VIT_210s0042g01150	1	GCUCAAACACGCACUUAGAUU	GGAGUUUGUGCGUGAAUCUAA	1	protein argonaute 2-like defense response to bacterium
vvi-miR403f-3p	VIT_210s0042g01180	2	GCUCAAACACGCACUUAGAUU	GGAGUUUGUGCGUGAAUCUAA	1	protein argonaute 2-like defense response to bacterium
vvi-miR403f-3p	VIT_210s0042g01200	2	GCUCAAACACGCACUUAGAUU	GGAGUUUGUGCGUGAAUCUAA	1	protein argonaute 2-like defense response to bacterium
vvi-miR403f-5p	VIT_207s0005g01690	1	AAAAUCUCAGUGCGUGUUUGA	UCUUAGAUUCACGCACAAACU	2	hypothetical protein
vvi-miR408-3p	VIT_203s0063g00990	1	CGGUCCCUUCUCCGUCACGUA	GCUAGGGAAGAUGCAGUGCUU	2.5	uncharacterized protein loc1002555dd copper ion binding
vvi-miR408-3p	VIT_203s0063g01010	1	CGGUCCCUUCUCCGUCACGUA	GCUAGGGAAGAUGCAGUGCUU	2.5	uncharacterized protein loc1002486dd copper ion binding
vvi-miR408-3p	VIT_207s0005g02730	3	CGGUCCCUUCUCCGUCACGUA	GCCAGGGAAGAGGCAGUGCAU	0	protein DNA binding
vvi-miR408-5p	VIT_207s0005g02720	1	GGUACGUGAUGGAGCAGGGGC	CCAUGCACUGCCUCUUCCCUG	2	hypothetical protein
vvi-miR408-5p	VIT_207s0005g02730	3	GGUACGUGAUGGAGCAGGGGC	CCAUGCACUACCUCGUCCCCG	0	protein DNA binding
vvi-miR408-5p	VIT_208s0007g05590	1	GGUACGUGAUGGAGCAGGGGC	CCAUGCACUACCACGUCCCCG	1	beta-8 tubulin plastid
vvi-miR408-5p	VIT_218s0041g01550	1	GGUACGUGAUGGAGCAGGGGC	CCAUGCACUAACACGUCCCCG	2	beta chain cytoskeleton organization
vvi-miR477b-5p	VIT_201s0010g02270	1	GUCUUCGGGAACUCCCUCUCA	UGGAAGCCUUUGAGGGAGAGA	2.5	gras family transcription factor regulation of transcription
vvi-miR477b-5p	VIT_204s0008g06260	5	GUCUUCGGGAACUCCCUCUCA	CAGAAGCCUUUGAAGAAGAGU	2.5	exosome complex exonuclease rrpdd RNA methylation
vvi-miR477b-5p	VIT_208s0058g01400	3	GUCUUCGGGAACUCCCUCUCA	CAGAAGCCCUUGAAGGGGAGA	2.5	aminophospholipid atpase integral to membrane
vvi-miR477b-5p	VIT_217s0000g10300	1	GUCUUCGGGAACUCCCUCUCA	UGGAAGCCUUUGAGGGAGAGA	2.5	gras family transcription factor regulation of transcription
vvi-miR479-3p	VIT_218s0001g03310	1	CUCACUAUAACCAAGCCGAGC	GGGUGAUAUUGGUUCGGCUCA	1.5	gras family protein nuclear membrane
vvi-miR482-3p	VIT_202s0012g02720	1	CCUUACCCUCCUCAUCCUUUCU	GGGAUGGGGGGACUAGGGAAGA	2.5	nbs-lrr resistance protein defense response
vvi-miR482-3p	VIT_218s0072g01090	1	CCUUACCCUCCUCAUCCUUUCU	GGAAUAGGAGGGGUAGGAAAGA	1.5	hypothetical protein
vvi-miR482-5p	VIT_200s1944g00010	1	UUCGAAAGGAUGAGAGGUUAA	AAGCUUUUCUACUCUUUAAUG	2.5	pentatricopeptide repeat-containing protein embryo development ending in seed dormancy

miRNA name	Target gene	Number of target sites	miRNA sequence	Target sequence	Score	Target annotation
vvi-miR482-5p	VIT_200s2304g00010	1	UUCGAAAGGAUGAGAGGUUAA	AAGCUUUUCUACUCUUUAAUG	2.5	pentatricopeptide repeat-containing protein embryo development ending in seed dormancy
vvi-miR482-5p	VIT_200s2397g00010	1	UUCGAAAGGAUGAGAGGUUAA	AAGCUUUUCUACUCUUUAAUG	2.5	pentatricopeptide repeat-containing protein embryo development ending in seed dormancy
vvi-miR482-5p	VIT_208s0007g03550	1	UUCGAAAGGAUGAGAGGUUAA	AAGUUUUCUUACUUUUCAGUU	2.5	solute carrier family dd member f1-like cytoplasmic membrane-bounded vesicle
vvi-miR535a-5p	VIT_200s0358g00010	1	CGCACGAGAGAGAGCAACAGU	GCGUGCUCUCUCUCGCUGUCA	1	hypothetical protein
vvi-miR535a-5p	VIT_207s0104g00030	1	CGCACGAGAGAGAGCAACAGU	GCGUGCUCUCUGUCGCUGUCA	2	hypothetical protein
vvi-miR535c-3p	VIT_204s0023g01080	1	AUACUGUCGCUGUCUCUCGUG	UUUGACAGAGACAGAGAGCAU	2.5	type receptor kinase protein phosphorylation
vvi-miR535c-5p	VIT_200s0358g00010	1	CGCACGAGAGAGAGCAACAGU	GCGUGCUCUCUCUCGCUGUCA	1	hypothetical protein
vvi-miR535c-5p	VIT_207s0104g00030	1	CGCACGAGAGAGAGCAACAGU	GCGUGCUCUCUGUCGCUGUCA	2	hypothetical protein

Supplementary Table 4: Differentially expressed (DE) miRNA, between cultivars or vineyards, in each developmental stage. Sequence of each DE read, its match type, Fold change and FDR-corrected p-value are indicated. Highlighted in green are FDR <= 0.05.

The description of small RNA libraries codes are shown in Table 1.

Pea size

Small RNA - Name	sequence read	Match type	Mon_CS_ps vs Bol_CS_ps - FC	Mon_CS_ps vs Bol_CS_ps - FDR	Mon_CS_ps vs Ric_CS_ps - FC	Mon_CS_ps vs Ric_CS_ps - FDR	Bol_CS_ps vs Ric_CS_ps - FC	Bol_CS_ps vs Ric_CS_ps - FDR	Mon_SG_ps vs Bol_SG_ps - FC	Mon_SG_ps vs Bol_SG_ps - FDR	Mon_SG_ps vs Ric_SG_ps - FC	Mon_SG_ps vs Ric_SG_ps - FDR	Bol_SG_ps vs Ric_SG_ps - FC	Bol_SG_ps vs Ric_SG_ps - FDR	Mon_CS_ps vs Mon_SG_ps - FC	Mon_CS_ps vs Mon_SG_ps - FDR	Bol_CS_ps vs Bol_SG_ps - FC	Bol_CS_ps vs Bol_SG_ps - FDR	Ric_CS_ps vs Ric_SG_ps - FC	Ric_CS_ps vs Ric_SG_ps - FDR
vvi-miR166b	TCGGACCAGGCTTCATTCCTCT	Mature 3' super	1.865	1	1.686	1	-1.106	1	1.020	1	1.215	1	1.191	1	15.499	0.034	8.474	0.338	11.167	0.171
vvi-miR166b.f	GGAATGTTGGCTGGCTCGAG	Mature 5' sub	3.987	1	3.454	1	-1.155	1	-2.267	1	-1.428	1	1.588	1	55.620	0.015	6.153	0.945	11.281	0.182
vvi-miR166b.f	AATGTTGGCTGGCTCGAGG	Mature 5' sub	-4.292	1	-3.052	1	1.406	1	-1.130	1	-5.762	1	-5.100	1	5.733	1.000	21.782	0.038	3.036	1

vvi-miR319c	ATTGAATGATGC GGGAGACA	Precursor	-1.785	1	-2.864	1	-1.604	1	22.249	1	21.474	1	-1.036	1	-301.140	0.024	-7.581	0.945	-4.897	1
vvi-miR319c	ATTGAATGATGC GGGAGACAA	Precursor	-1.147	1	-2.648	1	-2.308	1	-1.200	1	2.127	1	2.552	1	-26.312	0.015	-27.515	0.007	-4.671	1
vvi-miR319c	ATTGAATGATGC GGGAGACAAA	Precursor	-1.826	1	-2.881	1	-1.578	1	1	1	8.284	1	8.284	1	-302.786	0.016	-165.840	0.037	-12.685	0.590
vvi-miR319c	AGCTGCCGACTC ATTCATCCA	Precursor	-1.014	1	-1.711	1	-1.687	1	-3.644	1	-1.775	1	2.053	1	-4.833	1.000	-17.367	0.007	-5.015	0.590
vvi-miR319c.b.f.g	TTGGACTGAAGG GAGCTCCC	Mature 3'	-1.598	1	-2.572	1	-1.610	1	1.879	1	3.631	1	1.933	1	-9.707	0.018	-3.234	1	-1.040	1
vvi-miR3627	CGCCGCTCTCCTG TGACAAG	Mature 3' sub	3.286	1	2.990	1	-1.099	1	5.321	1	1.191	1	-4.466	1	-5.389	1.000	-3.328	1	-13.524	0.013
vvi-miR3627	TCGCCGCTCTCCT GTGACAAG	Mature 3'	1.918	1	1.836	1	-1.045	1	3.808	1	-1.520	1	-5.787	1	-4.270	1.000	-2.151	1	-11.918	0.047
vvi-miR3636	GTCTGTCGGAGA AGCAAGTCGGA	Mature 3' sub/super	1	1	-1	1	-1	1	1.617	1	2.008	1	1.242	1	499.055	0.015	806.820	0.000	1001.916	6.42E-06
vvi-miR3636	GTCTGTCGGAGA AGCAAGTCGGAG	Mature 3' sub/super	1	1	-1	1	-1	1	-1.352	1	1.446	1	1.955	1	431.339	0.015	318.923	0.005	623.599	1.13E-04
vvi-miR3640	TGCCACGTGTGC AACTCAGGTCT	Precursor	-1.390	1	-1.173	1	1.185	1	-1.912	1	-1.677	1	1.140	1	-13.711	0.124	-18.865	0.014	-19.604	0.008
vvi-miRC477o	GTTGGAAGTCGG TGGGGGACC	Mature 3'	-1.280	1	2.728	1	3.490	1	135.440	0.039	436.027	2.83E-04	3.219	1	-114.994	0.016	1.507	1	1.390	1

19 °Brix

Small RNA - Name	sequence read	Match type	Mon_CS_19 vs Ric_CS_19 - FC	Mon_CS_19 vs Ric_CS_19 - FDR	Mon_CS_19 vs Bol_CS_19 - FC	Mon_CS_19 vs Bol_CS_19 - FDR	Ric_CS_19 vs Bol_CS_19 - FC	Ric_CS_19 vs Bol_CS_19 - FDR	Mon_SG_19 vs Ric_SG_19 - FC	Mon_SG_19 vs Ric_SG_19 - FDR	Mon_SG_19 vs Bol_SG_19 - FC	Mon_SG_19 vs Bol_SG_19 - FDR	Ric_SG_19 vs Bol_SG_19 - FC	Ric_SG_19 vs Bol_SG_19 - FDR	Mon_CS_19 vs Mon_SG_19 - FC	Mon_CS_19 vs Mon_SG_19 - FDR	Ric_CS_19 vs Ric_SG_19 - FC	Ric_CS_19 vs Ric_SG_19 - FDR	Bol_CS_19 vs Bol_SG_19 - FC	Bol_CS_19 vs Bol_SG_19 - FDR
m1191	TTGCTGAACAAGAGAGAACCT	Mature 3'	1.042	1	-1.246	1	-1.298	1	3.566	1	-6.093	0.995	-21.726	0.046	-2.477	1	1.381	1	-12.117	0.094
vvi-miR166d.c.e.f.g.h	GACCAGGCTTCATTCCCC	Mature 3' sub	-652.408	0.858	6.197	1	4042.935	0.027	-28.606	1	8.061	1	230.592	0.210	-2.280	1	10.003	1	-1.753	1
vvi-miR169r.t	GGCAAGTTGACTTGACTCAGT	Mature 3'	-2.793	1	-10.907	0.022	-3.905	0.633	9.305	0.052	7.366	0.143	-1.263	1	-15.752	0.007	1.650	1	5.100	0.167
vvi-miR3633b	GGAATGGGTGGCTGGGATCTA	Mature 5' super	2.502	1	-4.793	1	-11.993	0.044	2.239	1	-1.279	1	-2.865	1	1.859	1	1.664	1	6.965	0.323
vvi-miR3636	GTCTGTCGGAGAAGCAAGTCGGAG	Mature 3' sub/super	-1.000	1	1.000	1	1.000	1	-1.420	1	1.198	1	1.701	1	158.559	0.078	111.695	0.263	190.010	0.033
vvi-miR3640	AAAGGCATCATCAATCAGGTAATG	Mature 3'	1.474	1	-1.023	1	-1.509	1	2.621	1	-9.536	0.361	-24.995	0.046	-1.361	1	1.306	1	-12.681	0.139
vvi-miR3640	TGCCACGTGTGCAACTCAGGTCT	Precursor	-2.531	1	-1.860	1	1.361	1	5.056	1	1	1	-5.056	1	-263.528	0.012	-20.592	0.460	-141.669	0.075
vvi-miR3640	ACCTGATTGGTGATGCTTTTTTGG	Mature 5'	1.245	1	-16.310	0.670	-20.302	0.166	8.543	0.294	13.392	0.111	1.568	1	-1.974	1	3.477	1	110.641	0.001
vvi-miR395c.e.h.k	GTTCCCTTGACCACTTCACTG	Mature 5'	1.210	1	4.047	1	3.345	1	1.717	1	-1.063	1	-1.825	1	-5.348	0.676	-3.768	1	-23.007	0.002
vvi-miR395f.i.j.l	GTTCCCCTGACCACTTCACTG	Mature 5'	1.771	1	4.664	1	2.634	1	1.725	1	-1.604	1	-2.767	1	-4.581	1	-4.703	1	-34.267	0.032

vvi-miR396a	TTCCACAGCTTTC TTGAACTA	Mature 5'	-2.723	1	-1.095	1	2.487	1	1.343	1	-4.834	0.547	-6.493	0.340	-2.524	1	1.449	1	-11.146	0.039
vvi-miR399a	GTGTGATTCTCCT TTGGCAGA	Mature 5'	7.169	1	-35.482	1	-254.354	0.044	213.505	0.081	170.749	0.145	-1.250	1	-35.482	1	-1.191	1	170.749	0.094
vvi-miR399a.h	TGCCAAAGGAGA ATTGCCCTG	Mature 3'	6.398	1	-1.717	1	-10.987	0.458	766.233	0.005	141.756	0.148	-5.405	1	-116.992	0.364	1.024	1	2.081	1
vvi-miR399b.c	TGCCAAAGGAGA GTTGCCCTG	Mature 3'	3.068	1	-16.128	0.265	-49.488	0.014	119.217	0.009	9.384	1	-12.704	0.404	-141.705	0.012	-3.647	1	1.068	1
vvi-miR399i	CGCCAAAGGAGA GTTGCCC	Mature 3' sub	1.184	1	-1.826	1	-2.162	1	77.202	0.005	35.833	0.111	-2.155	1	-47.629	0.035	1.369	1	1.373	1
vvi-miR399i	CGCCAAAGGAGA GTTGCCCTG	Mature 3'	1.823	1	-1.209	1	-2.204	1	47.194	0.005	18.231	0.111	-2.589	1	-19.659	0.088	1.317	1	1.122	1
vvi-miR408	CGGGGACGAGGT AGTGCA	Mature 5' sub	2.431	1	-1.811	1	-4.402	1	1.510	1	7.223	0.481	4.783	0.931	2.170	1	1.349	1	28.390	0.033
vvi-miRC477i.c.l.m	AAAGTTGGAAGC CGGTGGGGGACC	Mature 3' super	1.157	1	-38.975	0.260	-45.088	0.044	2.386	1	-1.014	1	-2.420	1	-3.947	1	-1.914	1	9.735	1
vvi-miRC477i.c.l.m	GTTGGAAGCCGG TGGGGGAC	Mature 3' sub	3.028	1	-10.308	0.829	-31.215	0.044	12.577	0.352	3.891	1	-3.232	1	-4.535	1	-1.092	1	8.846	0.377
vvi-miRC477i.c.l.m	GTTGGAAGCCGG TGGGGGACC	Mature 3'	2.774	1	-13.146	0.265	-36.472	0.011	3.974	1	1.979	1	-2.008	1	-3.157	1	-2.204	1	8.241	0.270
vvi-miRC477i.c.l.m	TTGGAAGCCGGT GGGGGACC	Mature 3' sub	5.817	1	-13.890	0.260	-80.795	1.94E-04	7.745	0.447	12.157	0.118	1.570	1	-2.798	1	-2.101	1	60.354	0.001

vvi-miRC477j	CTGGAAGCCGATGGGGGACC	Mature 3' sub	1.895	1	-10.209	0.265	-19.344	0.027	5.479	0.847	5.330	0.675	-1.028	1	-5.822	0.820	-2.013	1	9.346	0.139
vvi-miRC477o	TTGGAAGTCGGTGGGGGACC	Mature 3' sub	10.685	0.858	-11.784	1	-125.917	3.77E-04	13.118	0.372	2.902	1	-4.520	1	-3.133	1	-2.552	1	10.915	1
vvi-miRC482a	GAATGGGCTGATTGGGATA	Mature 5' sub	6.697	1	-2.739	1	-18.346	0.027	3.550	1	-22.608	1	-80.265	0.518	-1.926	1	-3.633	1	-15.894	1
vvi-miRC482a	AATGGGCTGATTGGGATAAAA	Mature 5' sub/super	1.945	1	1.683	1	-1.156	1	2.124	1	-2.302	1	-4.891	0.475	-2.941	1	-2.692	1	-11.392	0.033
vvi-miRC482b	ATGGGCGGTTTGGTAAGAGGGAA	Mature 5' sub/super	3.115	1	1.570	1	-1.984	1	1.014	1	-82.557	0.523	-83.727	0.475	-1.482	1	-4.551	1	-192.089	0.039
vvi-miRC530a.b	TCTGCATTTGCACCTGCACCT	Mature 5'	-1.073	1	-4.155	1	-3.873	1	37.392	0.005	17.855	0.111	-2.094	1	-16.850	0.078	2.381	1	4.403	0.793

Harvest

Small RNA - Name	Sequence read	Match type	Mon_CS_hv vs Bol_CS_hv - FC	Mon_CS_hv vs Bol_CS_hv - FDR	Mon_CS_hv vs Ric_CS_hv - FC	Mon_CS_hv vs Ric_CS_hv - FDR	Bol_CS_hv vs Ric_CS_hv - FC	Bol_CS_hv vs Ric_CS_hv - FDR	Mon_SG_hv vs Bol_SG_hv - FC	Mon_SG_hv vs Bol_SG_hv - FDR	Mon_SG_hv vs Ric_SG_hv - FC	Mon_SG_hv vs Ric_SG_hv - FDR	Bol_SG_hv vs Ric_SG_hv - FC	Bol_SG_hv vs Ric_SG_hv - FDR	Mon_CS_hv vs Mon_SG_hv - FC	Mon_CS_hv vs Mon_SG_hv - FDR	Bol_CS_hv vs Bol_SG_hv - FC	Bol_CS_hv vs Bol_SG_hv - FDR	Ric_CS_hv vs Ric_SG_hv - FC	Ric_CS_hv vs Ric_SG_hv - FDR
m1188	CCCGAGAGGACTTAGTGGATC	Mature 3'	-1.087	1	-1.970	1	-1.812	1	2.050	1	1.990	1	-1.030	1	-26.436	0.006	-11.863	0.047	-6.743	0.332
m1355	GCCGCGTTGGAGCAGGAGCTT	Mature 5'	-1.676	1	-1.324	1	1.266	1	96.267	0.143	49.660	1	-1.939	1	-181.578	0.048	-1.125	1	-2.761	1
vvi-miR156e	TGACAGAGGAGAGTGAGCAC	Mature 5'	14.931	1	20.917	1	1.401	1	19.448	0.075	39.969	0.016	2.055	1	8.689	1	11.318	0.178	16.604	0.033
vvi-miR166d.c.e.f.g.h	CGGACCAGGCTTCATTCCCC	Mature 3' sub	-5.661	0.832	3.211	1	18.180	0.040	-18.239	0.016	1.227	1	22.373	0.031	2.572	1	-1.253	1	-1.018	1

vvi-miR166d.c.e.f.g.h	GACCAGGCTTCATTCCCC	Mature 3' sub	3.915	1	-172.647	0.696	-675.876	0.021	1457.719	0.009	9.170	1	-158.958	0.196	-454.179	0.100	-1.220	1	3.486	1
vvi-miR3634	TTCCGACTCGCACTCATGCCGT	Mature 3' sub	1.559	1	2.467	1	1.583	1	-6.318	0.017	-2.020	1	3.127	1	3.600	0.311	-2.735	0.937	-1.384	1
vvi-miR3636	GTCTGTCGGAGAAGCAAGTCGGAG	Mature 3' sub/super	1.000	1	1.000	1	1.000	1	1.780	1	1.219	1	-1.460	1	164.641	0.046	292.992	3.67E-04	200.675	0.011
vvi-miR3640	ACCTGATTGGTGATGCTTTTTTGG	Mature 5'	-5.351	1	-1.870	1	2.861	1	6.335	0.928	8.954	0.575	1.413	1	-1.415	1	23.958	0.048	11.837	0.037
vvi-miR3640	ACCCGAAGCTGTACACGTGACACC	Precursor	1.209	1	-2.169	1	-2.623	1	1.455	1	1.597	1	1.098	1	5.125	0.876	6.167	0.380	17.760	0.033
vvi-miR393a.b	TCCAAAGGGATCGCATTGATCC	Mature 5' super	3.435	1	1.636	1	-2.100	1	-158.700	0.053	-8.257	0.673	19.220	1	1.311	1	-415.633	3.67E-04	-10.300	0.309
vvi-miR395c.e.h.k	GTTCCCTTGACCACTTCACTG	Mature 5'	6.416	0.215	1.487	1	-4.315	0.933	-1.137	1	-4.222	1	-3.713	1	-2.154	1	-15.711	0.002	-13.520	0.023
vvi-miR395f.i.j.l	GTTCCCCTGACCACTTCACTG	Mature 5'	11.986	0.016	3.458	1	-3.466	1	1.945	1	-2.668	1	-5.190	1	-2.764	1	-17.034	0.002	-25.504	0.011
vvi-miR395f.i.j.l.c.e.h.k.a.d	CTGAAGTGTTTGGGGGAACT	Mature 3' sub	2.363	1	2.134	1	-1.107	1	9.740	0.034	-4.305	1	-41.932	0.054	-1.378	1	2.991	1	-12.666	1
vvi-miR395f.i.j.l.c.e.h.k.a.d	CTGAAGTGTTTGGGGGAACTC	Mature 3'	2.181	1	2.777	1	1.273	1	12.922	0.003	-2.307	1	-29.809	3.10E-04	-1.282	1	4.621	0.307	-8.211	0.100
vvi-miR395f.i.j.l.c.e.h.k.a.d	TGAAGTGTTTGGGGAACTC	Mature 3' sub	4.533	1	29.538	1	6.516	1	106.882	9.57E-05	-4.930	1	-526.875	3.10E-04	4.930	1	116.229	1.97E-05	-29.538	1

vvi-miR399a.h	TGCCAAAGGAGAATTGCCCTG	Mature 3'	-2.380	1	2.600	1	6.189	1	544.849	0.028	-1.000	1	-544.849	0.031	-283.806	0.066	4.569	1	-737.985	0.011
vvi-miR399b.c	TGCCAAAGGAGAGTTGCCCTG	Mature 3'	-11.705	0.134	-1.372	1	8.529	0.360	523.182	0.003	48.819	1	-10.717	0.375	-2256.725	7.22E-06	2.714	1	-33.681	0.011
vvi-miR399i	CGCCAAAGGAGAGTTGCCC	Mature 3' sub	-1.420	1	-2.447	1	-1.724	1	90.902	0.001	9.909	1	-9.174	0.355	-150.616	7.50E-05	-1.167	1	-6.212	0.653
vvi-miR399i	CGCCAAAGGAGAGTTGCCCT	Mature 3' sub	-1.705	1	-1.774	1	-1.041	1	850.552	0.001	84.738	0.578	-10.037	0.539	-792.070	0.001	1.830	1	-5.268	1
vvi-miR399i	CGCCAAAGGAGAGTTGCCCTG	Mature 3'	-1.713	1	-1.868	1	-1.090	1	100.763	1.93E-07	11.745	0.032	-8.579	0.192	-108.047	3.17E-07	1.597	1	-4.925	0.563
vvi-miR399i.b.c	GCCAAAGGAGAGTTGCCCTG	Mature 3' sub	-5.581	1	1.806	1	10.076	0.273	21.734	0.365	1.352	1	-16.076	0.539	-21.247	0.311	5.709	0.885	-28.376	0.037
vvi-miR477b	GAAGCTCTTGAGGGGGACT	Precursor	-2.288	1	-2.551	1	-1.115	1	35.091	0.001	13.676	0.163	-2.566	1	-4.304	1	18.650	0.008	8.107	0.332
vvi-miR477b	CGAAGTCTTTGGGGAGAGTGG	Mature 3'	-7.649	0.215	-1.639	1	4.667	1	1.616	1	5.465	0.666	3.382	1	-11.617	0.046	1.064	1	-1.297	1
vvi-miRC477i.c.l.m.p	GTTGGAAGCCGGTGGGGGAC	Mature 3' sub	-7.515	0.011	1.234	1	9.272	0.001	29.015	7.94E-09	23.614	1.17E-07	-1.229	1	-8.968	0.002	24.312	1.69E-08	2.134	1
vvi-miRC477i.c.l.m.p	TTGGAAGCCGGTGGGGGACC	Mature 3' sub	-13.117	0.041	-3.145	1	4.171	1	5.494	0.428	4.751	0.858	-1.156	1	-4.388	0.748	16.422	0.005	3.405	1
vvi-miRC477i.c.l.m.p	GTTGGAAGCCGGTGGGGGACC	Mature 3'	-10.085	0.098	-1.916	1	5.264	0.877	14.805	0.008	12.220	0.032	-1.212	1	-12.422	0.026	12.020	0.018	1.885	1
vvi-miRC477i.c.l.m.	TCCCTCAAAGGCTTCCAATTT	Mature 5'	-13.023	0.113	-1.002	1	13.001	0.069	5.745	0.211	3.025	1	-1.899	1	-4.450	0.459	16.813	0.018	-1.468	1

p.n.o.k																				
vvi-miRC477j	CTGGAAGCCGATGGGGGACC	Mature 3' sub	-14.248	0.009	-2.320	1	6.140	0.210	3.433	0.873	3.677	0.976	1.071	1	-7.961	0.046	6.145	0.102	1.072	1
vvi-miRC477j	GCTGGAAGCCGATGGGGGACC	Mature 3'	-5.096	0.431	-2.175	1	2.343	1	8.728	0.028	9.910	0.032	1.136	1	-6.402	0.131	6.948	0.079	3.367	1
vvi-miRC477j	GCTGGAAGCCGATGGGGGAC	Mature 3' sub	-29.881	1	1.786	1	53.355	0.887	28.516	0.470	11.355	1	-2.511	1	-4.989	1	170.806	0.013	1.275	1
vvi-miRC477n	GTTGGAAGTCGGTGGGGGAAC	Mature 3'	-13.676	0.012	2.439	1	33.350	1.85E-05	735.740	1.81E-16	172.569	2.15E-10	-4.263	0.355	-17.615	0.005	571.213	5.28E-17	4.017	0.332
vvi-miRC477n	AGTTGGAAGTCGGTGGGGGAAC	Mature 3' super	-31.297	1	4.521	1	141.499	0.021	269.467	1.25E-10	99.396	3.13E-07	-2.711	1	-3.557	1	2370.884	4.11E-13	6.180	0.037
vvi-miRC477o	GTTGGAAGTCGGTGGGGGAC	Mature 3' sub	-60.460	0.689	1.307	1	79.001	0.251	212.953	0.008	40.271	1	-5.288	0.797	-60.460	0.472	212.953	0.002	-1.962	1
vvi-miRC477o	GTTGGAAGTCGGTGGGGGACC	Mature 3'	-7.608	0.113	-1.107	1	6.871	0.197	255.392	2.00E-09	154.625	1.01E-07	-1.652	1	-138.351	3.17E-07	14.044	0.003	1.237	1
vvi-miRC482a	GAATGGGCTGATTGGGATA	Mature 5' sub	-21.691	1	7.084	1	153.658	0.040	-1.144	1	2.177	1	2.491	1	1.046	1	19.829	1	-3.111	1

[a] : Match type indicates the similarity between sequencing reads and annotaed 3p or 5p miRNA. Super : when the reads is longer, sub: when reads are shorter. Precursor when the reads is shifted more than 5 nt respect to the mature sequence (3p or 5p).

FC: Fold change. In the comparison Library A vs Library B, it is positive when Lib B> LibA (FC = Lib B/ Lib A), it is negative when Lib B < Lib A (FC= - LibA/ Lib B)

FDR: corrected p-value, FDR
